# Supplementary material for: Oxidation of difluorocarbene and subsequent trifluoromethoxylation
Source: Nat Commun. 2019 Nov 25;10:5362. doi: 10.1038/s41467-019-13359-z (PMC6877537; doi:10.1038/s41467-019-13359-z)
Supplement: Supplementary file 2 — Supplementary Information [file 41467_2019_13359_MOESM2_ESM.pdf]

Supplementary Information

Oxidation of difluorocarbene and subsequent trifluoromethoxylation

Xiao et al.

Supplementary Methods

General information

^{1}H, ^{13}C and ^{19}F NMR spectra were detected on a 500 MHz, 400 MHz or 300 MHz NMR spectrometer. Data for ^{1}H NMR, ^{13}C NMR and ^{19}F NMR were recorded as follows: chemical shift (δ, ppm), multiplicity (s = singlet, d = doublet, t = triplet, m = multiplet, q = quartet, coupling constants in Hz). Mass spectra were obtained on GC-MS. High resolution mass data were recorded on a high resolution mass spectrometer in the EI mode. The mass analyzer type for HRMS-EI is time-of-flight mass spectrometer. The substrate **1-22**[1] was prepared according to the previous procedure. Unless otherwise noted, all reagents were obtained commercially and used without further purification.

Typical procedures for the preparation of substrate 1

Substrates **1-19** and **1-21** were prepared according to the reported procedure.[2]

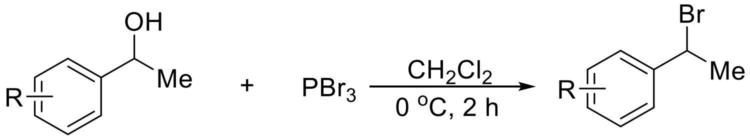

PBr_3 (0.66 mL, 7.0 mmol) in CH_2Cl_2 (5 mL) was added to a solution of 1-([1,1'-biphenyl]-4-yl)ethanol (1.0 g, 5.0 mmol) in CH_2Cl_2 (5.0 mL) at 0 °C. The resultant solution was stirred at 0 °C for 2 hours, then H_2O (10 mL) was added and the resultant mixture was stirred for 5 minutes. The layers were separated and the aqueous layer was extracted with CH_2Cl_2 (3 × 50 mL) and the combined organic extracts were washed sequentially with saturated $NaHCO_3$(aq) (50 mL) and brine (50 mL), dried and concentrated in vacuo. The residue was dissolved in Et_2O and filtered through a PTFE Acrodisc® syringe filters, and the resulting solution was concentrated under vacuum to yield compound **1-19** (1.1 g, 4.2 mmol) in 84% yield.

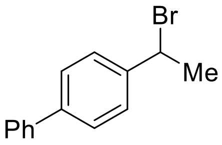

1-19

4-(1-bromoethyl)-1,1'-biphenyl[2]: 84%; ^{1}H NMR (400 MHz, $CDCl_3$) δ 7.62 – 7.55 (m, 4H), 7.52 (d, *J* = 8.3 Hz, 2H), 7.45 (t, *J* = 7.5 Hz, 2H), 7.36 (t, *J* = 7.3 Hz, 1H), 5.28 (q, *J* = 6.9 Hz, 1H), 2.10 (d,

J = 6.9 Hz, 3H); ^{13}C NMR (101 MHz, $CDCl_3$) δ 142.2 (s), 141.3 (s), 140.5 (s), 128.8 (s), 127.5 (s), 127.4 (s), 127.3 (s), 127.1 (s), 49.4 (s), 26.7 (s).

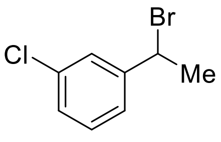

1-21

1-(1-bromoethyl)-3-chlorobenzene[3]: 62%; ^{1}H NMR (400 MHz, $CDCl_3$) δ 7.41 (s, 1H), 7.32 – 7.26 (m, 1H), 7.26 – 7.20 (m, 2H), 5.12 (q, J = 6.9 Hz, 1H), 2.01 (d, J = 6.9 Hz, 3H); ^{13}C NMR (101 MHz, $CDCl_3$) δ 145.1 (s), 134.4 (s), 129.9 (s), 128.4 (s), 127.0 (s), 125.0 (s), 47.8 (s), 26.6 (s).

Substrates **1-24, 1-25, 1-26** and **1-27** were prepared according to the reported procedure.[4]

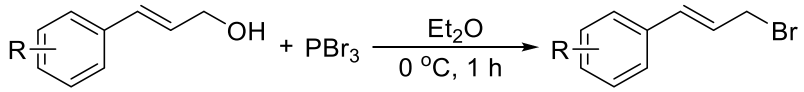

To a stirred solution of (E)-3-(4-chlorophenyl)prop-2-en-1-ol (0.843 g, 5.0 mmol) in Et_2O (50 mL) at 0 °C, PBr_3 (0.5 mL, 6.0 mmol) was added dropwise. The reaction was stirred for 1 h at 0 °C, and quenched by the saturated $NaHCO_3$ solution. The reaction mixture was allowed to warm to room temperature. The residue was then extracted twice with CH_2Cl_2. The combined organic layers were washed with brine, dried over $MgSO_4$, and concentrated in vacuo to give **1-24** (0.483 g, 2.1 mmol, 42% yield) without further purification.

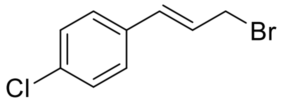

1-24

(E)-1-(3-bromoprop-1-en-1-yl)-4-chlorobenzene[5]: 42%; ^{1}H NMR (400 MHz, $CDCl_3$) δ 7.32 – 7.25 (m, 4H), 6.58 (d, J = 15.6 Hz, 1H), 6.35 (dt, J = 15.6, 7.7 Hz, 1H), 4.12 (dd, J = 7.7, 0.8 Hz, 2H); ^{13}C NMR (101 MHz, $CDCl_3$) δ 134.3 (s), 134.0 (s), 133.2 (s), 128.8 (s), 127.9 (s), 125.8 (s), 33.0 (s).

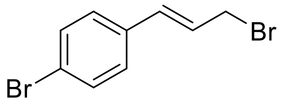

1-25

(E)-1-bromo-4-(3-bromoprop-1-en-1-yl)benzene[6]: 43%; ^{1}H NMR (400 MHz, $CDCl_3$) δ 7.45 (d, J = 8.4 Hz, 2H), 7.24 (d, J = 8.4 Hz, 2H), 6.57 (d, J = 15.6 Hz, 1H), 6.38 (dt, J = 15.6, 7.7 Hz, 1H),

4.13 (d, *J* = 7.7 Hz, 1H); ^{13}C NMR (101 MHz, $CDCl_3$) δ 134.7 (s), 133.2 (s), 131.8 (s), 128.2 (s), 126.0 (s), 122.2 (s), 32.9 (s).

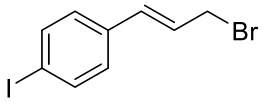

1-26

(*E*)-1-(3-bromoprop-1-en-1-yl)-4-iodobenzene[4]: 40%; ^{1}H NMR (400 MHz, $CDCl_3$) δ 7.66 (d, *J* = 8.4 Hz, 2H), 7.12 (d, *J* = 8.3 Hz, 2H), 6.56 (d, *J* = 15.6 Hz, 1H), 6.40 (dt, *J* = 15.6, 7.7 Hz, 1H), 4.13 (dd, *J* = 7.7, 0.7 Hz, 2H); ^{13}C NMR (101 MHz, $CDCl_3$) δ 137.7 (s), 135.3 (s), 133.4 (s), 128.4 (s), 126.1 (s), 93.8 (s), 32.9 (s).

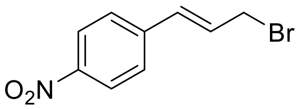

1-27

(*E*)-1-(3-bromoprop-1-en-1-yl)-4-nitrobenzene[4]: 50%; ^{1}H NMR (400 MHz, $CDCl_3$) δ 8.20 (d, *J* = 8.8 Hz, 2H), 7.53 (d, *J* = 8.8 Hz, 2H), 6.71 (d, *J* = 15.7 Hz, 1H), 6.57 (dt, *J* = 15.7, 7.6 Hz, 1H), 4.16 (dd, *J* = 7.6, 0.8 Hz, 1H); ^{13}C NMR (101 MHz, $CDCl_3$) δ 147.4 (s), 142.2 (s), 132.1 (s), 129.8 (s), 127.3 (s), 124.0 (s), 31.8 (s).

Substrates **1-30** and **1-32** were prepared according to the reported procedure.[7]

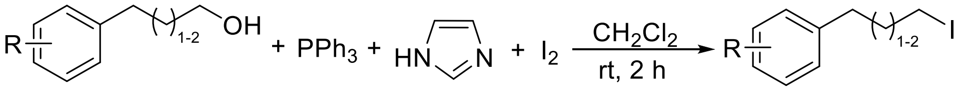

A solution of triphenylphosphine (1.73 g, 6.6 mmol) and I_2 (1.68 g, 6.6 mmol) in dichloromethane (30 mL) was allowed to stir for 10 min at ambient temperature. Imidazole (0.75 g, 11.0 mmol) was added and the resulting mixture was stirred for 10 min. 4-Phenylbutan-1-ol (0.66 g, 4.41 mmol) was added and the resulting mixture was stirred for 2 h. The mixture was quenched by saturated sodium metabisulfite (20 mL). The aqueous and organic layers were separated followed by extraction of the aqueous with dichloromethane (3 x 10 mL). The combined organic extracts were dried over $MgSO_4$ and concentrated under reduced pressure. The resulting residue was purified by flash chromatography to afford compound 1-30 (1.1 g, 96%) as a colorless oil.

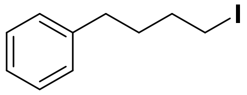

1-30

(4-iodobutyl)benzene[7]: 96%; ^{1}H NMR (400 MHz, $CDCl_3$) δ 7.28 (t, *J* = 7.4 Hz, 2H), 7.22 – 7.12 (m, 3H), 3.19 (t, *J* = 6.9 Hz, 2H), 2.63 (t, *J* = 7.5 Hz, 2H), 1.91 – 1.79 (m, 2H), 1.79 – 1.63 (m, 2H); ^{13}C NMR (101 MHz, $CDCl_3$) δ 141.8 (s), 128.4 (s), 125.9 (s), 34.7 (s), 32.9 (s), 32.2 (s), 6.7 (s).

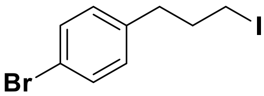

1-32

1-bromo-4-(3-iodopropyl)benzene[8]: 98%; ^{1}H NMR (400 MHz, $CDCl_3$) δ 7.39 (d, *J* = 8.4 Hz, 2H), 7.06 (d, *J* = 8.4 Hz, 2H), 3.13 (t, *J* = 6.8 Hz, 2H), 2.67 (t, *J* = 7.3 Hz, 2H), 2.14 – 2.00 (m, 2H); ^{13}C NMR (101 MHz, $CDCl_3$) δ 139.3 (s), 131.5 (s), 130.3 (s), 119.9 (s), 35.5 (s), 34.5 (s), 5.9 (s).

Typical procedure for trifluoromethoxylation of benzyl bromides

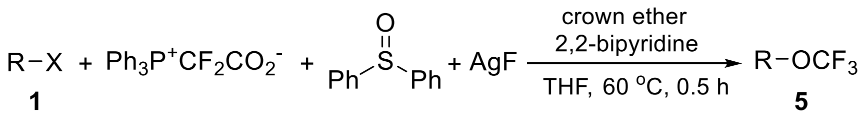

Into a 20 mL sealed tube were added benzyl bromide **1-1** (0.8 mmol, 197.7 mg, 1.0 equiv), $Ph_3P^+CF_2CO_2^-$ (2.0 mmol, 712.0 mg, 2.5 equiv), Ph_2S=O (2.0 mmol, 404.6 mg, 2.5 equiv), AgF (1.6 mmol, 203.2 mg, 2.0 equiv), 2,2'-bipyridine (1.2 mmol, 187.4 mg, 1.5 equiv), 2,3,11,12-dibenzo-18-crown-6 (0.4 mmol, 144.2 mg, 0.5 equiv) and THF (6 mL) under a N_2 atmosphere. The tube was sealed and the reaction mixture was stirred at 60 °C for 30 min. After the mixture was cooled to room temperature, the pure product was isolated by flash column chromatography.

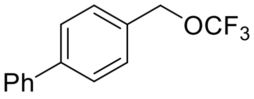

5-1

4-((trifluoromethoxy)methyl)-1,1'-biphenyl[9]: 71%; ^{1}H NMR (400 MHz, $CDCl_3$) δ 7.67 – 7.52 (m, 4H), 7.51 – 7.40 (m, 4H), 7.37 (t, *J* = 7.3 Hz, 1H), 5.03 (s, 2H); ^{19}F NMR (376 MHz, $CDCl_3$) δ -60.31 (s, 3F); ^{13}C NMR (101 MHz, $CDCl_3$) δ 142.0 (s), 140.5 (s), 132.8 (s), 128.9 (s), 128.6 (s), 127.7 (s), 127.5 (s), 127.2 (s), 121.7 (q, *J* = 255.3 Hz), 68.9 (q, *J* = 3.5 Hz).

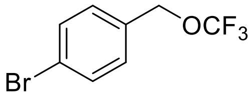

5-2

1-bromo-4-((trifluoromethoxy)methyl)benzene[9]: 68%; ^{1}H NMR (400 MHz, $CDCl_3$) δ 7.51 (d, *J* = 8.3 Hz, 2H), 7.23 (d, *J* = 8.3 Hz, 2H), 4.92 (s, 2H); ^{19}F NMR (376 MHz, $CDCl_3$) δ -60.50 (s, 3F); ^{13}C NMR (101 MHz, $CDCl_3$) δ 132.9 (s), 131.9 (s), 129.6 (s), 123.1 (s), 121.6 (q, *J* = 255.9 Hz), 68.3 (q, *J* = 3.5 Hz).

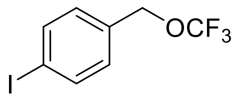

5-3

1-iodo-4-((trifluoromethoxy)methyl)benzene[9]: 65%; ^{1}H NMR (400 MHz, $CDCl_3$) δ 7.72 (d, *J* = 8.3 Hz, 2H), 7.09 (d, *J* = 8.3 Hz, 2H), 4.90 (s, 2H); ^{19}F NMR (376 MHz, $CDCl_3$) δ -60.50 (s, 3F); ^{13}C NMR (101 MHz, $CDCl_3$) δ 137.9 (s), 133.5 (s), 129.7 (s), 121.6 (q, *J* = 256.0 Hz), 94.7 (s), 68.4 (q, *J* = 3.6 Hz).

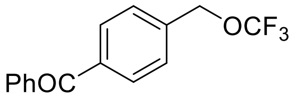

5-4

phenyl(4-((trifluoromethoxy)methyl)phenyl)methanone[10]: 68%; ^{1}H NMR (400 MHz, $CDCl_3$) δ 7.85 – 7.75 (m, 4H), 7.59 (tt, *J* = 6.8, 1.2 Hz, 1H), 7.51 – 7.41 (m, 4H), 5.06 (s, 2H); ^{19}F NMR (376 MHz, $CDCl_3$) δ -60.59 (s, 3F); ^{13}C NMR (101 MHz, $CDCl_3$) δ 196.1 (s), 138.2 (s), 138.0 (s), 137.3 (s), 132.7 (s), 130.4 (s), 130.1 (s), 128.4 (s), 127.5 (s), 121.7 (q, *J* = 255.9 Hz), 68.3 (q, *J* = 3.4 Hz).

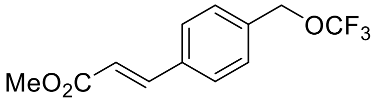

5-5

methyl (*E*)-3-(4-((trifluoromethoxy)methyl)phenyl)acrylate: 71%; White solid. M.P. 52.0-52.5 °C; ^{1}H NMR (400 MHz, $CDCl_3$) δ 7.66 (d, *J* = 16.0 Hz, 1H), 7.52 (d, *J* = 8.2 Hz, 2H), 7.36 (d, *J* = 8.2 Hz, 2H), 6.43 (d, *J* = 16.0 Hz, 1H), 4.97 (s, 2H), 3.79 (s, 3H); ^{19}F NMR (376 MHz, $CDCl_3$) δ -60.53 (s, 3F); ^{13}C NMR (101 MHz, $CDCl_3$) δ 167.2 (s), 143.9 (s), 135.9 (s), 135.0 (s), 128.33 (s), 128.31 (s), 121.7 (q, *J* = 256.0 Hz), 118.7 (s), 68.4 (q, *J* = 3.5 Hz), 51.7 (s); IR (neat) ν = 2960, 2849, 1716,

1325, 1292, 1205, 1175, 1144, 992, 824, 802 cm^{-1}; HRMS (EI) Calcd for $C_{12}H_{11}F_3O_3$ $[M]^+$: 260.0660, Found: 260.0663.

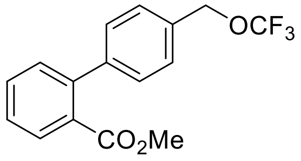

5-6

methyl 4'-((trifluoromethoxy)methyl)-[1,1'-biphenyl]-2-carboxylate: 72%; Slightly yellow liquid; ^1H NMR (400 MHz, $CDCl_3$) δ 7.86 (dd, *J* = 7.7, 1.0 Hz, 1H), 7.53 (td, *J* = 7.5, 1.3 Hz, 1H), 7.45 – 7.37 (m, 3H), 7.37 – 7.31 (m, 3H), 5.03 (s, 2H), 3.65 (s, 3H); ^{19}F NMR (376 MHz, $CDCl_3$) δ -60.30 (s, 3F); ^{13}C NMR (101 MHz, $CDCl_3$) δ 168.8 (s), 142.2 (s), 142.0 (s), 132.8 (s), 131.5 (s), 130.8 (s), 130.7 (s), 130.0 (s), 128.8 (s), 127.8 (s), 127.5 (s), 121.8 (q, *J* = 255.4 Hz), 69.0 (q, *J* = 3.5 Hz), 52.0 (s); IR (neat) ν = 2953, 1728, 1448, 1281, 1205, 1140, 1089, 1048, 827, 763, 734 cm^{-1}; HRMS (EI) Calcd for $C_{16}H_{13}F_3O_3$ $[M]^+$: 310.0817, Found: 310.0819.

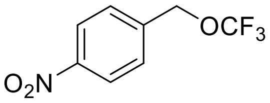

5-7

1-nitro-4-((trifluoromethoxy)methyl)benzene[9]: 62%; ^1H NMR (400 MHz, $CDCl_3$) δ 8.23 (d, *J* = 8.7 Hz, 2H), 7.53 (d, *J* = 8.7 Hz, 2H), 5.08 (s, 1H); ^{19}F NMR (376 MHz, $CDCl_3$) δ -60.86 (s, 3F); ^{13}C NMR (101 MHz, $CDCl_3$) δ 148.1 (s), 140.9 (s), 128.1 (s), 123.9 (s), 121.6 (q, *J* = 256.3 Hz), 67.4 (q, *J* = 3.6 Hz).

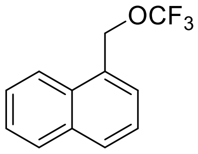

5-8

1-((trifluoromethoxy)methyl)naphthalene[9]: 47%; ^1H NMR (400 MHz, $CDCl_3$) δ 8.02 (d, *J* = 8.4 Hz, 1H), 7.90 (d, *J* = 7.9 Hz, 2H), 7.69 – 7.51 (m, 3H), 7.51 – 7.42 (m, 1H), 5.45 (s, 2H); ^{19}F NMR (376 MHz, $CDCl_3$) δ -60.36 (s, 3F); ^{13}C NMR (101 MHz, $CDCl_3$) δ 133.8 (s), 131.4 (s), 130.1 (s), 129.3

(s), 128.8 (s), 127.6 (s), 127.0 (s), 126.2 (s), 125.2 (s), 123.1 (s), 121.8 (q, J = 256.0 Hz), 67.6 (q, J = 3.5 Hz).

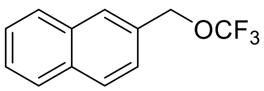

5-9

2-((trifluoromethoxy)methyl)naphthalene[11]: 58%; ^{1}H NMR (400 MHz, $CDCl_3$) δ 7.94 – 7.73 (m, 4H), 7.58 – 7.50 (m, 2H), 7.46 (dd, J = 8.5, 1.6 Hz, 1H), 5.14 (s, 2H); ^{19}F NMR (376 MHz, $CDCl_3$) δ -60.23 (s, 3F); ^{13}C NMR (101 MHz, $CDCl_3$) δ 133.4 (s), 133.2 (s), 131.3 (s), 128.7 (s), 128.1 (s), 127.8 (s), 127.4 (s), 126.7 (s), 126.6 (s), 125.3 (s), 121.8 (q, J = 255.6 Hz), 69.3 (q, J = 3.5 Hz).

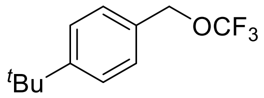

5-10

1-(tert-butyl)-4-((trifluoromethoxy)methyl)benzene[9]: 70%; ^{1}H NMR (400 MHz, $CDCl_3$) δ 7.44 (d, J = 8.4 Hz, 2H), 7.32 (d, J = 8.4 Hz, 2H), 4.97 (s, 2H), 1.35 (s, 9H); ^{19}F NMR (376 MHz, $CDCl_3$) δ -60.33 (s, 3F); ^{13}C NMR (101 MHz, $CDCl_3$) δ 152.2 (s), 130.9 (s), 128.1 (s), 125.7 (s), 121.7 (q, J = 255.2 Hz), 69.1 (q, J = 3.5 Hz), 34.7 (s), 31.3 (s).

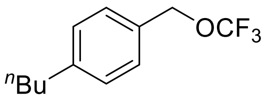

5-11

1-butyl-4-((trifluoromethoxy)methyl)benzene: 68%; Colourless liquid; ^{1}H NMR (400 MHz, $CDCl_3$) δ 7.29 (d, J = 8.1 Hz, 2H), 7.22 (d, J = 8.1 Hz, 2H), 4.96 (s, 2H), 2.64 (t, J = 7.7 Hz, 2H), 1.68 – 1.56 (m, 2H), 1.44 – 1.29 (m, 2H), 0.95 (t, J = 7.3 Hz, 3H); ^{19}F NMR (376 MHz, $CDCl_3$) δ -60.31 (s, 3F); ^{13}C NMR (101 MHz, $CDCl_3$) δ 144.0 (s), 131.1 (s), 128.8 (s), 128.3 (s), 121.7 (q, J = 255.3 Hz), 69.2 (q, J = 3.3 Hz), 35.4 (s), 33.6 (s), 22.4 (s), 13.9 (s); IR (neat) ν = 2960, 2932, 2860, 1270, 1231, 1207, 1145, 909, 821, 735, 650 cm^{-1}; HRMS (EI) Calcd for $C_{12}H_{15}F_3O$ $[M]^+$: 232.1075, Found: 232.1070.

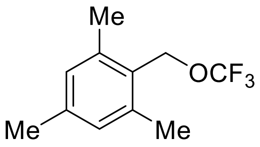

5-12

1,3,5-trimethyl-2-((trifluoromethoxy)methyl)benzene: 42%; Colourless liquid; ^{1}H NMR (400 MHz, $CDCl_3$) δ 6.90 (s, 2H), 5.06 (s, 2H), 2.37 (s, 6H), 2.28 (s, 3H); ^{19}F NMR (376 MHz, $CDCl_3$) δ -60.72 (s, 3F); ^{13}C NMR (101 MHz, $CDCl_3$) δ 139.3 (s), 138.4 (s), 129.3 (s), 127.0 (s), 121.7 (q, *J* = 255.4 Hz), 63.7 (q, *J* = 3.4 Hz), 21.0 (s), 19.1 (s); IR (neat) ν = 2926, 2253, 1615, 1396, 1265, 1224, 1206, 1142, 908, 734, 650 cm^{-1}; HRMS (EI) Calcd for $C_{11}H_{13}F_3O$ $[M]^+$: 218.0918, Found: 218.0923.

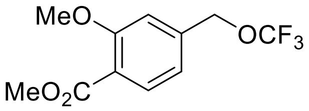

5-13

methyl 2-methoxy-4-((trifluoromethoxy)methyl)benzoate: 47%; Slightly yellow liquid; ^{1}H NMR (400 MHz, $CDCl_3$) δ 7.78 (d, *J* = 8.3 Hz, 1H), 6.93 (s, 1H), 6.92 (d, *J* = 8.3 Hz, 1H), 4.97 (s, 2H), 3.89 (s, 3H), 3.86 (s, 3H); ^{19}F NMR (376 MHz, $CDCl_3$) δ -60.66 (s, 3F); ^{13}C NMR (101 MHz, $CDCl_3$) δ 166.2 (s), 159.4 (s), 139.5 (s), 132.1 (s), 121.7 (q, *J* = 254.6 Hz), 120.4 (s), 118.9 (s), 110.9 (s), 68.2 (q, *J* = 3.5 Hz), 56.1 (s), 52.0 (s); IR (neat) ν = 2954, 2846, 2255, 1730, 1619, 1465, 1436, 1258, 1219, 1146, 1089, 1036, 911, 734 cm^{-1}; HRMS (EI) Calcd for $C_{11}H_{11}F_3O_4$ $[M]^+$: 264.0609, Found: 264.0605.

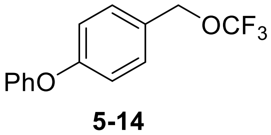

5-14

1-phenoxy-4-((trifluoromethoxy)methyl)benzene: 43%; Yellow liquid; ^{1}H NMR (400 MHz, $CDCl_3$) δ 7.41 – 7.29 (m, 4H), 7.14 (tt, *J* = 7.3, 1.0 Hz, 1H), 7.09 – 6.92 (m, 4H), 4.95 (s, 2H); ^{19}F NMR (376 MHz, $CDCl_3$) δ -60.23 (s, 3F); ^{13}C NMR (101 MHz, $CDCl_3$) δ 158.2 (s), 156.7 (s), 130.0 (s), 129.9 (s), 128.4 (s), 123.8 (s), 121.7 (q, *J* = 255.5 Hz), 119.3 (s), 118.7 (s), 68.8 (q, *J* = 3.4 Hz); IR

(neat) ν = 2253, 1590, 1509, 1489, 1241, 1205, 1148, 908, 733, 693, 650 cm^{-1}; HRMS (EI) Calcd for $C_{14}H_{11}F_3O_2$ $[M]^+$: 268.0711, Found: 268.0719.

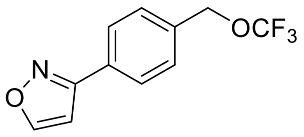

5-15

3-(4-((trifluoromethoxy)methyl)phenyl)isoxazole: 59%; White solid. M.P. 44.6-48.7 °C; ^{1}H NMR (400 MHz, $CDCl_3$) δ 8.46 (d, J = 1.7 Hz, 1H), 7.85 (d, J = 8.2 Hz, 2H), 7.46 (d, J = 8.2 Hz, 2H), 6.66 (d, J = 1.7 Hz, 1H), 5.02 (s, 2H); ^{19}F NMR (376 MHz, $CDCl_3$) δ -60.48 (s, 3F); ^{13}C NMR (101 MHz, $CDCl_3$) δ 161.0 (s), 159.1 (s), 135.7 (s), 129.4 (s), 128.4 (s), 127.3 (s), 121.7 (q, J = 256.0 Hz), 102.5 (s), 68.5 (q, J = 3.4 Hz); IR (neat) ν = 3155, 3127, 1552, 1437, 1262, 1227, 1147, 1125, 889, 834, 768 cm^{-1}; HRMS (EI) Calcd for $C_{11}H_8NF_3O_2$ $[M]^+$: 243.0507, Found: 243.0500.

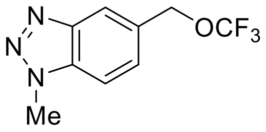

5-16

1-methyl-5-((trifluoromethoxy)methyl)-1H-benzo[d][1,2,3]triazole: 61%; White solid. M.P. 69.5-70.6 °C; ^{1}H NMR (400 MHz, $CDCl_3$) δ 8.01 (s, 1H), 7.63 – 7.41 (m, 2H), 5.11 (s, 2H), 4.28 (s, 3H); ^{19}F NMR (376 MHz, $CDCl_3$) δ -60.30 (s, 3F); ^{13}C NMR (101 MHz, $CDCl_3$) δ 145.9 (s), 133.7 (s), 130.0 (s), 127.6 (s), 121.6 (q, J = 256.0 Hz), 119.8 (s), 109.8 (s), 69.0 (q, J = 3.5 Hz), 34.4 (s); IR (neat) ν = 2952, 1506, 1425, 1279, 1234, 1145, 1016, 876, 806, 670, 657 cm^{-1}; HRMS (EI) Calcd for $C_9H_8N_3F_3O$ $[M]^+$: 231.0619, Found: 231.0620.

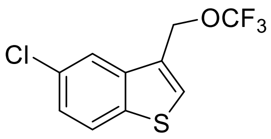

5-17

5-chloro-3-((trifluoromethoxy)methyl)benzo[b]thiophene: 64%; White solid. M.P. 46.1-48.6 °C; ^{1}H NMR (400 MHz, $CDCl_3$) δ 7.79 – 7.73 (m, 2H), 7.56 (s, 1H), 7.35 (dd, J = 8.4, 1.8 Hz, 1H), 5.17 (s, 2H); ^{19}F NMR (376 MHz, $CDCl_3$) δ -60.57 (s, 3F); ^{13}C NMR (101 MHz, $CDCl_3$) δ 138.6 (s), 138.5

(s), 131.2 (s), 129.1 (s), 128.3 (s), 125.5 (s), 123.9 (s), 121.7 (q, *J* = 257.0 Hz), 121.4 (s), 62.9 (q, *J* = 3.7 Hz); IR (neat) ν = 3128, 2964, 1589, 1424, 1249, 1204, 1130, 1076, 834, 789, cm^{-1}; HRMS (EI) Calcd for $C_{10}H_6F_3OSCl$ $[M]^+$: 265.9780, Found: 265.9788.

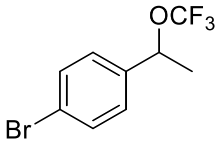

5-18

1-bromo-4-(1-(trifluoromethoxy)ethyl)benzene[11]: 62%; ^1H NMR (400 MHz, $CDCl_3$) δ 7.49 (d, *J* = 8.5 Hz, 2H), 7.21 (d, *J* = 8.5 Hz, 2H), 5.24 (q, *J* = 6.6 Hz, 1H), 1.59 (d, *J* = 6.6 Hz, 3H); ^{19}F NMR (376 MHz, $CDCl_3$) δ -58.21 (s, 3F); ^{13}C NMR (101 MHz, $CDCl_3$) δ 139.5 (s), 131.8 (s), 127.4 (s), 122.4 (s), 121.6 (q, *J* = 255.4 Hz), 76.4 (q, *J* = 2.8 Hz), 23.2 (s).

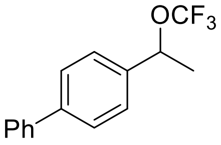

5-19

4-(1-(trifluoromethoxy)ethyl)-1,1'-biphenyl[10]: 47%; ^1H NMR (400 MHz, $CDCl_3$) δ 7.64 – 7.52 (m, 4H), 7.49 – 7.38 (m, 4H), 7.35 (t, *J* = 7.3 Hz, 1H), 5.34 (q, *J* = 6.5 Hz, 1H), 1.66 (d, *J* = 6.5 Hz, 3H); ^{19}F NMR (376 MHz, $CDCl_3$) δ -57.99 (s, 3F); ^{13}C NMR (101 MHz, $CDCl_3$) δ 141.4 (s), 140.5 (s), 139.3 (s), 128.8 (s), 127.5 (s), 127.4 (s), 127.1 (s), 126.2 (s), 121.7 (q, *J* = 254.7 Hz), 76.9 (q, *J* = 2.5 Hz), 23.3 (s).

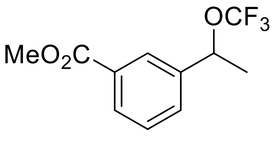

5-20

methyl 3-(1-(trifluoromethoxy)ethyl)benzoate: After flash column chromatography, further purification by HPLC (Prep C18 OBD™ column (19×150mm, 5um), CH_3CN:H_2O (v:v) = 6:4, 254 nm, 20 mL/min, retention time = 7.73 min) was necessary to isolate the pure product. 38% yield; Slightly yellow liquid; ^1H NMR (400 MHz, $CDCl_3$) δ 8.05 – 7.94 (m, 2H), 7.54 (d, *J* = 7.6 Hz, 1H), 7.44 (t, *J* = 8.0 Hz, 1H), 5.32 (q, *J* = 6.5 Hz, 1H), 3.91 (s, 3H), 1.62 (d, *J* = 6.5 Hz, 3H); ^{19}F NMR (376 MHz, $CDCl_3$) δ -58.22 (s, 3F); ^{13}C NMR (101 MHz, $CDCl_3$) δ 166.6 (s), 140.9 (s), 130.6 (s), 130.1 (s), 129.6 (s), 128.8 (s), 126.8 (s), 121.6 (q, *J* = 255.4 Hz), 76.5 (q, *J* = 2.6 Hz), 52.2 (s), 23.3

(s); IR (neat) ν = 2992, 1727, 1559, 1436, 1280, 1223, 1204, 1142, 1111, 1066, 786, 756 cm^{-1}; HRMS (EI) Calcd for $C_{11}H_{11}F_3O_3$ $[M]^+$: 248.0660, Found: 248.0668.

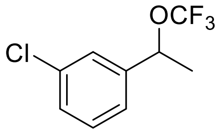

5-21

1-chloro-3-(1-(trifluoromethoxy)ethyl)benzene[11]: 41%; ^{1}H NMR (400 MHz, $CDCl_3$) δ 7.33 (s, 1H), 7.31 – 7.27 (m, 2H), 7.23 – 7.17 (m, 1H), 5.24 (q, *J* = 6.6 Hz, 1H), 1.60 (d, *J* = 6.6 Hz, 3H); ^{19}F NMR (376 MHz, $CDCl_3$) δ -58.30 (s, 3F); ^{13}C NMR (101 MHz, $CDCl_3$) δ 142.4 (s), 134.5 (s), 130.0 (s), 128.5 (s), 125.9 (s), 123.8 (s), 121.5 (q, *J* = 255.7 Hz), 76.2 (q, *J* = 2.6 Hz), 23.3 (s).

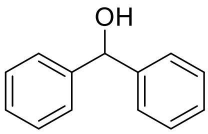

5-22'

diphenylmethanol[12]: 35%; ^{1}H NMR (400 MHz, $CDCl_3$) δ 7.40 – 7.29 (m, 8H), 7.26 (tt, *J* = 6.2, 2.0 Hz, 2H), 5.82 (s, 1H), 2.28 (s, 1H); ^{13}C NMR (101 MHz, $CDCl_3$) δ 143.8 (s), 128.5 (s), 127.6 (s), 126.5 (s), 76.2 (s).

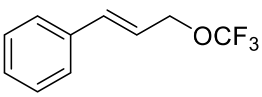

5-23

(E)-(3-(trifluoromethoxy)prop-1-en-1-yl)benzene[9]: 46%; ^{1}H NMR (400 MHz, $CDCl_3$) δ 7.43 – 7.37 (m, 2H), 7.37 – 7.31 (m, 2H), 7.28 (tt, *J* = 5.8, 1.3 Hz, 1H), 6.69 (d, *J* = 15.8 Hz, 1H), 6.25 (dt, *J* = 15.8, 6.4 Hz, 1H), 4.62 (dd, *J* = 6.4, 1.3 Hz, 2H); ^{19}F NMR (376 MHz, $CDCl_3$) δ -60.14 (s, 3F); ^{13}C NMR (101 MHz, $CDCl_3$) δ 135.7 (s), 135.2 (s), 128.7 (s), 128.5 (s), 126.8 (s), 121.7 (q, *J* = 255.3 Hz), 121.4 (s), 68.0 (q, *J* = 3.4 Hz).

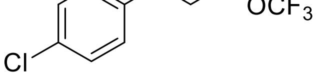

5-24

(*E*)-1-chloro-4-(3-(trifluoromethoxy)prop-1-en-1-yl)benzene: 63%; Colourless liquid; ^{1}H NMR (400 MHz, $CDCl_3$) δ 7.34 – 7.26 (m, 4H), 6.63 (d, *J* = 15.8 Hz, 1H), 6.21 (dt, *J* = 15.8, 6.3 Hz, 1H),

4.60 (dd, J = 6.3, 1.3 Hz, 2H); ^{19}F NMR (376 MHz, $CDCl_3$) δ -60.24 (s, 3F); ^{13}C NMR (101 MHz, $CDCl_3$) δ 134.2 (s), 133.7 (s), 128.9 (s), 127.9 (s), 122.0 (s), 121.7 (q, J = 255.2 Hz), 67.7 (q, J = 3.4 Hz); IR (neat) ν = 2957, 1640, 1492, 1263, 1142, 1094, 1013, 967, 842, 796 cm^{-1}; HRMS (EI) Calcd for $C_{10}H_8F_3OCl$ $[M]^+$: 236.0216, Found: 236.0218.

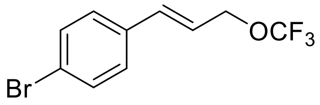

5-25

(*E*)-1-bromo-4-(3-(trifluoromethoxy)prop-1-en-1-yl)benzene[13]: 53%; ^{1}H NMR (400 MHz, $CDCl_3$) δ 7.45 (d, J = 8.4 Hz, 2H), 7.25 (d, J = 8.4 Hz, 2H), 6.62 (d, J = 15.8 Hz, 1H), 6.23 (dt, J = 15.8, 6.3 Hz, 1H), 4.60 (dd, J = 6.3, 1.3 Hz, 2H); ^{19}F NMR (376 MHz, $CDCl_3$) δ -60.25 (s, 3F); ^{13}C NMR (101 MHz, $CDCl_3$) δ 134.6 (s), 133.7 (s), 131.8 (s), 128.2 (s), 122.3 (s), 122.2 (s), 121.7 (q, J = 255.4 Hz), 67.6 (q, J = 3.3 Hz).

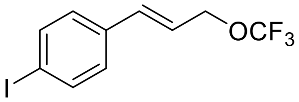

5-26

(*E*)-1-iodo-4-(3-(trifluoromethoxy)prop-1-en-1-yl)benzene: 56%; White solid. M.P. 58.4-58.8 °C; ^{1}H NMR (400 MHz, $CDCl_3$) δ 7.65 (d, J = 8.3 Hz, 2H), 7.11 (d, J = 8.3 Hz, 2H), 6.60 (d, J = 15.9 Hz, 1H), 6.24 (dt, J = 15.9, 6.3 Hz, 1H), 4.59 (dd, J = 6.3, 1.3 Hz, 2H); ^{19}F NMR (376 MHz, $CDCl_3$) δ -60.26 (s, 3F); ^{13}C NMR (126 MHz, $CDCl_3$) δ 137.7 (s), 135.1 (s), 133.8 (s), 128.4 (s), 122.3 (s), 121.6 (q, J = 255.2 Hz), 93.9 (s), 67.6 (q, J = 3.5 Hz); IR (neat) ν = 1660, 1582, 1482, 1331, 1264, 1205, 1146, 1013, 973, 841, 819, 795 cm^{-1}; HRMS (EI) Calcd for $C_{10}H_8F_3OI$ $[M]^+$: 327.9572, Found: 327.9577.

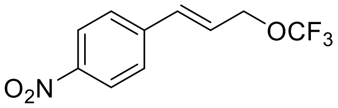

5-27

(*E*)-1-nitro-4-(3-(trifluoromethoxy)prop-1-en-1-yl)benzene: 53%; Yellow liquid; ^{1}H NMR (400 MHz, $CDCl_3$) δ 8.19 (d, J = 8.7 Hz, 2H), 7.52 (d, J = 8.7 Hz, 2H), 6.75 (d, J = 15.9 Hz, 1H), 6.40 (dt, J = 15.9, 5.8 Hz, 1H), 4.66 (dd, J = 5.8, 1.4 Hz, 1H); ^{19}F NMR (376 MHz, $CDCl_3$) δ -60.49 (s, 3F); ^{13}C NMR (101 MHz, $CDCl_3$) δ 147.5 (s), 142.0 (s), 132.0 (s), 127.3 (s), 126.2 (s), 124.0 (s), 121.6 (q, J = 256.0 Hz), 67.0 (q, J = 3.6 Hz); IR (neat) ν = 3081, 1599, 1559, 1520, 1346, 1267,

1216, 1143, 1110, 971, 860 cm^{-1}; HRMS (EI) Calcd for $C_{10}H_8NF_3O_3$ $[M]^+$: 247.0456, Found: 247.0454.

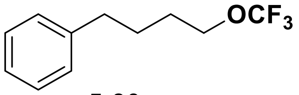

5-30

(4-(trifluoromethoxy)butyl)benzene[11]: 51%; ^{1}H NMR (400 MHz, $CDCl_3$) δ 7.28 (t, *J* = 7.4 Hz, 2H), 7.22 – 7.12 (m, 3H), 3.95 (t, *J* = 5.9 Hz, 2H), 2.64 (t, *J* = 7.0 Hz, 2H), 1.80 – 1.62 (m, 4H); ^{19}F NMR (376 MHz, $CDCl_3$) δ -60.74 (s, 3F); ^{13}C NMR (101 MHz, $CDCl_3$) δ 141.7 (s), 128.4 (s), 128.3 (s), 125.9 (s), 121.6 (q, *J* = 254.0 Hz), 67.2 (q, *J* = 3.0 Hz), 35.2 (s), 28.2 (s), 27.2 (s).

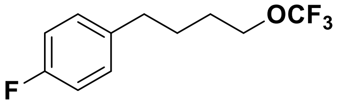

5-31

1-fluoro-4-(4-(trifluoromethoxy)butyl)benzene: 51%; Slightly yellow liquid; ^{1}H NMR (400 MHz, $CDCl_3$) δ 7.16 – 7.05 (m, 2H), 6.96 (t, *J* = 8.7 Hz, 2H), 3.95 (t, *J* = 5.9 Hz, 2H), 2.61 (t, *J* = 6.5 Hz, 2H), 1.75 – 1.65 (m, 4H); ^{19}F NMR (376 MHz, $CDCl_3$) δ -60.78 (s, 3F), -117.58 – -117.82 (m, 1F); ^{13}C NMR (126 MHz, $CDCl_3$) δ 161.3 (d, *J* = 243.5 Hz), 137.2 (d, *J* = 3.1 Hz), 129.6 (d, *J* = 7.8 Hz), 121.6 (q, *J* = 253.8 Hz), 115.0 (d, *J* = 21.1 Hz), 67.1 (q, *J* = 3.2 Hz), 34.4 (s), 28.1 (s), 27.3 (s); IR (neat) ν = 2946, 1509, 1273, 1223, 1144, 908, 734 cm^{-1}; HRMS (EI) Calcd for $C_{11}H_{12}OF_4$ $[M]^+$: 236.0824, Found: 236.0829.

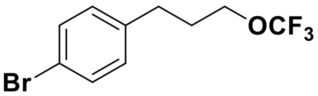

5-32

1-bromo-4-(3-(trifluoromethoxy)propyl)benzene[11]: 61%; ^{1}H NMR (400 MHz, $CDCl_3$) δ 7.40 (d, *J* = 8.3 Hz, 2H), 7.05 (d, *J* = 8.3 Hz, 2H), 3.93 (t, *J* = 6.2 Hz, 2H), 2.68 (t, *J* = 7.3 Hz, 2H), 2.03 – 1.85 (m, 2H); ^{19}F NMR (376 MHz, $CDCl_3$) δ -60.73 (s, 3F); ^{13}C NMR (126 MHz, $CDCl_3$) δ 139.4 (s), 131.5 (s), 130.1 (s), 121.6 (q, *J* = 254.2 Hz), 119.9 (s), 66.1 (q, *J* = 3.2 Hz), 30.9 (s), 30.1 (s).

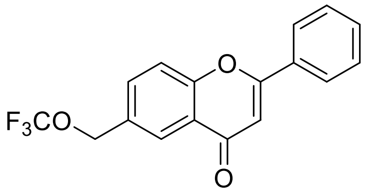

5-34

2-phenyl-6-((trifluoromethoxy)methyl)-4H-chromen-4-one: A 0.2 mmol scale was performed. The product isolated by flash column chromatography was not very pure, and therefore crystalization from petroleum ether / ethyl acetate was necessary to get the pure product. 66% yield ; White solid. M.P. 126.2-129.9 °C; ^{1}H NMR (400 MHz, $CDCl_3$) δ 8.23 (d, *J* = 2.1 Hz, 1H), 7.94 (dd, *J* = 7.6, 1.8 Hz, 2H), 7.74 (dd, *J* = 8.6, 2.1 Hz, 1H), 7.63 (d, *J* = 8.6 Hz, 1H), 7.60 – 7.47 (m, 3H), 6.87 (s, 1H), 5.09 (s, 2H); ^{19}F NMR (376 MHz, $CDCl_3$) δ -60.46 (s, 3F); ^{13}C NMR (101 MHz, $CDCl_3$) δ 178.0 (s), 163.9 (s), 156.3 (s), 133.3 (s), 131.9 (s), 131.5 (s), 131.3 (s), 129.2 (s), 126.4 (s), 125.3 (s), 123.8 (s), 121.6 (q, *J* = 256.1 Hz), 118.9 (s), 107.6 (s), 68.1 (q, *J* = 3.3 Hz); IR (neat) ν = 3066, 1651, 1621, 1497, 1275, 1211, 1164, 1138, 1041, 818, cm^{-1}; HRMS (EI) Calcd for $C_{17}H_{11}F_3O_3$ $[M]^+$: 320.0660, Found: 320.0652.

The synthesis of the CF_3O-containing Trioxsalen derivaive

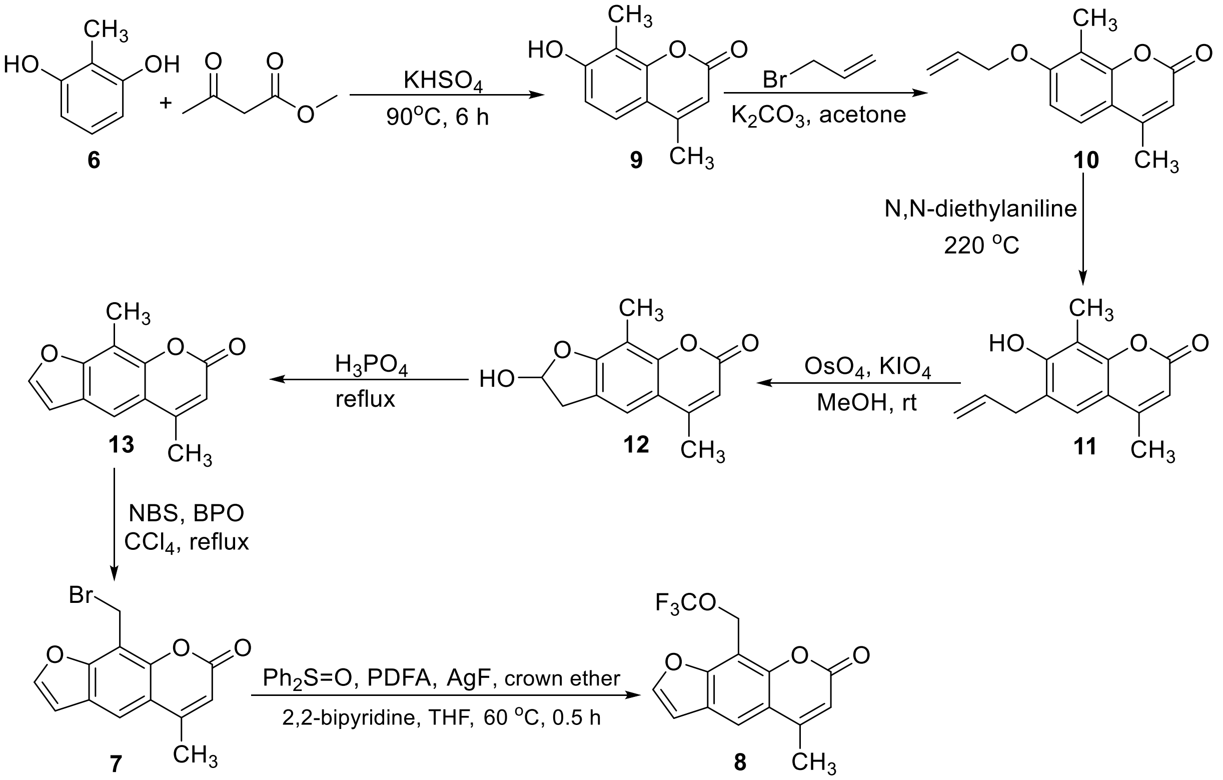

Supplementary Figure 1. The synthesis of the CF_3O-containing Trioxsalen derivaive

Compound **9** was prepared according to the reported procedure.[14]

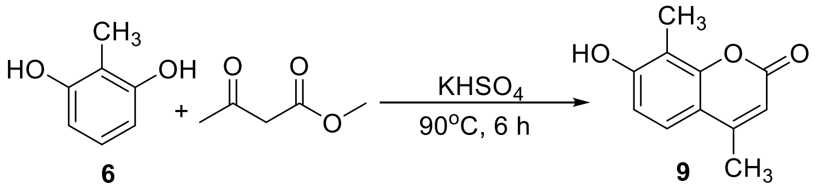

A mixture of $KHSO_4$ (2.72 g, 20.0 mmol), resorcinol (12.4 g, 100.0 mmol) and methyl acetoacetate (12 g, 100.0 mmol) was stirred vigorously at 90 °C for 6 h. The liquid mixture solidified after the reaction mixture was cooled to room temperature. Ethanol (100 ml) was added and the resulting mixture was warmed to 50 °C. This ethanol solution was then put into a 500 ml beaker containing 300 mL of water. The solid was collected by filtration and was then recrystallized from EtOH to give pure 7-hydroxy-4-methylcoumarin (11.6 g, 61 %).

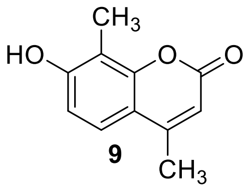

7-hydroxy-4,8-dimethyl-2*H*-chromen-2-one[14]: 61%; ^{1}H NMR (400 MHz, DMSO) δ 10.37 (s, 1H), 7.41 (d, *J* = 8.7 Hz, 1H), 6.82 (d, *J* = 8.7 Hz, 1H), 6.08 (s, 1H), 2.32 (s, 3H), 2.11 (s, 3H).

Compound **10** was prepared according to the reported procedure.[15]

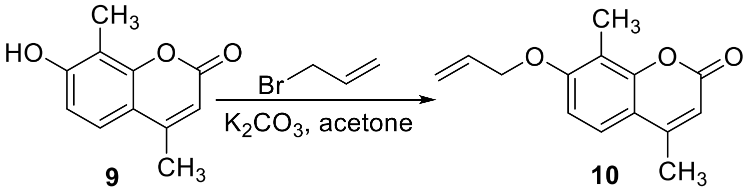

A mixture of **9** (2.3 g, 12.0 mmol), allyl bromide (8.7 g, 72 mmol), anhydrous potassium carbonate (8.3 g, 60 mmol) and acetone (50 mL) was refluxed for 10 h. After filtration, the filtrate was concentrated under vacuum to give compound **10** (2.6 g, 96%).

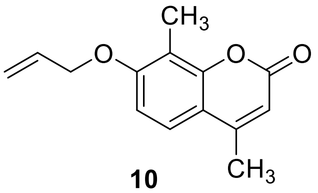

7-(allyloxy)-4,8-dimethyl-2*H*-chromen-2-one[15]: 96%; ^{1}H NMR (400 MHz, $CDCl_3$) δ 7.37 (d, *J* = 8.8 Hz, 1H), 6.80 (d, *J* = 8.8 Hz, 1H), 6.11 (s, 1H), 6.09 – 5.98 (m, 1H), 5.42 (dd, *J* = 17.3, 1.2 Hz, 1H),

5.30 (dd, *J* = 10.6, 1.2 Hz, 1H), 4.62 (d, *J* = 5.0 Hz, 2H), 2.37 (s, 3H), 2.31 (s, 3H).

Compound **11** was prepared according to the reported procedure.[15]

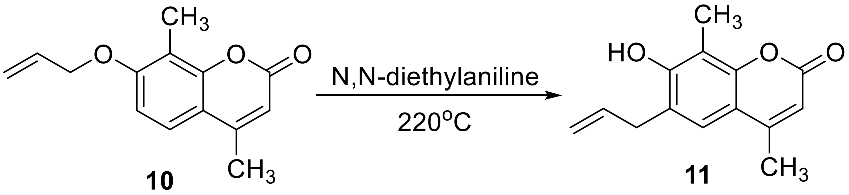

A solution of **10** (2.6 g, 11.5 mmol) in N,N-diethylaniline (20 mL) was heated under reflux for two hours, allowed to cool, and diluted with pet ether. Filtration gave 2.4 g (89%).

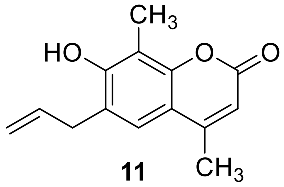

6-allyl-7-hydroxy-4,8-dimethyl-2*H*-chromen-2-one[15]: 91%; ^{1}H NMR (400 MHz, $CDCl_3$) δ 7.18 (s, 1H), 6.12 (s, 1H), 6.08 – 5.92 (m, 1H), 5.58 (s, 1H), 5.23 (q, *J* = 1.7 Hz, 1H), 5.19 (dd, *J* = 10.6, 1.7 Hz, 1H), 3.46 (d, *J* = 6.3 Hz, 2H), 2.37 (s, 3H), 2.31 (s, 3H).

Compound **12** and **13** was prepared according to the reported procedure.[15]

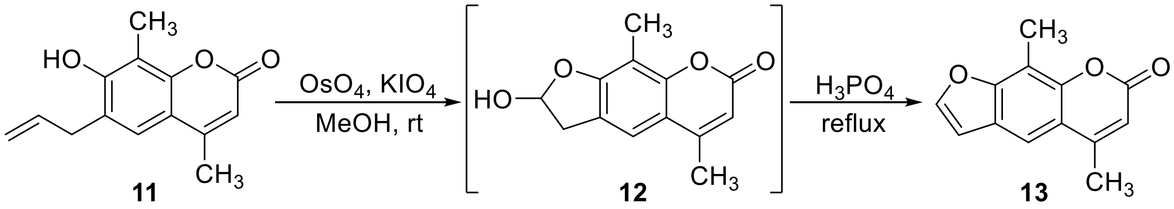

A solution of osminu tetroxide (23 mg, 0.09 mmol) in water (10 mL) was added to a vigorously stirred mixture of potassium periodate (5.1 g, 24 mmol) and compound **11** (2.4 g, 10.4 mmol) in methanol (60 mL) for 24 h. The mixture was diluted with dichloromethane (100 mL), filtered, and the residue was washed with two portions(50 mL) of dichloromethane. The combined filtrate was washed with saturated brine (100 ml × 2). The combined brine solutions were extracted with dichloromethane (200 mL). All dichloromethane layers were combined, dried with sodium sulfate, and concentrated in vacuo to obtain crude product, **12** (2.3 g, 96%), which was used for the next step without further purification.

The mixture of compound **12** (2.3 g, 10 mmol) and 85% phosphoric acid (60 mL) was stirred at 130 °C for 30 min. The mixture was poured into water (300 mL). The product was extracted with CH_2Cl_2 (150 mL × 3). The combined CH_2Cl_2 solution was dried with Na_2SO_4, and then concentrated under vacuum. The residue was subjected to flash column chromatography to afford compound **13** as a white solid (0.27 g, 13%).

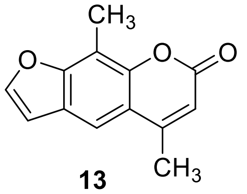

13

5,9-dimethyl-7*H*-furo[3,2-*g*]chromen-7-one[15]: 13%; ^{1}H NMR (400 MHz, $CDCl_3$) δ 7.68 (d, *J* = 2.2 Hz, 1H), 7.65 (s, 1H), 6.81 (d, *J* = 2.2 Hz, 1H), 6.25 (s, 1H), 2.60 (s, 3H), 2.48 (s, 3H).

Compound **7** was prepared according to the reported procedure.[15]

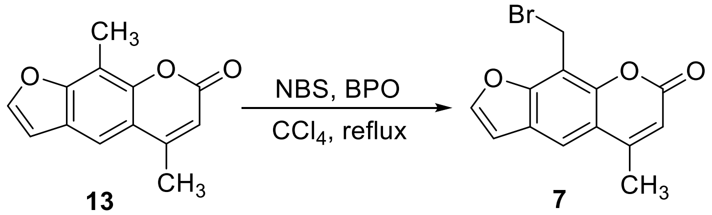

A mixture of *N*-bromosuccinimide (0.23 g, 1.30 mmol), carbon tetrachloride (20 mL), compound **13** (0.27 g, 1.28 mmol), and benzoyl peroxide (0.032 g, 0.13 mmol) was refluxed for 4 h. The mixture was cooled to room temperature, and the solution was concentrated to remove the solvent. The residue was subjected to flash column chromatography to afford **7** as a slightly yellow solid (0.161 g, 43%).

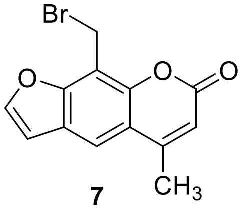

7

9-(bromomethyl)-5-methyl-7*H*-furo[3,2-*g*]chromen-7-one[15]: 43%; ^{1}H NMR (400 MHz, $CDCl_3$) δ 7.79 (s, 1H), 7.74 (d, *J* = 2.2 Hz, 1H), 6.86 (d, *J* = 2.2 Hz, 1H), 6.28 (d, *J* = 1.0 Hz, 1H), 4.98 (s, 2H), 2.49 (d, *J* = 1.0 Hz, 3H); ^{13}C NMR (101 MHz, $CDCl_3$) δ 160.3 (s), 154.4 (s), 152.7 (s), 148.9

(s), 147.0 (s), 124.2 (s), 116.9 (s), 116.8 (s), 113.5 (s), 110.0 (s), 106.9 (s), 19.4 (s), 19.2 (s).

The synthesis of the CF_3O-containing Trioxsalen derivative (**8**).

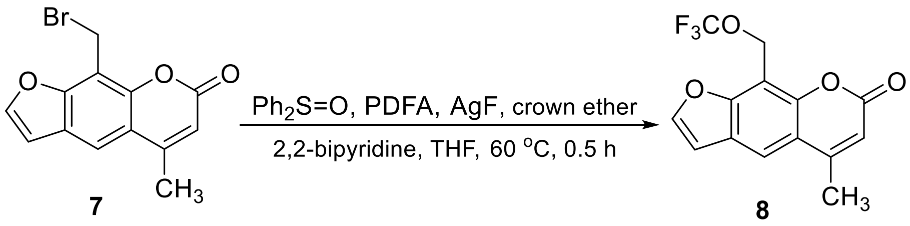

Into a 10 mL sealed tube were added benzyl bromide **7** (0.2 mmol, 58.6 mg, 1.0 equiv.), $Ph_3P^+CF_2CO_2^-$ (0.5 mmol, 178.0 mg, 2.5 equiv), Ph_2S=O (0.5 mmol, 101.2 mg, 2.5 equiv), AgF (0.4 mmol, 51.0 mg, 2.0 equiv), 2,2'-bipyridine (0.3 mmol, 47.0 mg, 1.5 equiv), 2,3,11,12-dibenzo-18-crown-6 (0.1 mmol, 36.0 mg, 0.5 equiv) and THF (1.5 mL) under a N_2 atmosphere. The tube was sealed and the reaction mixture was stirred at 60 °C for 30 min. After the mixture was cooled to room temperature, the pure product was isolated by flash column chromatography to afford **8** (32 mg, 54 %).

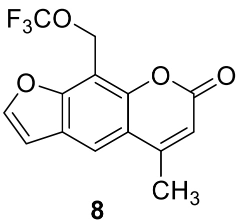

5-methyl-9-((trifluoromethoxy)methyl)-7*H*-furo[3,2-*g*]chromen-7-one: 54%; White solid. M.P. 144.6-152.5 °C; ^1H NMR (400 MHz, $CDCl_3$) δ 7.86 (s, 1H), 7.73 (d, J = 2.2 Hz, 1H), 6.86 (d, J = 2.2 Hz, 1H), 6.28 (q, J = 1.0 Hz, 1H), 5.53 (s, 2H), 2.49 (d, J = 1.0 Hz, 3H); ^{19}F NMR (376 MHz, $CDCl_3$) δ -60.69 (s, 3F); ^{13}C NMR (101 MHz, $CDCl_3$) δ 160.1 (s), 155.2 (s), 152.6 (s), 149.8 (s), 147.1 (s), 124.2 (s), 121.7 (q, J = 256.5 Hz), 118.0 (s), 116.6 (s), 113.6 (s), 106.8 (s), 105.6 (s), 57.7 (q, J = 3.9 Hz), 19.2 (s). IR (neat) ν = 3415, 2358, 1721, 1349, 1326, 1290, 1213, 900, 849, 758, 608 cm^{-1}; HRMS (EI) Calcd for $C_{14}H_9F_3O_4$ $[M]^+$: 298.0453, Found: 298.0450.

The procedure for the synthesis of ^{18}O-diphenyl sulfoxide[16]

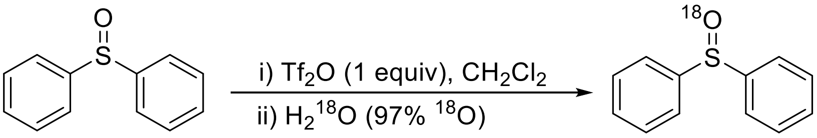

Tf_2O (8.36 μL, 49.6 mmol) was added to a solution of diphenyl sulfoxide (10.0 g, 49.6 mmol) in CH_2Cl_2 (190 mL) at −70 ºC in a dry ice / ethanol bath. The reaction mixture was stirred at −70 ºC for 1 h, and then $H_2{}^{18}O$ (^{18}O content: 97%, 1.08 mL, 59.6 mmol) was added. The reaction mixture was instantly removed from the dry ice / ethanol bath and then stirred at room temperature for 3 h. The mixture was diluted with CH_2Cl_2 (100 mL), and was then quenched with trimethylamine (15 mL). The resulting mixture was washed with HCl (1 M, 3 x 200 mL), aq. $NaHCO_3$ solution (3 x 200 mL), saturated aq. NaCl solution (3 x 200 mL), and dried with $MgSO_4$. The solution was concentrated to afford a crude solid. The crude solid was purified by flash column chromatography (hexane-EtOAc, v/v = 2:1) to afford $Ph_2S{=}^{18}O$ (9.12 g, 90% yield, ^{18}O content: 71%) as a colourless solid. The ^{18}O content was determined by MS-EI spectroscopy.

The above procedure was repeated once more to get $Ph_2S{=}^{18}O$ in a high ^{18}O content. The product obtained above (9.1 g, 45.2 mmol) was re-dissolved in CH_2Cl_2 (180 mL) at −70 ºC in a dry ice / ethanol bath, and Tf_2O (7.62 mL, 45.2 mmol) was added to the solution. The reaction mixture was stirred at −70 ºC for 10 min, and then $H_2{}^{18}O$ (^{18}O content: 97%, 0.984 mL, 54.4 mmol) was added. The reaction mixture was instantly removed from the dry ice / ethanol bath and then stirred at room temperature for 3 h. The mixture was diluted with CH_2Cl_2 (100 mL), and was then quenched with trimethylamine (15 mL). The resulting mixture was washed with HCl (1 M, 3 x 200 mL), aq. $NaHCO_3$ solution (3 x 200 mL), saturated aq. NaCl solution (3 x 200 mL), and dried with $MgSO_4$. The solution was concentrated to afford a crude solid. The crude solid was purified by flash column chromatography (hexane-EtOAc, v/v = 2:1) to afford $Ph_2S{=}^{18}O$ (7.2 g, 78% yield, ^{18}O content: 89%) as a colourless solid. The ^{18}O content was determined by MS-EI spectroscopy.

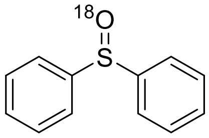

(sulfinyl-^{18}O)dibenzene: 83%; ^{1}H NMR (400 MHz, $CDCl_3$) δ 7.67 – 7.57 (m, 4H), 7.47 – 7.34 (m, 6H); ^{13}C NMR (101 MHz, $CDCl_3$) δ 145.6 (s), 131.1 (s), 129.3 (s), 124.8 (s); GC-MS (EI): 204.0 (M^+).

Typical procedure for ^{18}O-trifluoromethoxylation of benzyl bromides

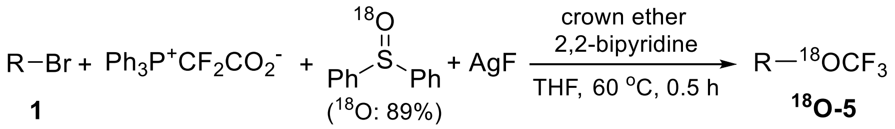

Into a 10 mL sealed tube were added benzyl bromide **1-1** (0.2 mmol, 49.4 mg, 1.0 equiv.), $Ph_3P^+CF_2CO_2^-$ (0.5 mmol, 178.0 mg, 2.5 equiv), $Ph_2S{=}^{18}O$ (0.5 mmol, 102.1 mg, 2.5 equiv), AgF (0.4 mmol, 51.0 mg, 2.0 equiv), 2,2'-bipyridine (0.3 mmol, 47.0 mg, 1.5 equiv), 2,3,11,12-dibenzo-18-crown-6 (0.1 mmol, 36.0 mg, 0.5 equiv) and THF (1.5 mL) under a N_2 atmosphere. The tube was sealed and the reaction mixture was stirred at 60 °C for 30 min., and the mixture was cooled to room temperature. The yields of the desired products were determined by ^{19}F NMR spectroscopy, and the ^{18}O contents were determined by GC-MS (EI) spectroscopy.

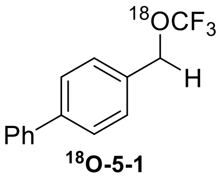

^{18}O-5-1

^{18}O-4-((trifluoromethoxy)methyl)-1,1'-biphenyl: 67%; ^{1}H NMR (400 MHz, $CDCl_3$) δ 7.69 – 7.58 (m, 4H), 7.52 – 7.44 (m, 4H), 7.40 (t, J = 7.3 Hz, 1H), 5.06 (s, 2H); ^{19}F NMR (376 MHz, $CDCl_3$) -60.29 (s, 3F); EI: 254.1 (M^+); ^{18}O content: 89% determined by GC-MS spectroscopy.

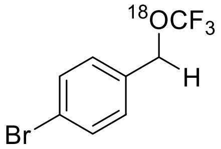

^{18}O-5-2

^{18}O-1-bromo-4-((trifluoromethoxy)methyl)benzene: 69%; ^{1}H NMR (400 MHz, $CDCl_3$) δ 7.53 (d, J = 8.3 Hz, 2H), 7.24 (d, J = 8.3 Hz, 2H), 4.93 (s, 2H); ^{19}F NMR (376 MHz, $CDCl_3$) δ -60.53 (s, 3F); EI: 255.9 (M^+); ^{18}O content: 91% determined by GC-MS spectroscopy.

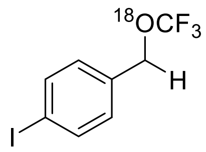

^{18}O-5-3

^{18}O-1-iodo-4-((trifluoromethoxy)methyl)benzene: 79%; ^{1}H NMR (400 MHz, $CDCl_3$) δ 7.72 (d, *J* = 8.3 Hz, 2H), 7.09 (d, *J* = 8.3 Hz, 2H), 4.91 (s, 2H); ^{19}F NMR (376 MHz, $CDCl_3$) δ -60.53 (s, 3F). EI: 303.9 (M^+); ^{18}O content: 89% determined by GC-MS spectroscopy.

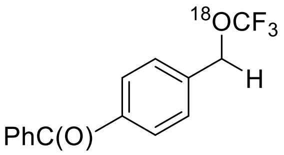

^{18}O-5-4

^{18}O-phenyl(4-((trifluoromethoxy)methyl)phenyl)methanone: 55%;^{1}H NMR (400 MHz, $CDCl_3$) δ 7.89 – 7.80 (m, 4H), 7.63 (tt, *J* = 6.8, 1.2 Hz, 1H), 7.55 – 7.48 (m, 4H), 5.10 (s, 2H); ^{19}F NMR (376 MHz, $CDCl_3$) δ -60.57 (s, 3F). EI: 282.0 (M^+); ^{18}O content: 89% determined by GC-MS spectroscopy.

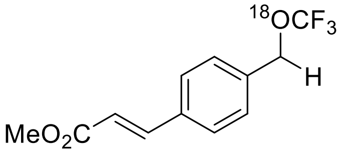

^{18}O-5-5

^{18}O-methyl (*E*)-3-(4-((trifluoromethoxy)methyl)phenyl)acrylate: 63%; ^{1}H NMR (400 MHz, $CDCl_3$) δ 7.71 (d, *J* = 16.0 Hz, 1H), 7.57 (d, *J* = 8.2 Hz, 2H), 7.41 (d, *J* = 8.2 Hz, 2H), 6.49 (d, *J* = 16.0 Hz, 1H), 5.02 (s, 2H), 3.84 (s, 3H); ^{19}F NMR (376 MHz, $CDCl_3$) δ -60.48 (s, 3F); EI: 262.0 (M^+); ^{18}O content: 89% determined by GC-MS spectroscopy.

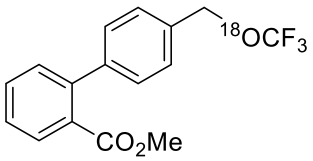

^{18}O-5-6

^{18}O-methyl-4'-((trifluoromethoxy)methyl)-[1,1'-biphenyl]-2-carboxylate: 64%; ^{1}H NMR (400 MHz, $CDCl_3$) δ 7.89 (dd, *J* = 7.7, 1.2 Hz, 1H), 7.57 (td, *J* = 7.6, 1.4 Hz, 1H), 7.49 – 7.41 (m, 3H), 7.41 – 7.33 (m, 3H), 5.06 (s, 2H), 3.68 (s, 3H); ^{19}F NMR (376 MHz, $CDCl_3$) δ -60.30 (s, 3F); EI: 312.0

(M+); ^{18}O content: 89% determined by GC-MS spectroscopy.

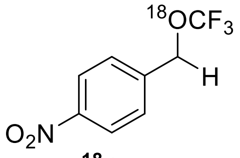

18O-5-7

^{18}O-1-nitro-4-((trifluoromethoxy)methyl)benzene: 60%; ^{1}H NMR (400 MHz, $CDCl_3$) δ 8.29 (d, *J* = 8.7 Hz, 2H), 7.57 (d, *J* = 8.7 Hz, 2H), 5.12 (s, 2H); ^{19}F NMR (376 MHz, $CDCl_3$) δ -60.81 (s, 3F); EI: 223.0 (M^+); ^{18}O content: 90% determined by GC-MS spectroscopy.

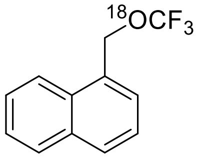

18O-5-8

^{18}O-1-((trifluoromethoxy)methyl)naphthalene: 39%; ^{1}H NMR (400 MHz, $CDCl_3$) δ 8.03 (d, *J* = 8.4 Hz, 1H), 7.91 (d, *J* = 7.9 Hz, 2H), 7.65 – 7.52 (m, 3H), 7.52 – 7.43 (m, 1H), 5.45 (s, 2H). ^{19}F NMR (376 MHz, $CDCl_3$) δ -60.38 (s, 3F); EI: 228.0 (M^+); ^{18}O content: 88% determined by GC-MS spectroscopy.

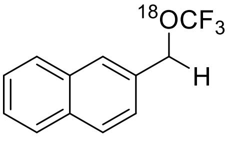

18O-5-9

^{18}O-2-((trifluoromethoxy)methyl)naphthalene: 61%; ^{1}H NMR (400 MHz, $CDCl_3$) δ 7.91 – 7.80 (m, 4H), 7.55 – 7.48 (m, 2H), 7.46 (dd, *J* = 8.4, 1.7 Hz, 1H), 5.14 (s, 2H); ^{19}F NMR (376 MHz, $CDCl_3$) δ -60.25 (s, 3F); EI: 228.0 (M^+); ^{18}O content: 89% determined by GC-MS spectroscopy.

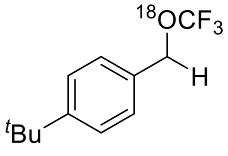

18O-5-10

^{18}O-1-(tert-butyl)-4-((trifluoromethoxy)methyl)benzene: 62%; ^{1}H NMR (400 MHz, $CDCl_3$) δ 7.42

(d, J = 8.3 Hz, 2H), 7.31 (d, J = 8.2 Hz, 2H), 4.95 (s, 2H), 1.33 (s, 9H); ^{19}F NMR (376 MHz, $CDCl_3$) δ -60.33 (s, 3F); EI: 234.0 (M^+); ^{18}O content: 89% determined by GC-MS spectroscopy.

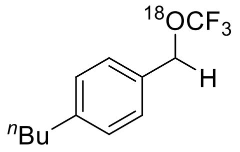

^{18}O-5-11

^{18}O-1-butyl-4-((trifluoromethoxy)methyl)benzene: 58%; ^{1}H NMR (400 MHz, $CDCl_3$) δ 7.28 (d, J = 8.0 Hz, 2H), 7.20 (d, J = 8.0 Hz, 2H), 4.94 (s, 2H), 2.62 (t, J = 7.7 Hz, 2H), 1.65 – 1.57 (m, 2H), 1.41 – 1.29 (m, 2H), 0.92 (t, J = 7.3 Hz, 3H). ^{19}F NMR (376 MHz, $CDCl_3$) δ -60.30 (s, 3F); EI: 234.1 (M^+); ^{18}O content: 87% determined by GC-MS spectroscopy.

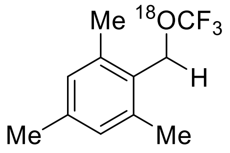

^{18}O-5-12

^{18}O-1,3,5-trimethyl-2-((trifluoromethoxy)methyl)benzene: 46%; ^{1}H NMR (400 MHz, $CDCl_3$) δ 6.88 (s, 2H), 5.04 (s, 2H), 2.35 (s, 6H), 2.26 (s, 3H); ^{19}F NMR (376 MHz, $CDCl_3$) δ -60.74 (s, 3F); EI: 220.0 (M^+); ^{18}O content: 89% determined by GC-MS spectroscopy.

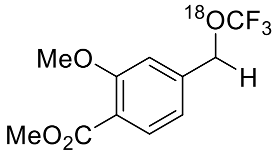

^{18}O-5-13

^{18}O-methyl-2-methoxy-4-((trifluoromethoxy)methyl)benzoate: 43%; ^{1}H NMR (400 MHz, $CDCl_3$) δ 7.81 (d, J = 8.3 Hz, 1H), 6.97 (s, 1H), 6.96 (d, J = 8.3 Hz, 1H), 5.00 (s, 2H), 3.93 (s, 3H), 3.90 (s, 3H); ^{19}F NMR (376 MHz, $CDCl_3$) δ -60.65 (s, 3F); EI: 266.0 (M^+); ^{18}O content: 87% determined by GC-MS spectroscopy.

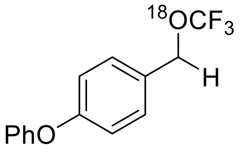

^{18}O-5-14

^{18}O-1-phenoxy-4-((trifluoromethoxy)methyl)benzene: 54%; ^{1}H NMR (400 MHz, $CDCl_3$) δ 7.39 –

7.29 (m, 4H), 7.14 (tt, *J* = 7.3, 1.0 Hz, 1H), 7.06 – 6.97 (m, 4H), 4.95 (s, 2H); ^{19}F NMR (376 MHz, $CDCl_3$) δ -60.27 (s, 3F); EI: 270.0 (M^+); ^{18}O content: 89% determined by GC-MS spectroscopy.

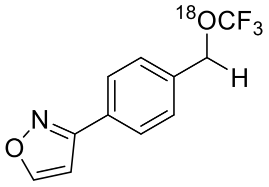

18O-5-15

^{18}O-3-(4-((trifluoromethoxy)methyl)phenyl)isoxazole: 53%; ^{1}H NMR (400 MHz, $CDCl_3$) δ 8.48 (d, *J* = 1.7 Hz, 1H), 7.87 (d, *J* = 8.2 Hz, 2H), 7.47 (d, *J* = 8.1 Hz, 2H), 6.68 (d, *J* = 1.6 Hz, 1H), 5.04 (s, 2H); ^{19}F NMR (376 MHz, $CDCl_3$) δ -60.50 (s, 3F); EI: 245.0 (M^+); ^{18}O content: 86% determined by GC-MS spectroscopy.

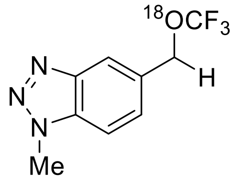

18O-5-16

^{18}O-1-methyl-5-((trifluoromethoxy)methyl)-1H-benzo[d][1,2,3]triazole: 56%; ^{1}H NMR (400 MHz, $CDCl_3$) δ 8.06 (s, 1H), 7.61 – 7.48 (m, 2H), 5.15 (s, 2H), 4.33 (s, 3H); ^{19}F NMR (376 MHz, $CDCl_3$) δ -60.35 (s, 3F); EI: 233.0 (M^+); ^{18}O content: 89% determined by GC-MS spectroscopy.

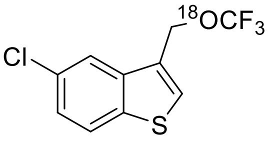

18O-5-17

^{18}O-5-chloro-3-((trifluoromethoxy)methyl)benzo[b]thiophene: 44%; ^{1}H NMR (400 MHz, ^{1}H NMR (400 MHz, $CDCl_3$) δ 7.83 – 7.73 (m, 2H), 7.58 (s, 1H), 7.37 (dd, *J* = 8.5, 1.9 Hz, 1H), 5.19 (s, 2H); ^{19}F NMR (376 MHz, $CDCl_3$) δ -60.49 (s, 3F); EI: 267.9 (M^+); ^{18}O content: 89% determined by GC-MS spectroscopy.

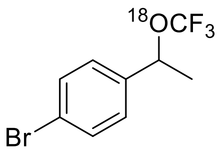

^{18}O-5-18

^{18}O-1-bromo-4-(1-(trifluoromethoxy)ethyl)benzene: 63%; ^{1}H NMR (400 MHz, $CDCl_3$) δ 7.50 (d, *J* = 8.4 Hz, 2H), 7.22 (d, *J* = 8.5 Hz, 2H), 5.25 (q, *J* = 6.6 Hz, 1H), 1.60 (d, *J* = 6.6 Hz, 3H); ^{19}F NMR (376 MHz, $CDCl_3$) δ -58.24 (s, 3F); EI: 270.0 (M^+); ^{18}O content: 91% determined by GC-MS spectroscopy.

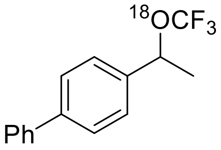

^{18}O-5-19

^{18}O-4-(1-(trifluoromethoxy)ethyl)-1,1'-biphenyl: 54%; ^{1}H NMR (400 MHz, $CDCl_3$) δ 7.65 – 7.54 (m, 4H), 7.48 – 7.38 (m, 4H), 7.36 (t, *J* = 7.3 Hz, 1H), 5.35 (q, *J* = 6.6 Hz, 1H), 1.67 (d, *J* = 6.6 Hz, 3H); ^{19}F NMR (376 MHz, $CDCl_3$) δ -57.96 (s, 3F); EI: 268.1 (M^+); ^{18}O content: 90% determined by GC-MS spectroscopy.

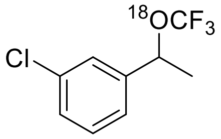

^{18}O-5-21

^{18}O-1-chloro-3-(1-(trifluoromethoxy)ethyl)benzene: 44%; ^{1}H NMR (400 MHz, $CDCl_3$) δ 7.34 (s, 1H), 7.33 – 7.28 (m, 2H), 7.24 – 7.19 (m, 1H), 5.25 (q, *J* = 6.6 Hz, 1H), 1.62 (d, *J* = 6.6 Hz, 3H); ^{19}F NMR (376 MHz, $CDCl_3$) δ -58.35 (s, 3F); EI: 226.0 (M^+); ^{18}O content: 89% determined by GC-MS spectroscopy.

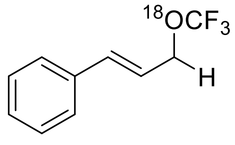

^{18}O-5-23

^{18}O- (E)-(3-(trifluoromethoxy)prop-1-en-1-yl)benzene: 43%; ^{1}H NMR (400 MHz, $CDCl_3$) δ 7.42 –

7.36 (m, 2H), 7.36 – 7.30 (m, 2H), 7.31 – 7.26 (m, 1H), 6.68 (d, *J* = 15.8 Hz, 1H), 6.24 (dt, *J* = 15.8, 6.4 Hz, 1H), 4.61 (d, *J* = 6.4 Hz, 2H); ^{19}F NMR (376 MHz, $CDCl_3$) δ -60.16 (s, 3F); EI: 204.0 (M^+); ^{18}O content: 90% determined by GC-MS spectroscopy.

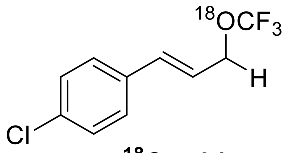

^{18}O-5-24

^{18}O-(*E*)-1-chloro-4-(3-(trifluoromethoxy)prop-1-en-1-yl)benzene: 55%;^{1}H NMR (400 MHz, $CDCl_3$) δ 7.36 – 7.26 (m, 4H), 6.63 (d, *J* = 15.9 Hz, 1H), 6.21 (dt, *J* = 15.8, 6.3 Hz, 1H), 4.60 (d, *J* = 6.3 Hz, 2H); ^{19}F NMR (376 MHz, $CDCl_3$) δ -60.20 (s, 3F); EI: 238.0 (M^+); ^{18}O content: 89% determined by GC-MS spectroscopy.

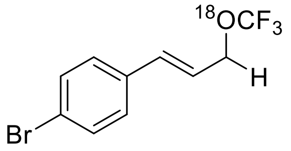

^{18}O-5-25

^{18}O-(*E*)-1-bromo-4-(3-(trifluoromethoxy)prop-1-en-1-yl)benzene: 62%; ^{1}H NMR (400 MHz, $CDCl_3$) δ 7.46 (d, *J* = 8.5 Hz, 2H), 7.25 (d, *J* = 8.5 Hz, 2H), 6.62 (d, *J* = 15.9 Hz, 1H), 6.24 (dt, *J* = 15.9, 6.3 Hz, 1H), 4.60 (dd, *J* = 6.3, 1.2 Hz, 2H); ^{19}F NMR (376 MHz, $CDCl_3$) δ -60.23 (s, 3F); EI: 282.0 (M^+); ^{18}O content: 90% determined by GC-MS spectroscopy.

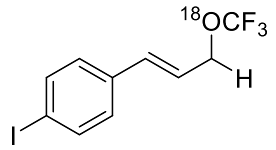

^{18}O-5-26

^{18}O-(*E*)-1-iodo-4-(3-(trifluoromethoxy)prop-1-en-1-yl)benzene: 55%; ^{1}H NMR (400 MHz, $CDCl_3$) δ 7.67 (d, *J* = 8.3 Hz, 2H), 7.14 (d, *J* = 8.3 Hz, 2H), 6.62 (d, *J* = 16.0 Hz, 1H), 6.26 (dt, *J* = 16.0, 6.3 Hz, 1H), 4.61 (dd, *J* = 6.3, 1.1 Hz, 2H); ^{19}F NMR (376 MHz, $CDCl_3$) δ -60.21 (s, 3F); EI: 330.0 (M^+); ^{18}O content: 88% determined by GC-MS spectroscopy.

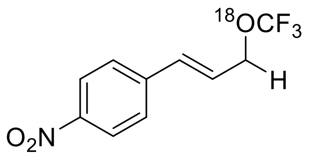

^{18}O-5-27

^{18}O-(*E*)-1-nitro-4-(3-(trifluoromethoxy)prop-1-en-1-yl)benzene: 57%; ^{1}H NMR (400 MHz, $CDCl_3$) δ 8.18 (d, *J* = 8.8 Hz, 2H), 7.52 (d, *J* = 8.8 Hz, 2H), 6.75 (d, *J* = 16.0 Hz, 1H), 6.40 (dt, *J* = 16.0, 5.8 Hz, 1H), 4.66 (dd, *J* = 5.8, 1.4 Hz, 1H); ^{19}F NMR (376 MHz, $CDCl_3$) δ -60.52 (s, 3F); EI: 249.0 (M^+); ^{18}O content: 89% determined by GC-MS spectroscopy.

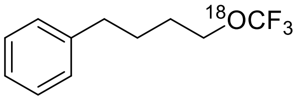

^{18}O-5-30

^{18}O-(4-(trifluoromethoxy)butyl)benzene: 52%; ^{1}H NMR (400 MHz, $CDCl_3$) δ 7.29 (t, *J* = 7.4 Hz, 2H), 7.22 – 7.14 (m, 3H), 3.96 (t, *J* = 5.9 Hz, 2H), 2.65 (t, *J* = 7.0 Hz, 2H), 1.81 – 1.65 (m, 4H); ^{19}F NMR (376 MHz, $CDCl_3$) δ -60.73 (s, 3F); EI: 220.1 (M^+); ^{18}O content: 89% determined by GC-MS spectroscopy.

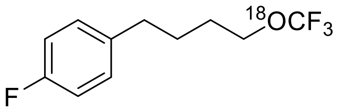

^{18}O-5-31

^{18}O-1-fluoro-4-(4-(trifluoromethoxy)butyl)benzene: 46%; ^{1}H NMR (400 MHz, $CDCl_3$) δ 7.17 – 7.06 (m, 2H), 6.97 (t, *J* = 8.6 Hz, 2H), 3.96 (t, *J* = 5.5 Hz, 2H), 2.62 (t, *J* = 6.4 Hz, 2H), 1.74 – 1.67 (m, 4H); ^{19}F NMR (376 MHz, $CDCl_3$) δ -60.80 (s, 3F), -117.51 – -117.65 (m, 1F); EI: 238.1 (M^+); ^{18}O content: 89% determined by GC-MS spectroscopy.

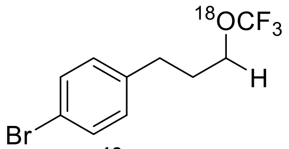

^{18}O-5-32

1-bromo-4-(3-(trifluoromethoxy)propyl)benzene: 52%;^{1}H NMR (400 MHz, $CDCl_3$) δ 7.42 (d, *J* = 8.2 Hz, 2H), 7.06 (d, *J* = 8.2 Hz, 2H), 3.94 (t, *J* = 6.2 Hz, 2H), 2.69 (t, *J* = 7.6 Hz, 2H), 2.05 – 1.88

(m, 2H); ^{19}F NMR (376 MHz, $CDCl_3$) δ -60.82 (s, 3F); EI: 284.0 (M^+); ^{18}O content: 89% determined by GC-MS spectroscopy.

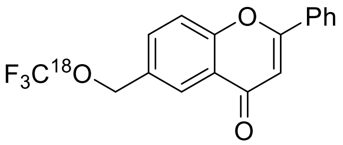

^{18}O-5-34

^{18}O-2-phenyl-6-((trifluoromethoxy)methyl)-4H-chromen-4-one: 58%; ^{1}H NMR (400 MHz, $CDCl_3$) δ 8.20 (d, *J* = 2.1 Hz, 1H), 7.90 (dd, *J* = 7.6, 1.8 Hz, 2H), 7.70 (dd, *J* = 8.6, 2.1 Hz, 1H), 7.60 (d, *J* = 8.6 Hz, 1H), 7.55 – 7.46 (m, 3H), 6.82 (s, 1H), 5.06 (s, 2H); ^{19}F NMR (376 MHz, $CDCl_3$) δ -60.49 (s, 3F); EI: 322.2 (M^+); ^{18}O content: 88% determined by GC-MS spectroscopy.

Deoxygenation of diphenyl sulfoxide

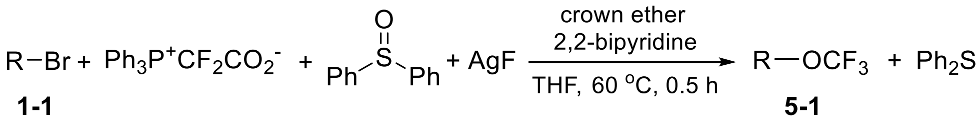

1-1 **5-1**

Into a 10 mL sealed tube were added benzyl bromide **1-1** (0.2 mmol, 49.4 mg, 1.0 equiv.), $Ph_3P^+CF_2CO_2^-$ (0.5 mmol, 178.0 mg, 2.5 equiv), Ph_2S=O (0.5 mmol, 101.2 mg, 2.5 equiv), AgF (0.4 mmol, 51.0 mg, 2.0 equiv), 2,2'-bipyridine (0.3 mmol, 47.0 mg, 1.5 equiv), 2,3,11,12-dibenzo-18-crown-6 (0.1 mmol, 36.0 mg, 0.5 equiv) and THF (1.5 mL) under a N_2 atmosphere. The tube was sealed and the reaction mixture was stirred at 60 °C for 30 min., and the mixture was cooled to room temperature. Product **5-1** (35 mg, 69%), Ph_2S (51 mg, 90% yield based on Ph_2S=O consumed) and remaining Ph_2S=O (40 mg, 39% remained) were isolated by flash column chromatography.

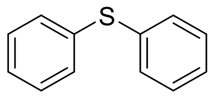

Diphenylsulfane: ^{1}H NMR (400 MHz, $CDCl_3$) δ 7.48 – 7.41 (m, 4H), 7.41 – 7.35 (m, 4H), 7.35 – 7.29 (m, 2H); ^{13}C NMR (101 MHz, $CDCl_3$) δ 135.9 (s), 131.1 (s), 129.3 (s), 127.1 (s); GC-MS (EI): 186.1 (M^+).

A stepwise reaction for the observation of $AgOCF_3$ complex

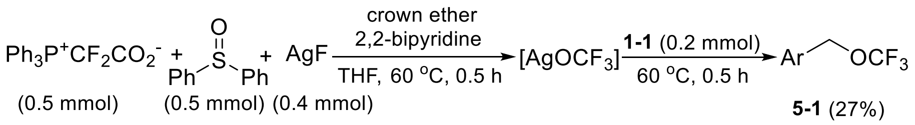

For the first step, the same two reactions were performed: Into a 10 mL sealed tube were added $Ph_3P^+CF_2CO_2^-$ (0.5 mmol, 178.0 mg, 2.5 equiv), $Ph_2S{=}O$ (0.5 mmol, 101.2 mg, 2.5 equiv), AgF (0.4 mmol, 51.0 mg, 2.0 equiv), 2,2'-bipyridine (0.3 mmol, 47.0 mg, 1.5 equiv), 2,3,11,12-dibenzo-18-crown-6 (0.1 mmol, 36.0 mg, 0.5 equiv) and THF (1.5 mL) under a N_2 atmosphere. The tube was sealed and the reaction mixture was stirred at 60 ^oC for 30 min. One of the reaction systems was monitored by ^{19}F NMR spectroscopy to confirm the generation of $AgOCF_3$ complex, and the ^{19}F NMR spectrum is shown as follows. For the other system, the substrate was added to give the final product. The procedure is shown as follows: After the mixture was cooled to room temperature, benzyl bromide **1-1** (0.2 mmol, 49.4 mg, 1.0 equiv.) was added under a N_2 atmosphere and the reaction mixture was stirred at 60 ^oC for 30 min. The mixture was cooled to room temperature. The yield of the desired product was determined by ^{19}F NMR spectroscopy.

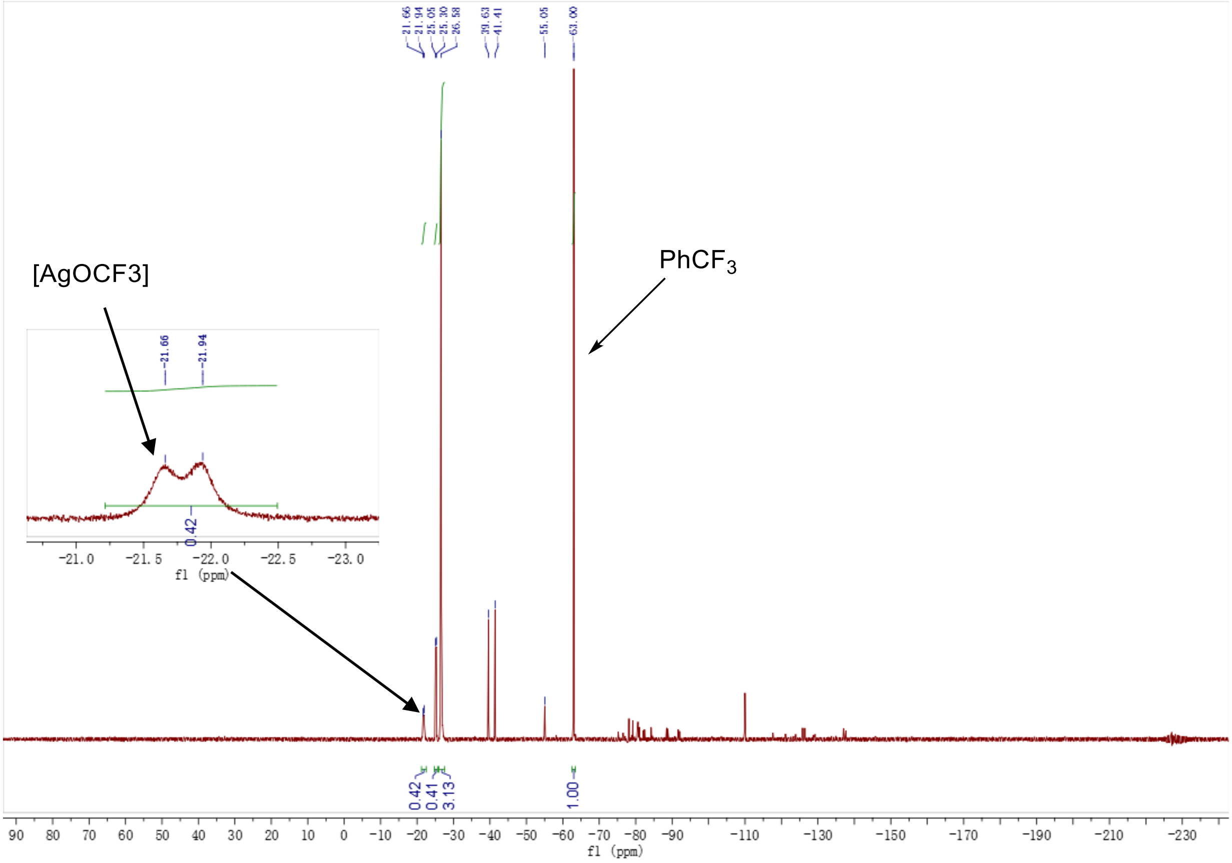

Supplementary Figure 2. ^{19}F NMR (376 MHz, THF) spectrum of $AgOCF_3$ complex

DFT calculations[17]: optimized geometrical coordinates and calculated total energies

Relative free energies for the generation of difluorocarbene

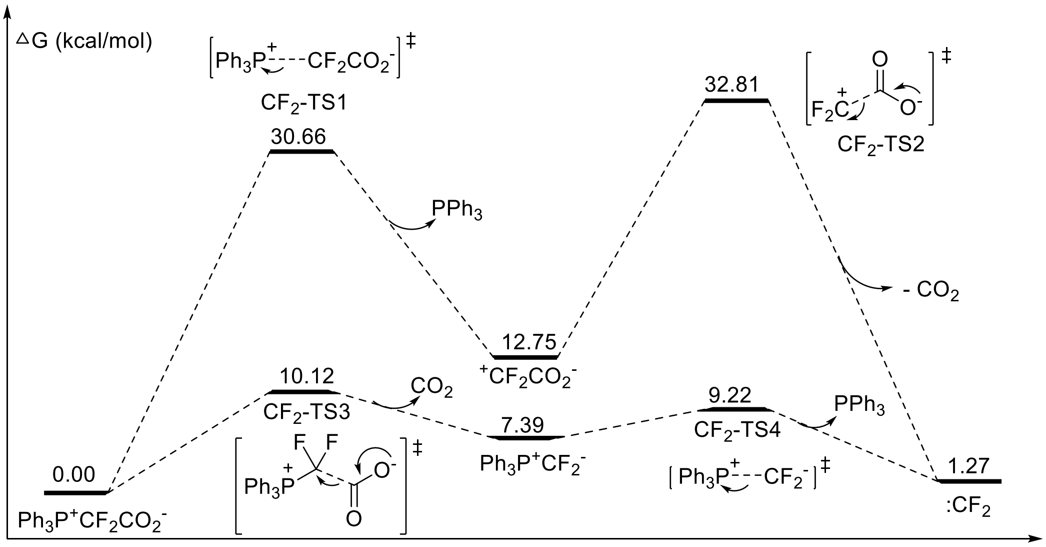

Supplementary Figure 3. Relative free energies for the generation of difluorocarbene from $Ph_3P^+CF_2CO_2^-$ calculated at the M062X//6-31++G(d,p)/LANL2DZ level in tetrahydrofuran with the solution model of SMD. All

calculations were performed in Gaussian 09 D01 package.

Relative free energies for trifluoromethoxylation

As shown in Table 1 in the main text, the presence of ligands significantly increased the yield. However, calculations indicated that there were only negligible differences in the relative free energies irrespective of whether Ag complexes were coordinated to ligands or not (Figure S2 for the case with 2,2'-bipyridine as a ligand). Apparently, ligands increased the solubility and stability of $AgOCF_3$ complex in the solvent, and thus higher yields were obtained. But DFT calculations usually do not take the solubility into account, and therefore only negligible differences in the relative free energies were observed.

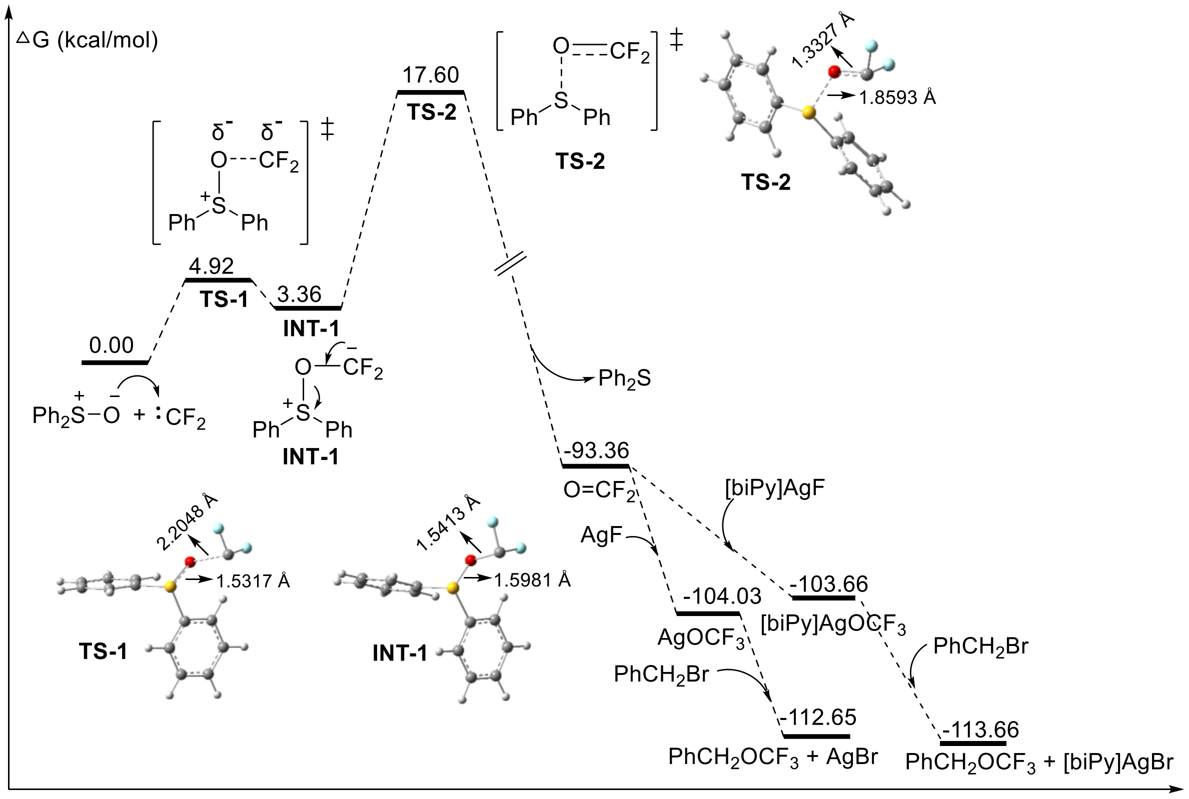

Supplementary Figure 4. Relative free energies for trifluoromethoxylation calculated at the M062X//6-31++G(d,p)/LANL2DZ level in tetrahydrofuran with the solution model of SMD. All calculations were performed in Gaussian 09 D01 package. [biPy] = 2,2'-bipyridine.

.

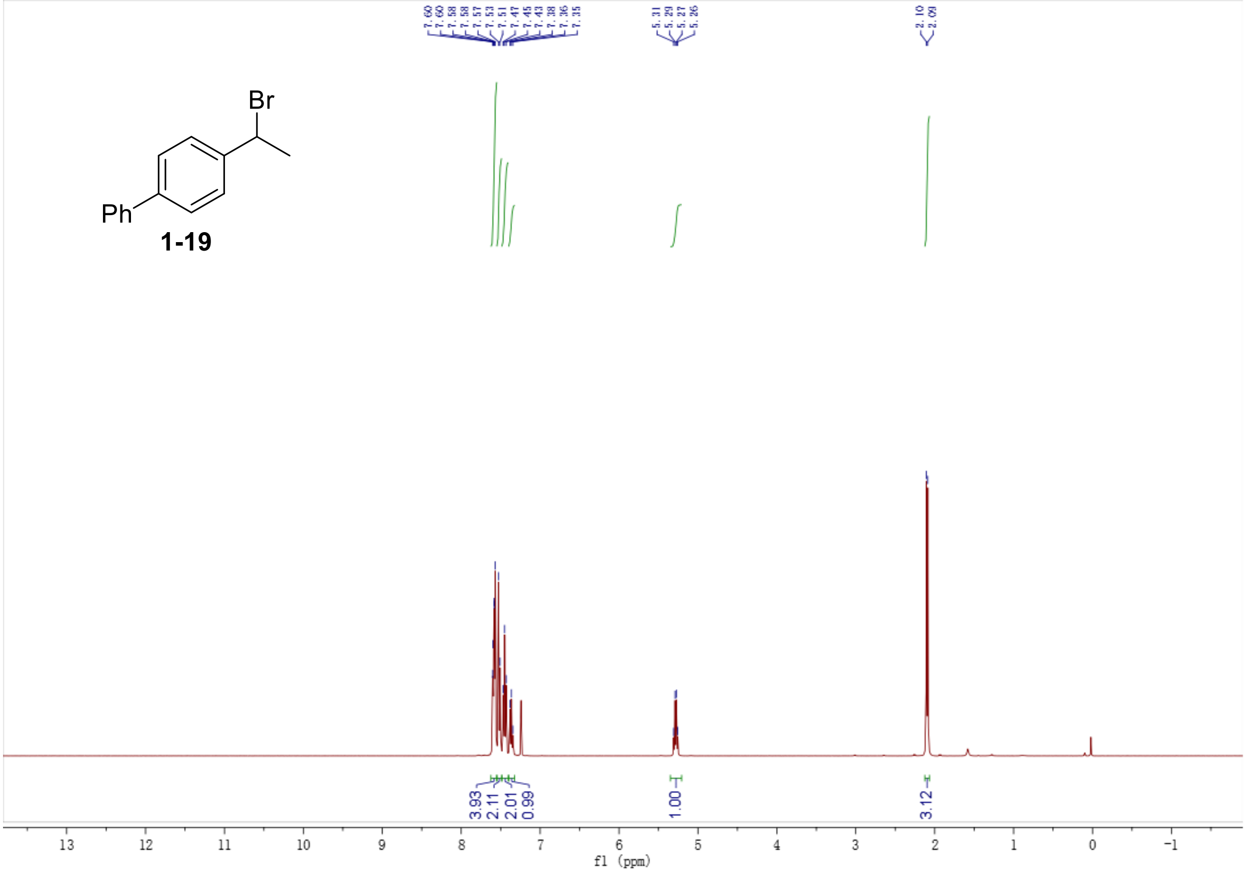

Supplementary Figure 5. ^{1}H NMR spectrum (400 MHz, $CDCl_3$) of **1-19**

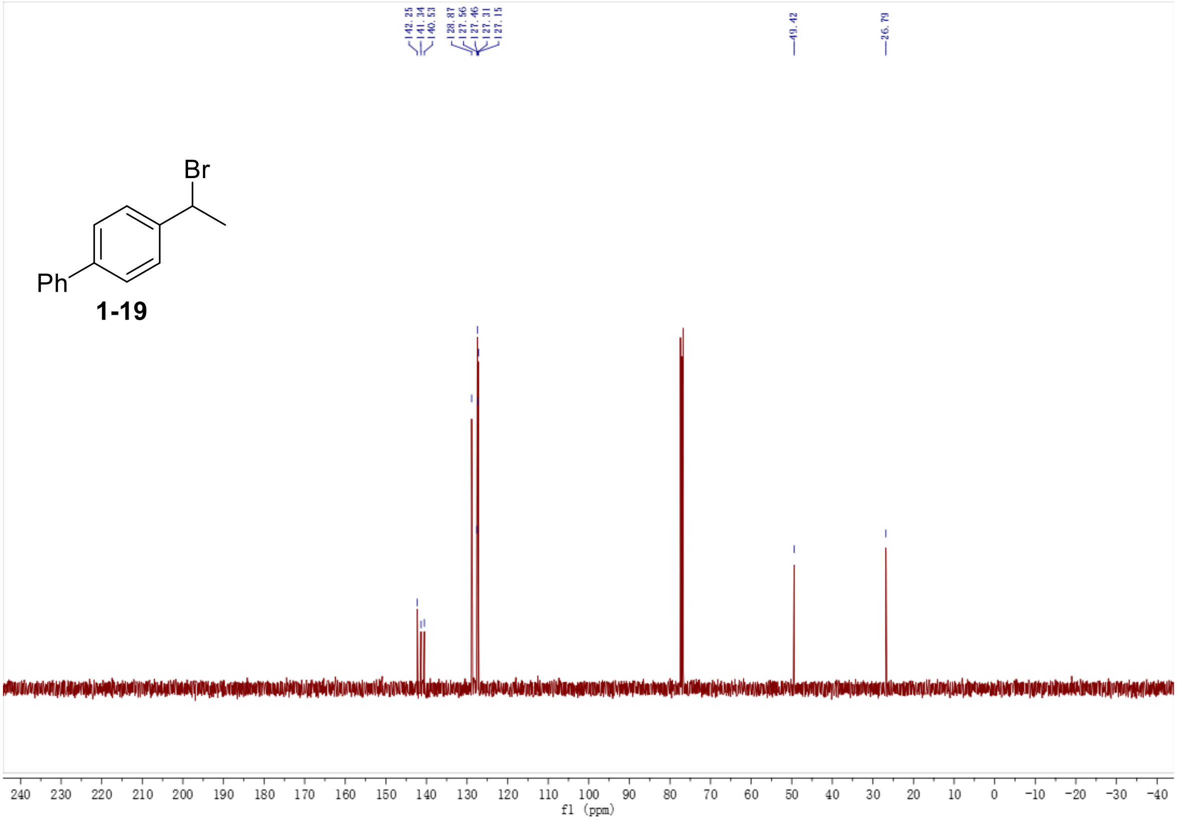

Supplementary Figure 6. ^{13}C NMR spectrum (101 MHz, $CDCl_3$) of **1-19**

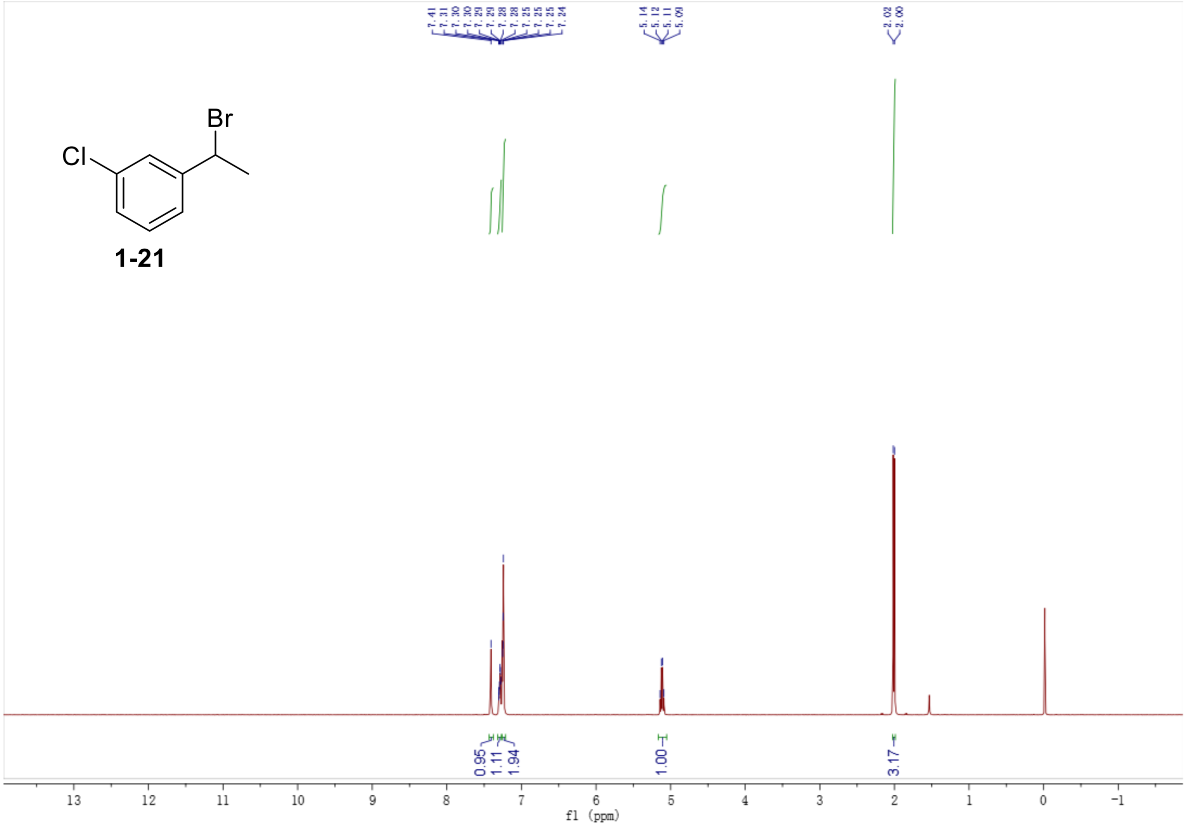

Supplementary Figure 7. ^{1}H NMR spectrum (400 MHz, $CDCl_3$) of **1-21**

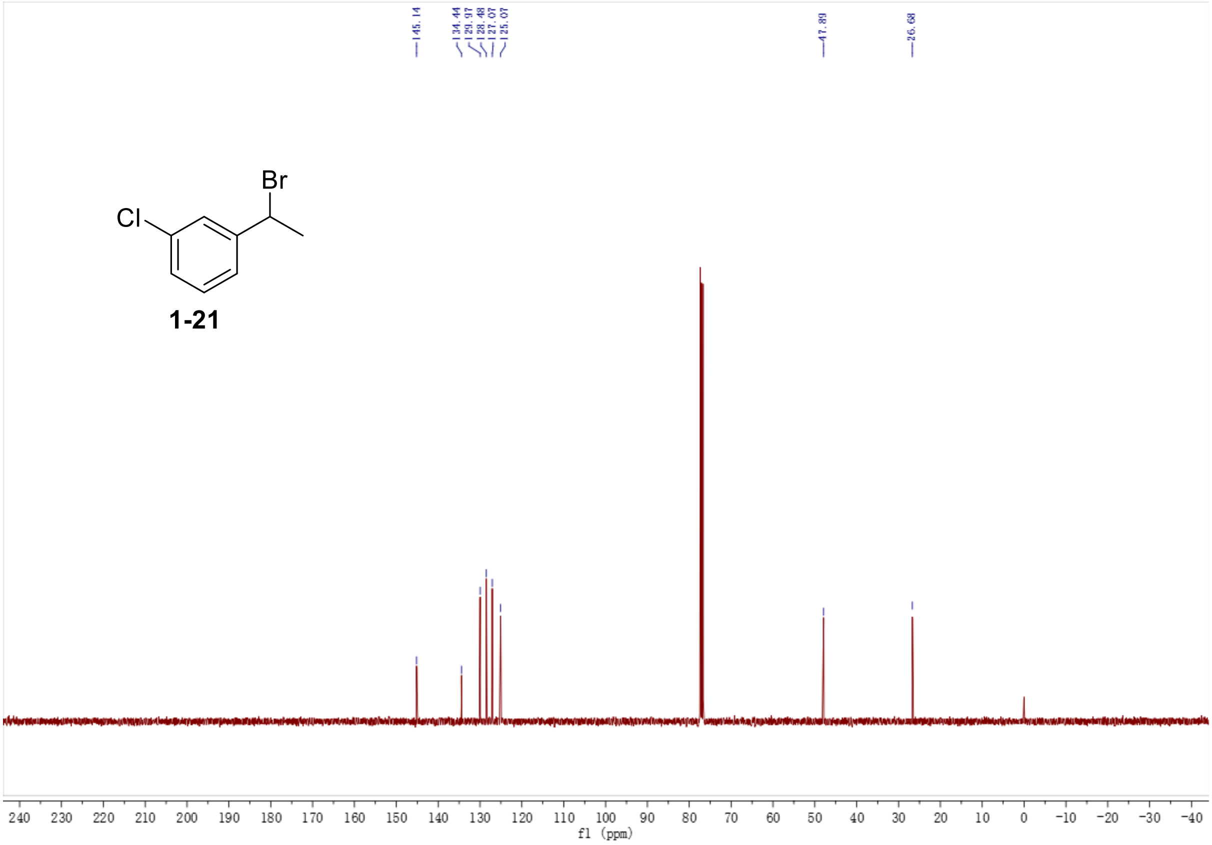

Supplementary Figure 8. ^{13}C NMR spectrum (101 MHz, $CDCl_3$) of **1-21**

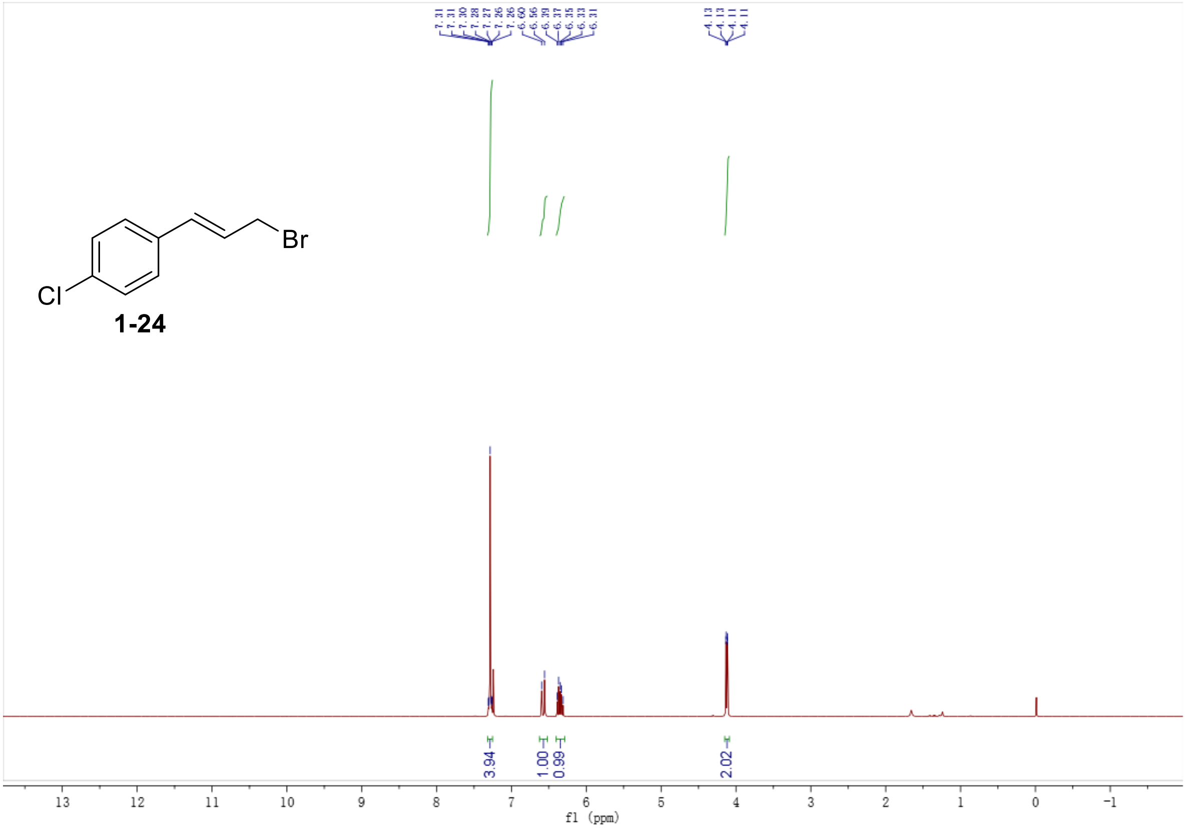

Supplementary Figure 9. ^{1}H NMR spectrum (400 MHz, $CDCl_3$) of **1-24**

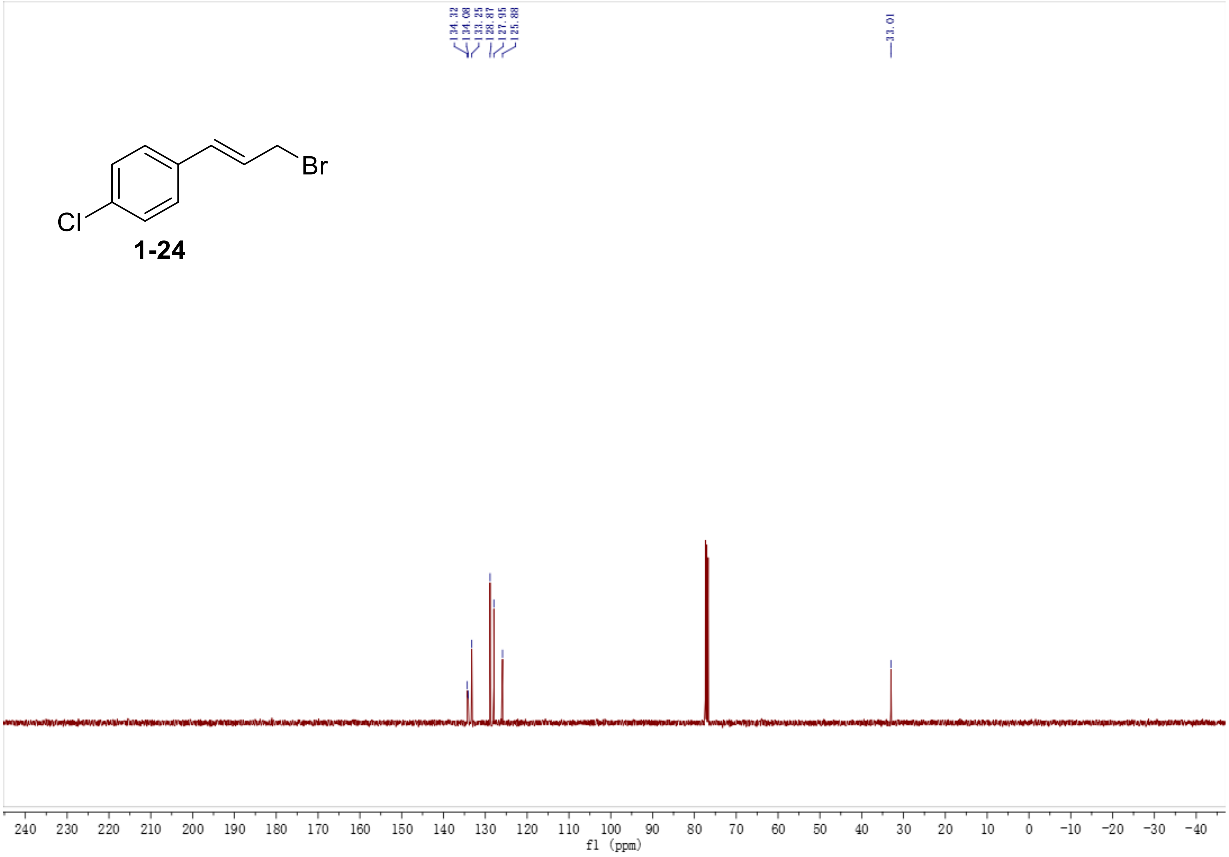

Supplementary Figure 10. ^{13}C NMR spectrum (101 MHz, $CDCl_3$) of **1-24**

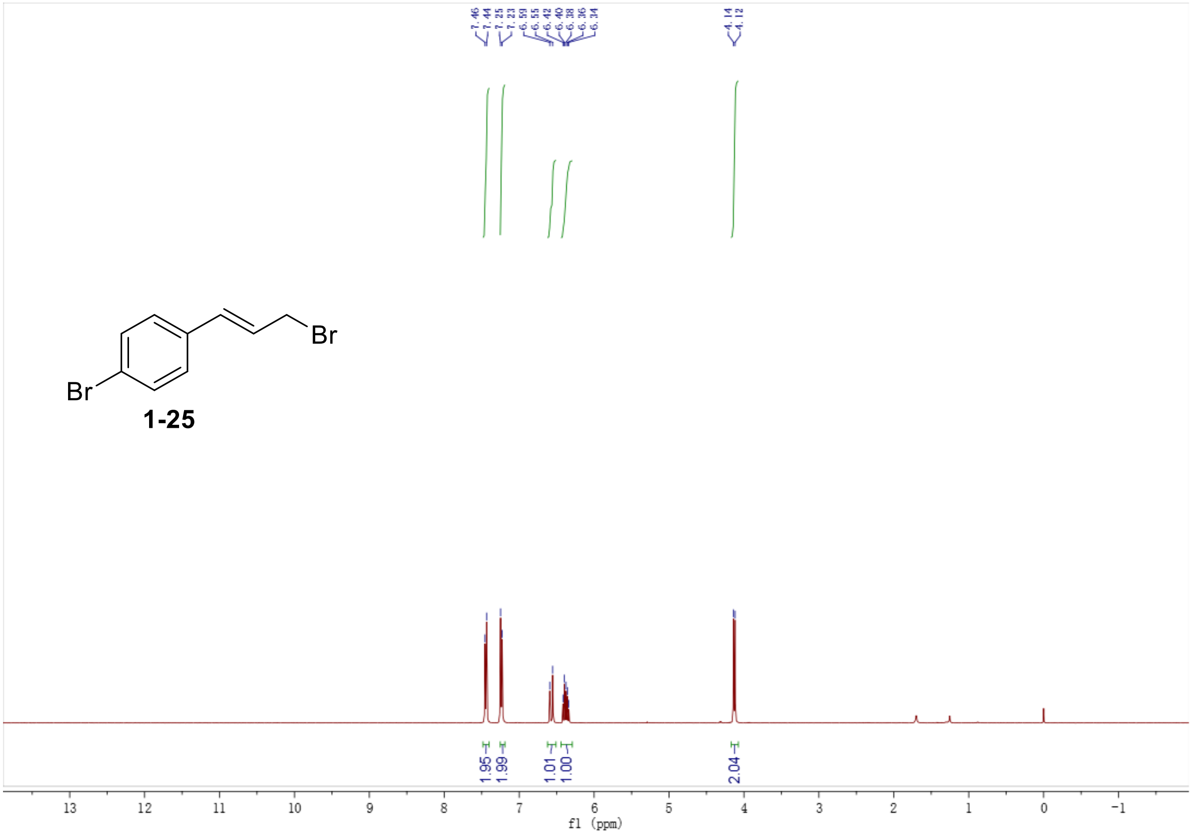

Supplementary Figure 11. ^{1}H NMR spectrum (400 MHz, $CDCl_3$) of **1-25**

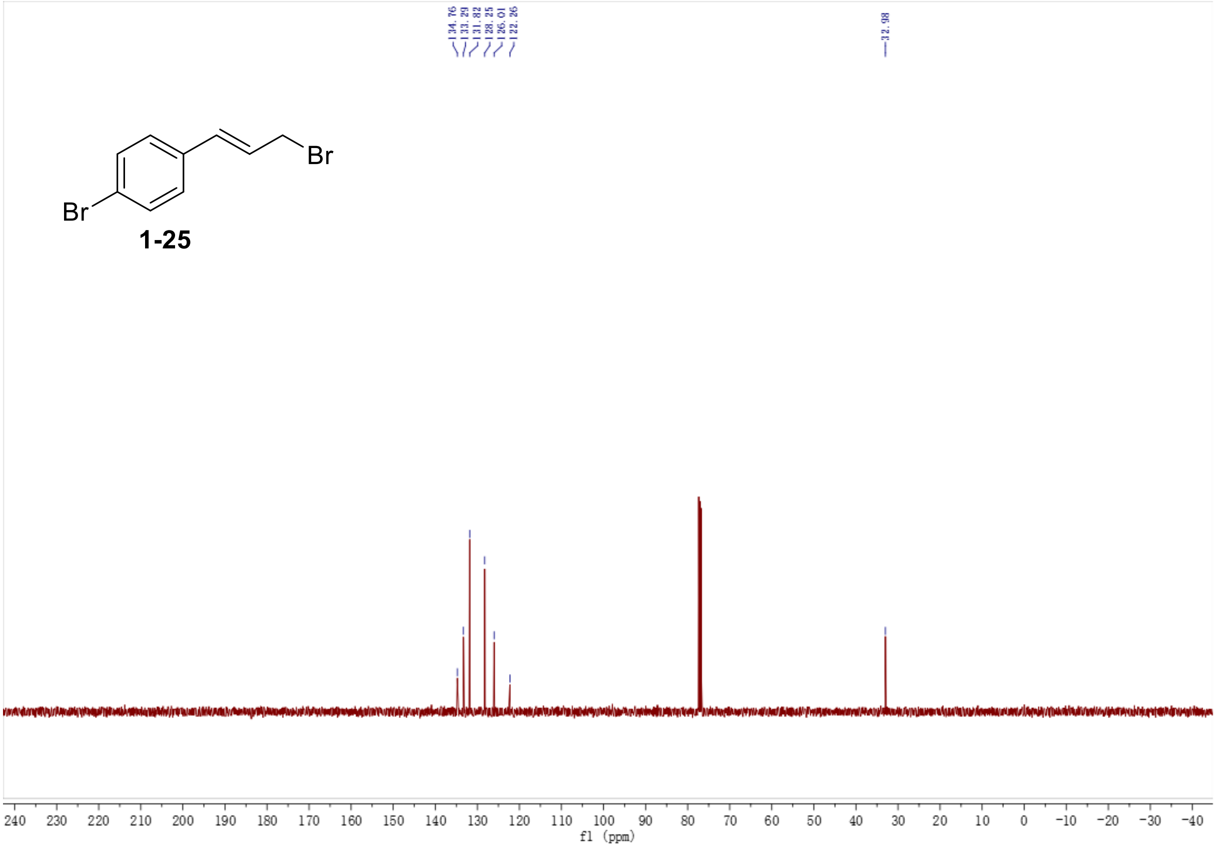

Supplementary Figure 12. ^{13}C NMR spectrum (101 MHz, $CDCl_3$) of **1-25**

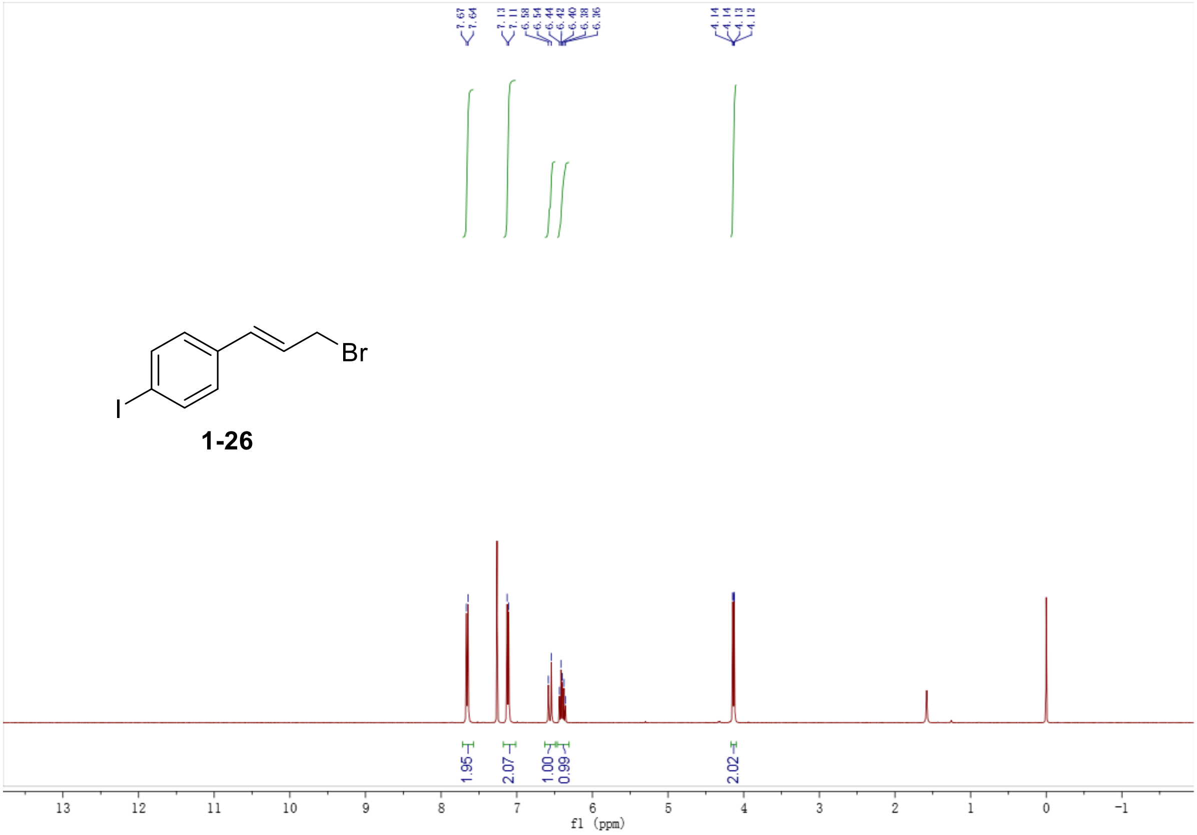

Supplementary Figure 13. ^{1}H NMR spectrum (400 MHz, $CDCl_3$) of **1-26**

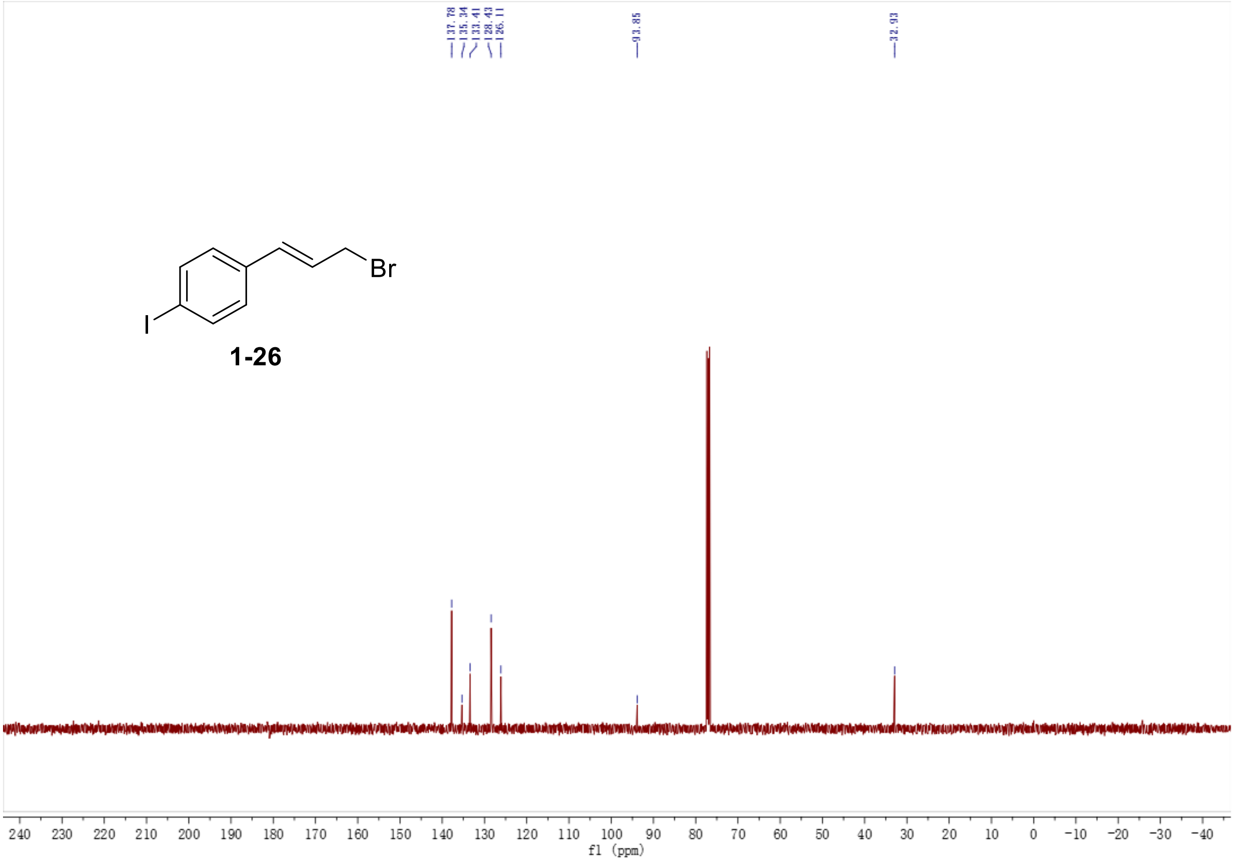

Supplementary Figure 14. ^{13}C NMR spectrum (101 MHz, $CDCl_3$) of **1-26**

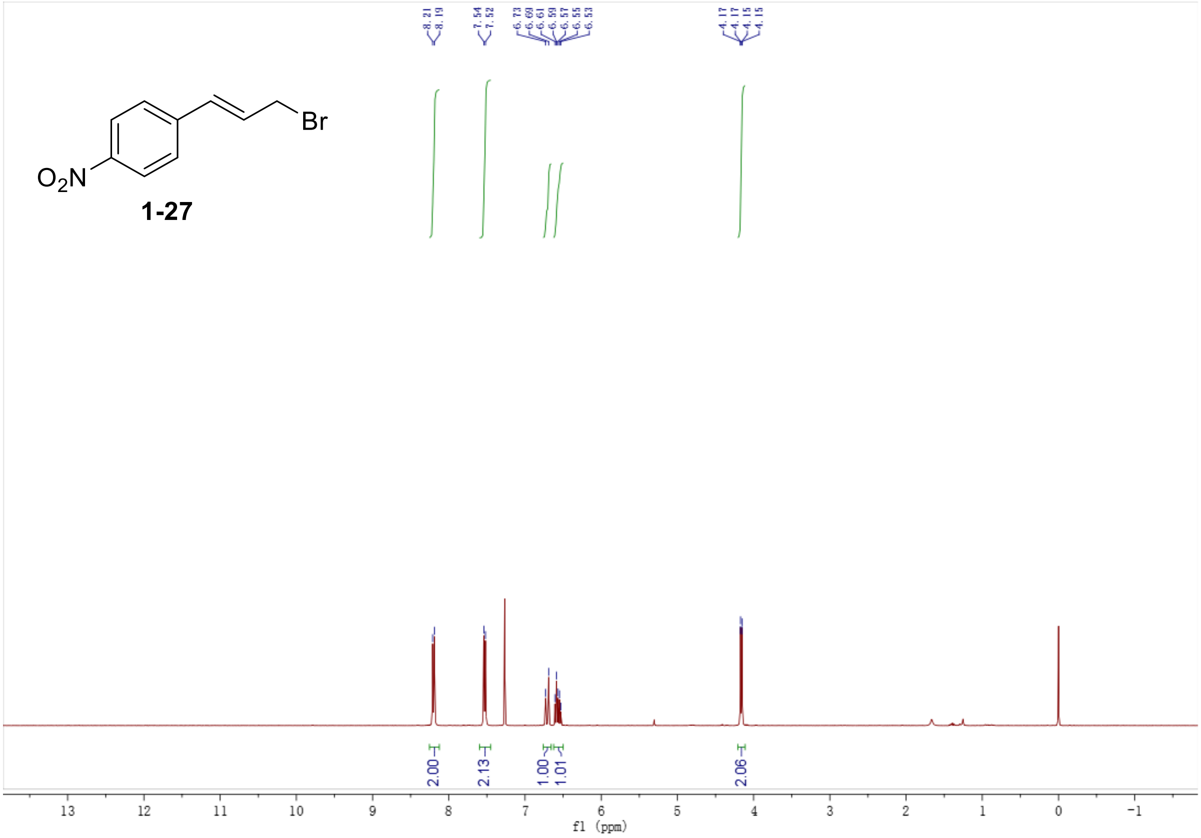

Supplementary Figure 15. ^{1}H NMR spectrum (400 MHz, $CDCl_3$) of **1-27**

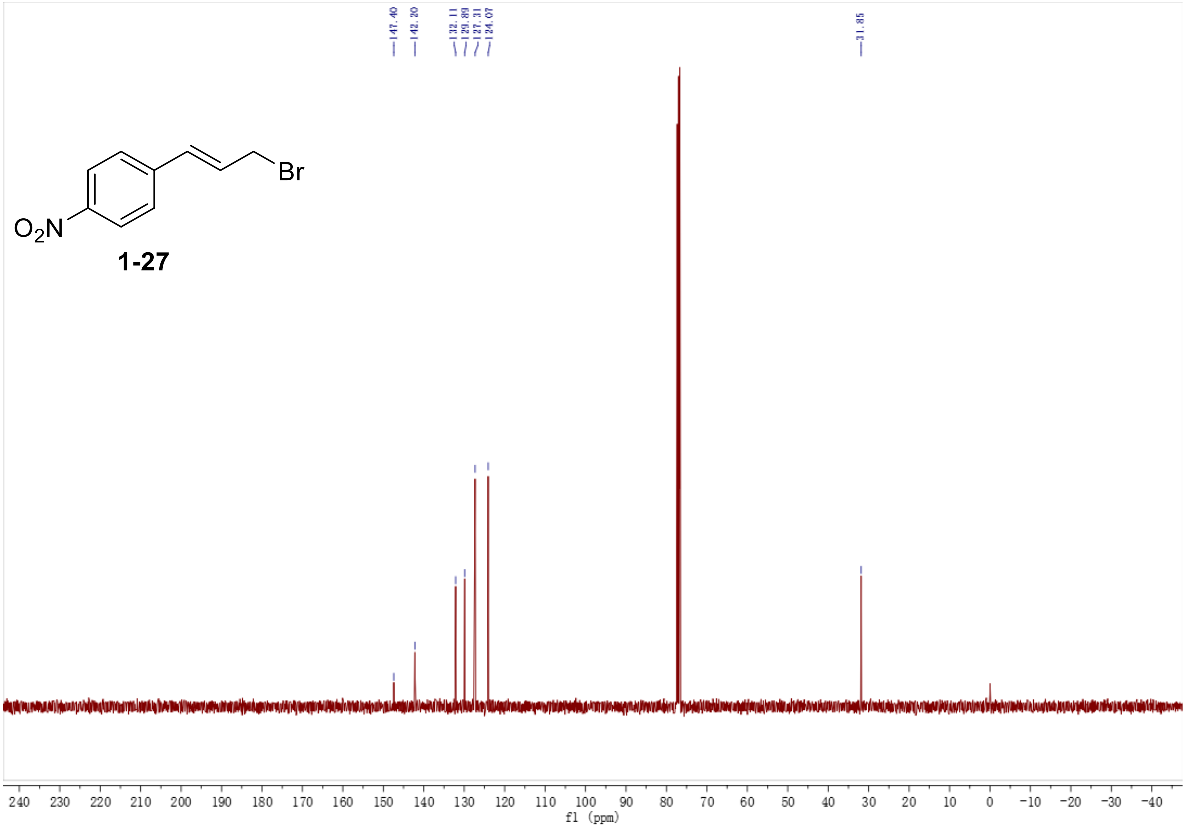

Supplementary Figure 16. ^{13}C NMR spectrum (101 MHz, $CDCl_3$) of **1-27**

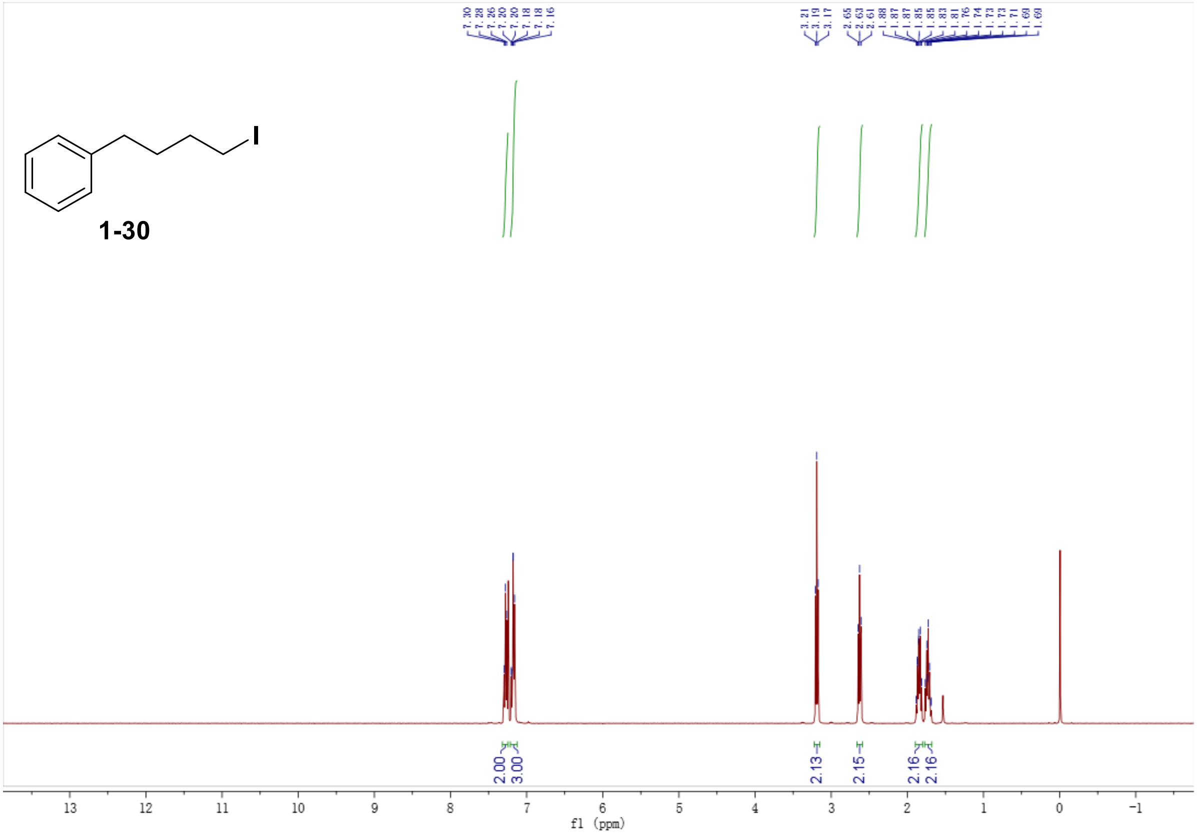

Supplementary Figure 17. ^{1}H NMR spectrum (400 MHz, $CDCl_3$) of **1-30**

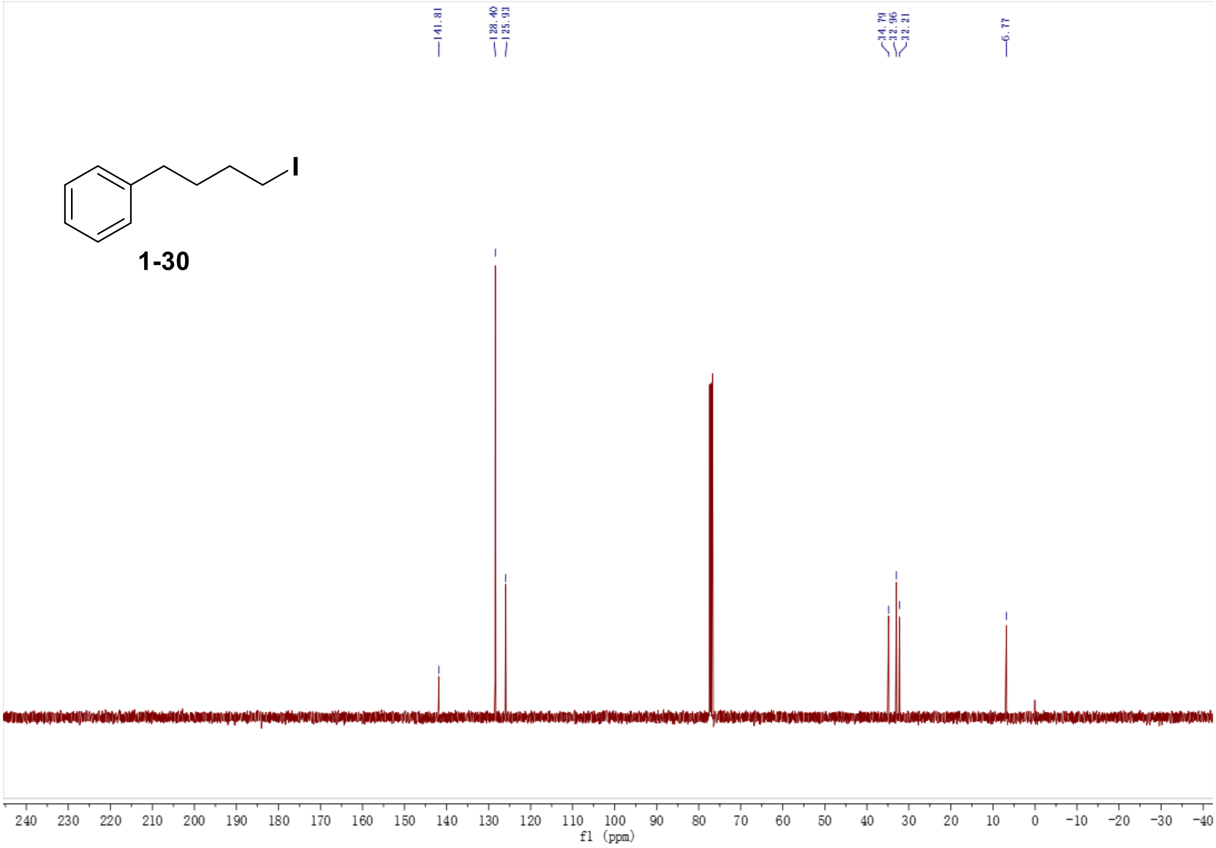

Supplementary Figure 18. ^{13}C NMR spectrum (101 MHz, $CDCl_3$) of **1-30**

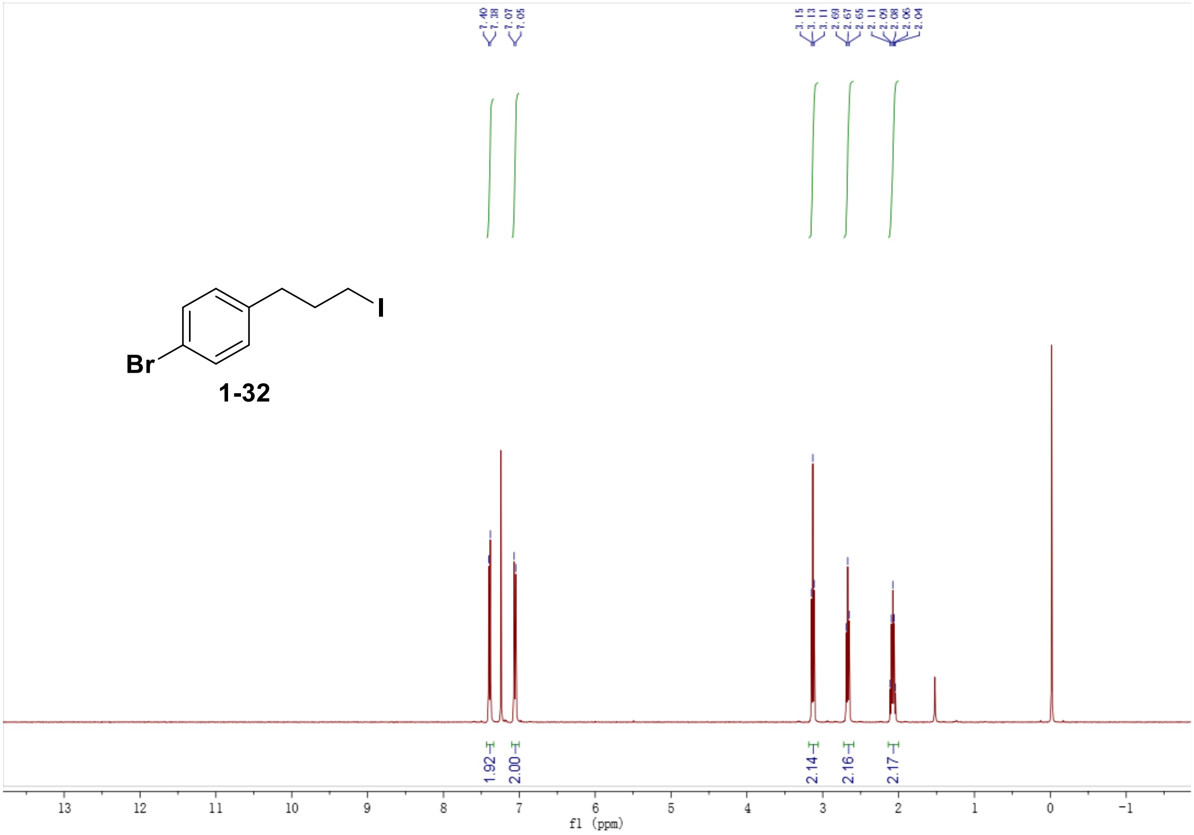

Supplementary Figure 19. ^{1}H NMR spectrum (400 MHz, $CDCl_3$) of **1-32**

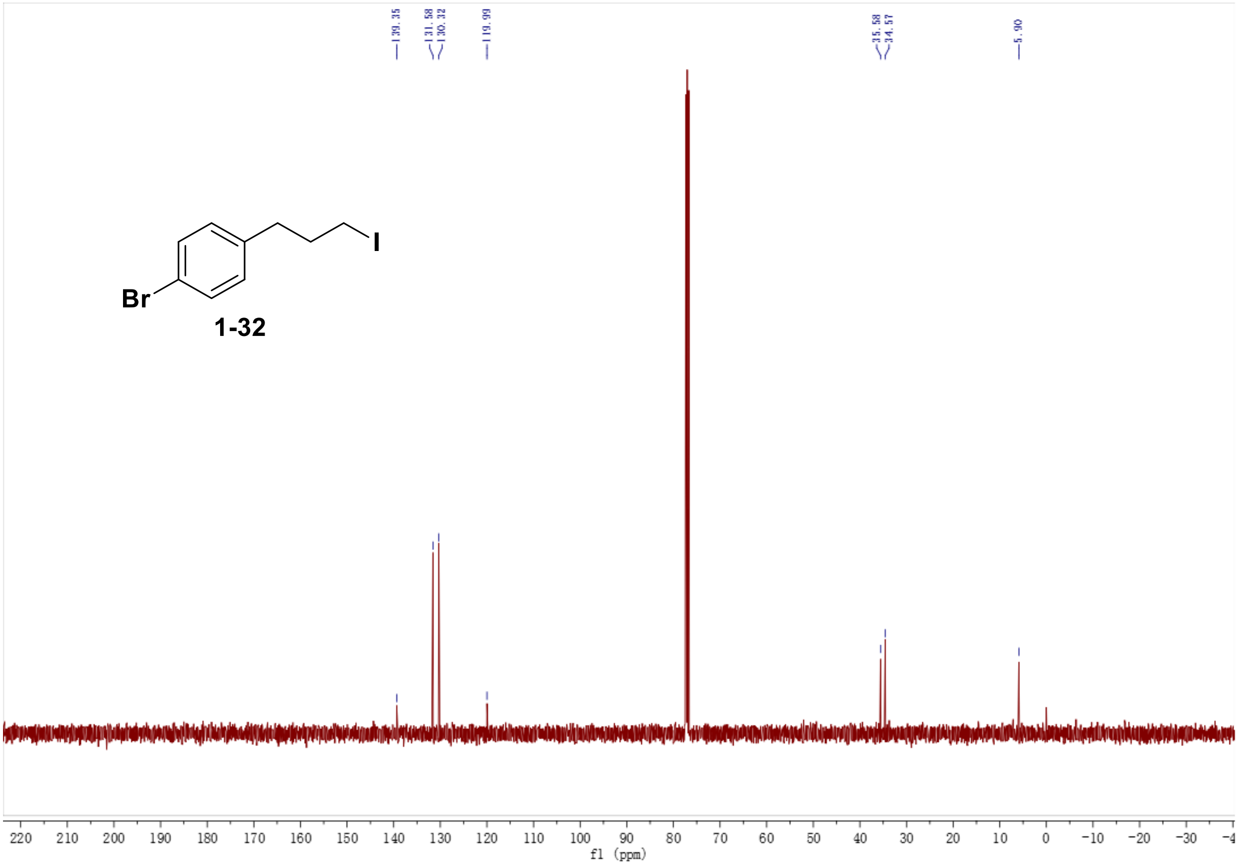

Supplementary Figure 20. ^{13}C NMR spectrum (101 MHz, $CDCl_3$) of **1-32**

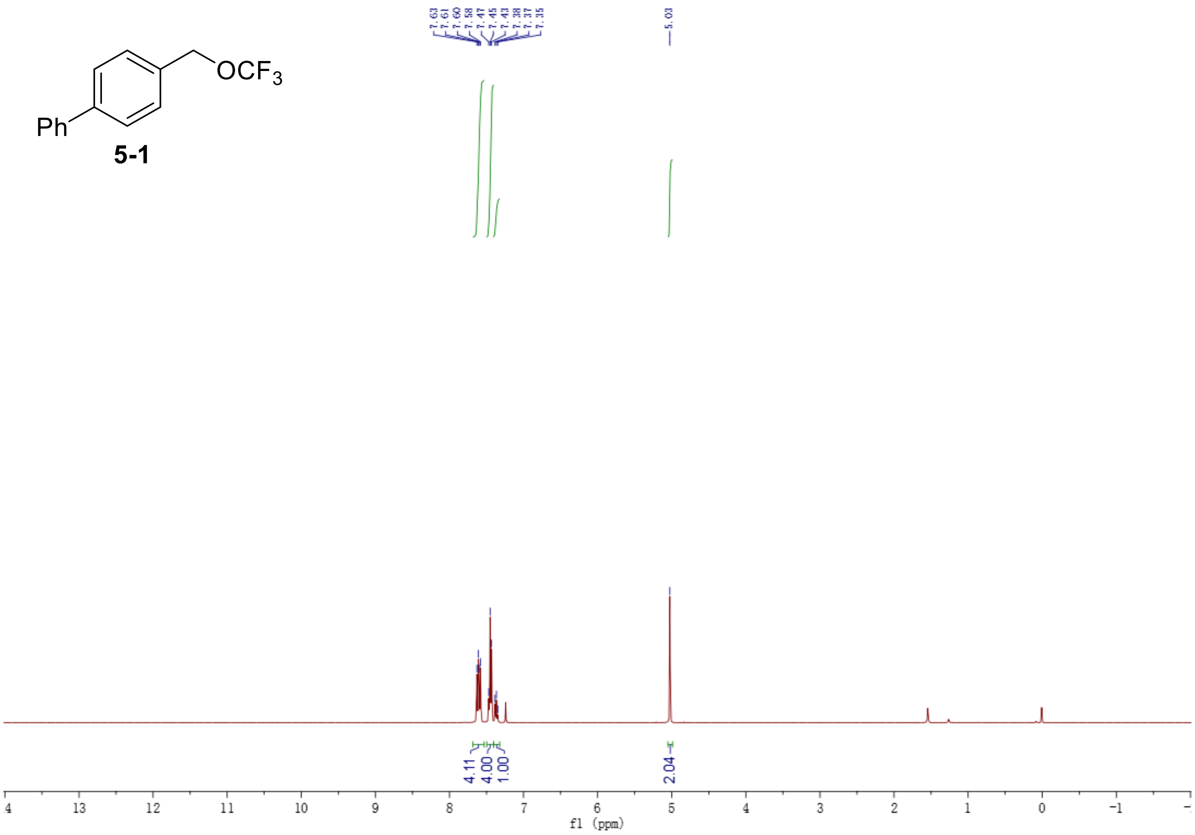

Supplementary Figure 21. ^{1}H NMR spectrum (400 MHz, $CDCl_3$) of **5-1**

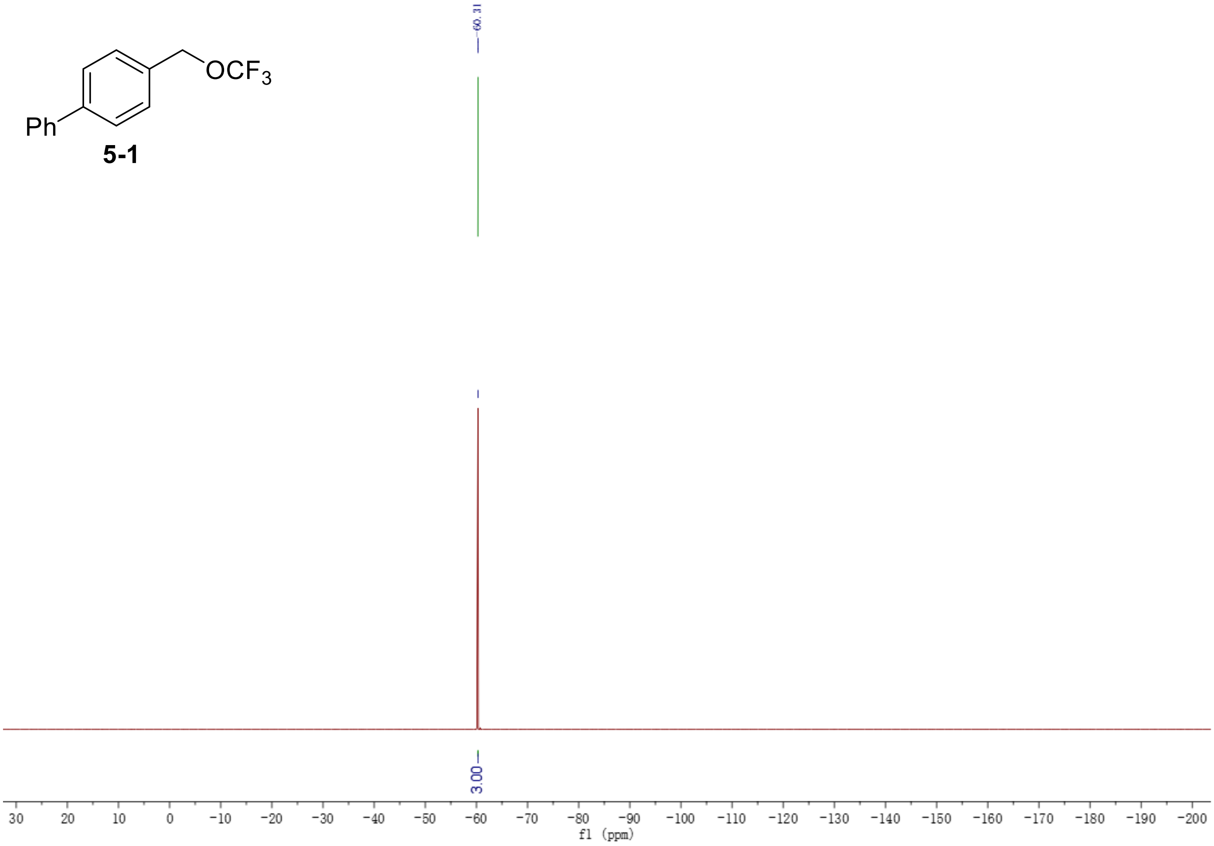

Supplementary Figure 22. ^{19}F NMR spectrum (376 MHz, $CDCl_3$) of **5-1**

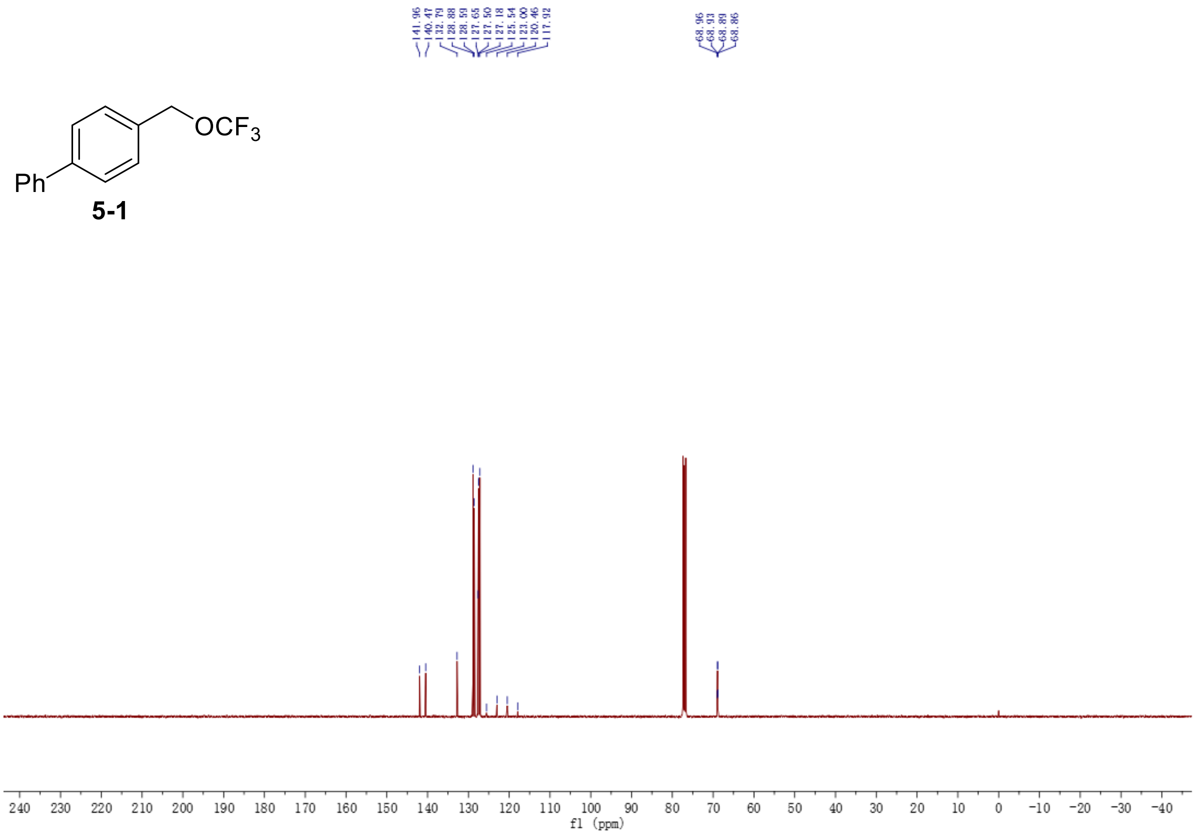

Supplementary Figure 23. ^{13}C NMR spectrum (101 MHz, $CDCl_3$) of **5-1**

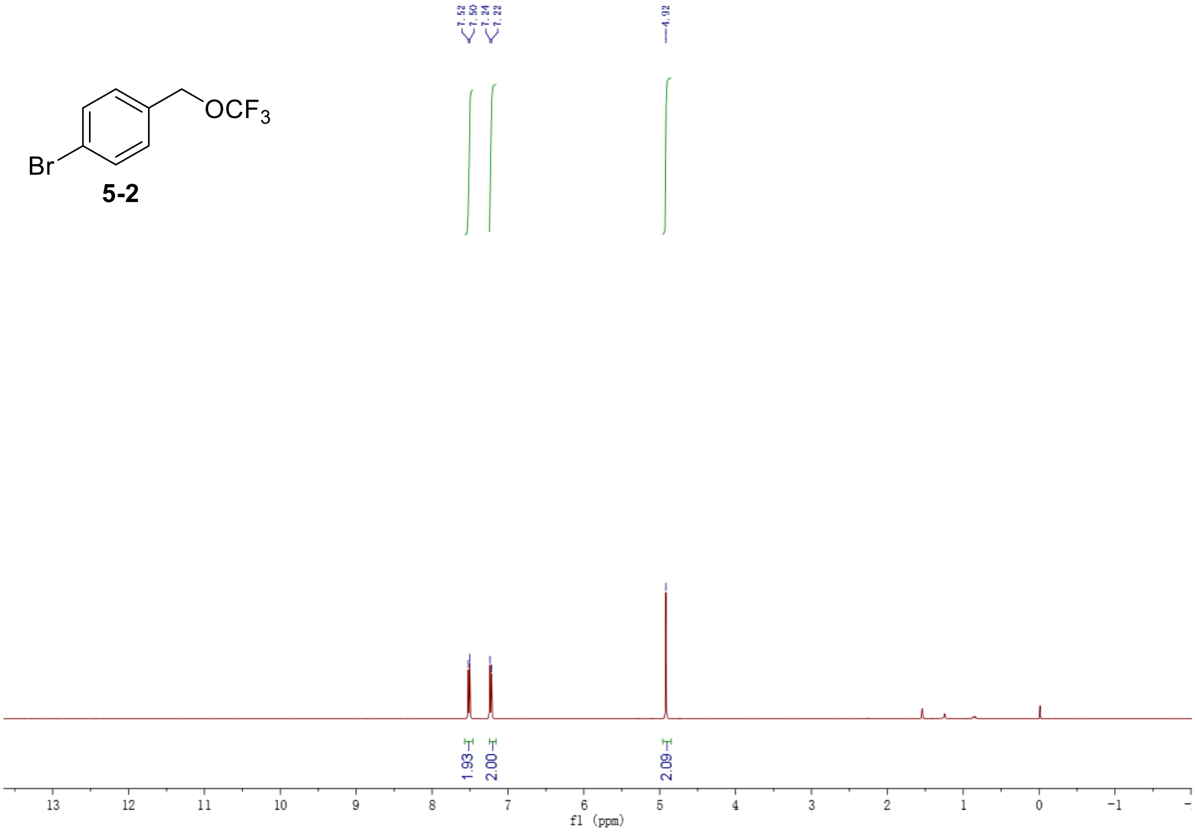

Supplementary Figure 24. ^{1}H NMR spectrum (400 MHz, $CDCl_3$) of **5-2**

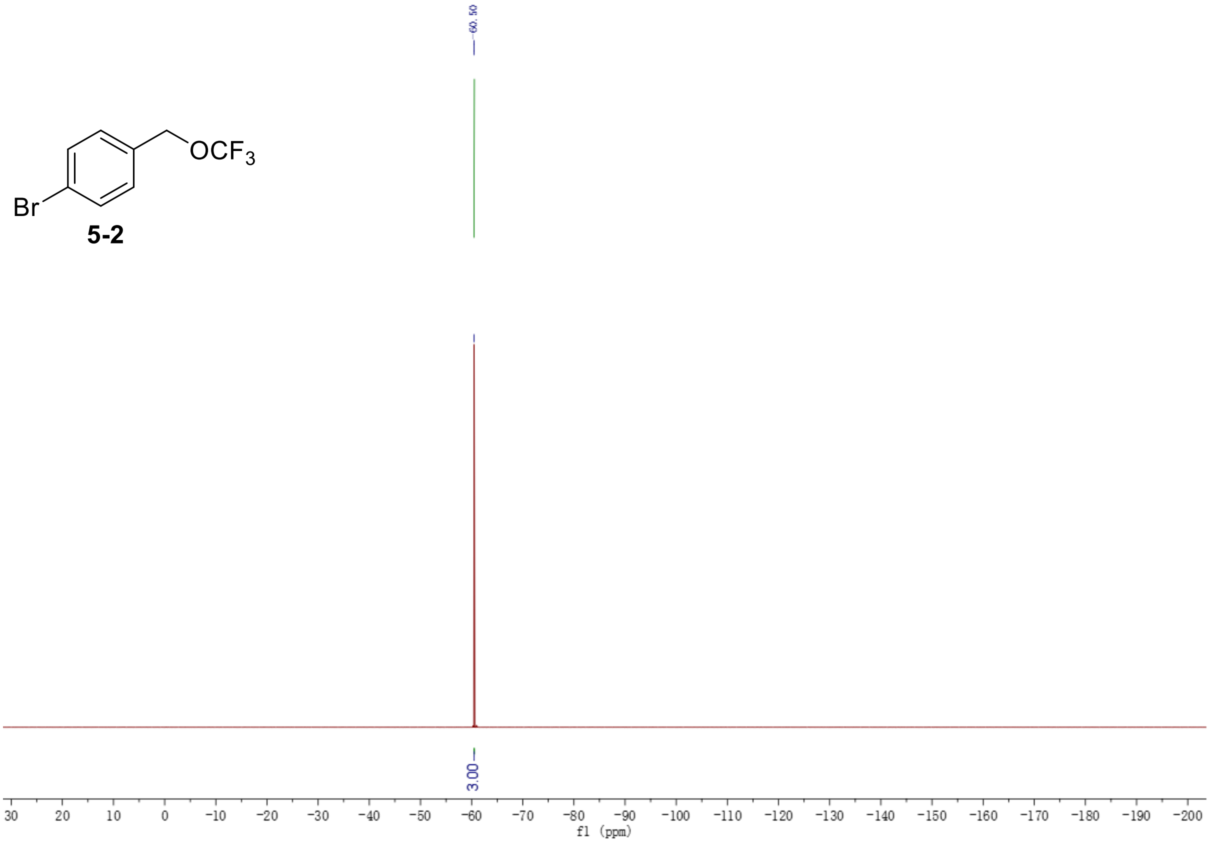

Supplementary Figure 25. ^{19}F NMR spectrum (376 MHz, $CDCl_3$) of **5-2**

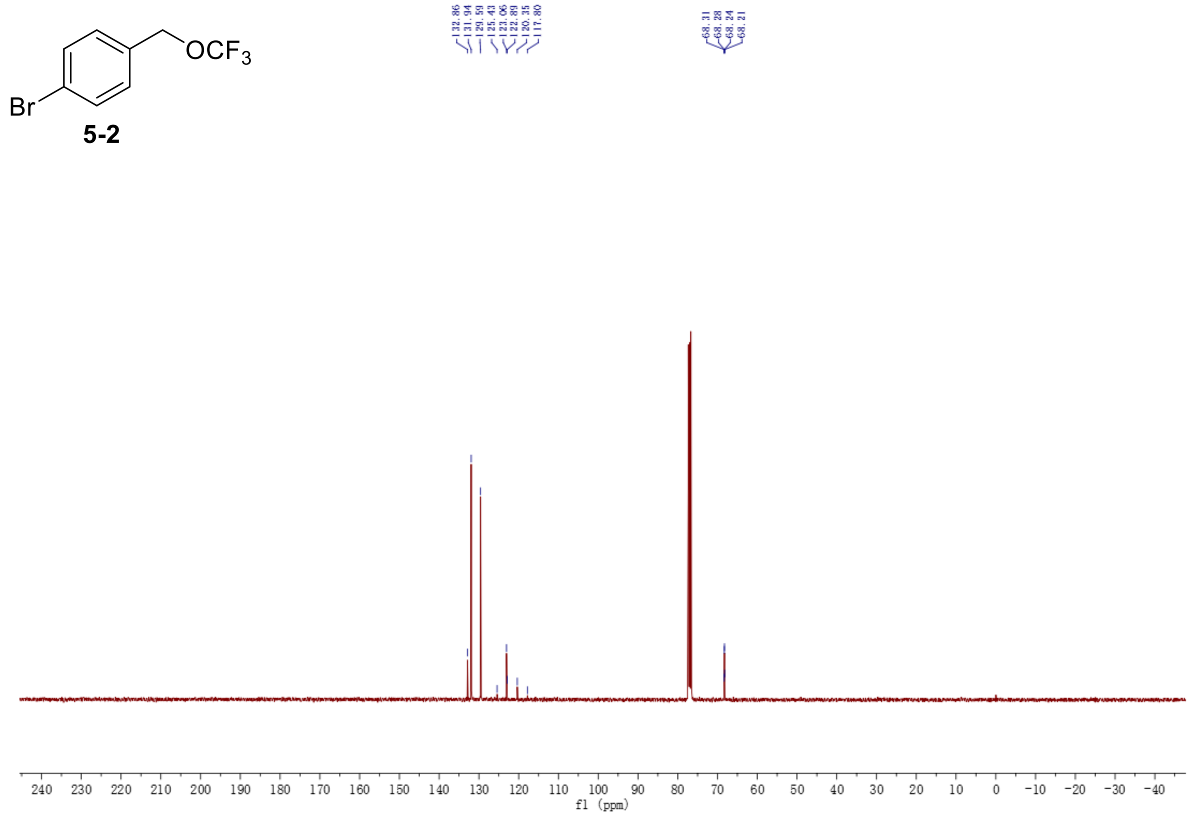

Supplementary Figure 26. ^{13}C NMR spectrum (101 MHz, $CDCl_3$) of **5-2**

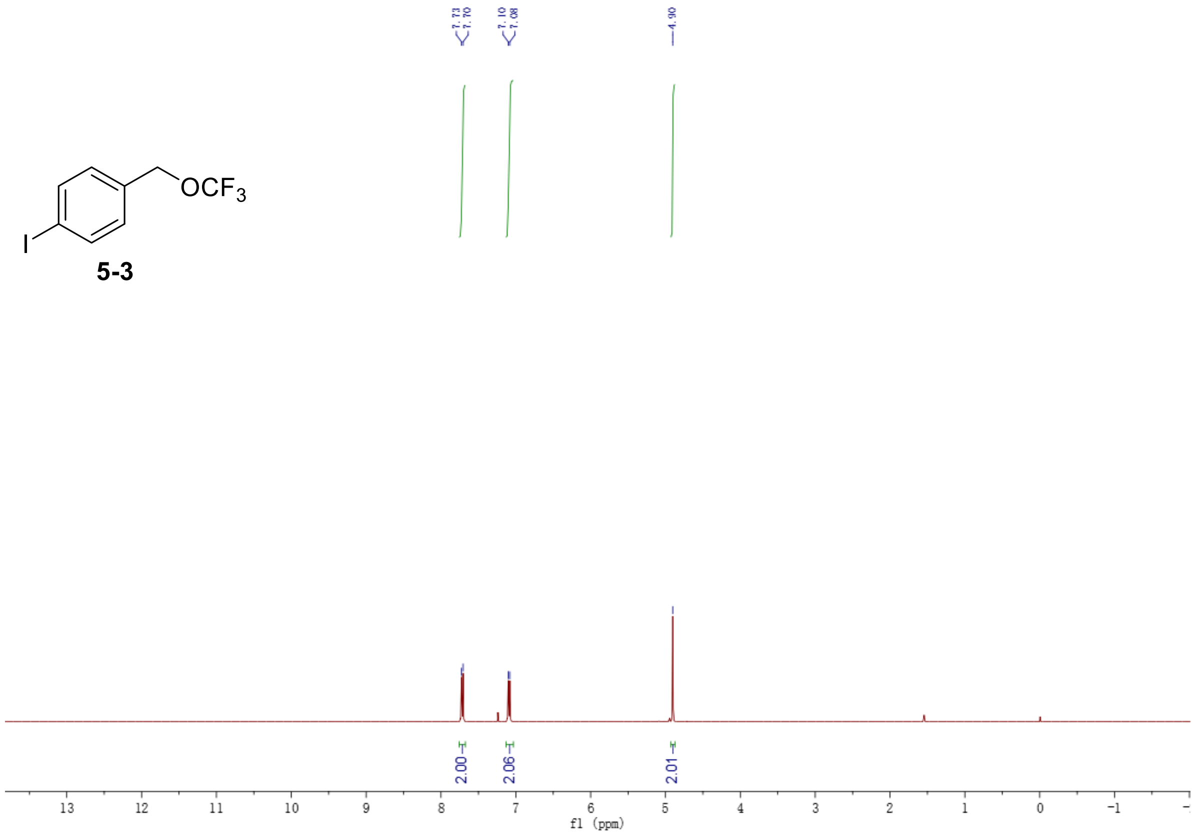

Supplementary Figure 27. ^{1}H NMR spectrum (400 MHz, $CDCl_3$) of **5-3**

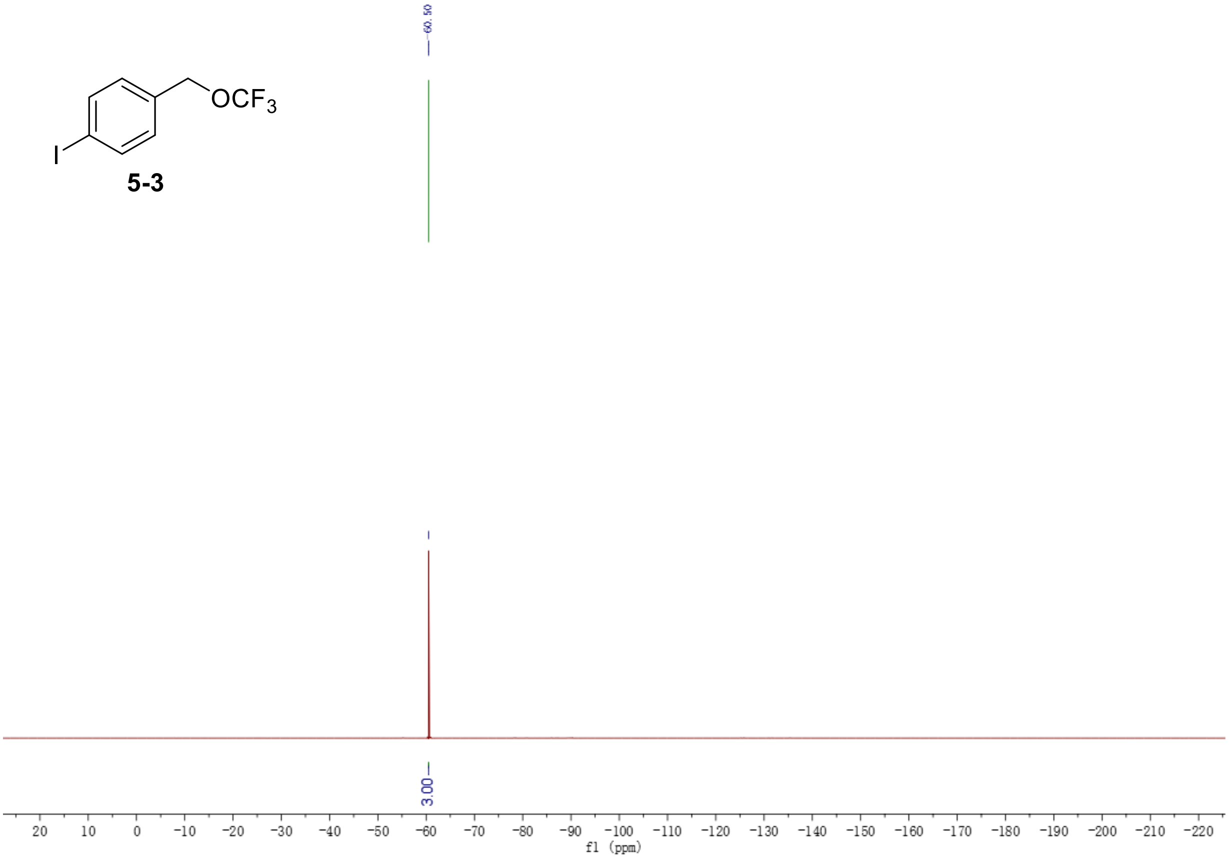

Supplementary Figure 28. ^{19}F NMR spectrum (376 MHz, $CDCl_3$) of **5-3**

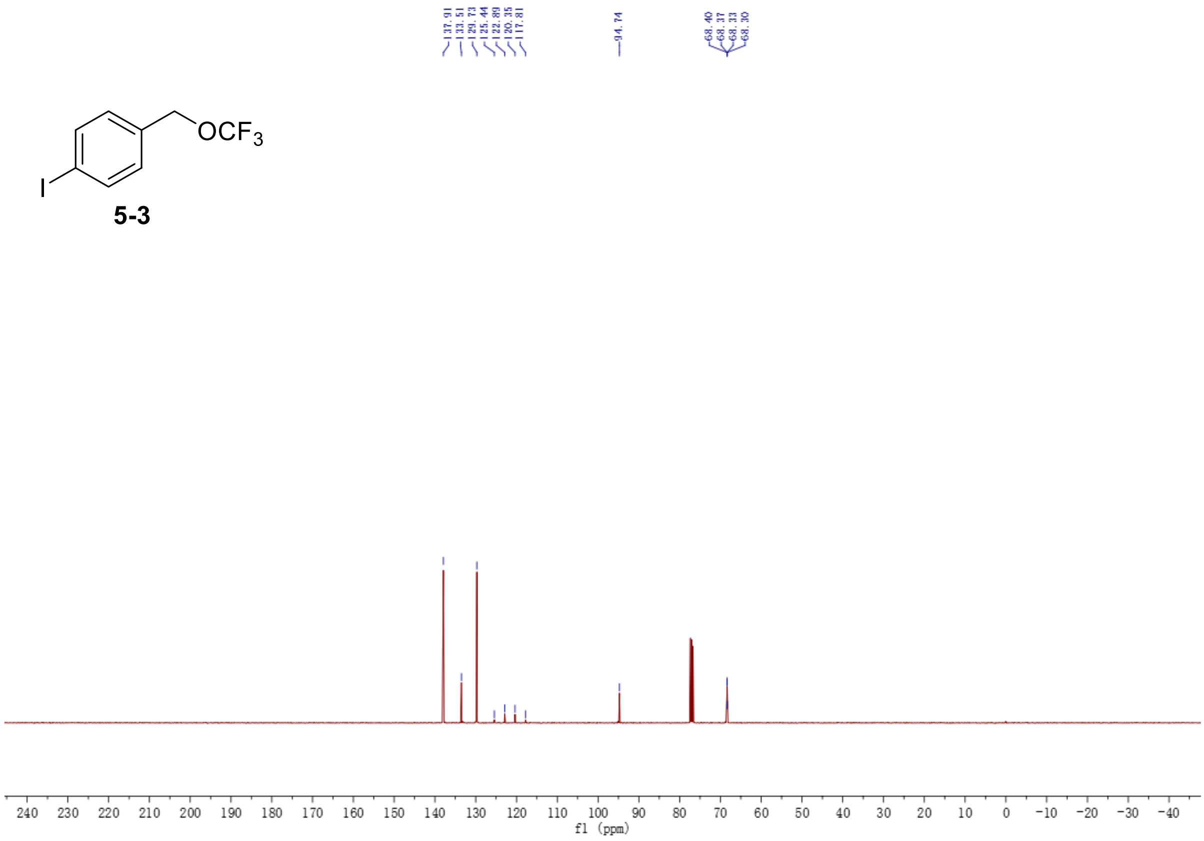

Supplementary Figure 29. ^{13}C NMR spectrum (101 MHz, $CDCl_3$) of **5-3**

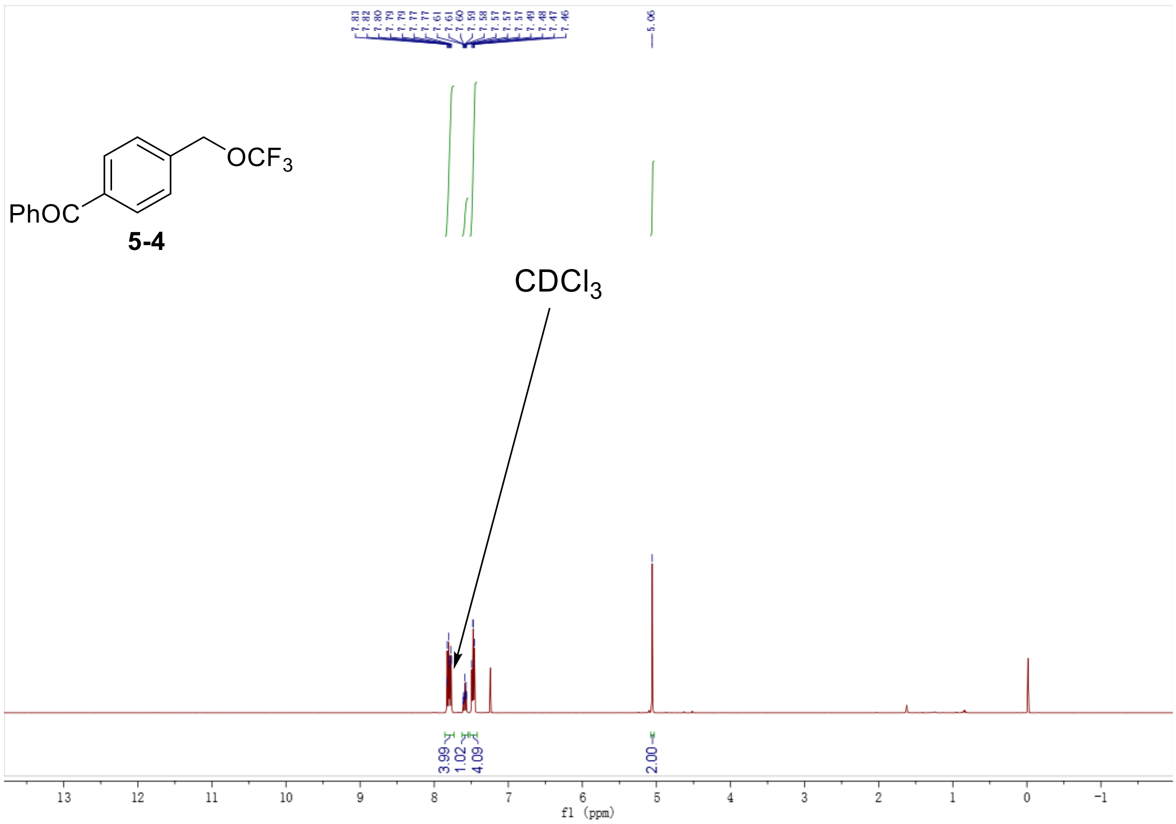

Supplementary Figure 30. ^{1}H NMR spectrum (400 MHz, $CDCl_3$) of **5-4**

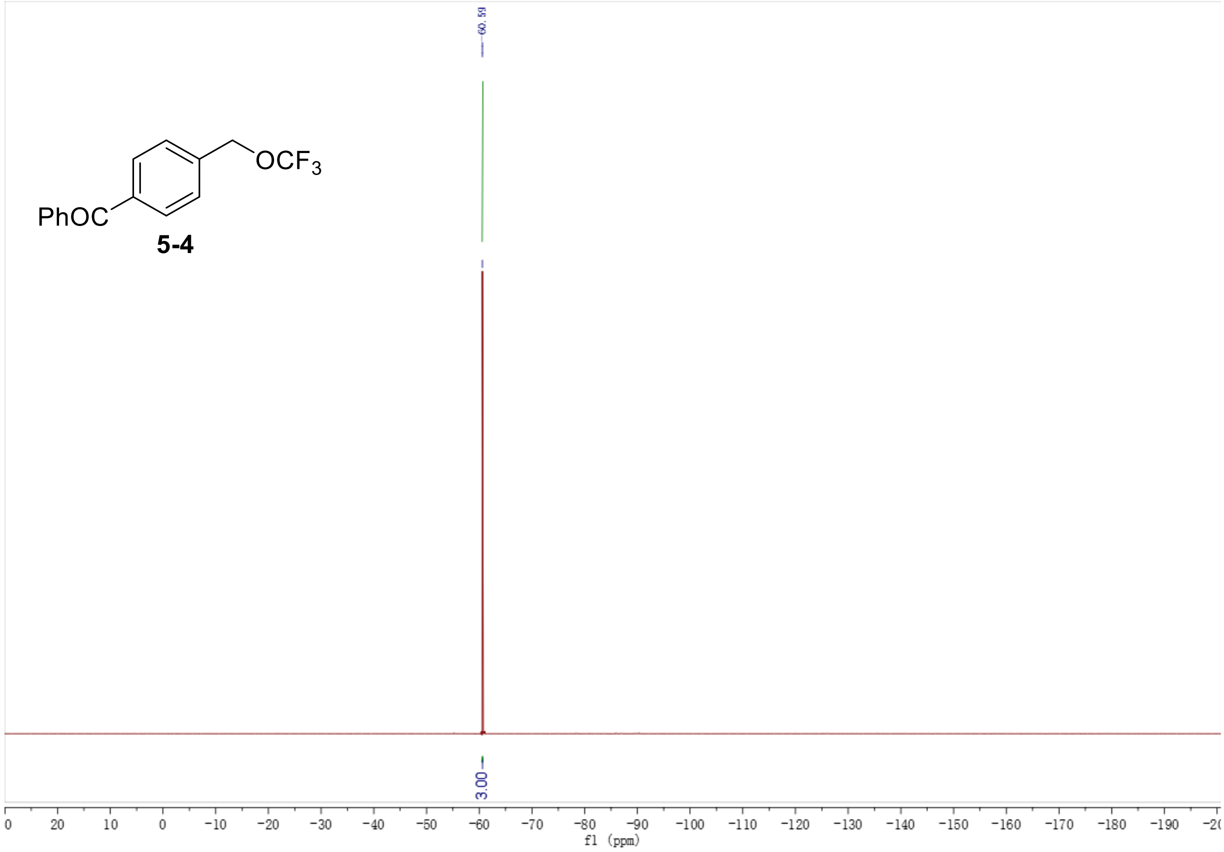

Supplementary Figure 31. ^{19}F NMR spectrum (376 MHz, $CDCl_3$) of **5-4**

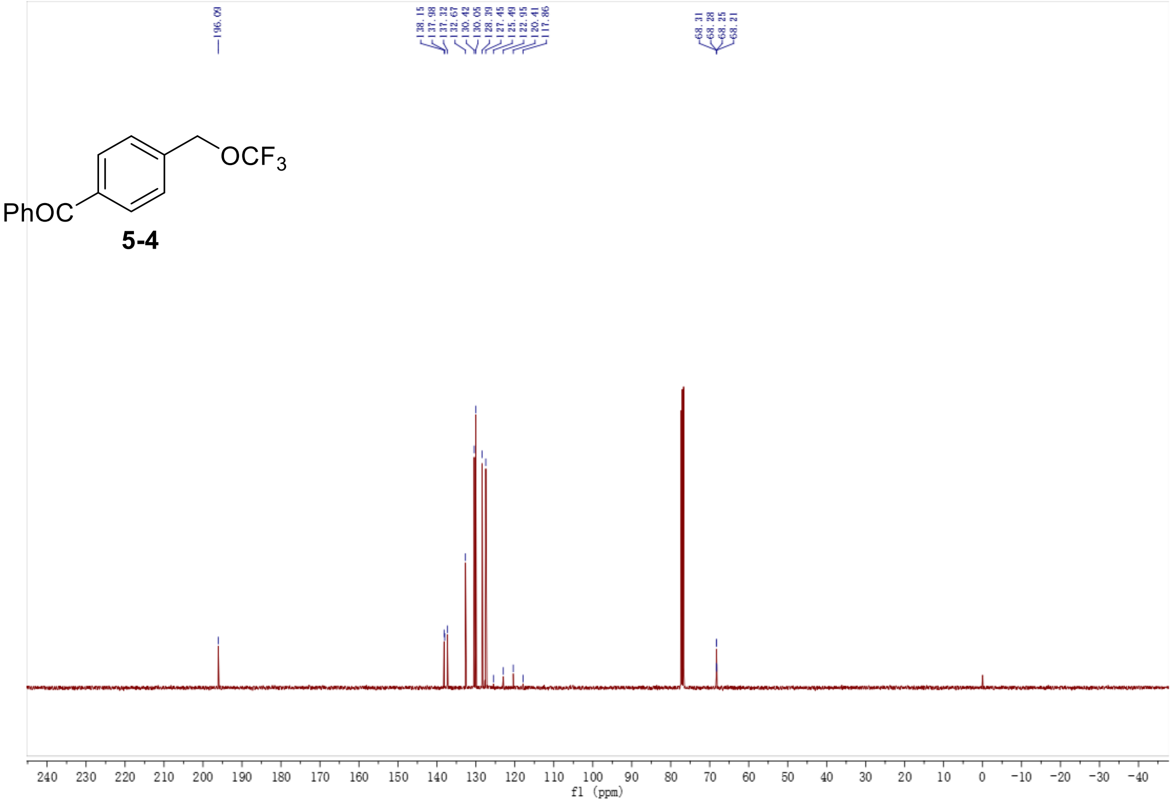

Supplementary Figure 32. ^{13}C NMR spectrum (101 MHz, $CDCl_3$) of **5-4**

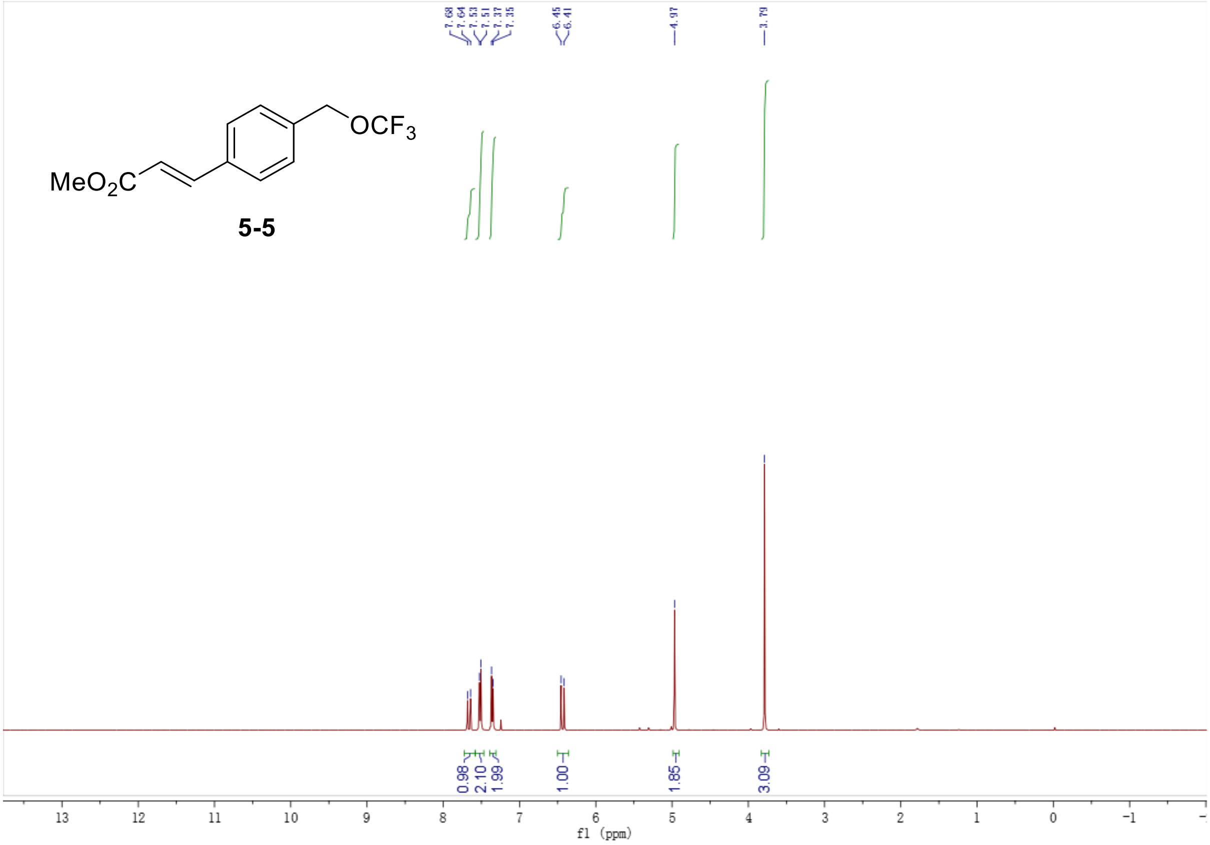

Supplementary Figure 33. ^{1}H NMR spectrum (400 MHz, $CDCl_3$) of **5-5**

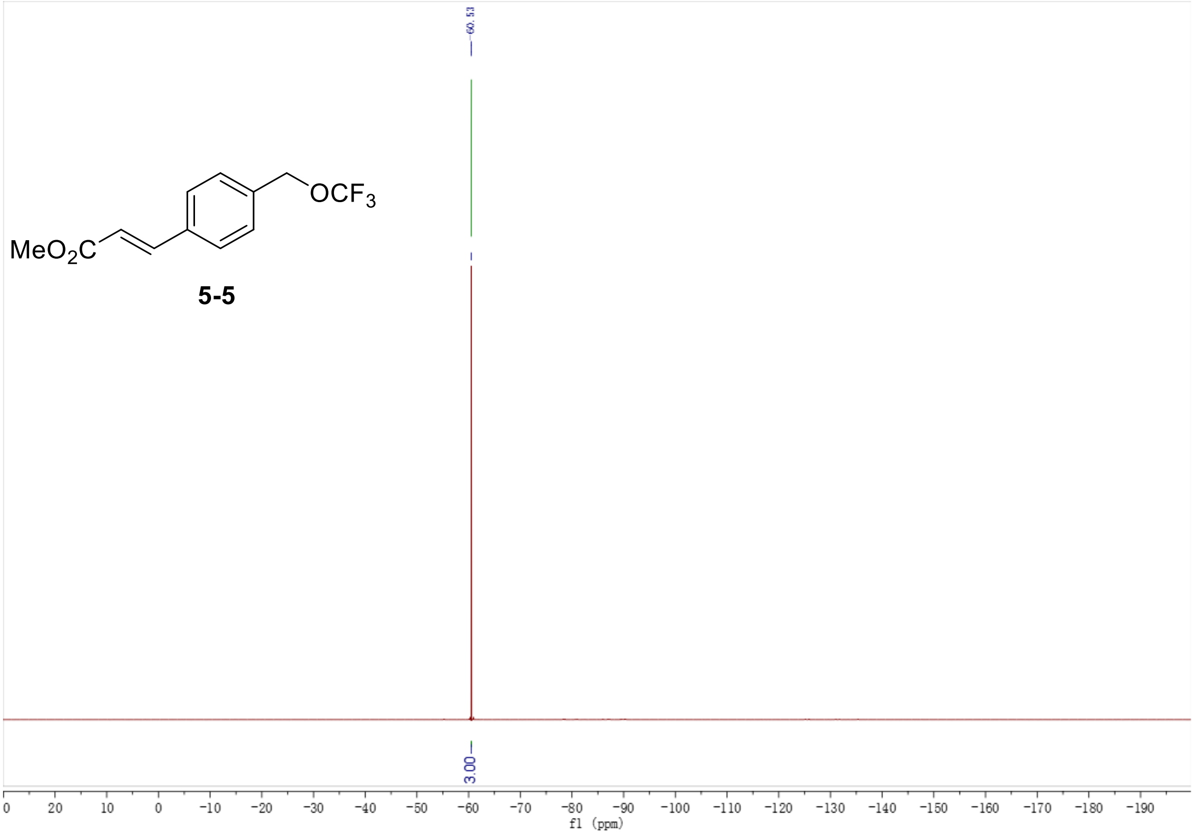

Supplementary Figure 34. ^{19}F NMR spectrum (376 MHz, $CDCl_3$) of **5-5**

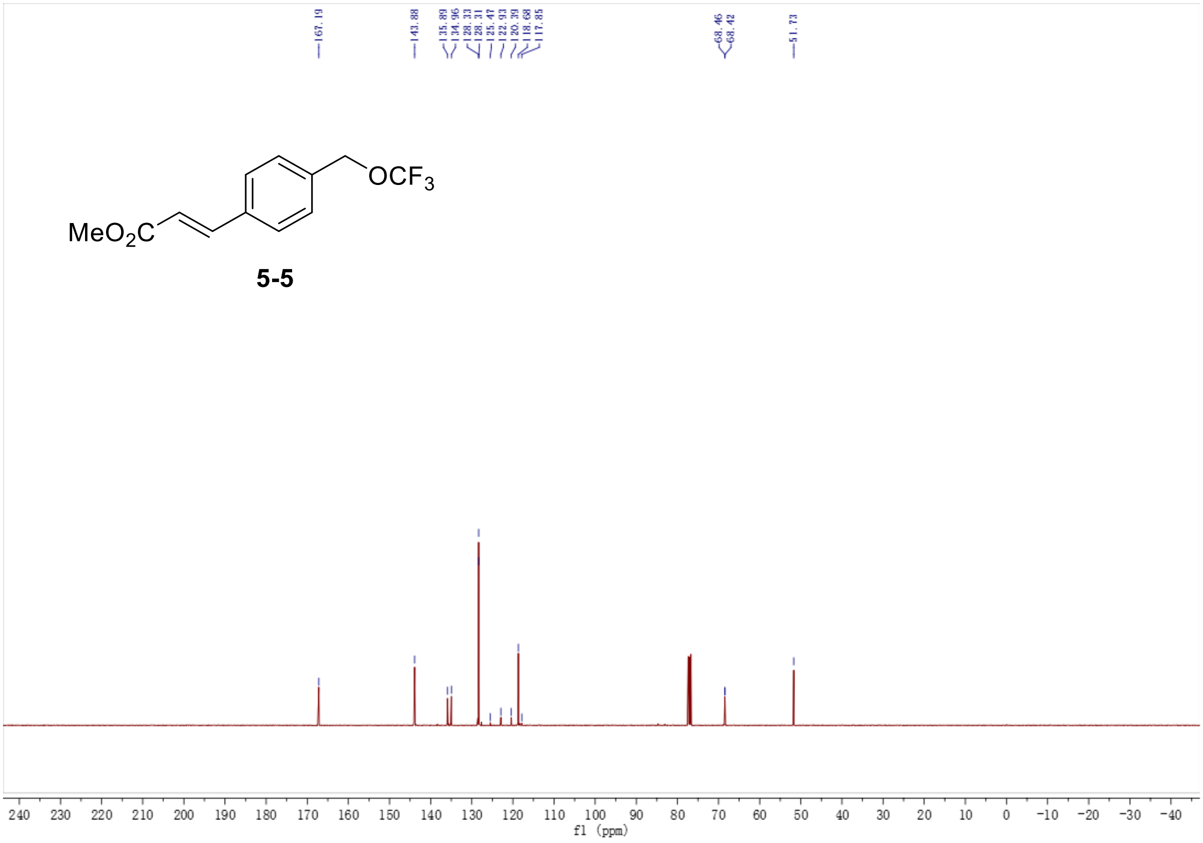

Supplementary Figure 35. ^{13}C NMR spectrum (101 MHz, $CDCl_3$) of **5-5**

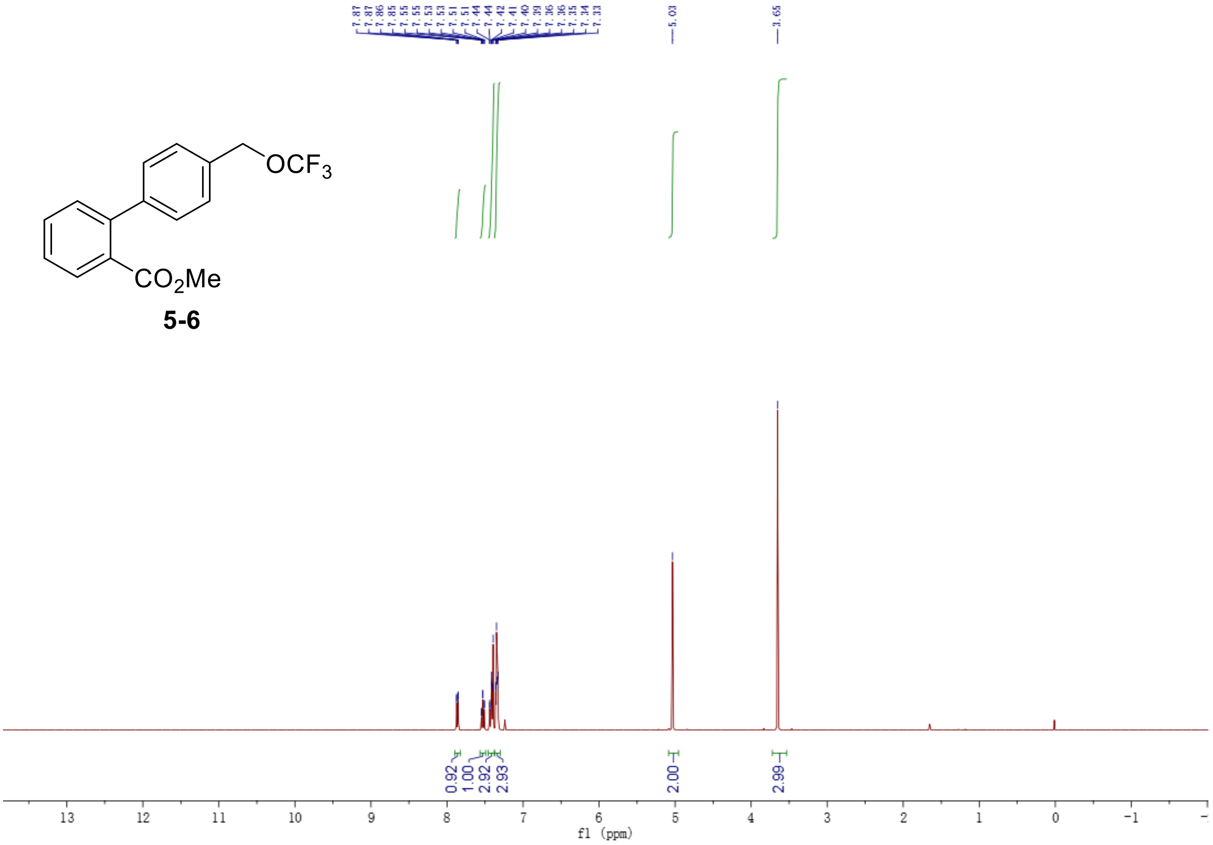

Supplementary Figure 36. ^{1}H NMR spectrum (400 MHz, $CDCl_3$) of **5-6**

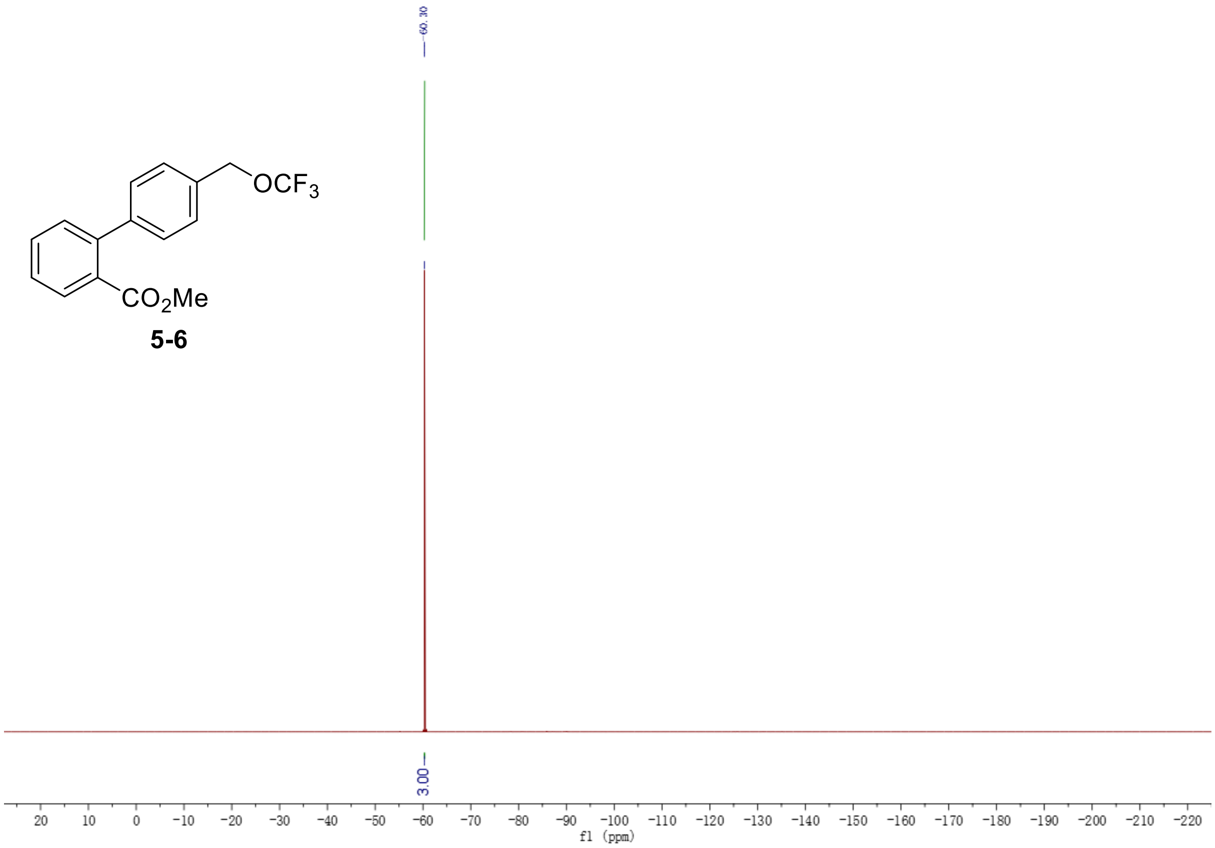

Supplementary Figure 37. ^{19}F NMR spectrum (376 MHz, $CDCl_3$) of **5-6**

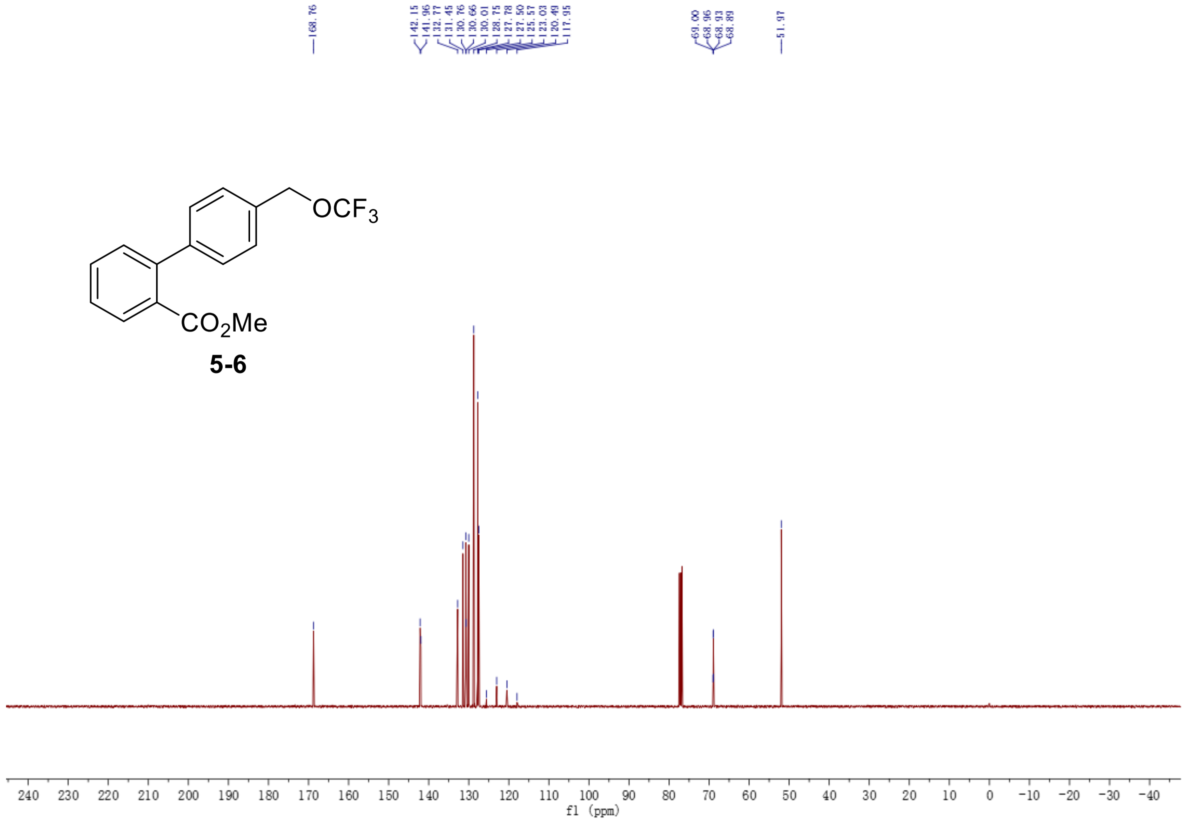

Supplementary Figure 38. ^{13}C NMR spectrum (101 MHz, $CDCl_3$) of **5-6**

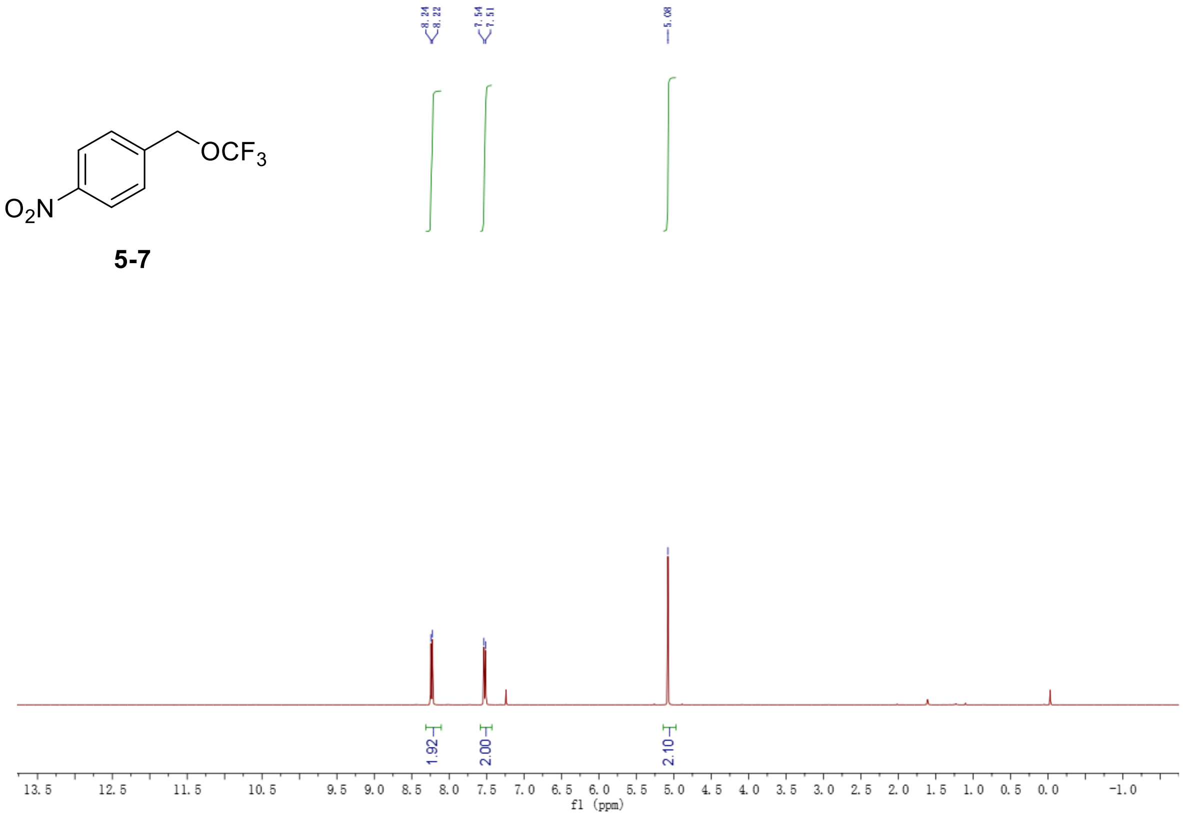

Supplementary Figure 39. ^{1}H NMR spectrum (400 MHz, $CDCl_3$) of **5-7**

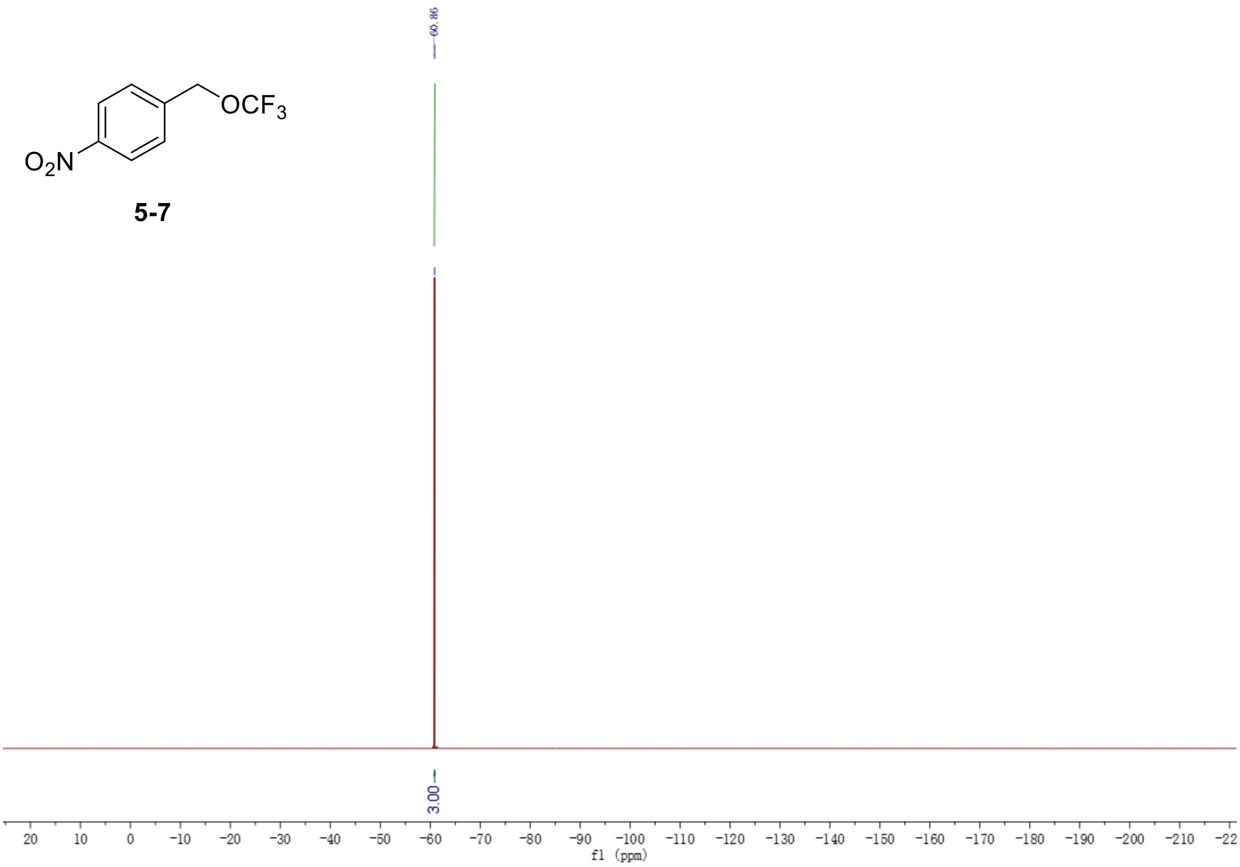

Supplementary Figure 40. ^{19}F NMR spectrum (376 MHz, $CDCl_3$) of **5-7**

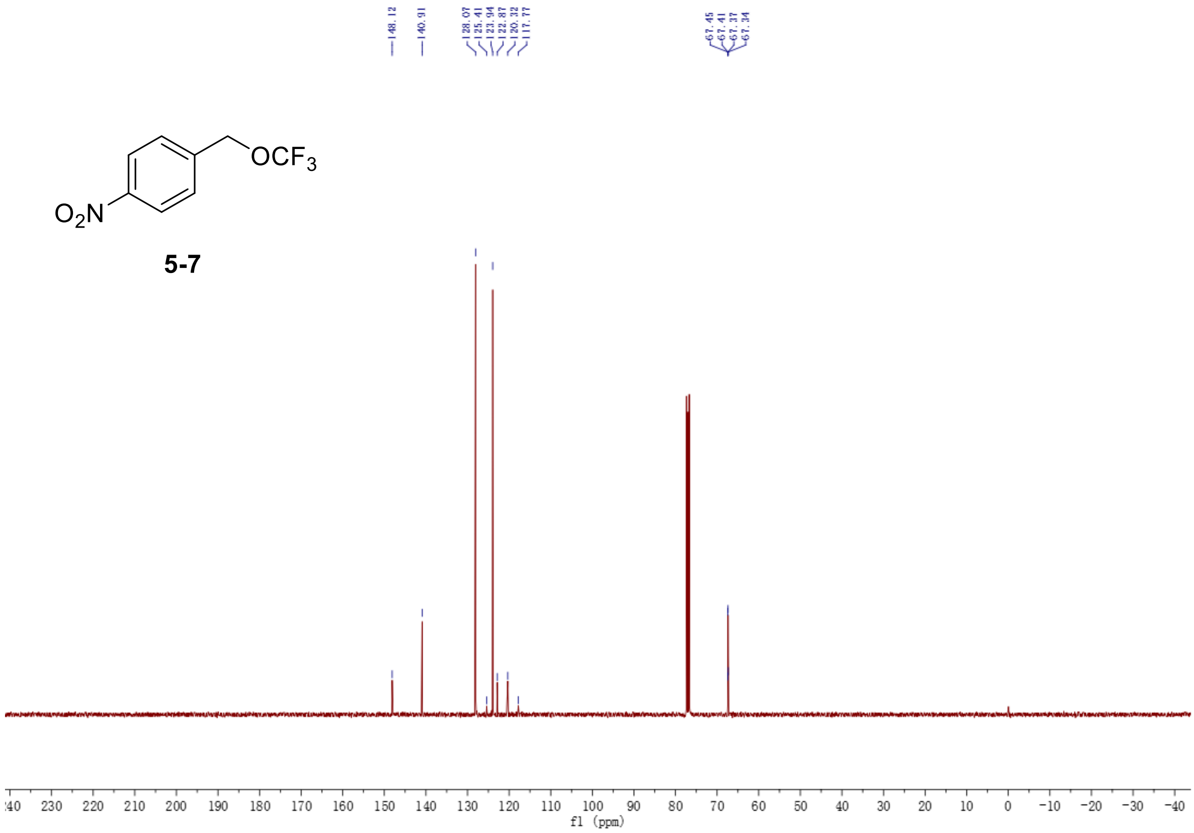

Supplementary Figure 41. ^{13}C NMR spectrum (101 MHz, $CDCl_3$) of **5-7**

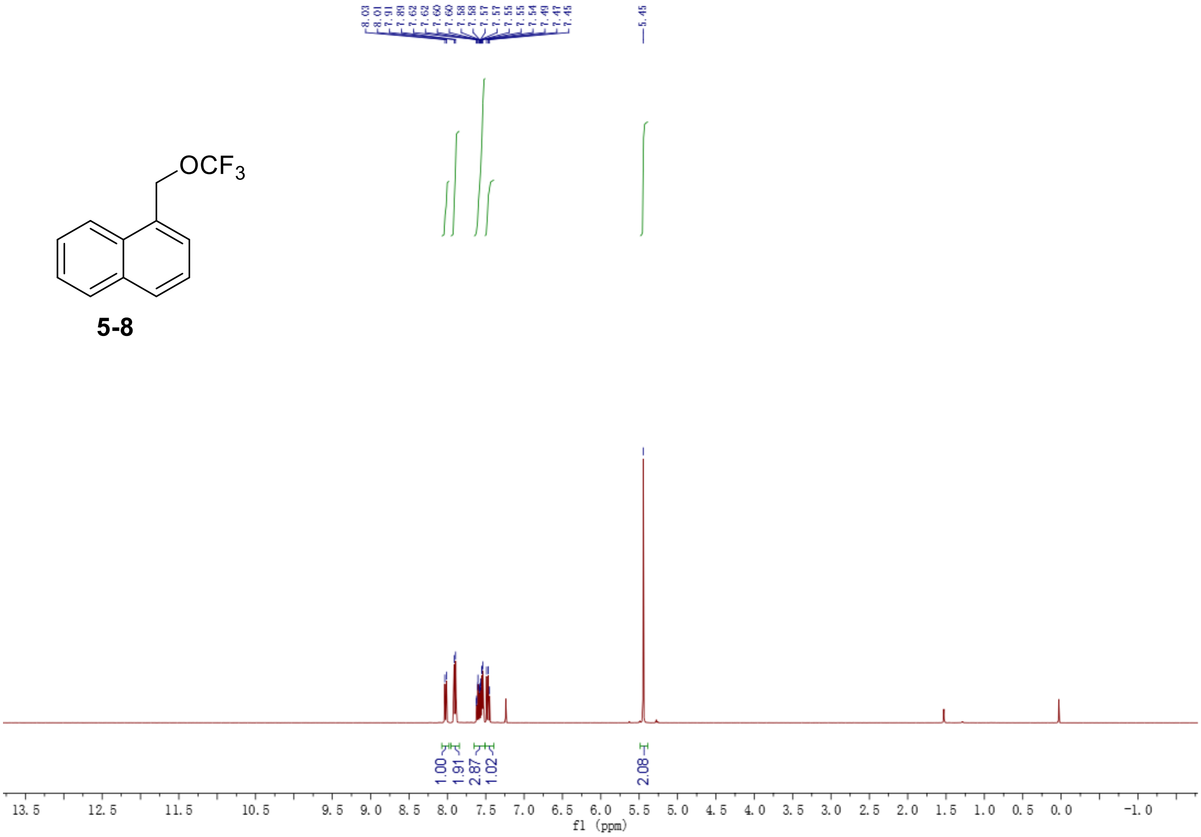

Supplementary Figure 42. ^{1}H NMR spectrum (400 MHz, $CDCl_3$) of **5-8**

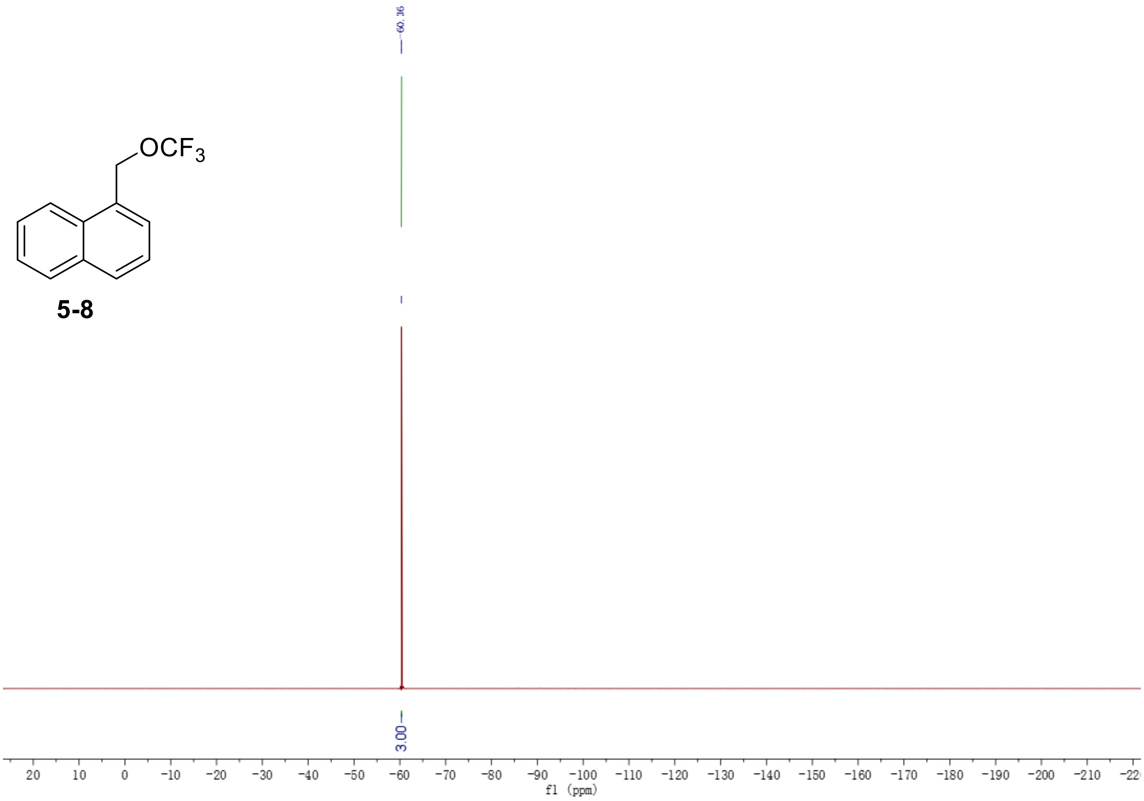

5-8

Supplementary Figure 43. ^{19}F NMR spectrum (376 MHz, $CDCl_3$) of **5-8**

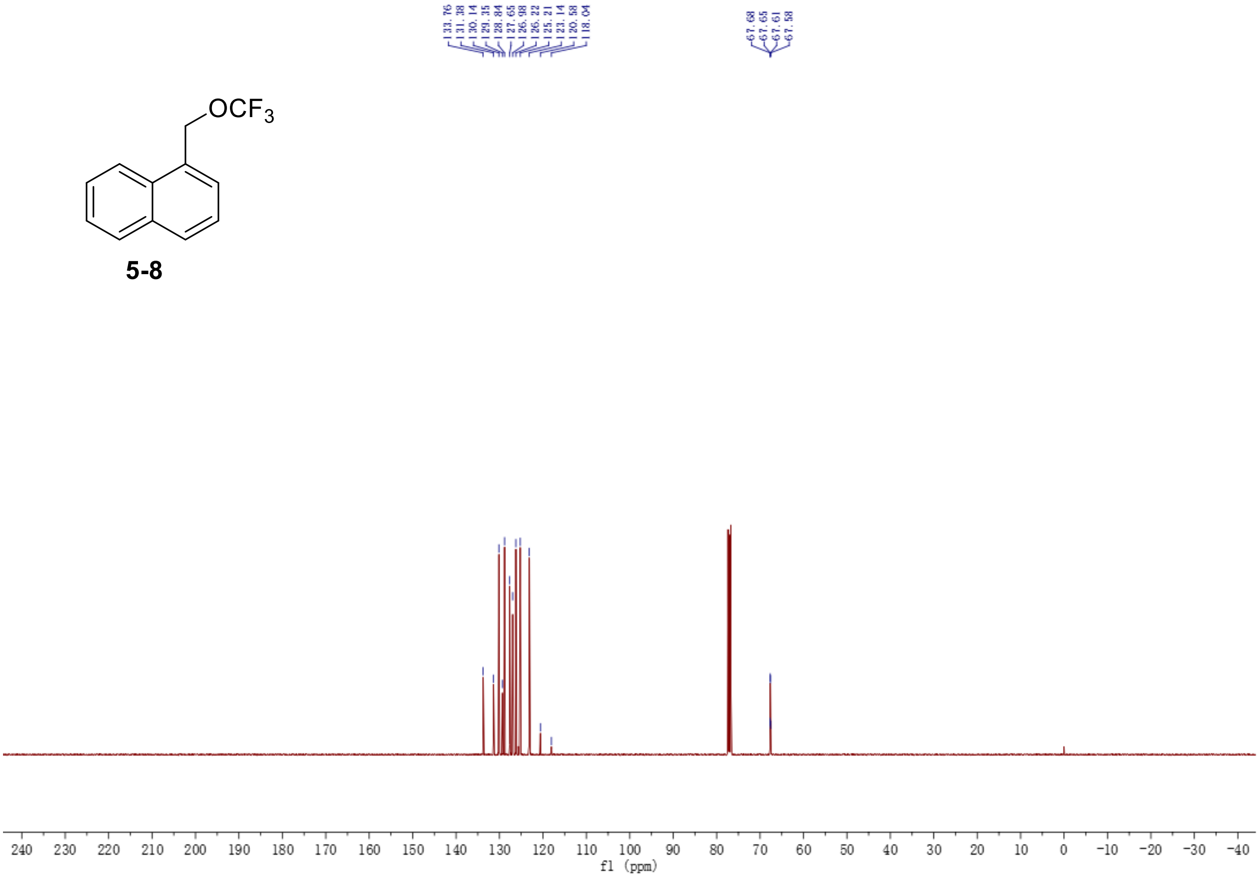

5-8

Supplementary Figure 44. ^{13}C NMR spectrum (101 MHz, $CDCl_3$) of **5-8**

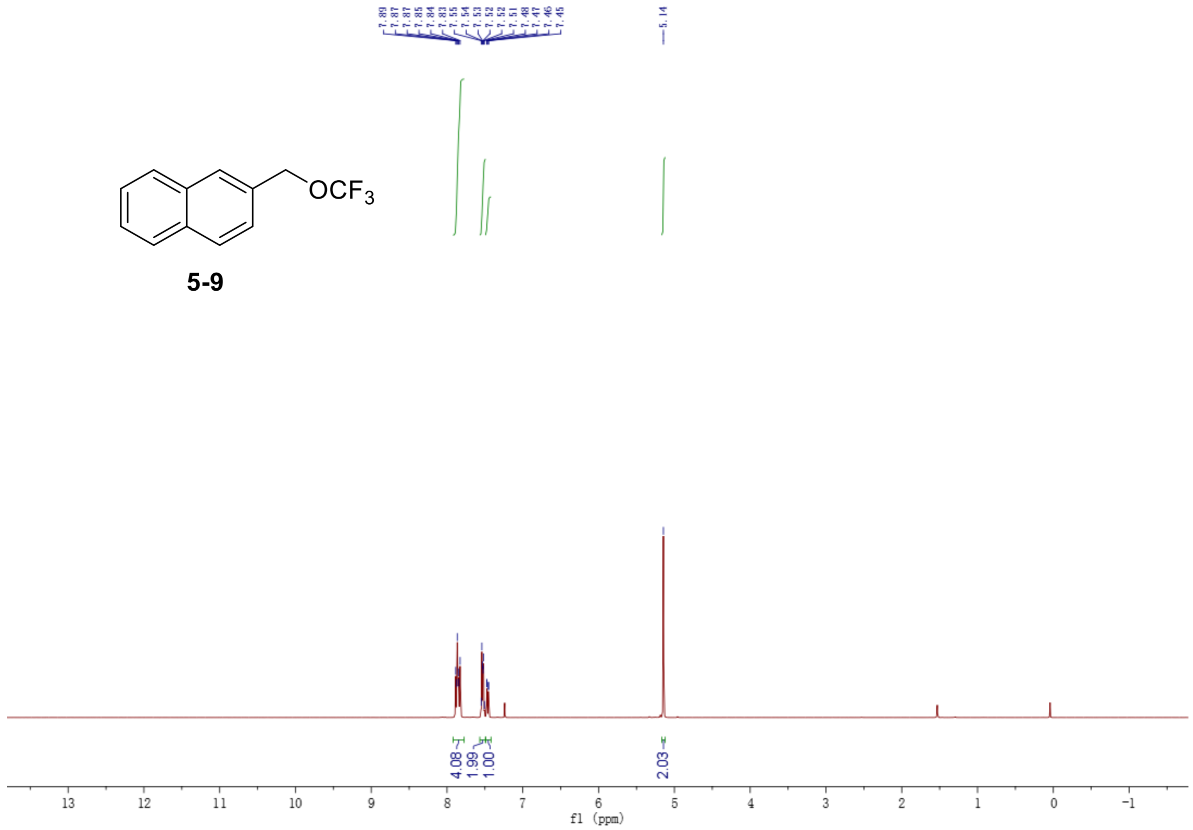

Supplementary Figure 45. ^{1}H NMR spectrum (400 MHz, $CDCl_3$) of **5-9**

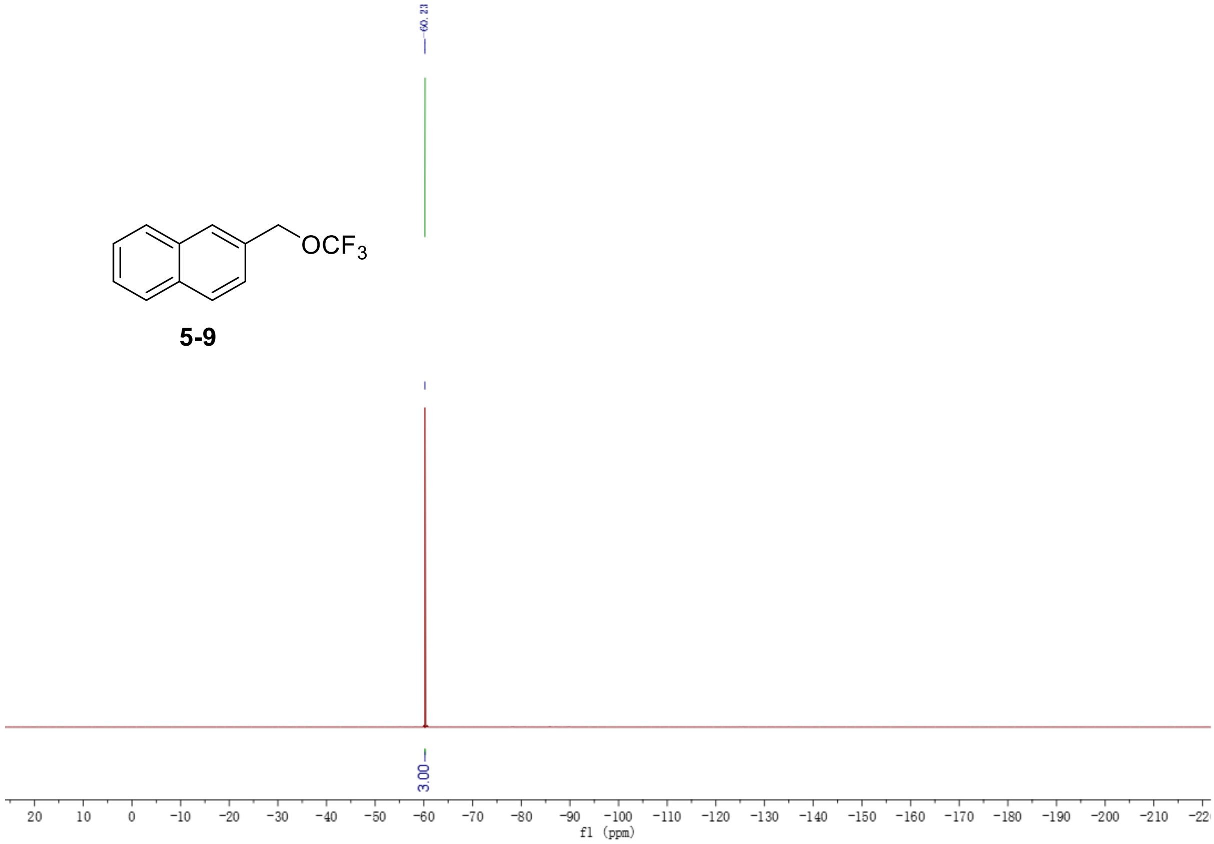

Supplementary Figure 46. ^{19}F NMR spectrum (376 MHz, $CDCl_3$) of **5-9**

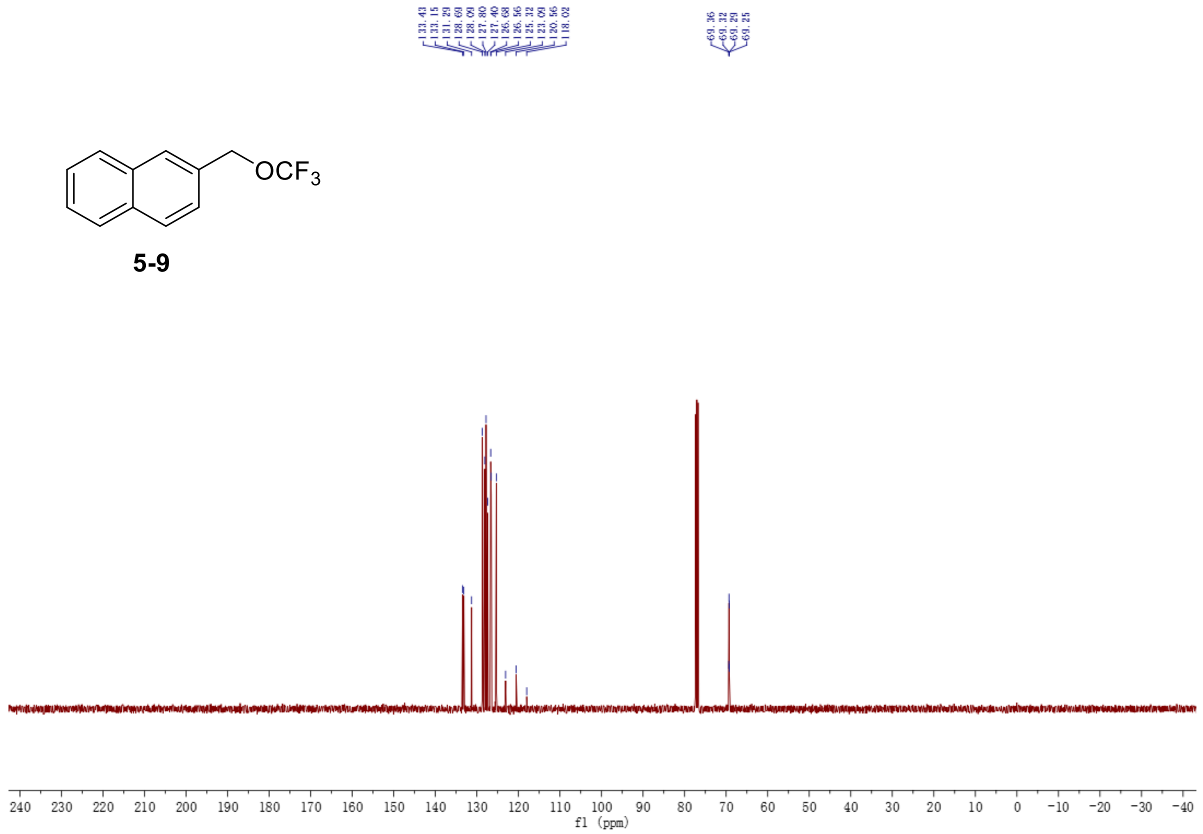

Supplementary Figure 47. ^{13}C NMR spectrum (101 MHz, $CDCl_3$) of **5-9**

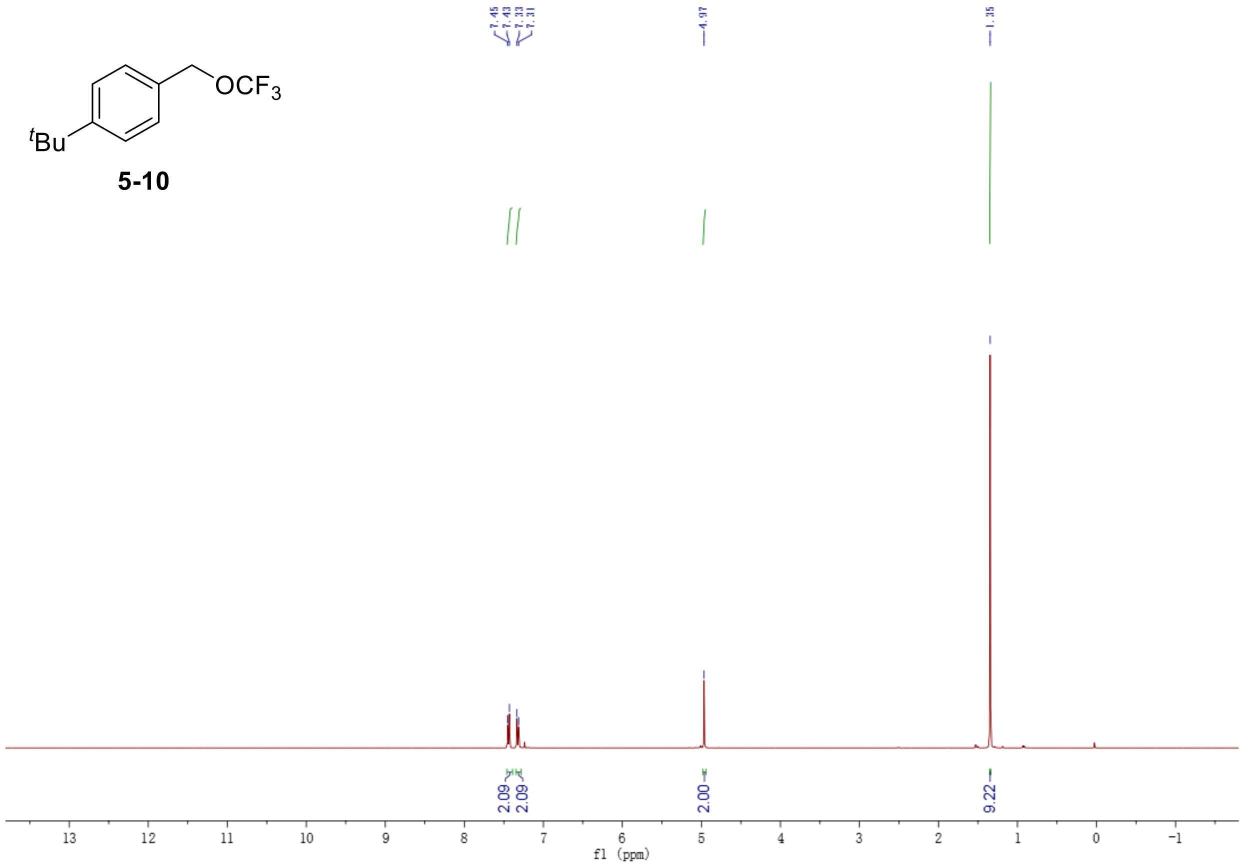

Supplementary Figure 48. ^{1}H NMR spectrum (400 MHz, $CDCl_3$) of **5-10**

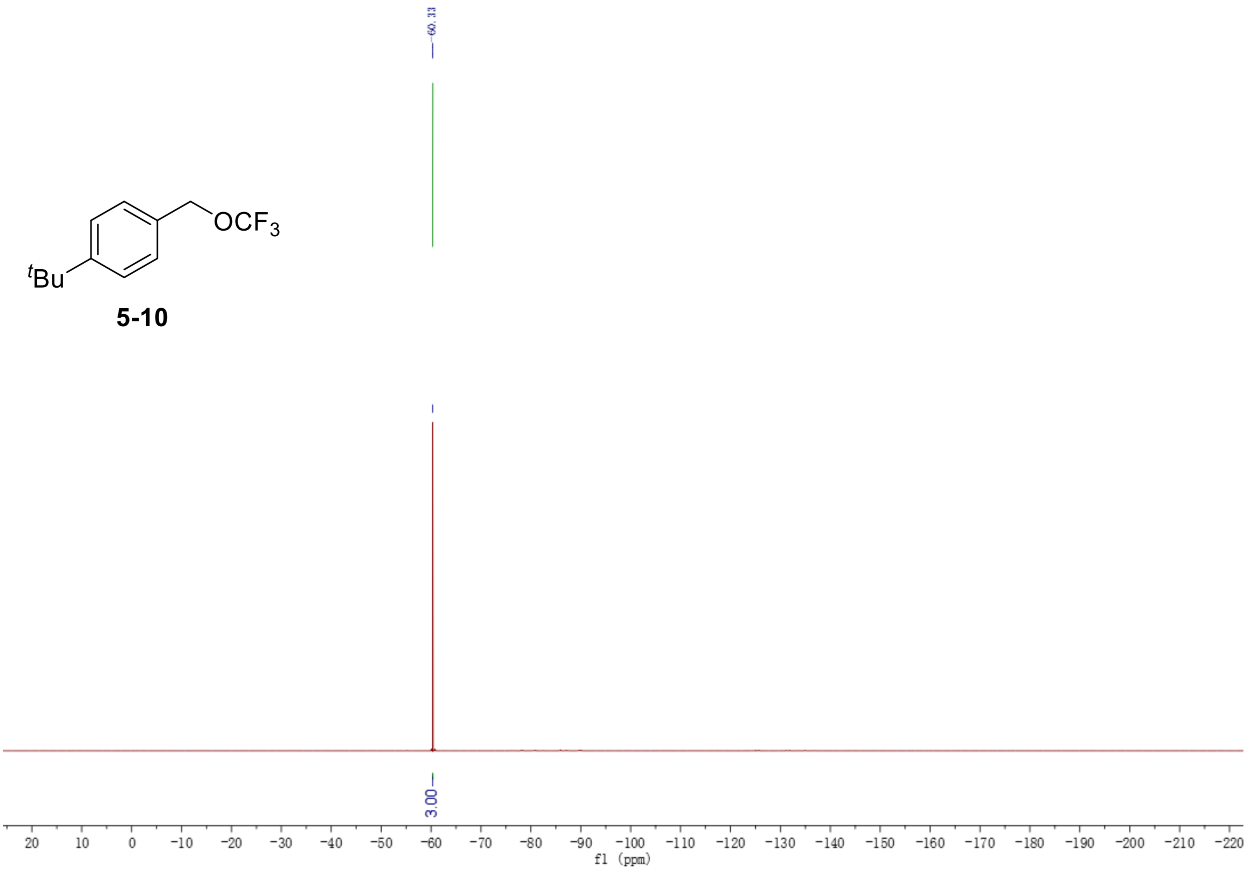

Supplementary Figure 49. ^{19}F NMR spectrum (376 MHz, $CDCl_3$) of **5-10**

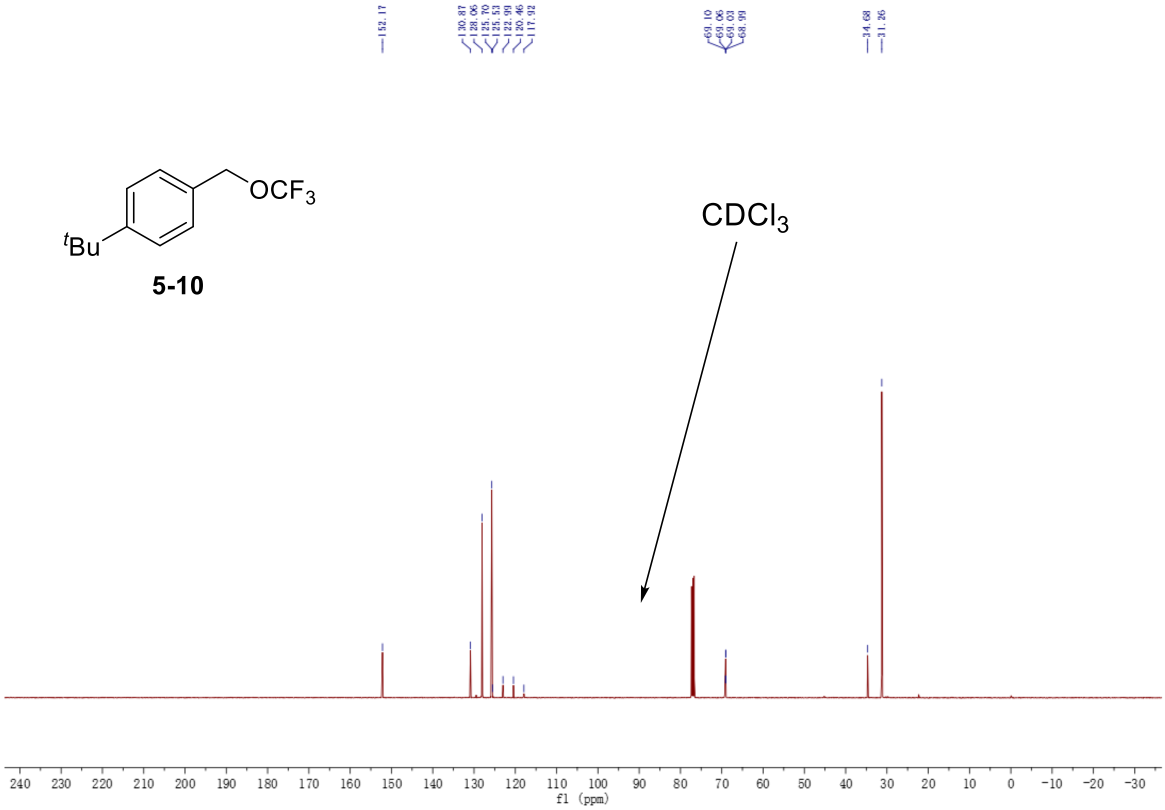

Supplementary Figure 50. ^{13}C NMR spectrum (101 MHz, $CDCl_3$) of **5-10**

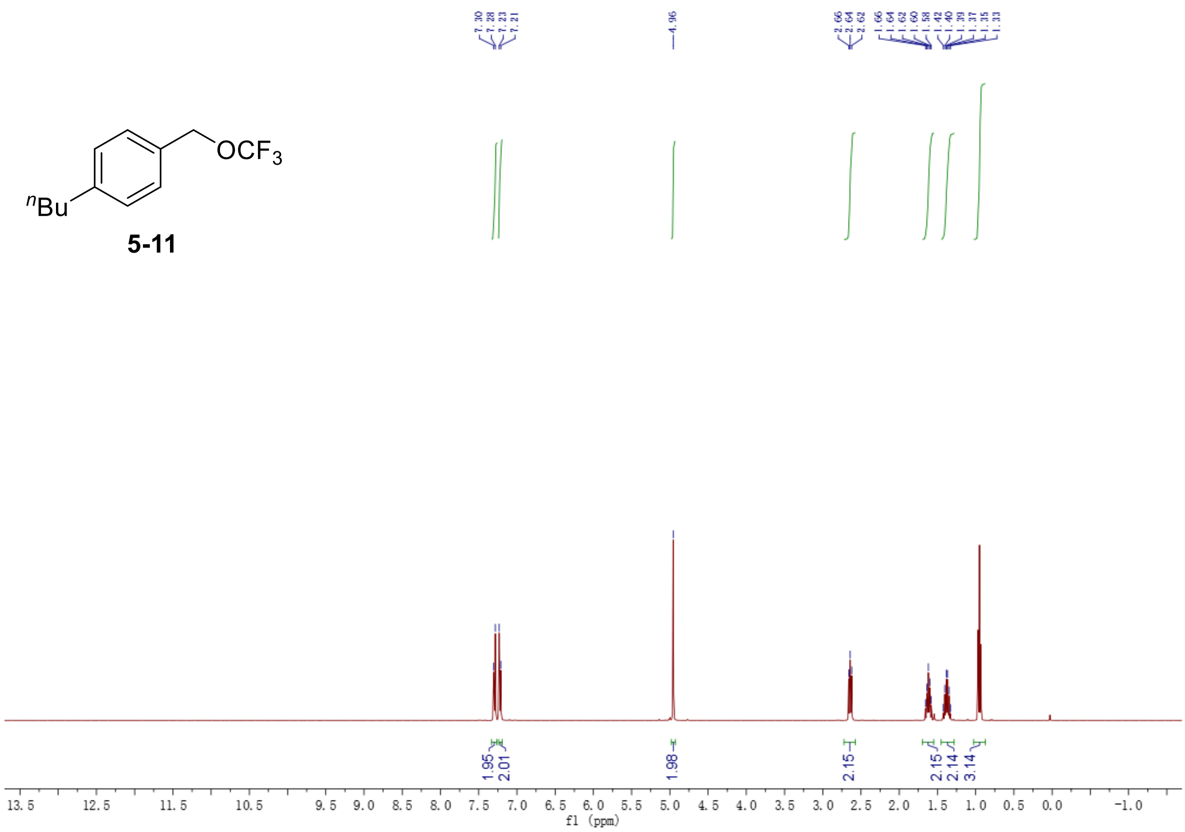

Supplementary Figure 51. ^{1}H NMR spectrum (400 MHz, $CDCl_3$) of **5-11**

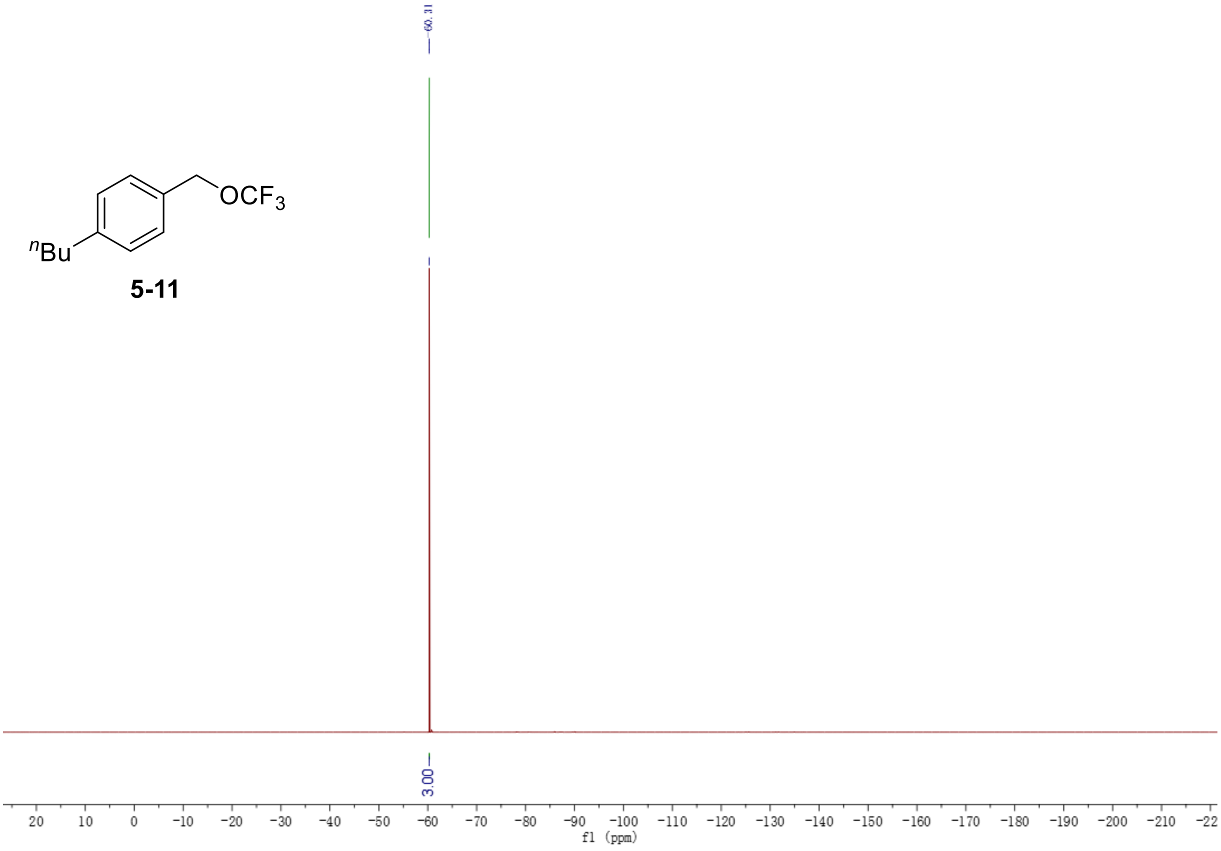

Supplementary Figure 52. ^{19}F NMR spectrum (376 MHz, $CDCl_3$) of **5-11**

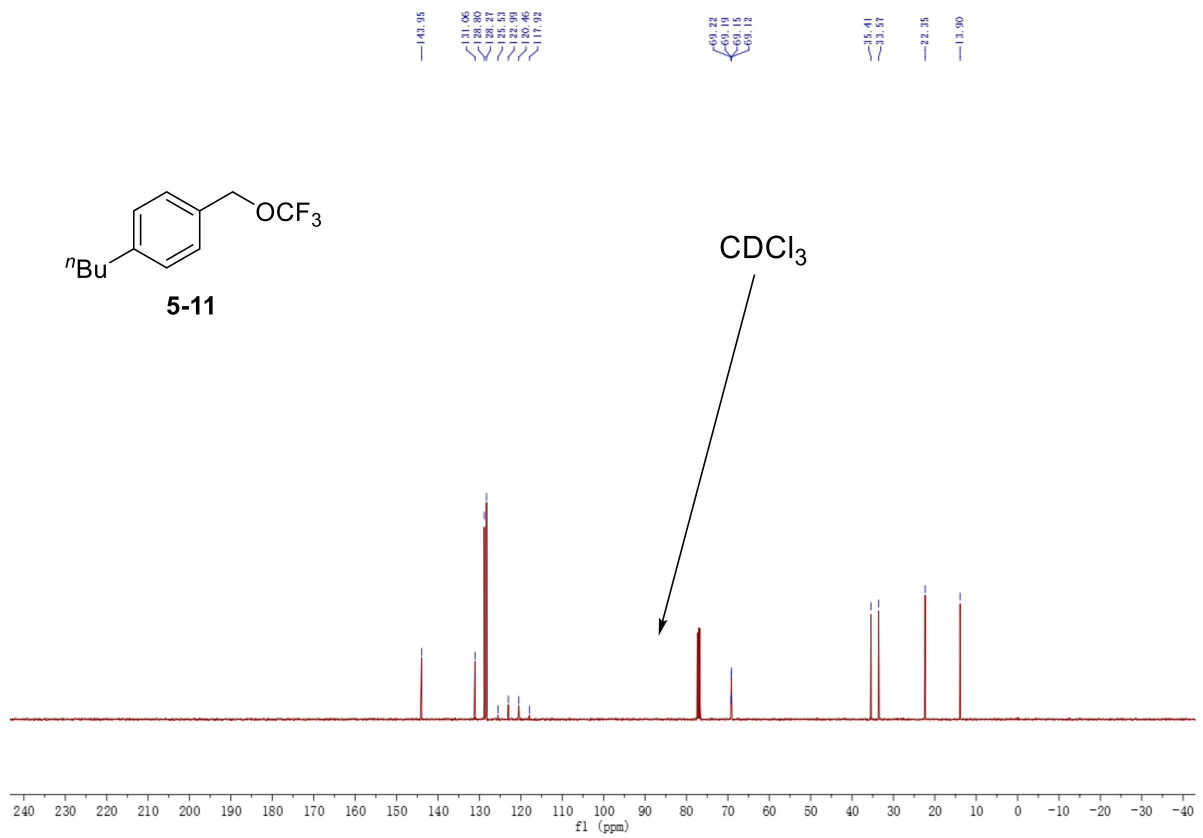

Supplementary Figure 53. ^{13}C NMR spectrum (101 MHz, $CDCl_3$) of **5-11**

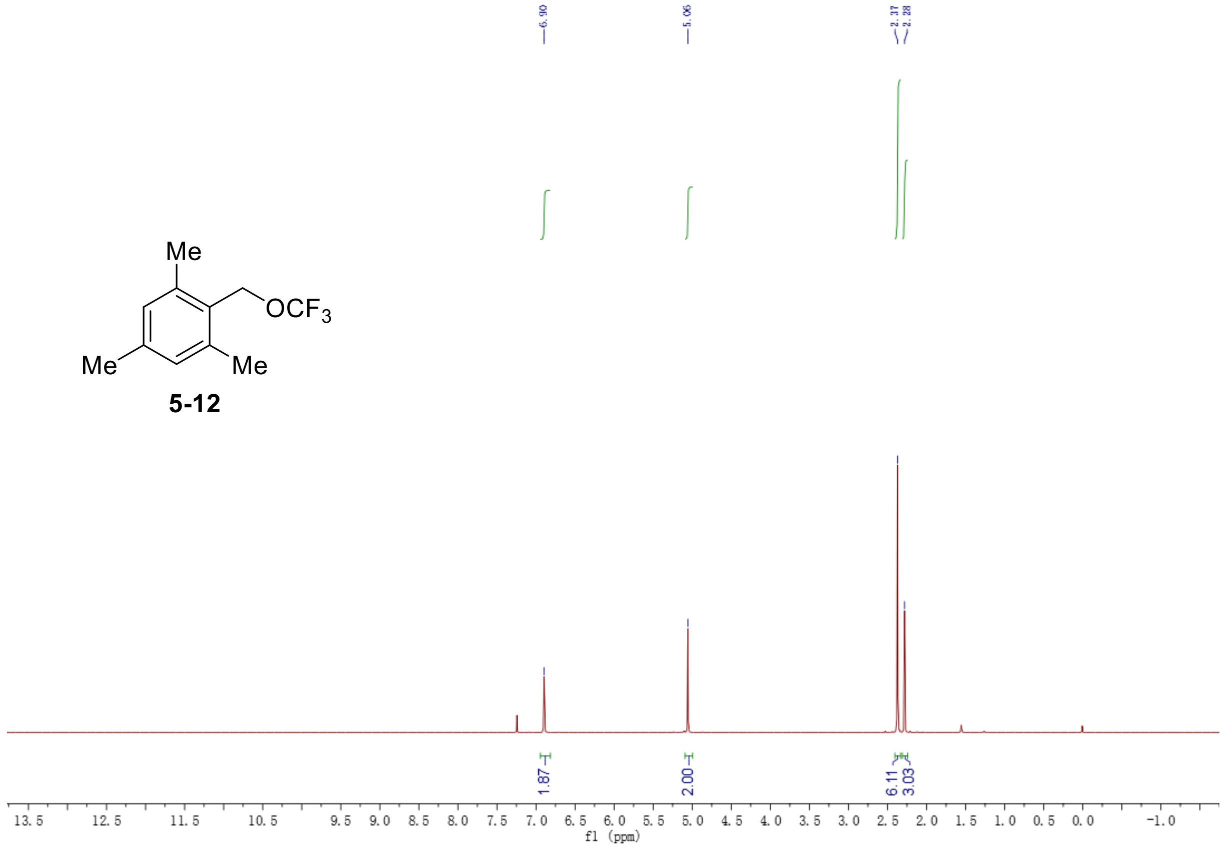

Supplementary Figure 54. ^{1}H NMR spectrum (400 MHz, $CDCl_3$) of **5-12**

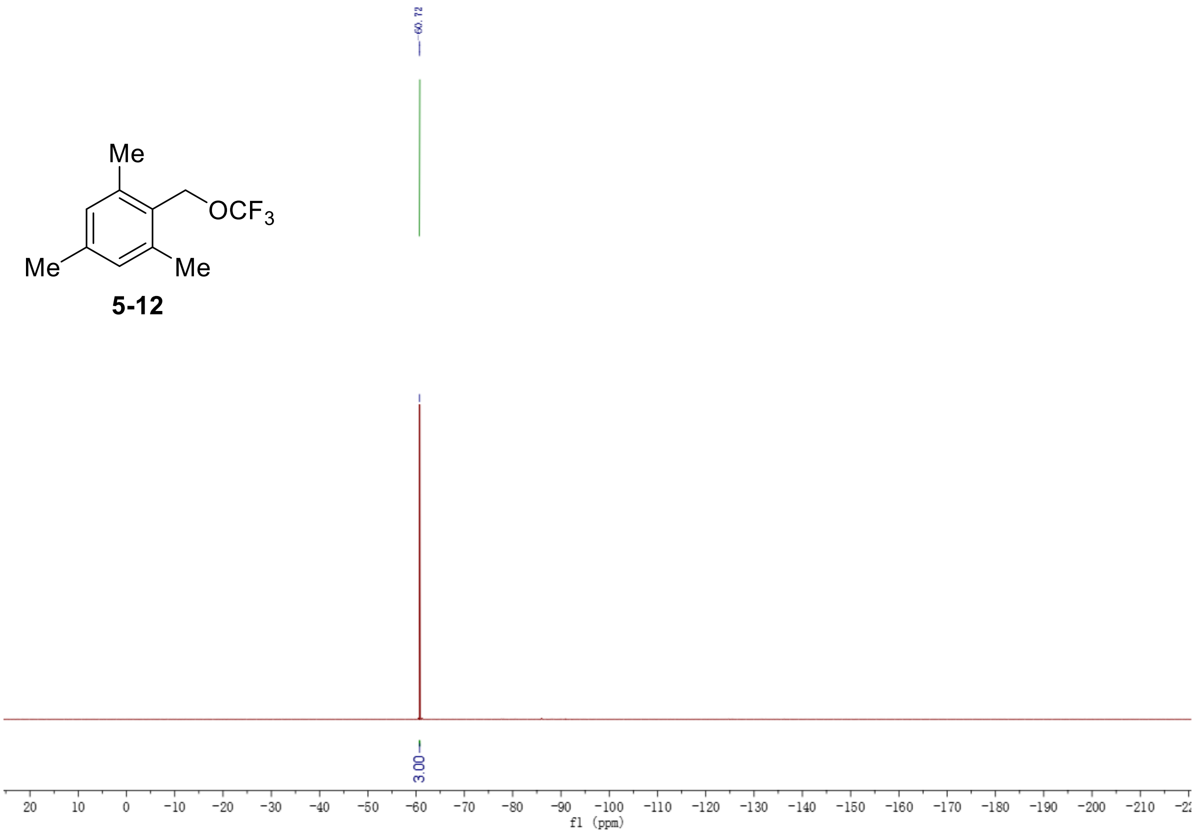

Supplementary Figure 55. ^{19}F NMR spectrum (376 MHz, $CDCl_3$) of **5-12**

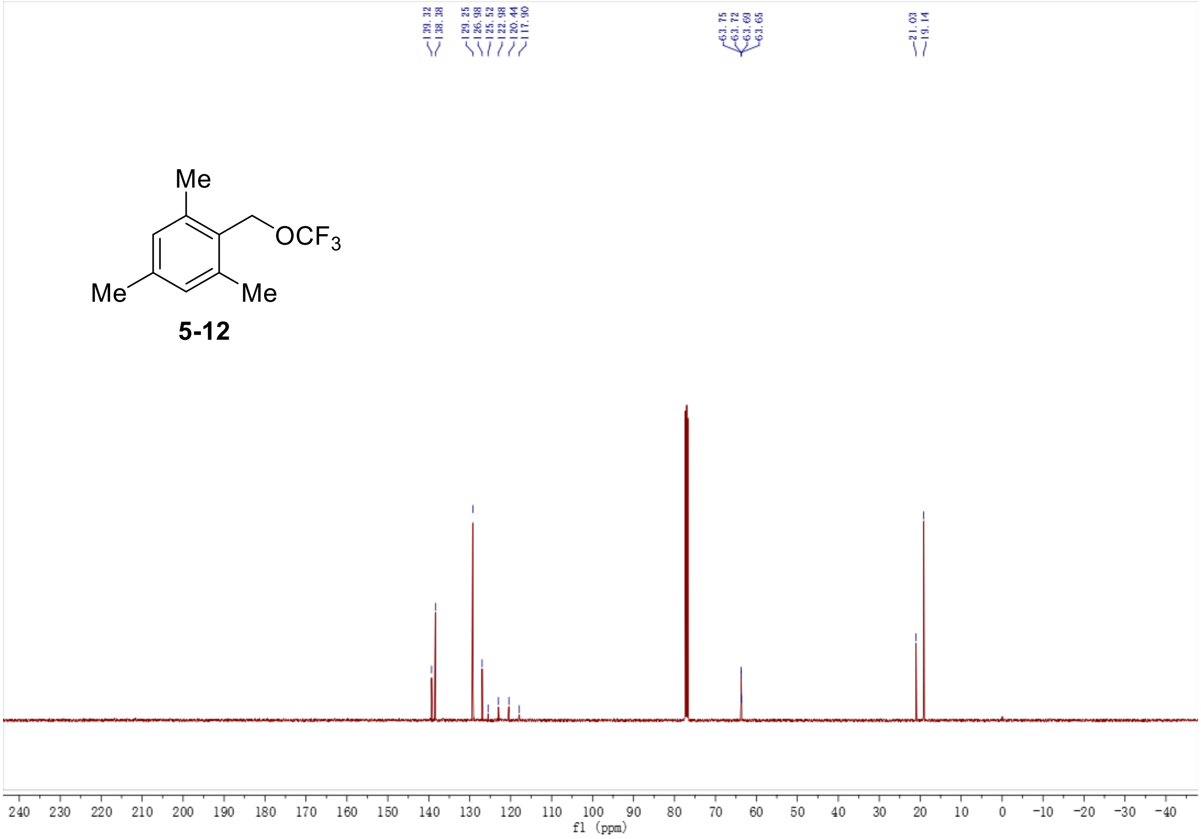

Supplementary Figure 56. ^{13}C NMR spectrum (101 MHz, $CDCl_3$) of **5-12**

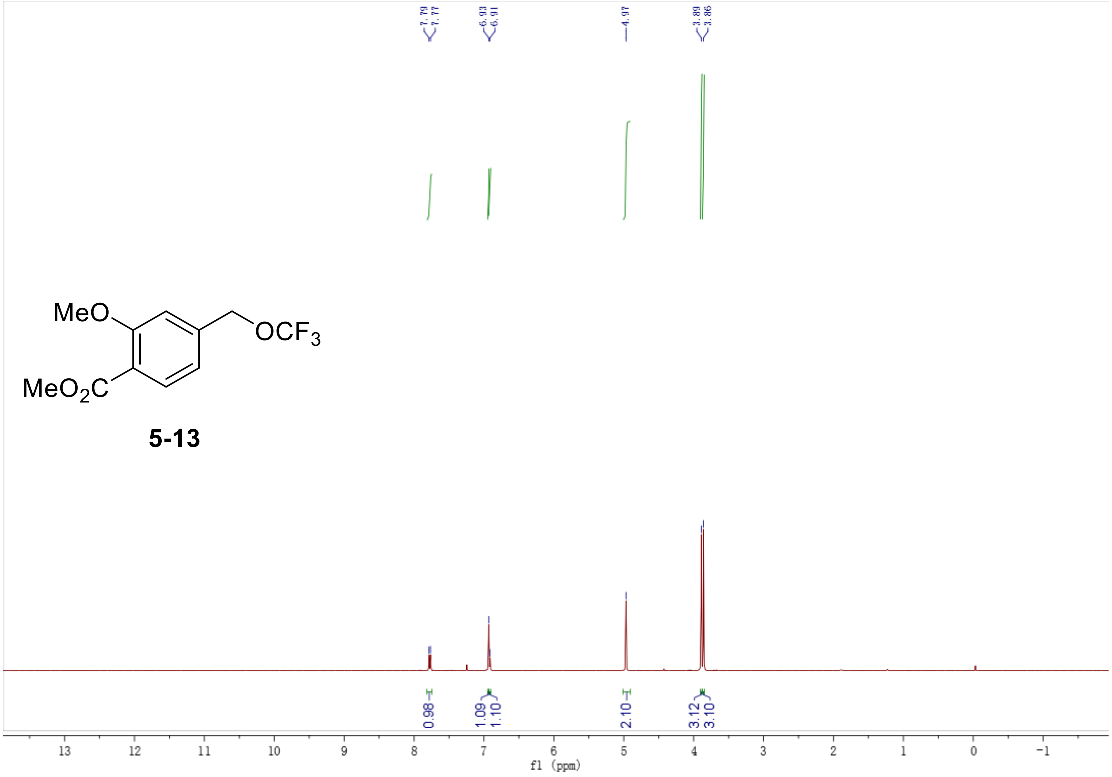

Supplementary Figure 57. ^{1}H NMR spectrum (400 MHz, $CDCl_3$) of **5-13**

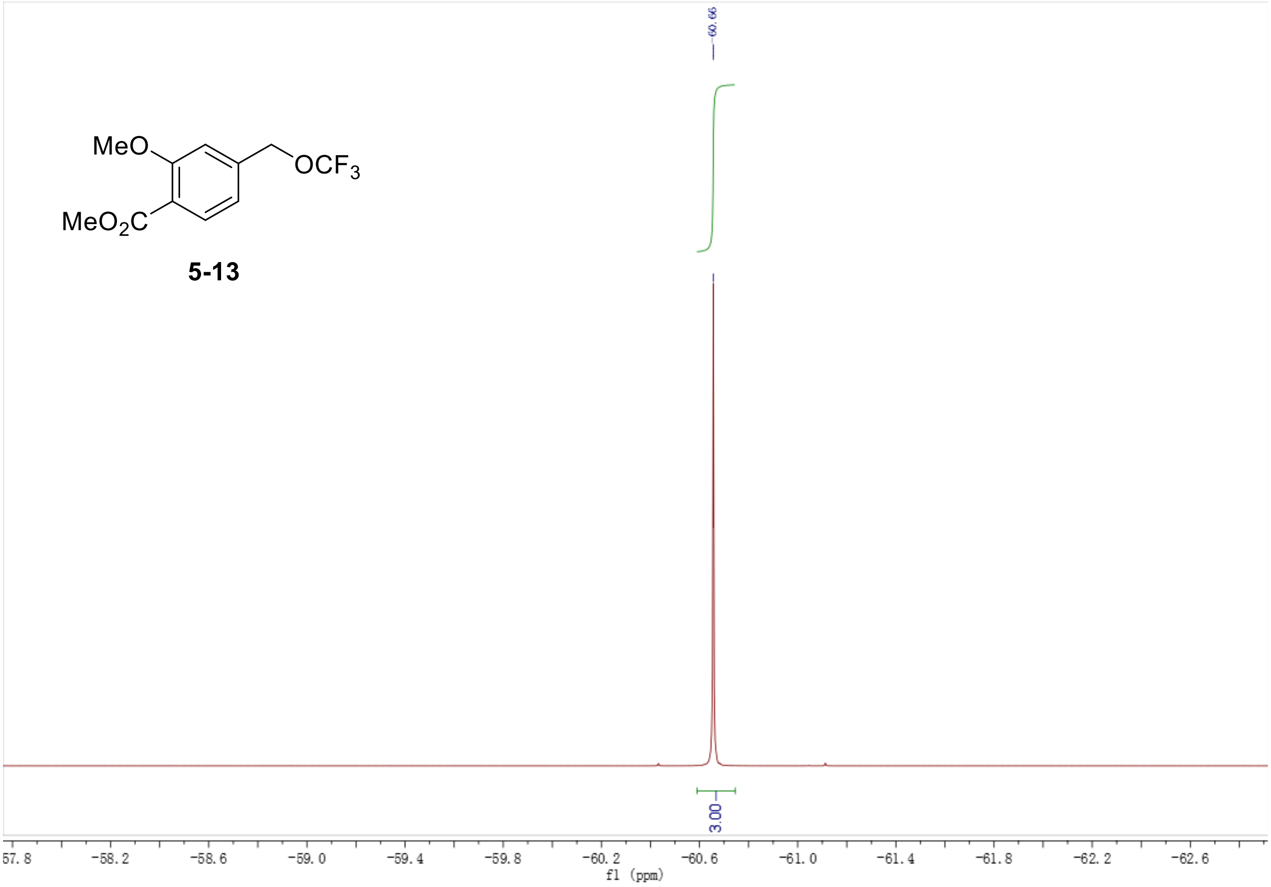

Supplementary Figure 58. ^{19}F NMR spectrum (376 MHz, $CDCl_3$) of **5-13**

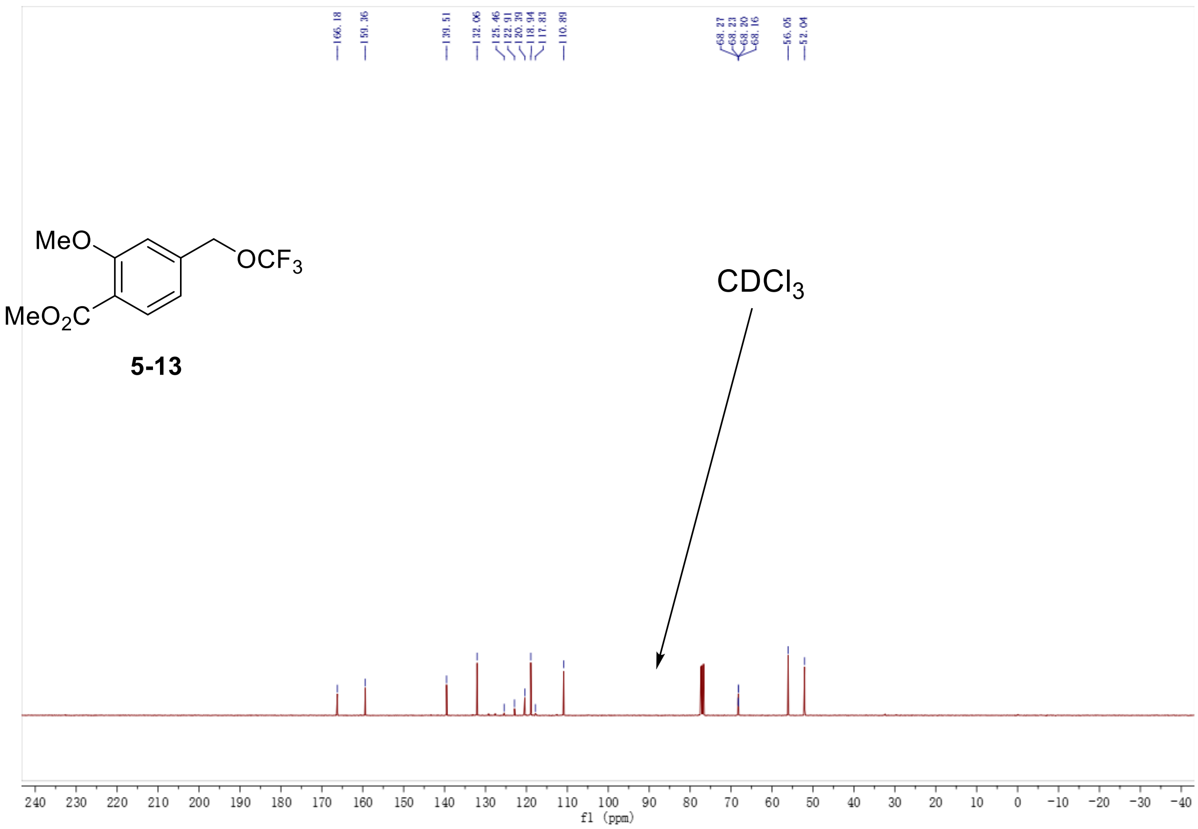

Supplementary Figure 59. ^{13}C NMR spectrum (101 MHz, CDCl$_3$) of **5-13**

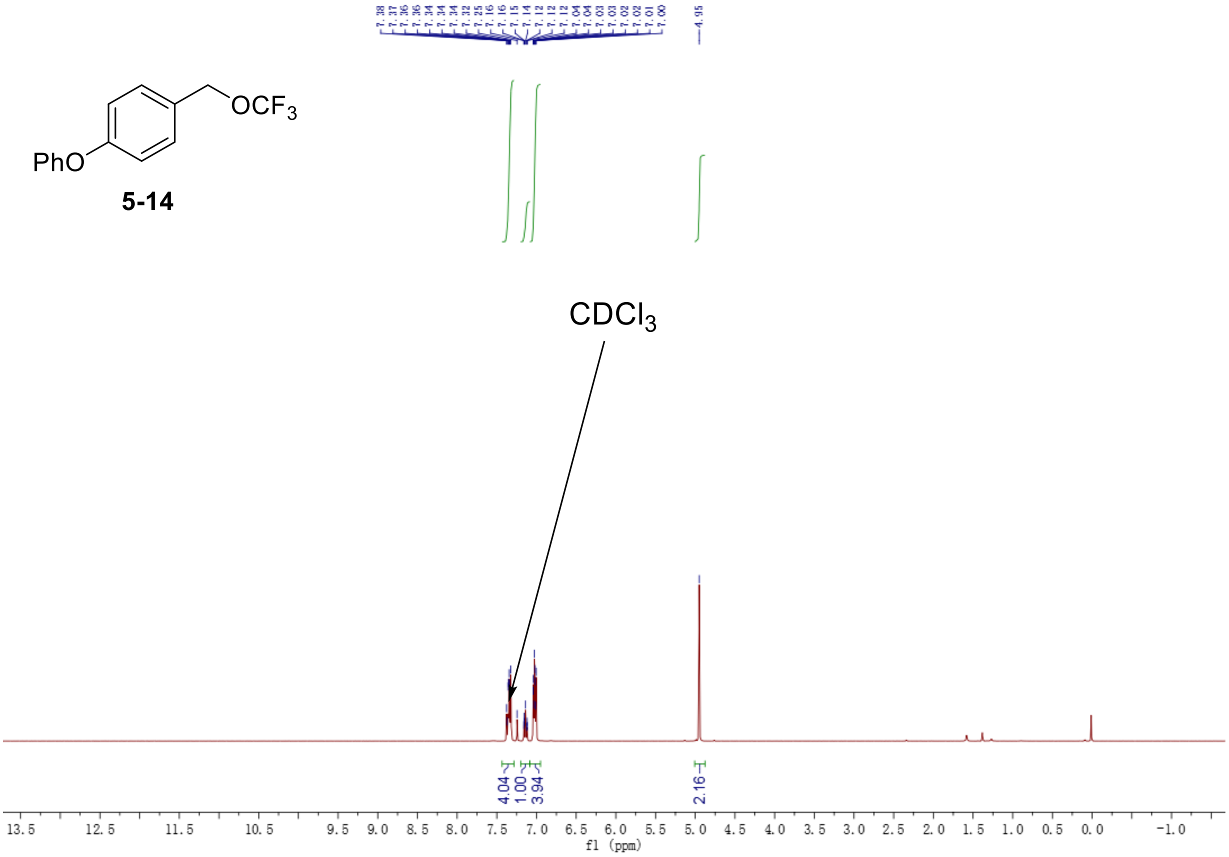

Supplementary Figure 60. ^{1}H NMR spectrum (400 MHz, CDCl$_3$) of **5-14**

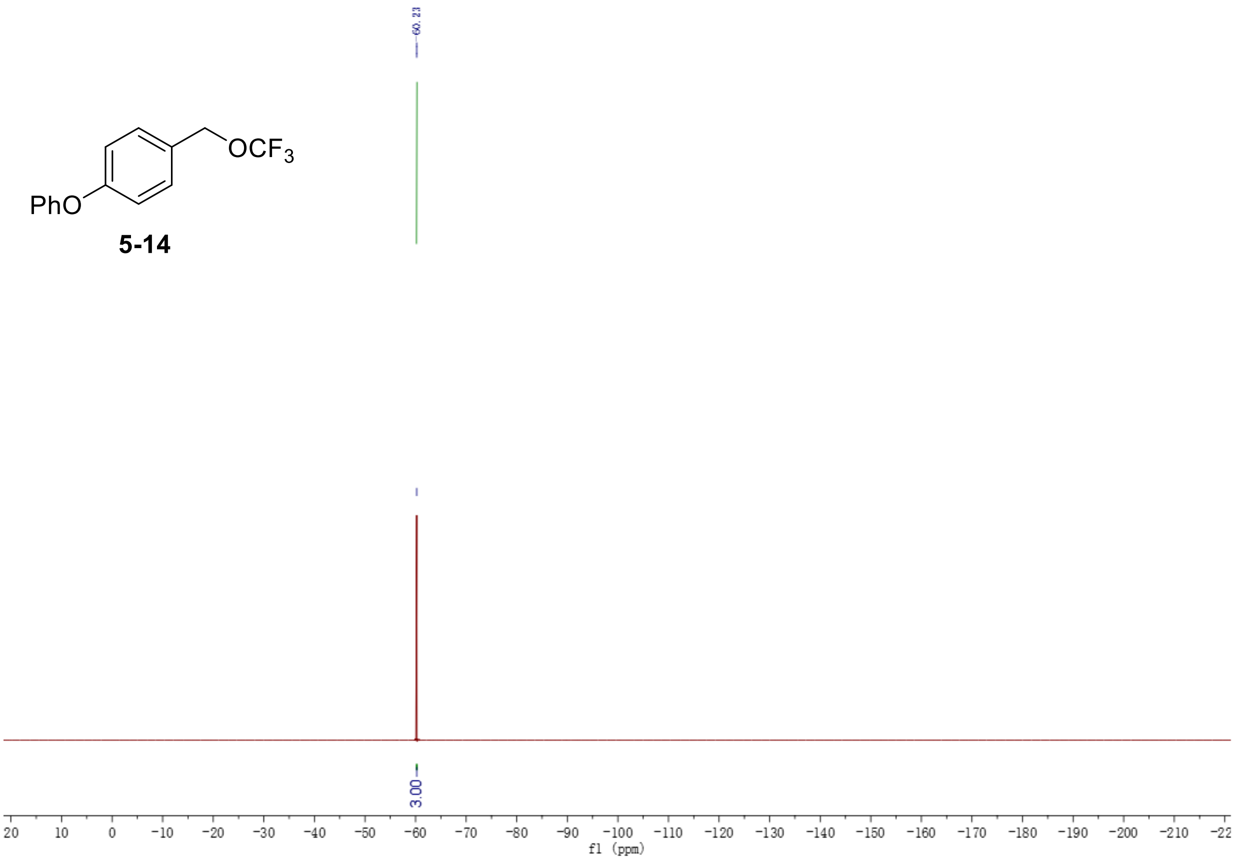

Supplementary Figure 61. ^{19}F NMR spectrum (376 MHz, $CDCl_3$) of **5-14**

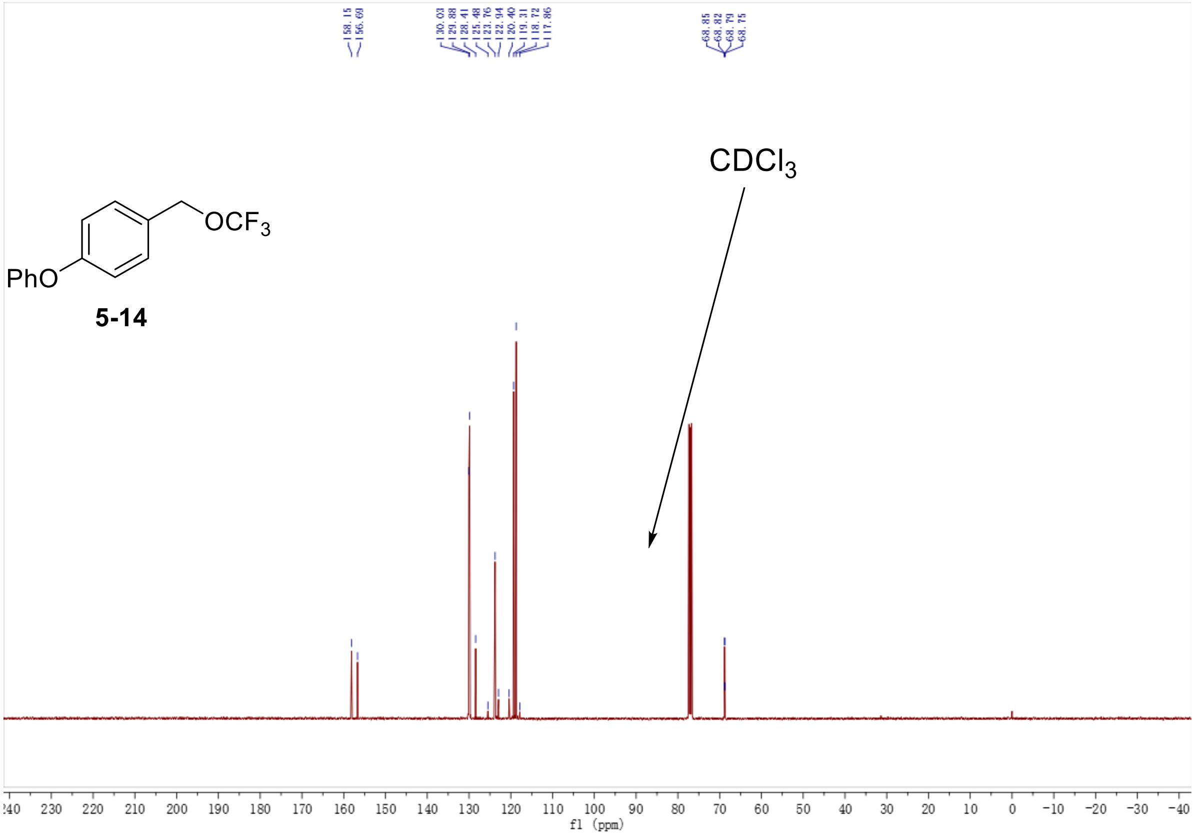

Supplementary Figure 62. ^{13}C NMR spectrum (101 MHz, $CDCl_3$) of **5-14**

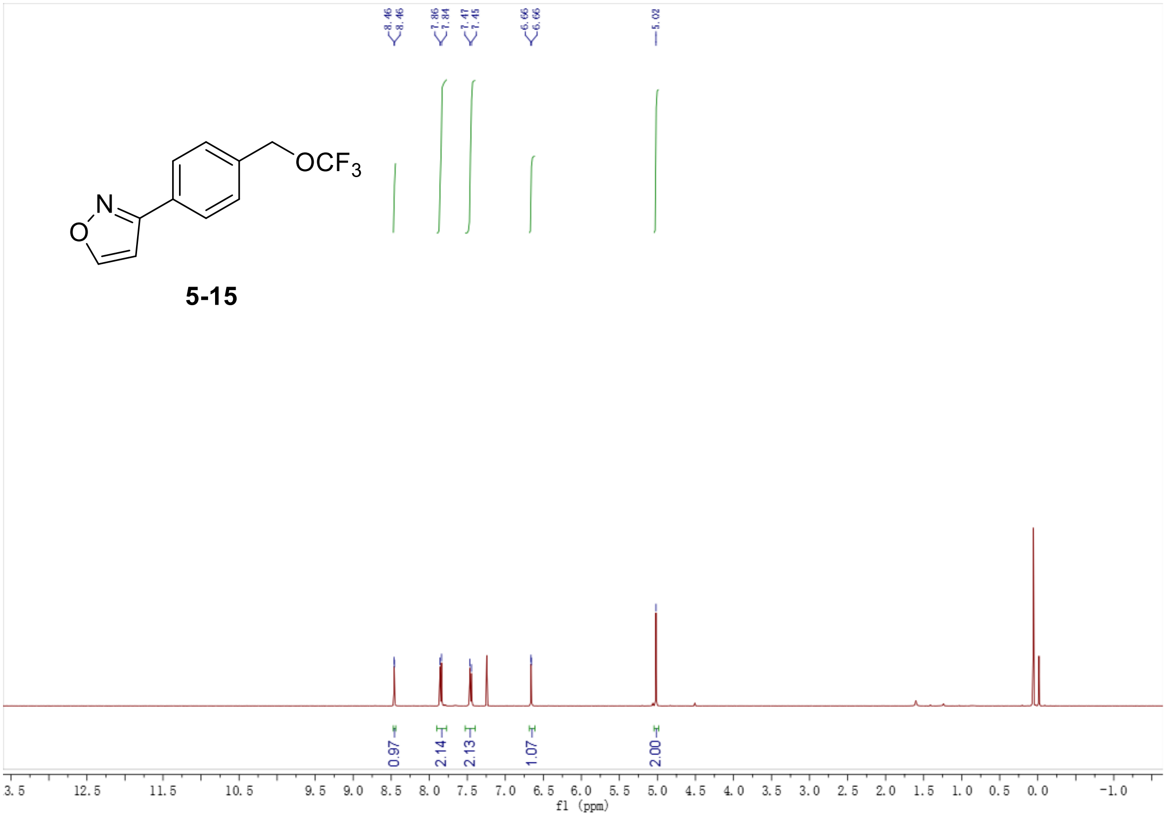

Supplementary Figure 63. ^{1}H NMR spectrum (400 MHz, $CDCl_3$) of **5-15**

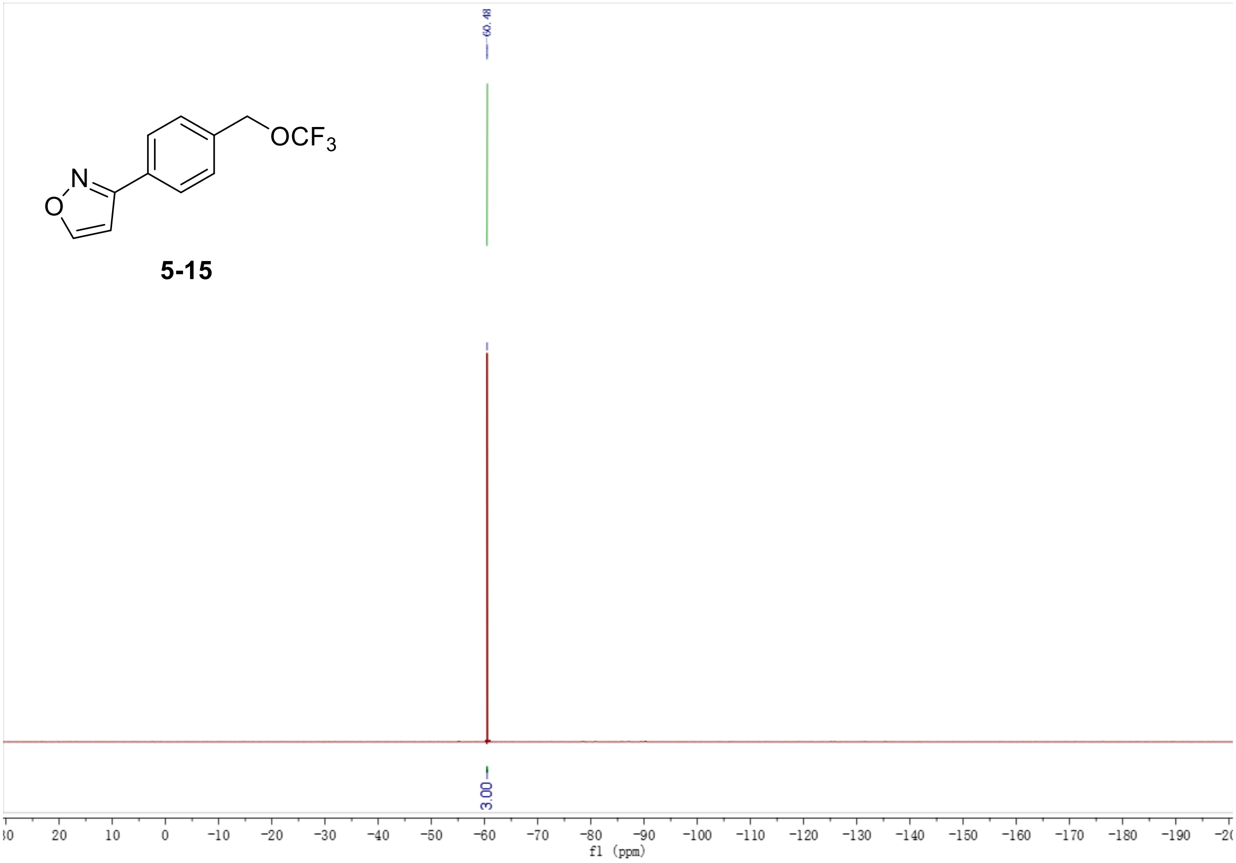

Supplementary Figure 64. ^{19}F NMR spectrum (376 MHz, $CDCl_3$) of **5-15**

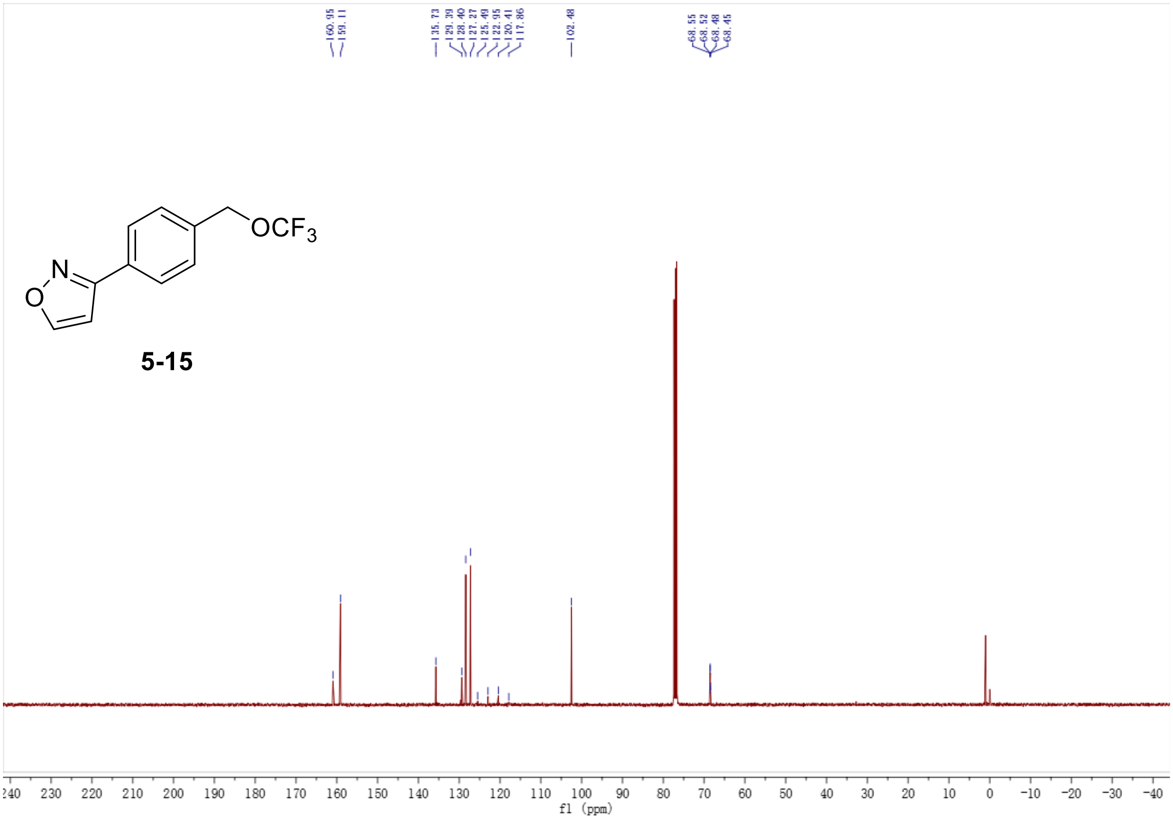

Supplementary Figure 65. ^{13}C NMR spectrum (101 MHz, $CDCl_3$) of **5-15**

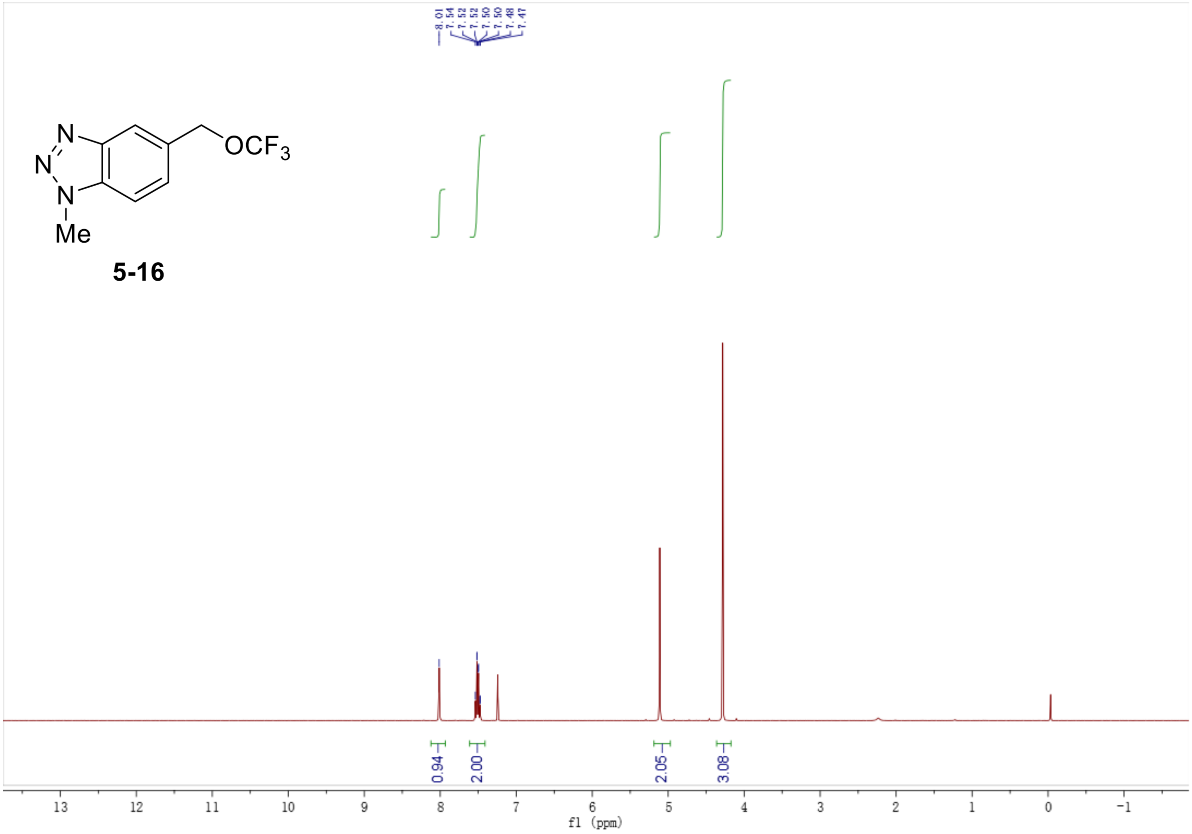

Supplementary Figure 66. ^{1}H NMR spectrum (400 MHz, $CDCl_3$) of **5-16**

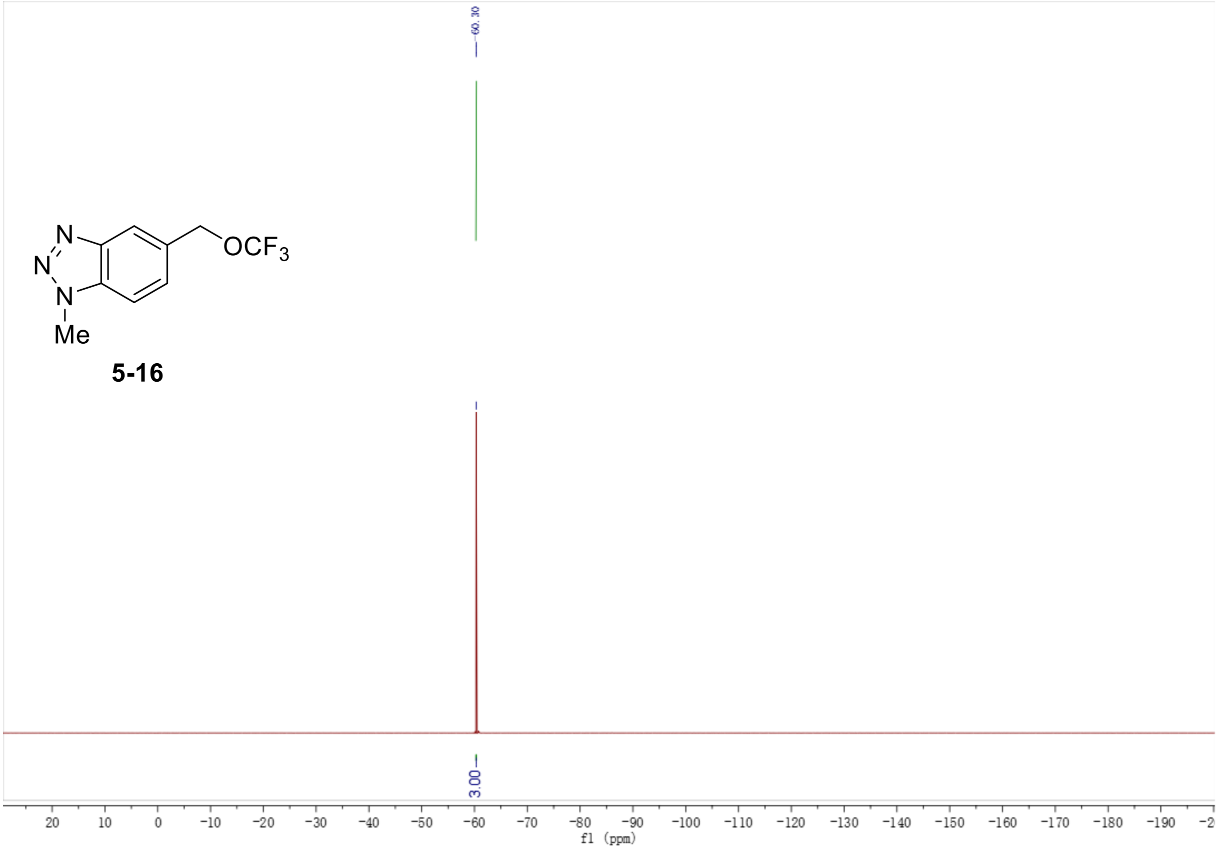

Supplementary Figure 67. ^{19}F NMR spectrum (376 MHz, $CDCl_3$) of **5-16**

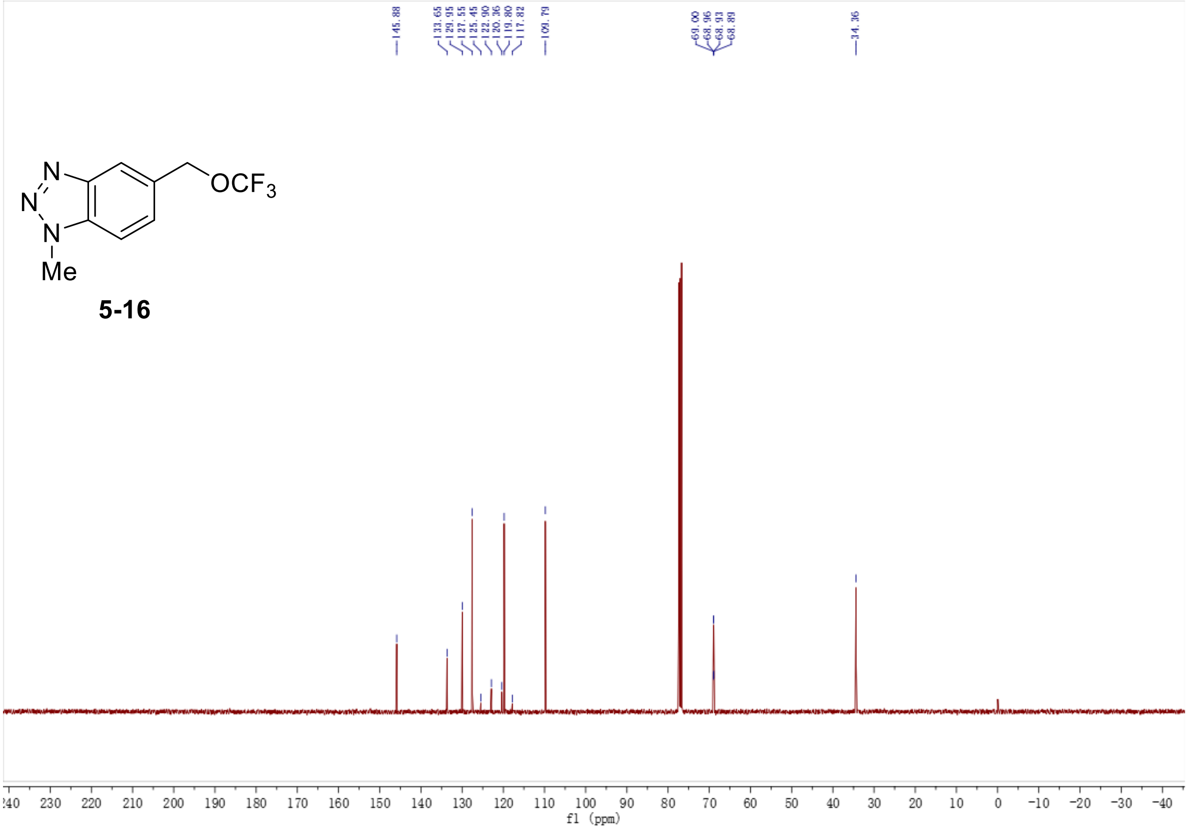

Supplementary Figure 68. ^{13}C NMR spectrum (101 MHz, $CDCl_3$) of **5-16**

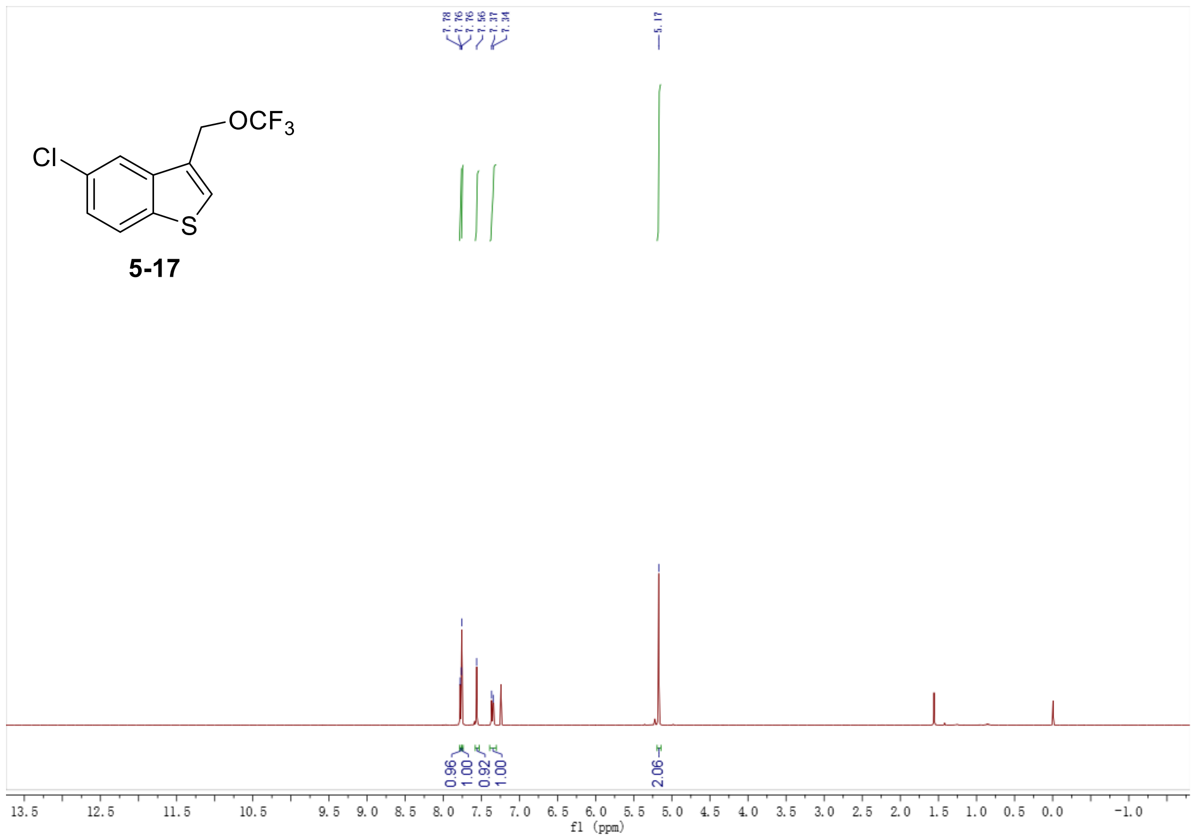

Supplementary Figure 69. ^{1}H NMR spectrum (400 MHz, $CDCl_3$) of **5-17**

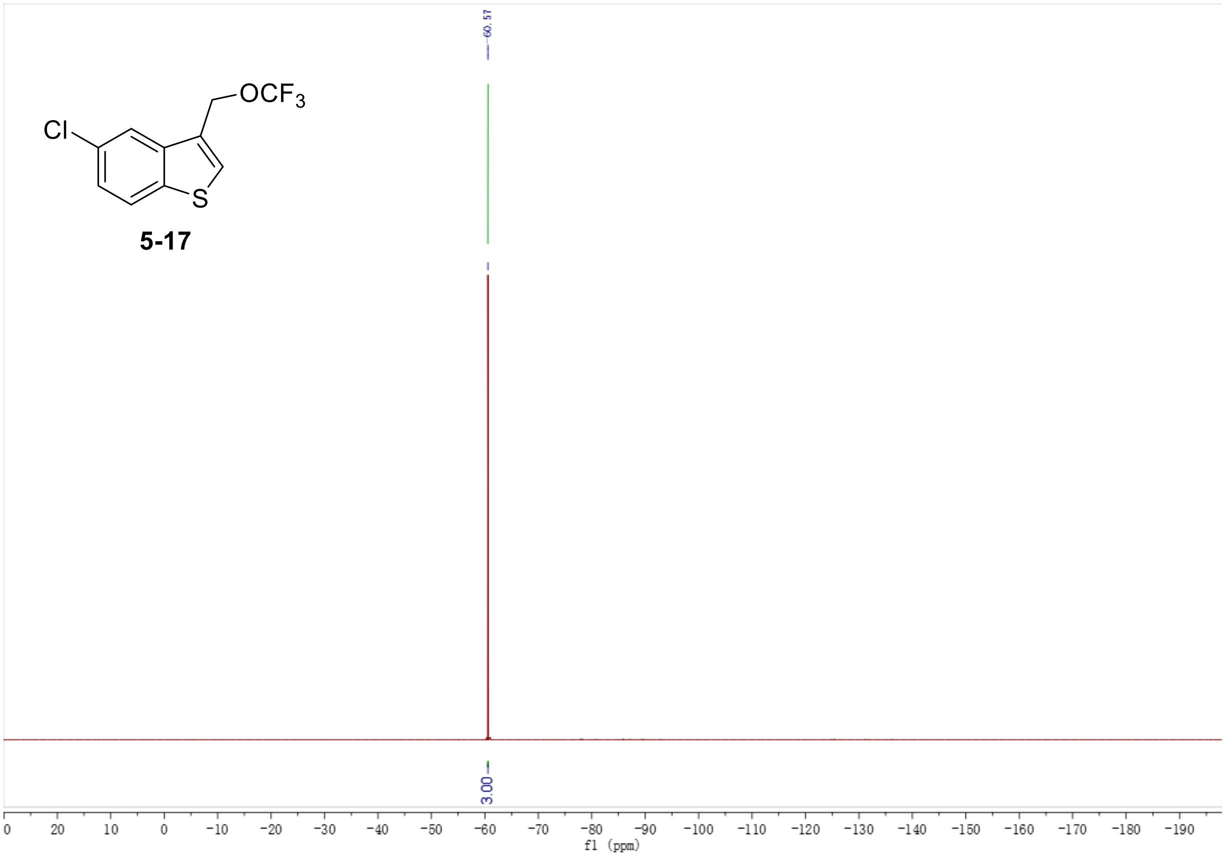

Supplementary Figure 70. ^{19}F NMR spectrum (376 MHz, $CDCl_3$) of **5-17**

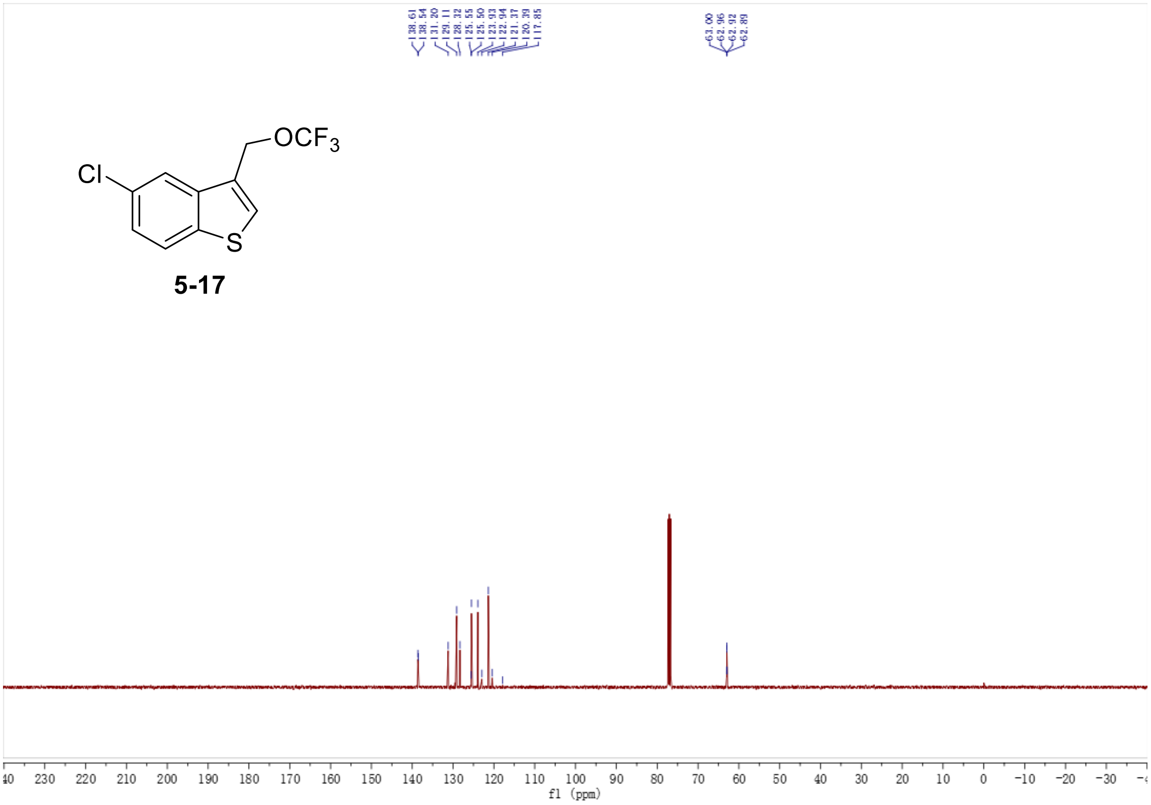

Supplementary Figure 71. ^{13}C NMR spectrum (101 MHz, $CDCl_3$) of **5-17**

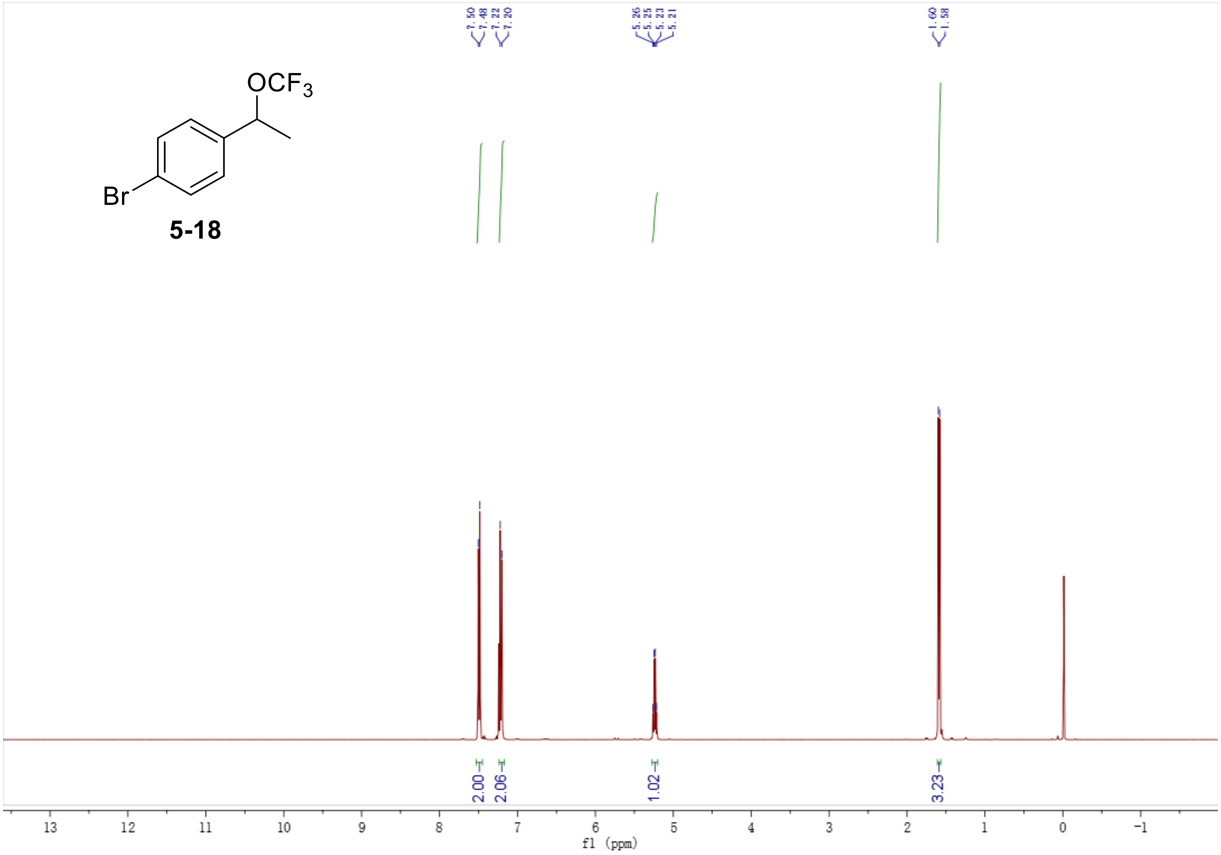

Supplementary Figure 72. ^{1}H NMR spectrum (400 MHz, $CDCl_3$) of **5-18**

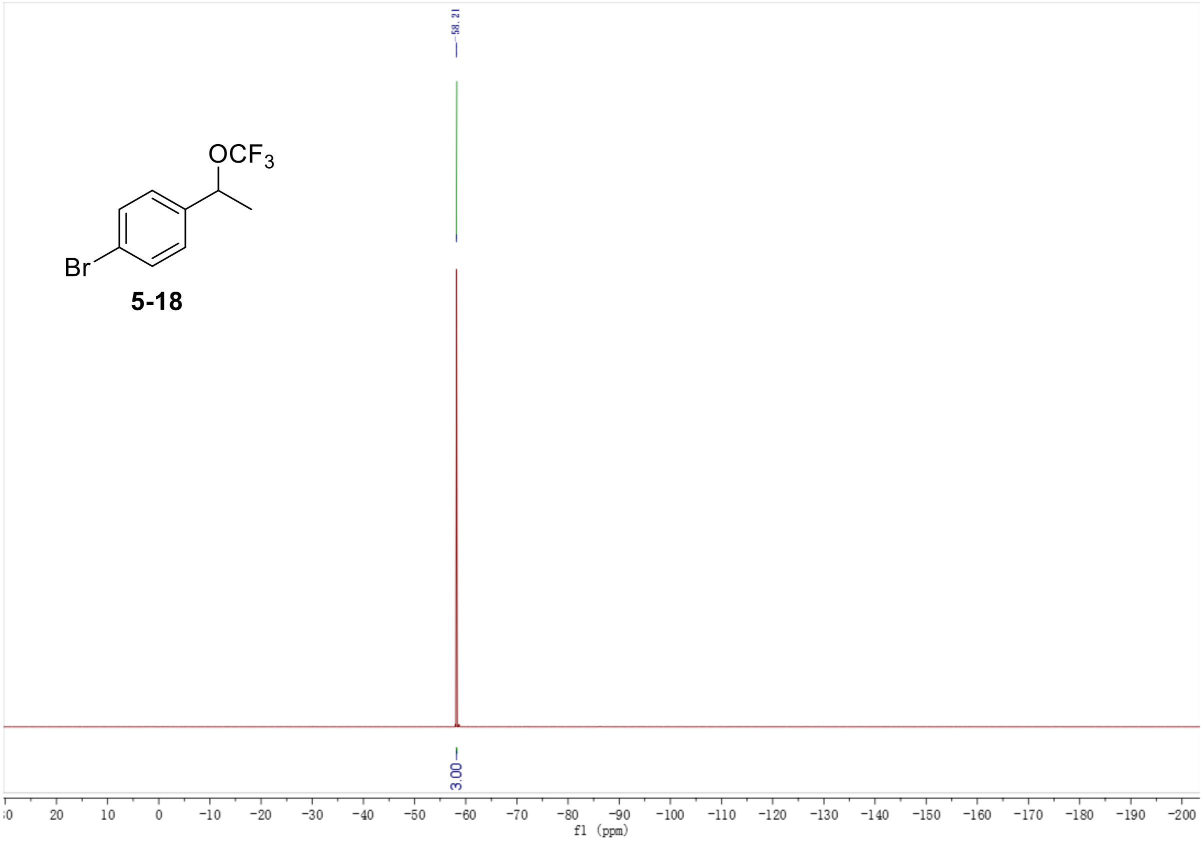

Supplementary Figure 73. ^{19}F NMR spectrum (376 MHz, $CDCl_3$) of **5-18**

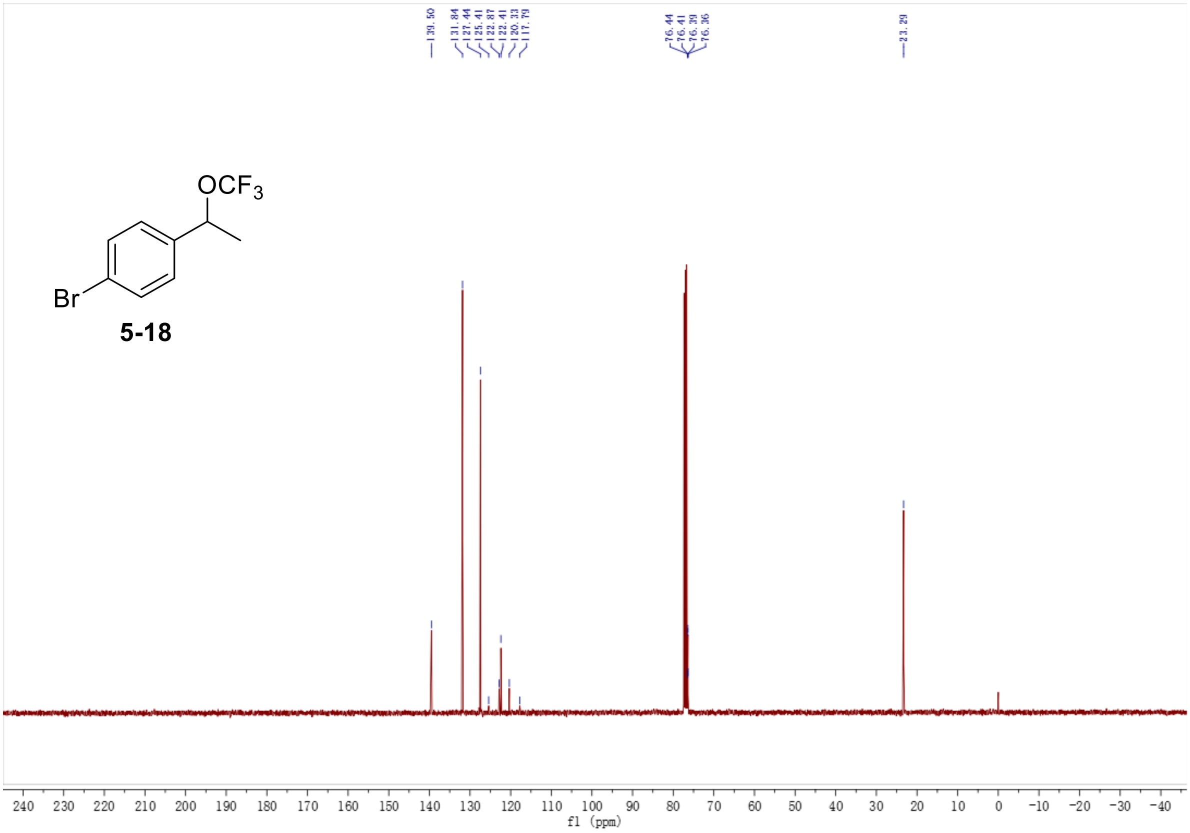

Supplementary Figure 74. ^{13}C NMR spectrum (101 MHz, $CDCl_3$) of **5-18**

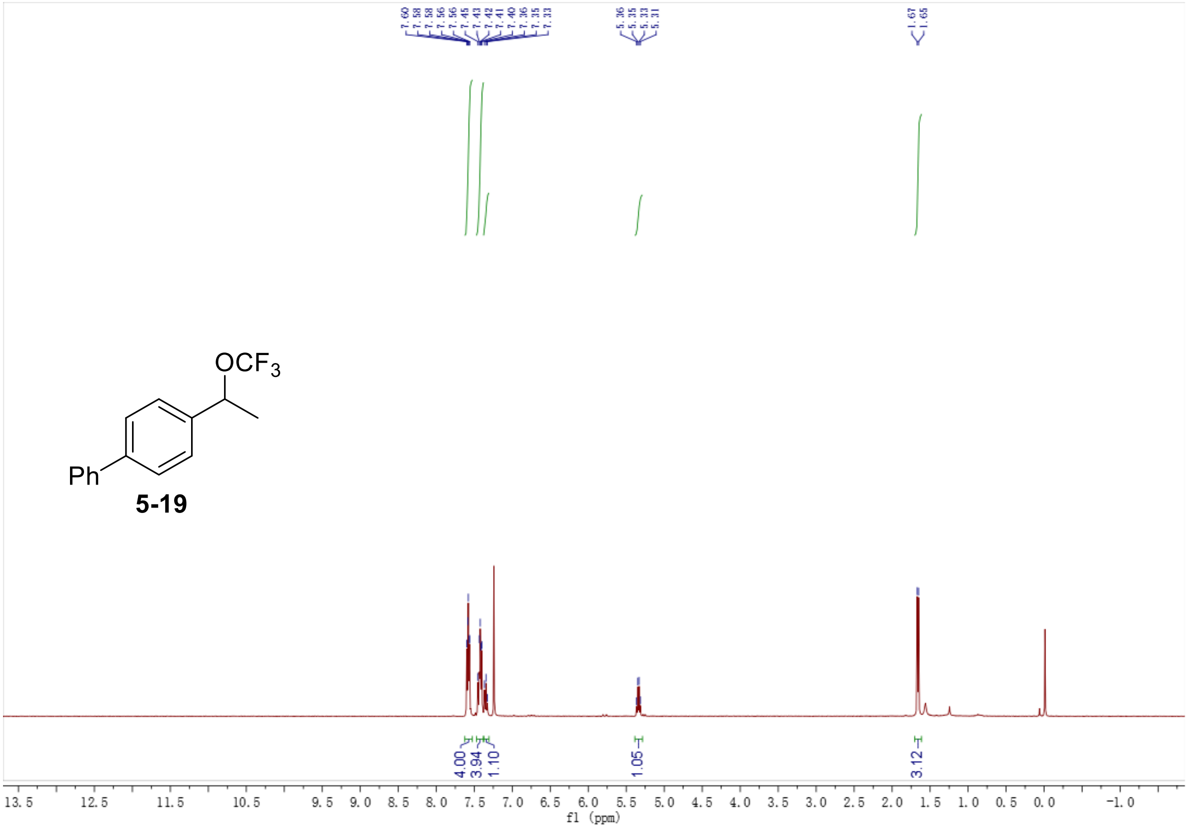

Supplementary Figure 75. ^{1}H NMR spectrum (400 MHz, $CDCl_3$) of **5-19**

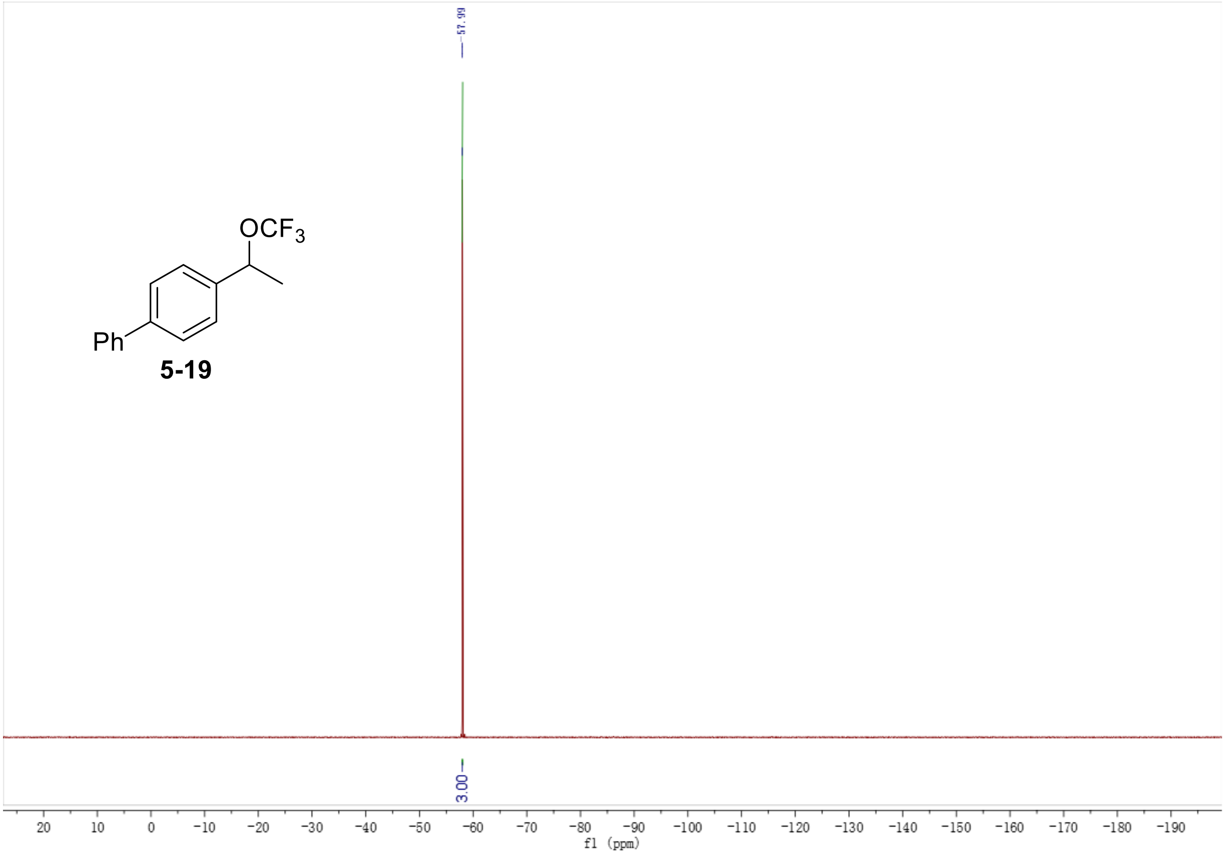

Supplementary Figure 76. ^{19}F NMR spectrum (376 MHz, $CDCl_3$) of **5-19**

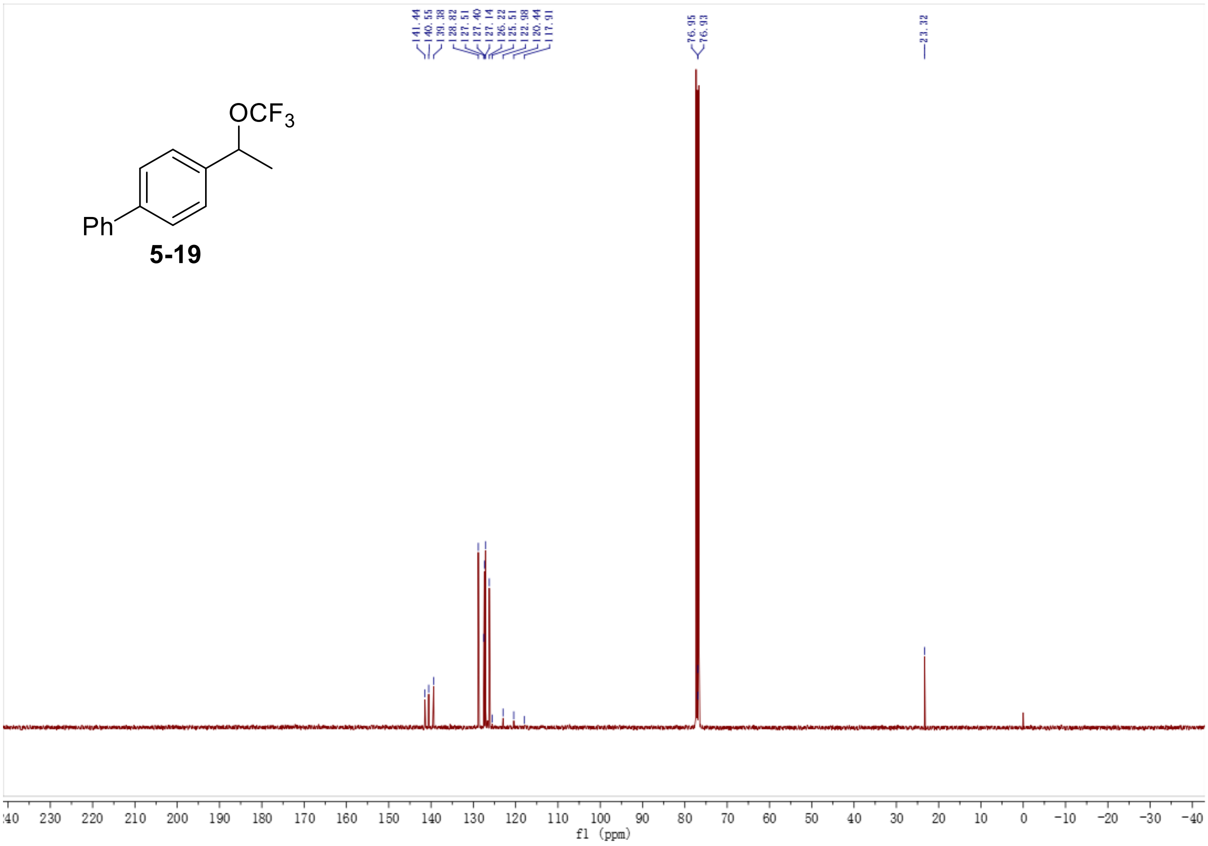

Supplementary Figure 77. ^{13}C NMR spectrum (101 MHz, $CDCl_3$) of **5-19**

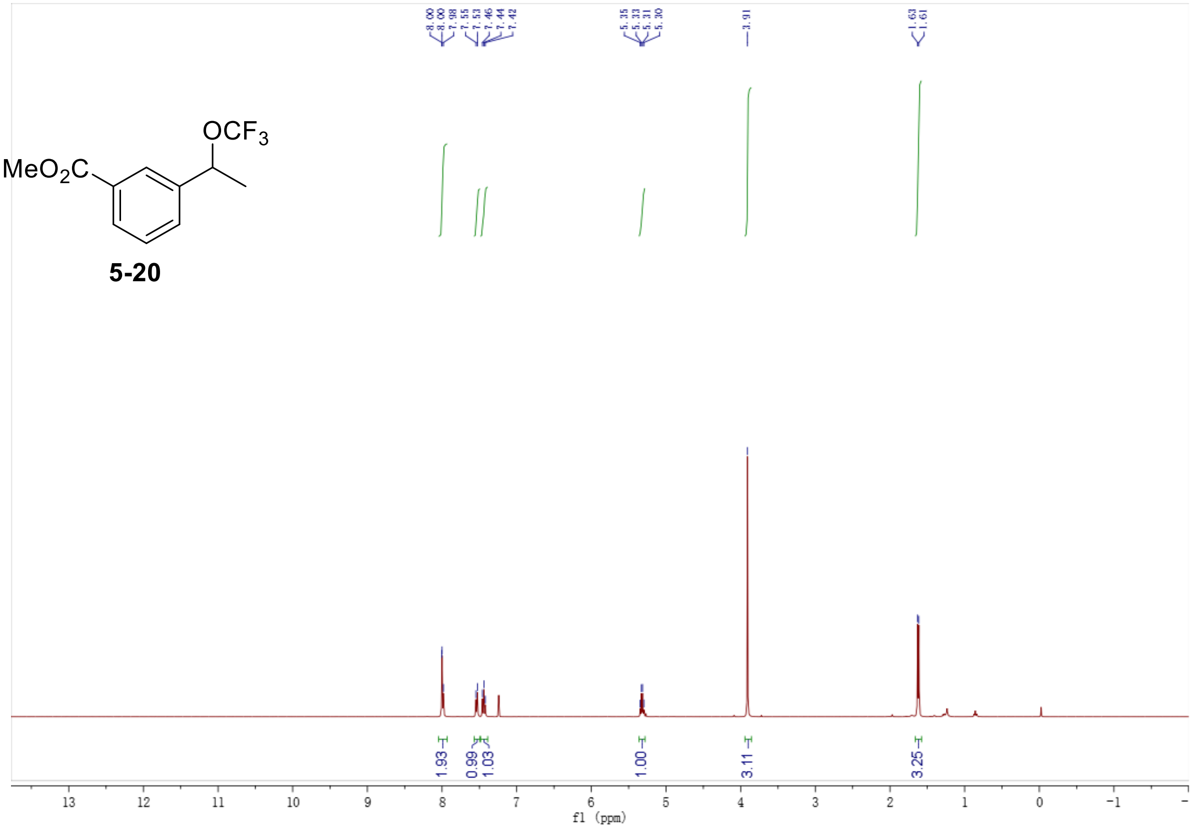

Supplementary Figure 78. ^{1}H NMR spectrum (400 MHz, $CDCl_3$) of **5-20**

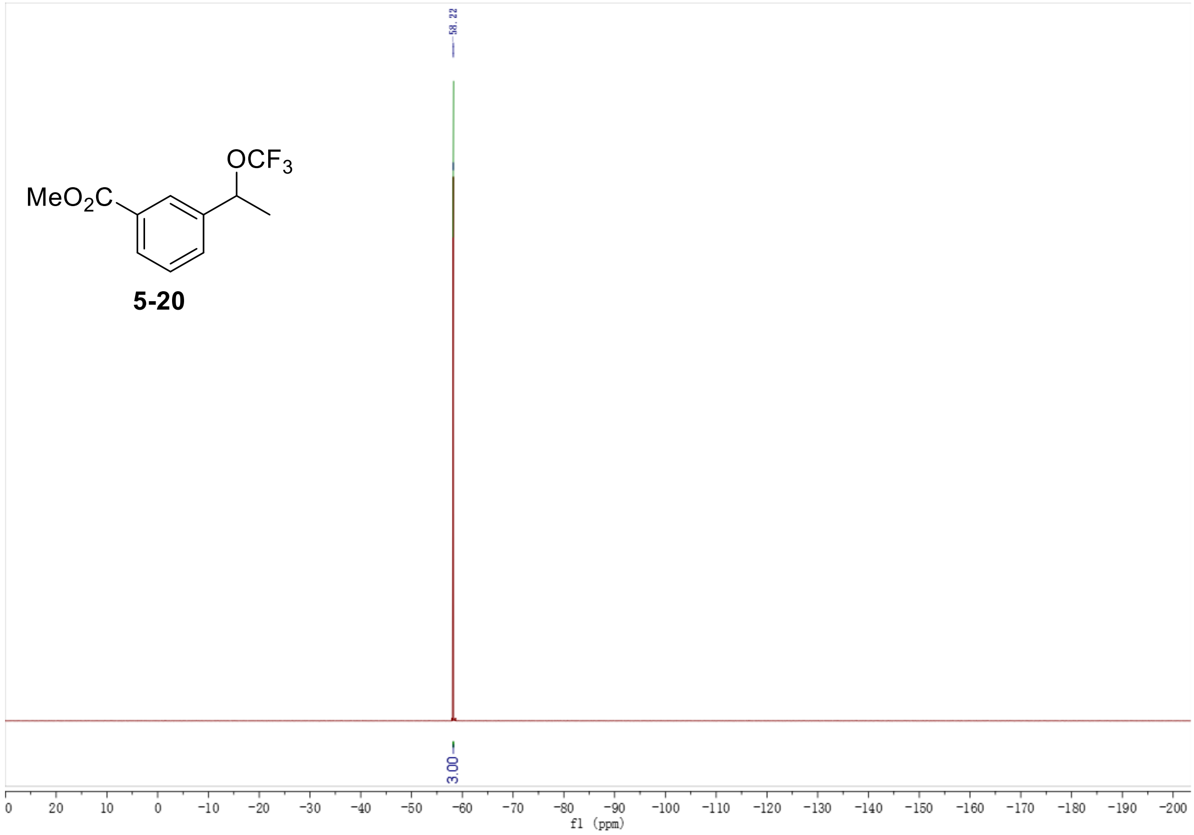

Supplementary Figure 79. ^{19}F NMR spectrum (376 MHz, $CDCl_3$) of **5-20**

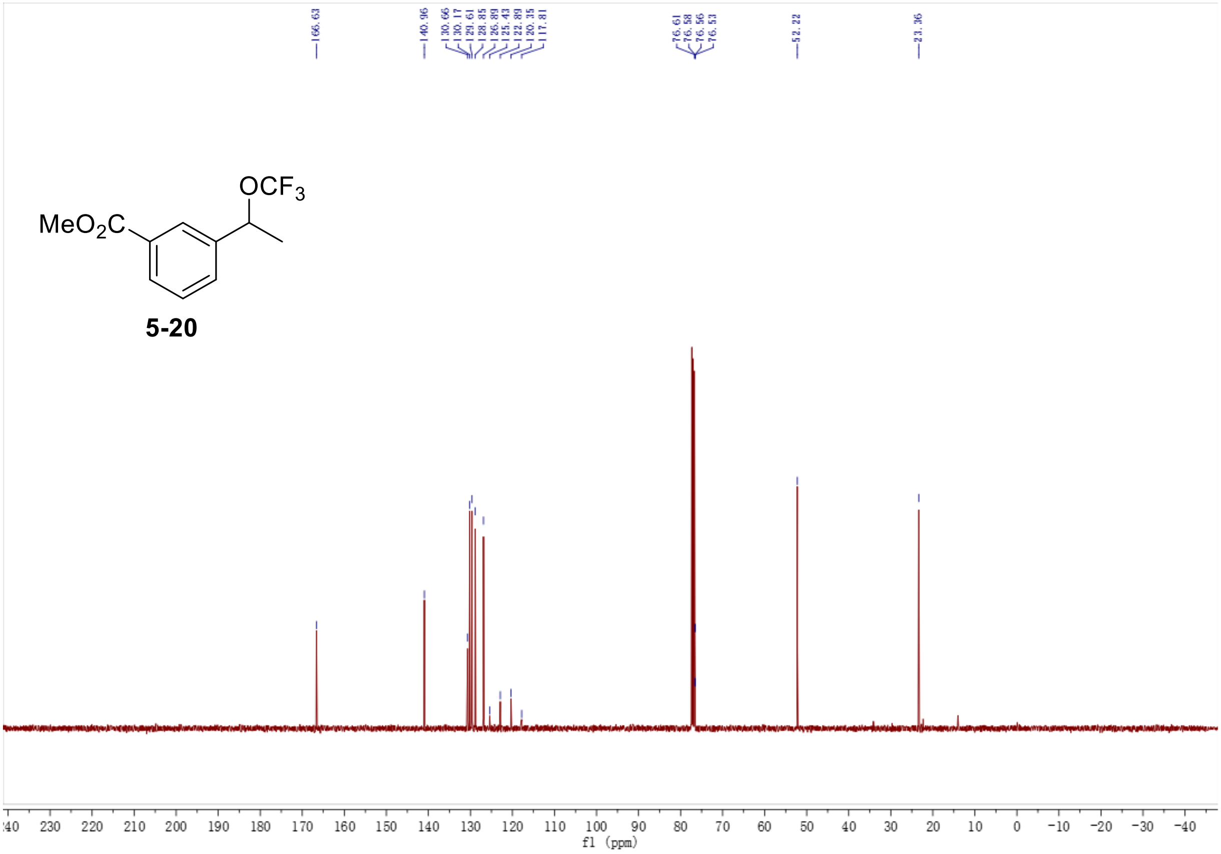

Supplementary Figure 80. ^{13}C NMR spectrum (101 MHz, $CDCl_3$) of **5-20**

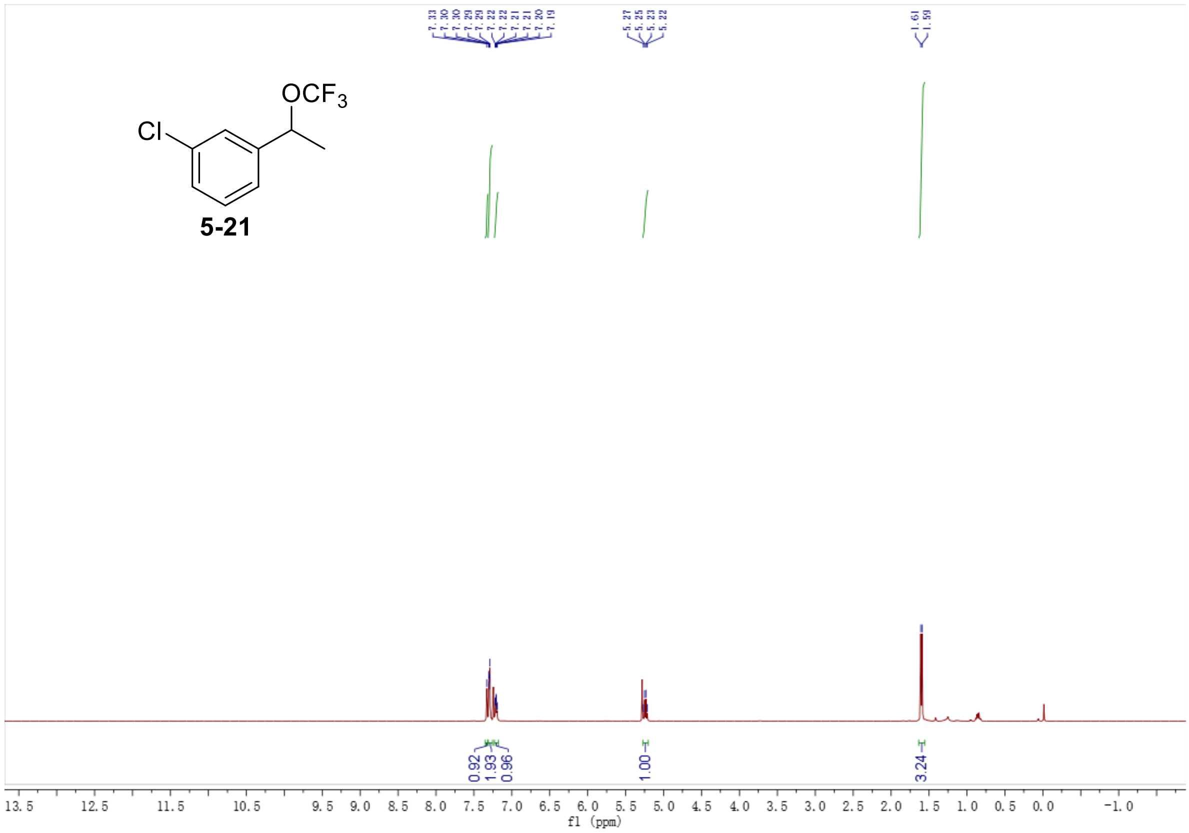

Supplementary Figure 81. ^{1}H NMR spectrum (400 MHz, $CDCl_3$) of **5-21**

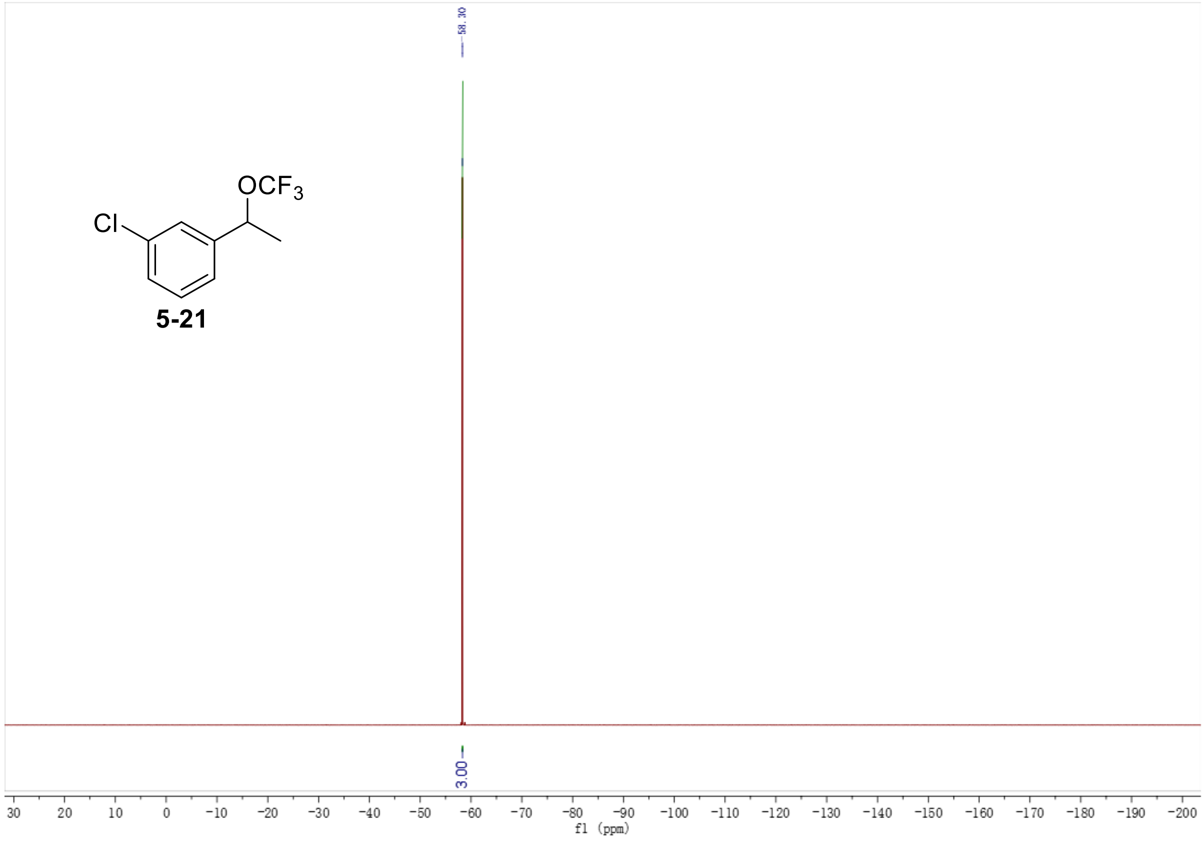

Supplementary Figure 82. ^{19}F NMR spectrum (376 MHz, $CDCl_3$) of **5-21**

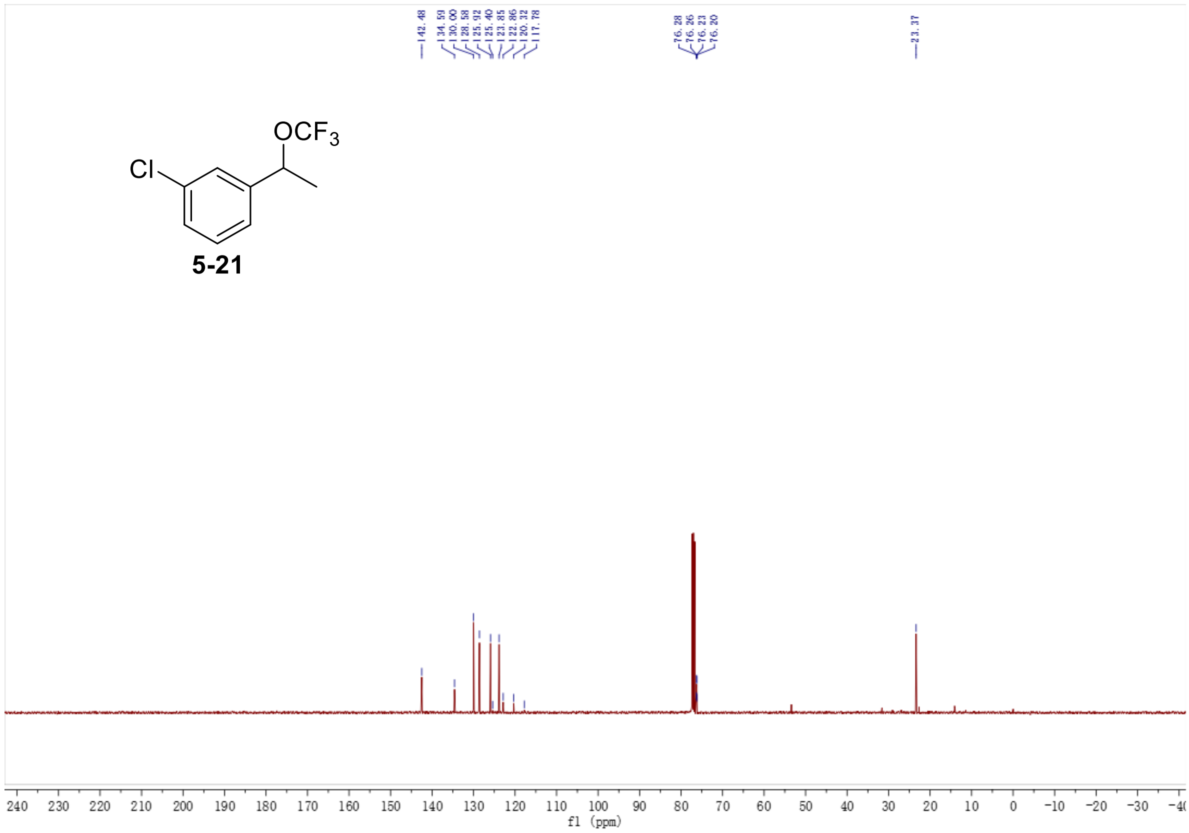

Supplementary Figure 83. ^{13}C NMR spectrum (101 MHz, $CDCl_3$) of **5-21**

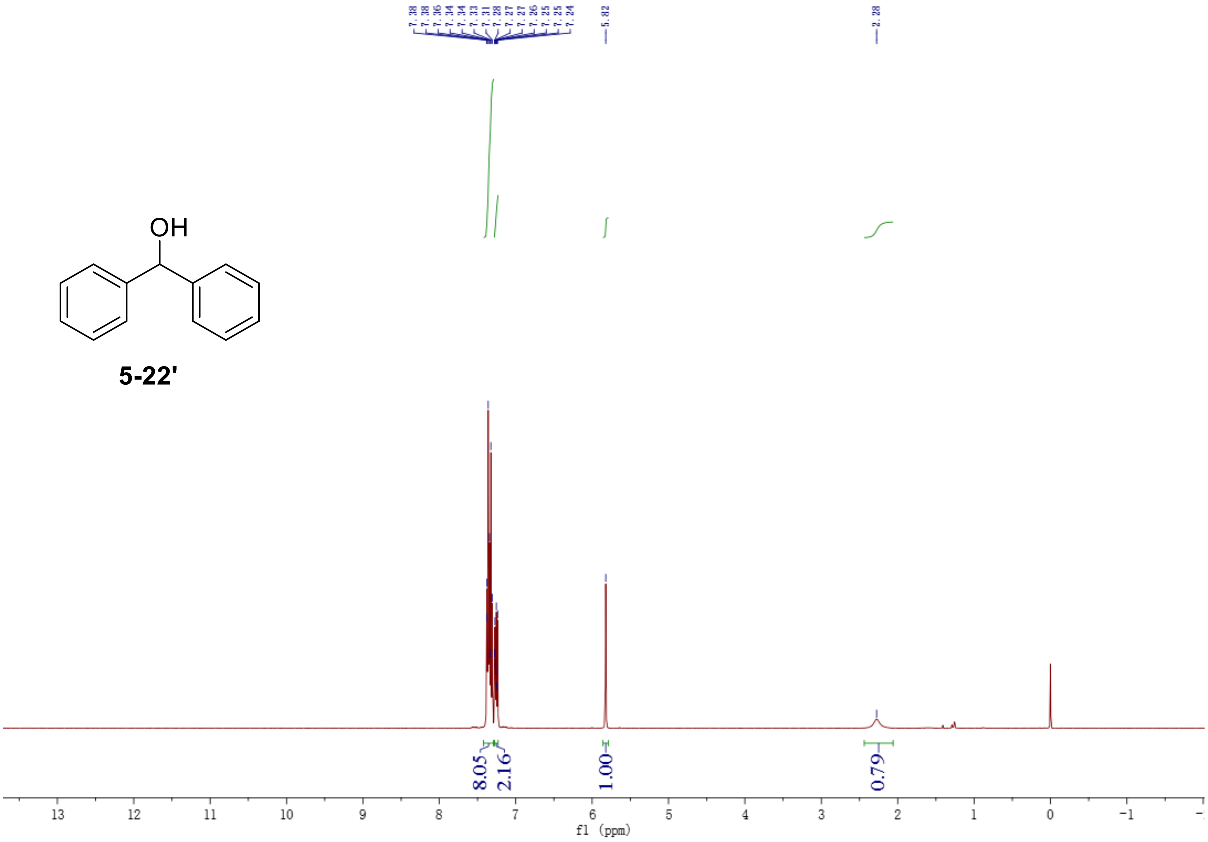

Supplementary Figure 84. ^{1}H NMR spectrum (400 MHz, $CDCl_3$) of **5-22’**

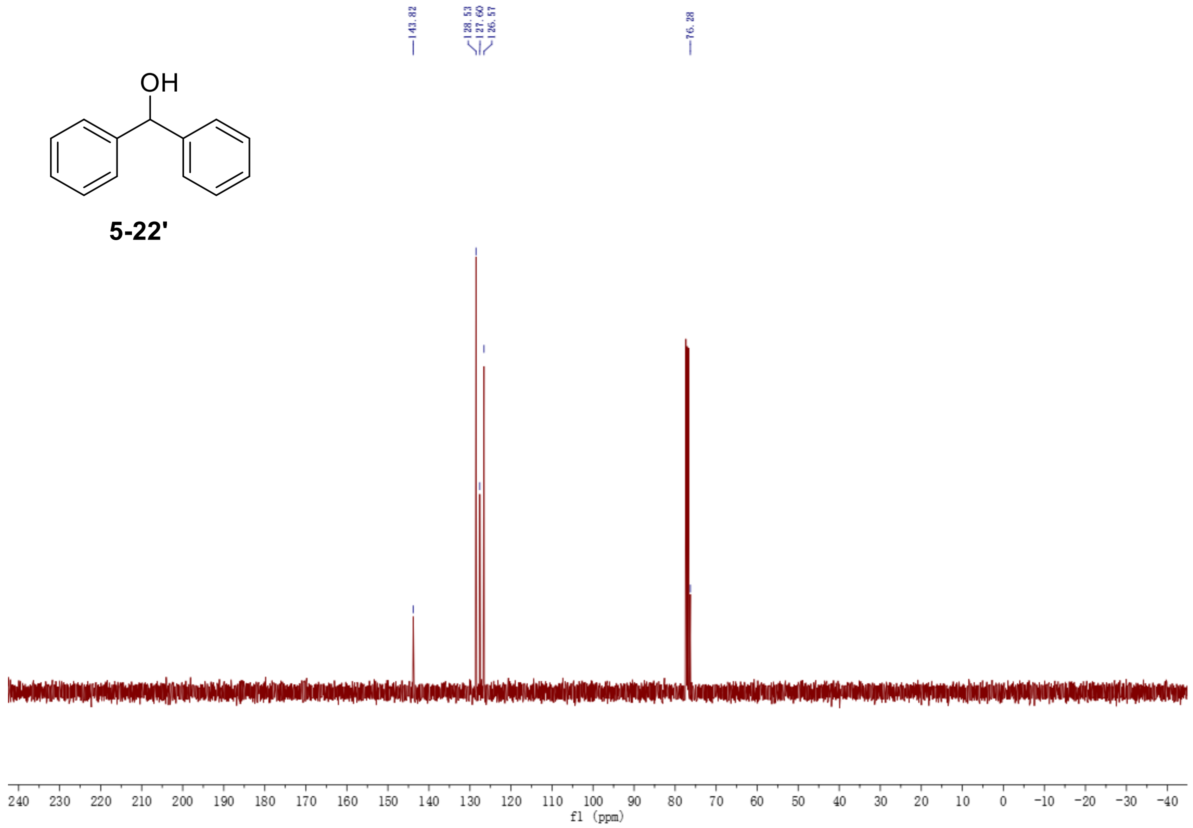

Supplementary Figure 85. ^{13}C NMR spectrum (101 MHz, $CDCl_3$) of **5-22’**

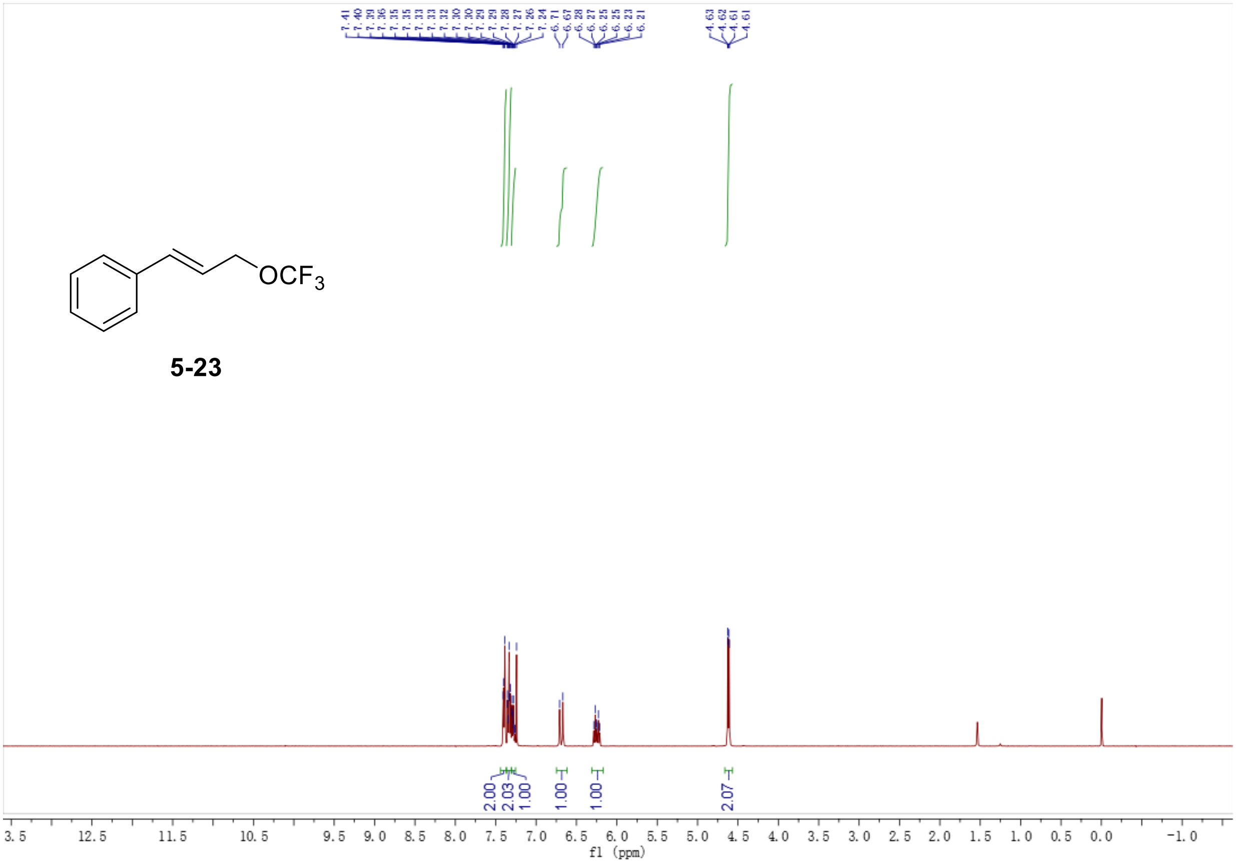

Supplementary Figure 86. ^{1}H NMR spectrum (400 MHz, $CDCl_3$) of **5-23**

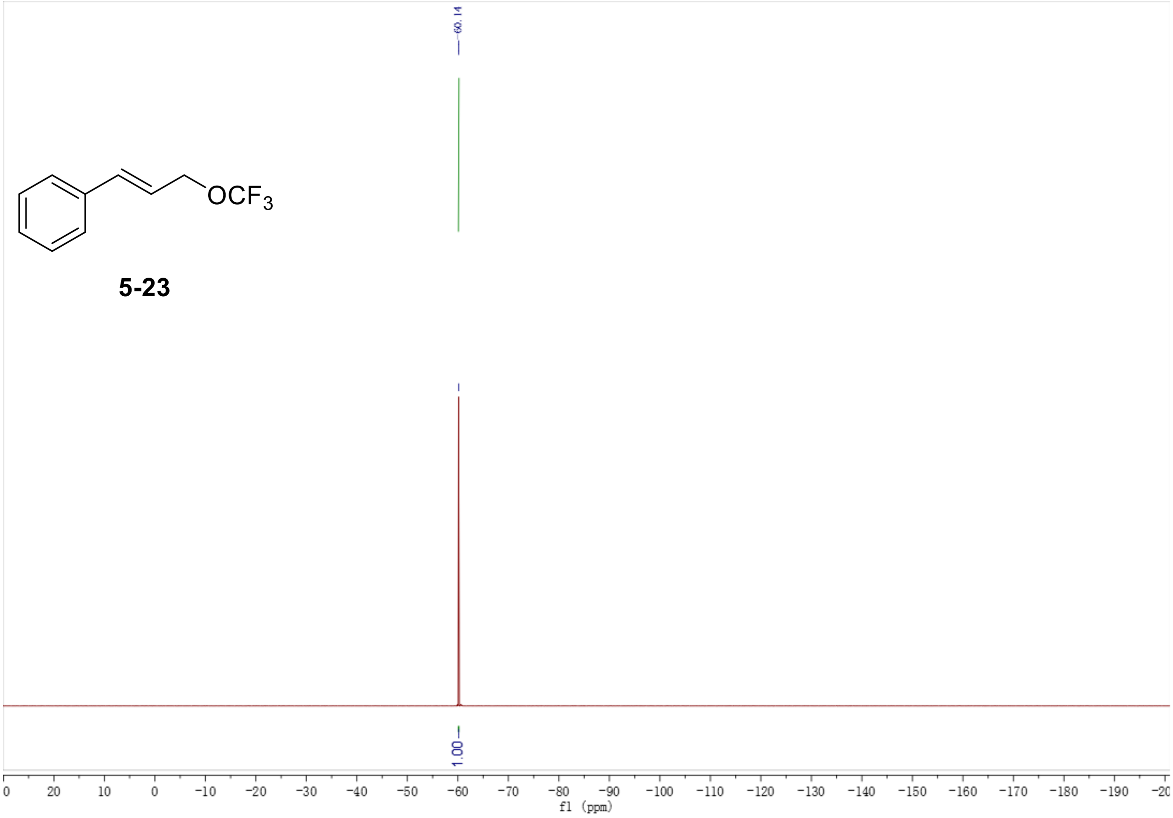

Supplementary Figure 87. ^{19}F NMR spectrum (376 MHz, $CDCl_3$) of **5-23**

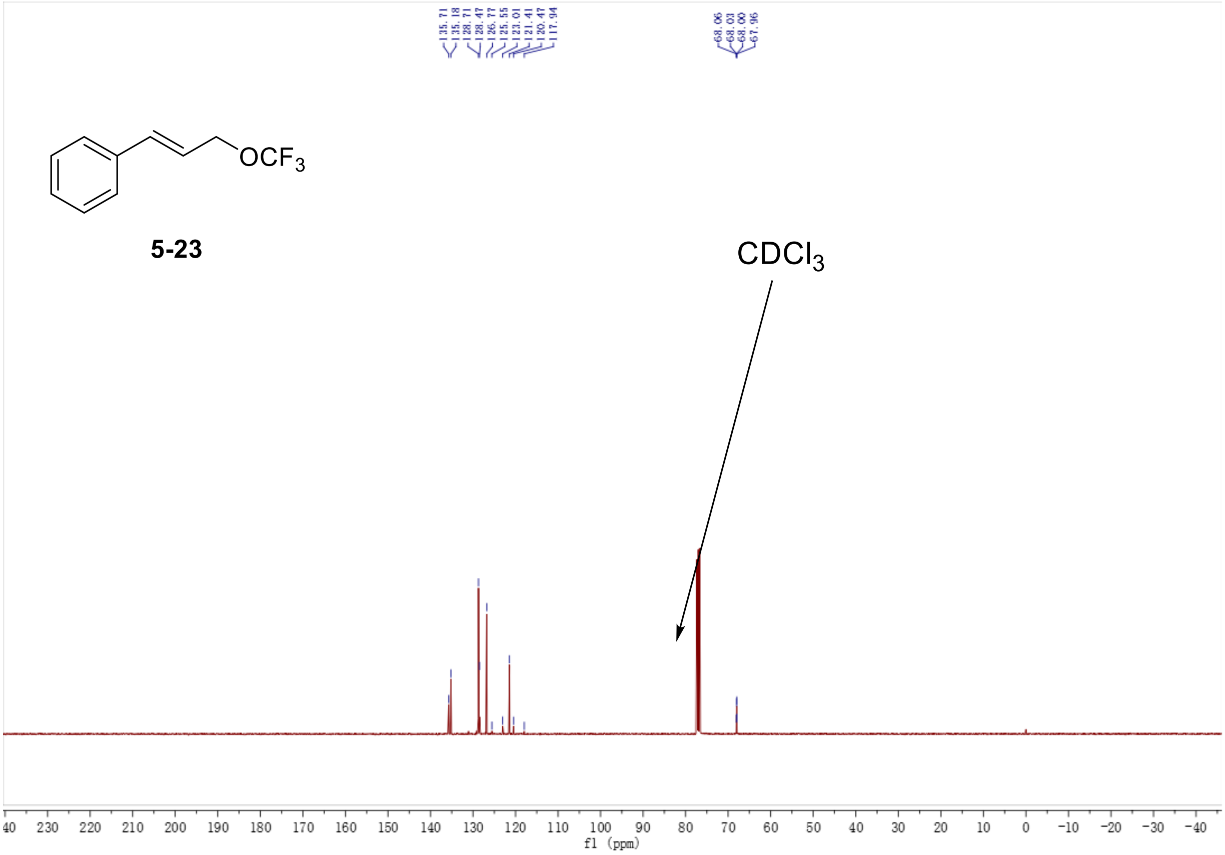

Supplementary Figure 88. ^{13}C NMR spectrum (101 MHz, $CDCl_3$) of **5-23**

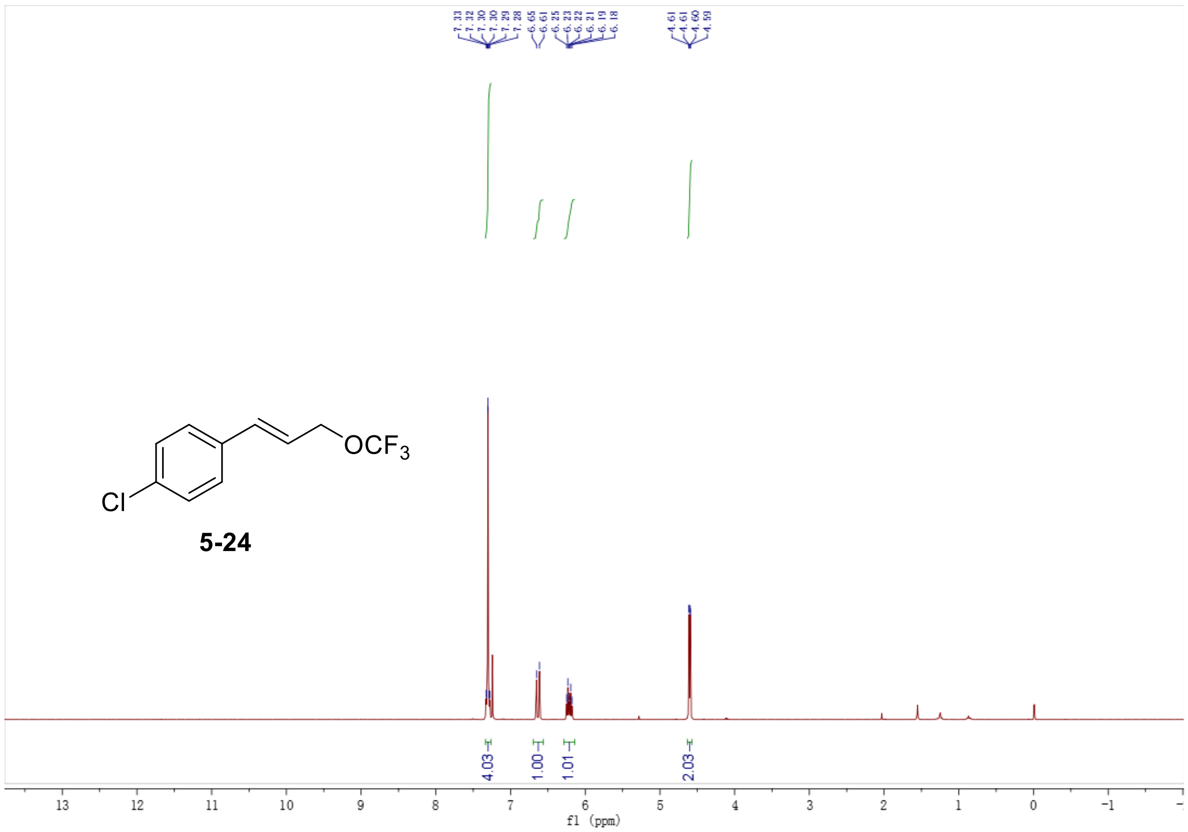

Supplementary Figure 89. ^{1}H NMR spectrum (400 MHz, $CDCl_3$) of **5-24**

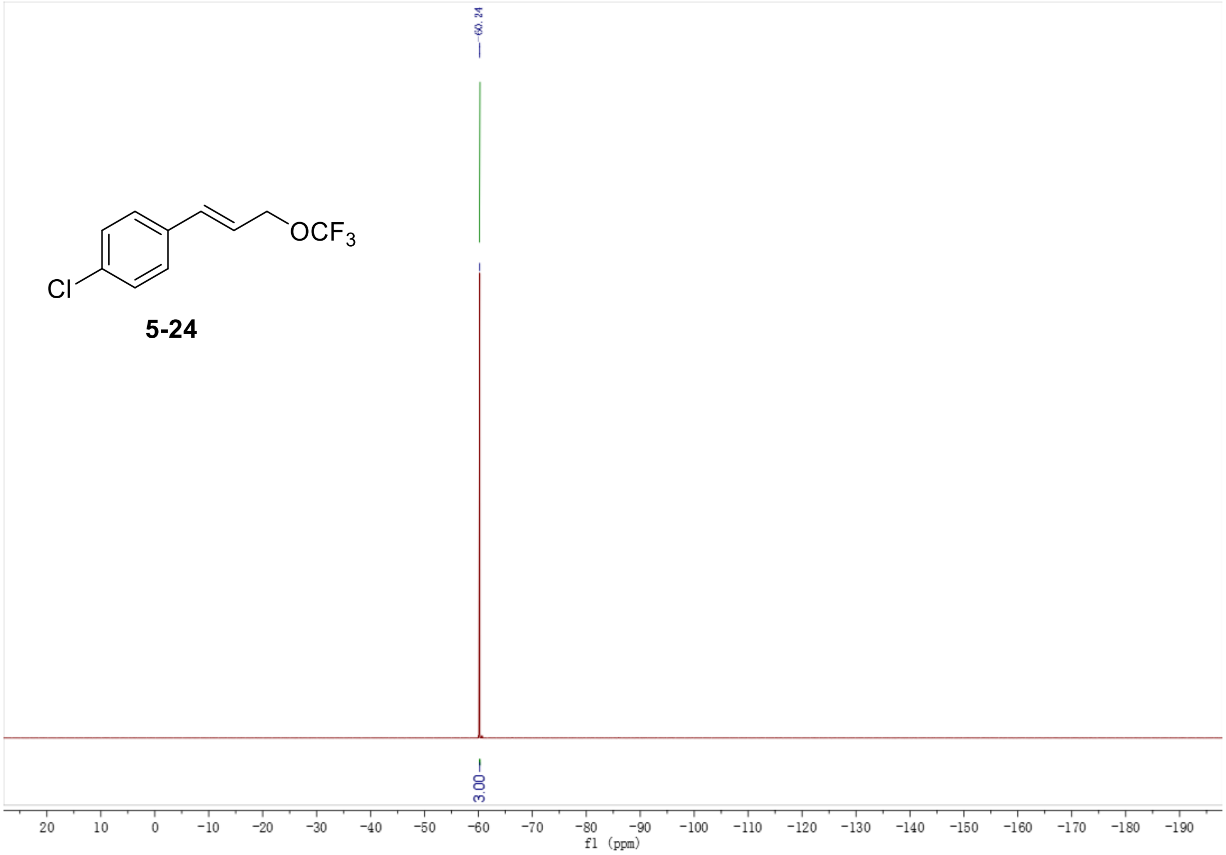

Supplementary Figure 90. ^{19}F NMR spectrum (376 MHz, $CDCl_3$) of **5-24**

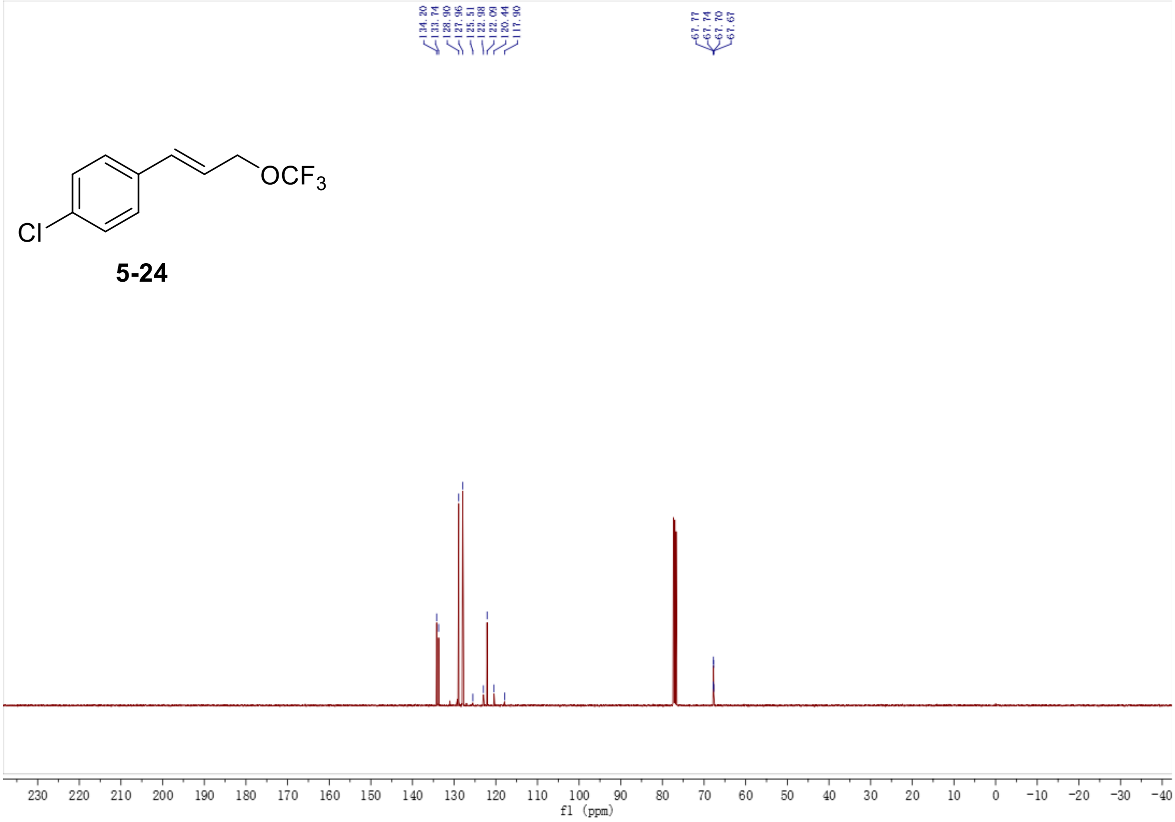

Supplementary Figure 91. ^{13}C NMR spectrum (101 MHz, $CDCl_3$) of **5-24**

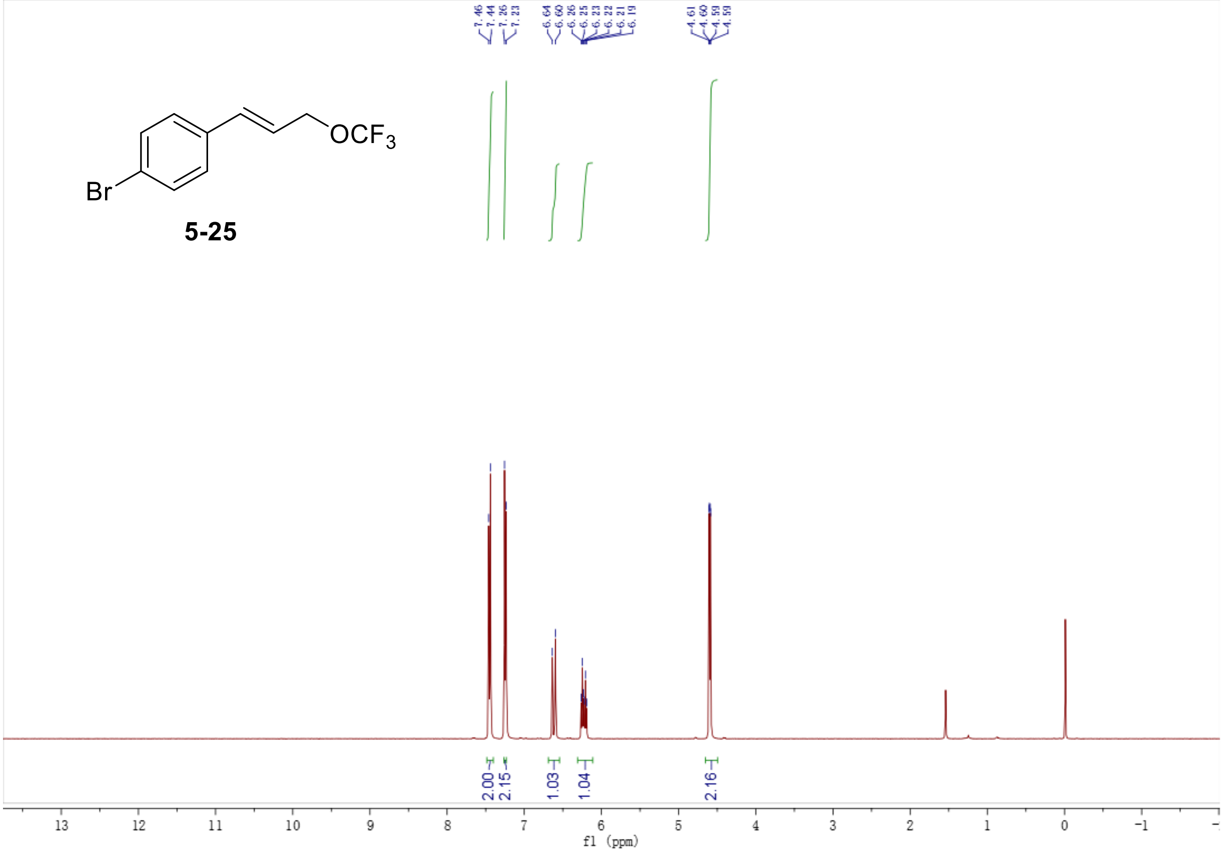

Supplementary Figure 92. ^{1}H NMR spectrum (400 MHz, $CDCl_3$) of **5-25**

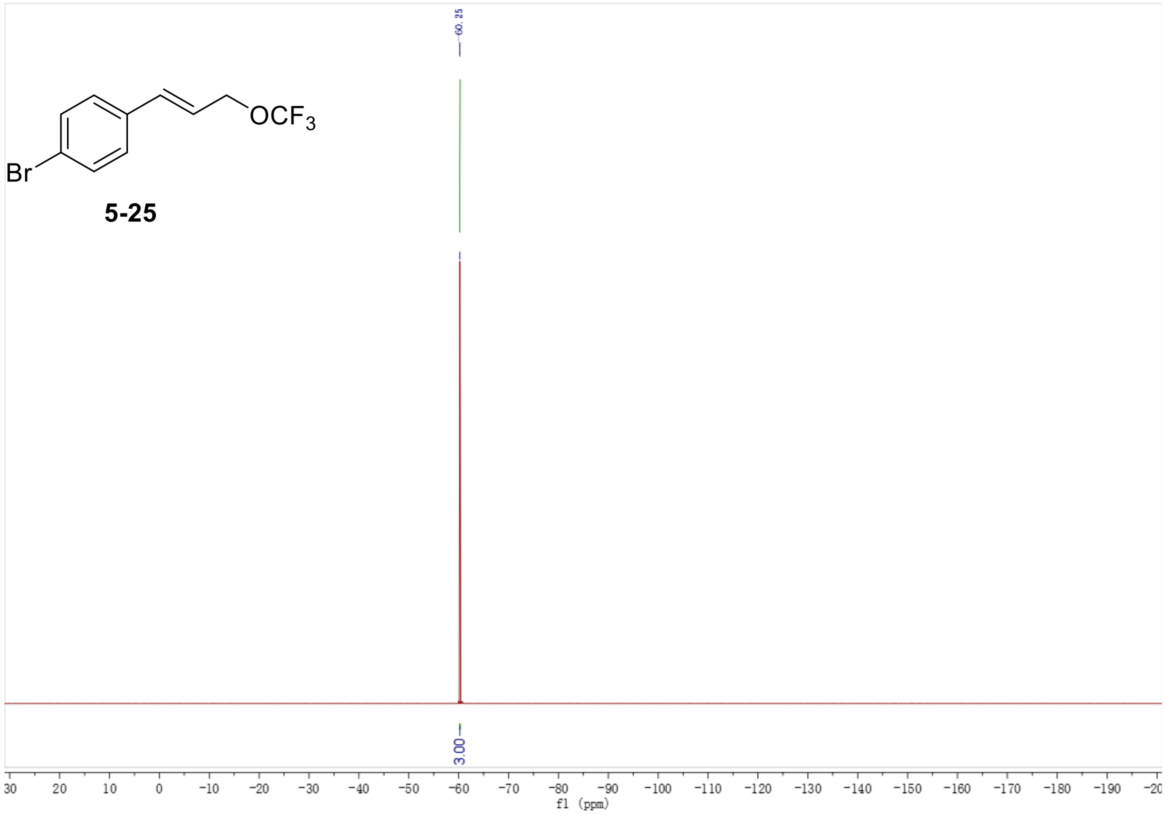

Supplementary Figure 93. ^{19}F NMR spectrum (376 MHz, $CDCl_3$) of **5-25**

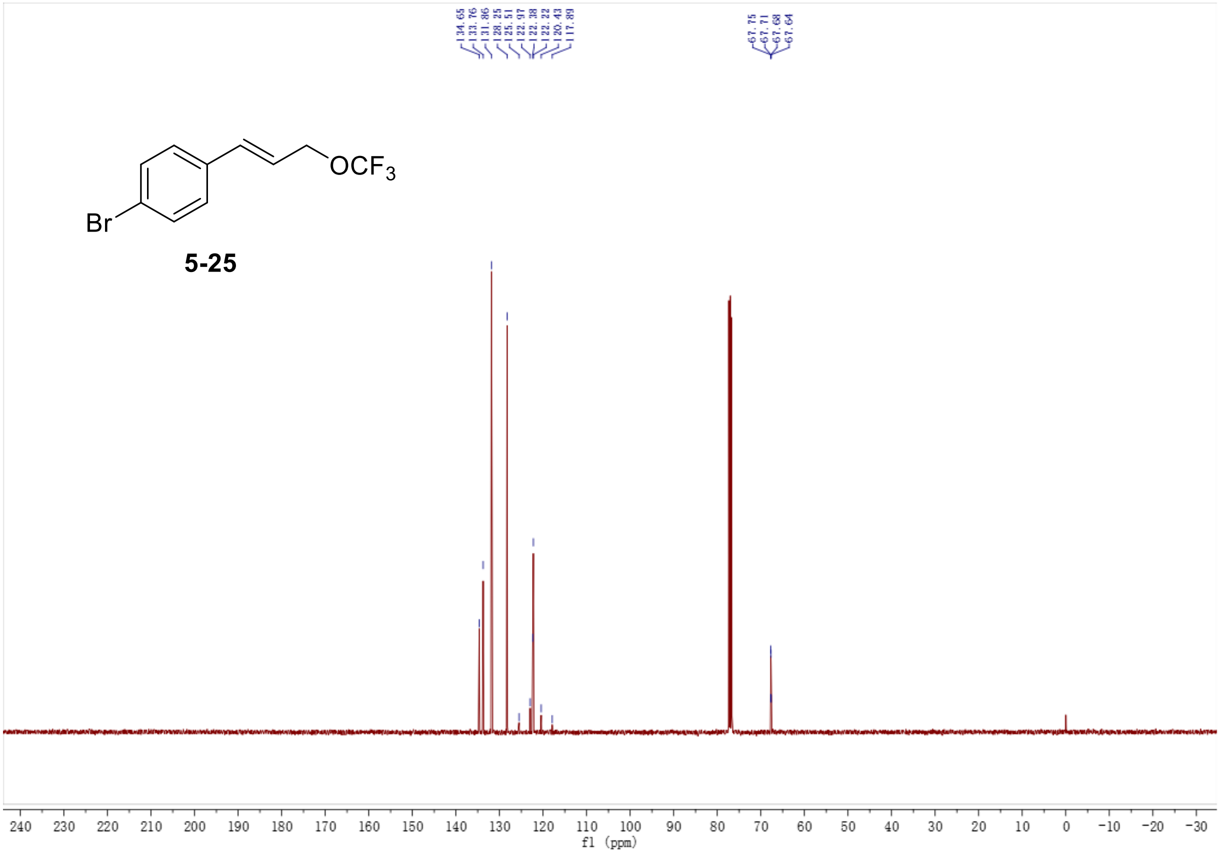

Supplementary Figure 94. ^{13}C NMR spectrum (101 MHz, $CDCl_3$) of **5-25**

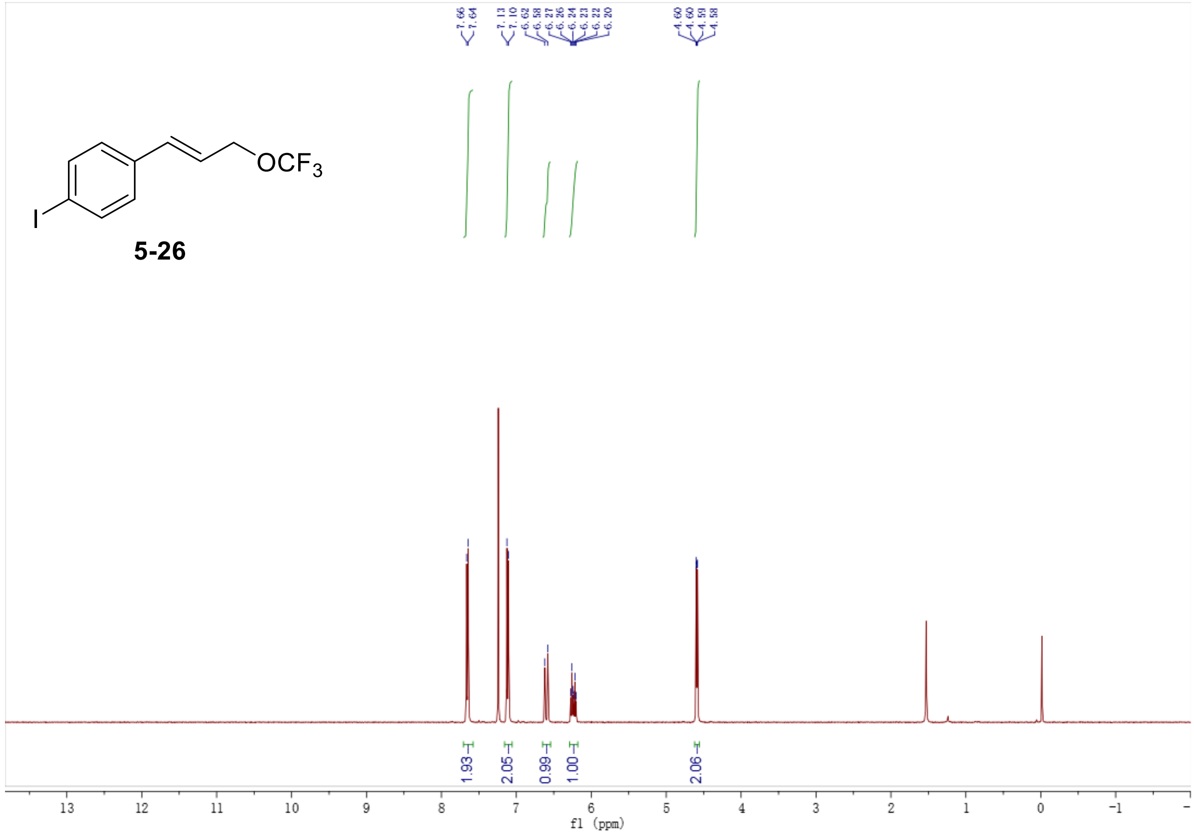

Supplementary Figure 95. ^{1}H NMR spectrum (400 MHz, $CDCl_3$) of **5-26**

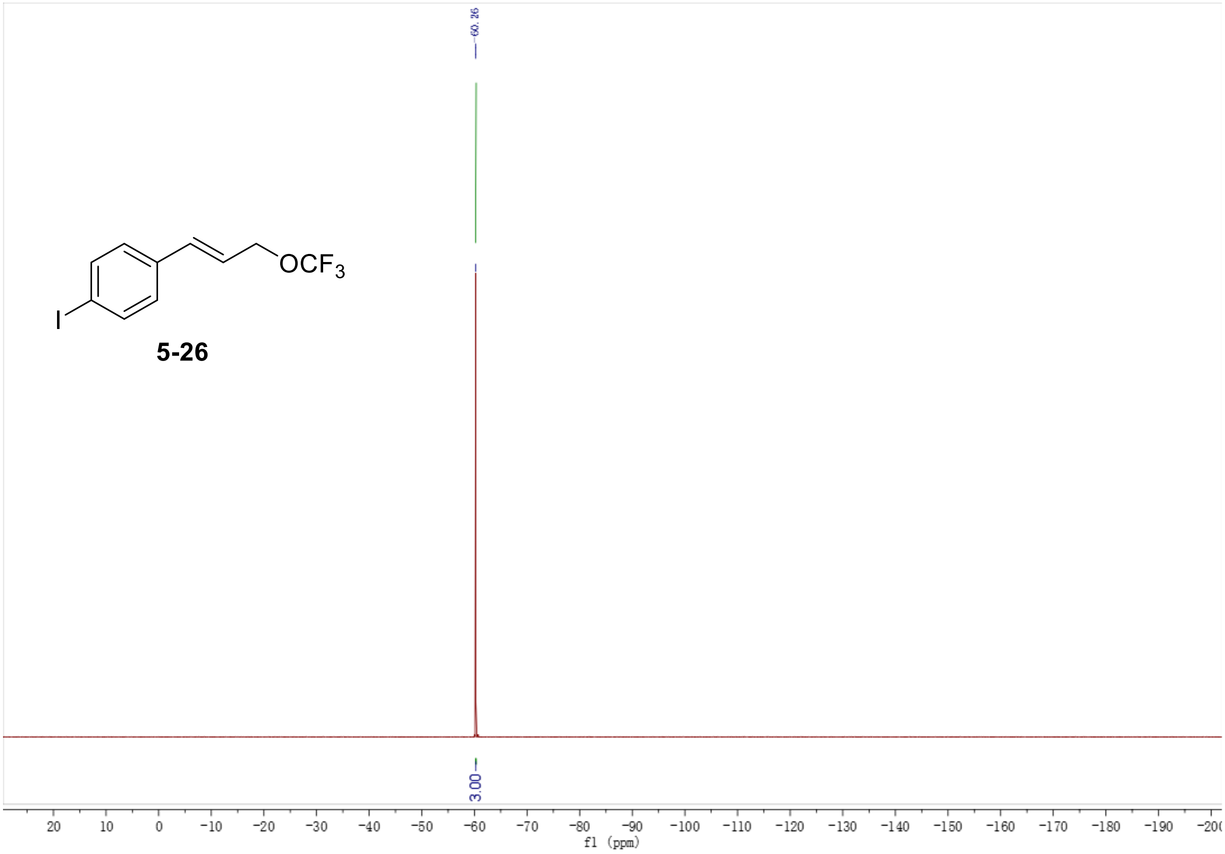

Supplementary Figure 96. ^{19}F NMR spectrum (376 MHz, $CDCl_3$) of **5-26**

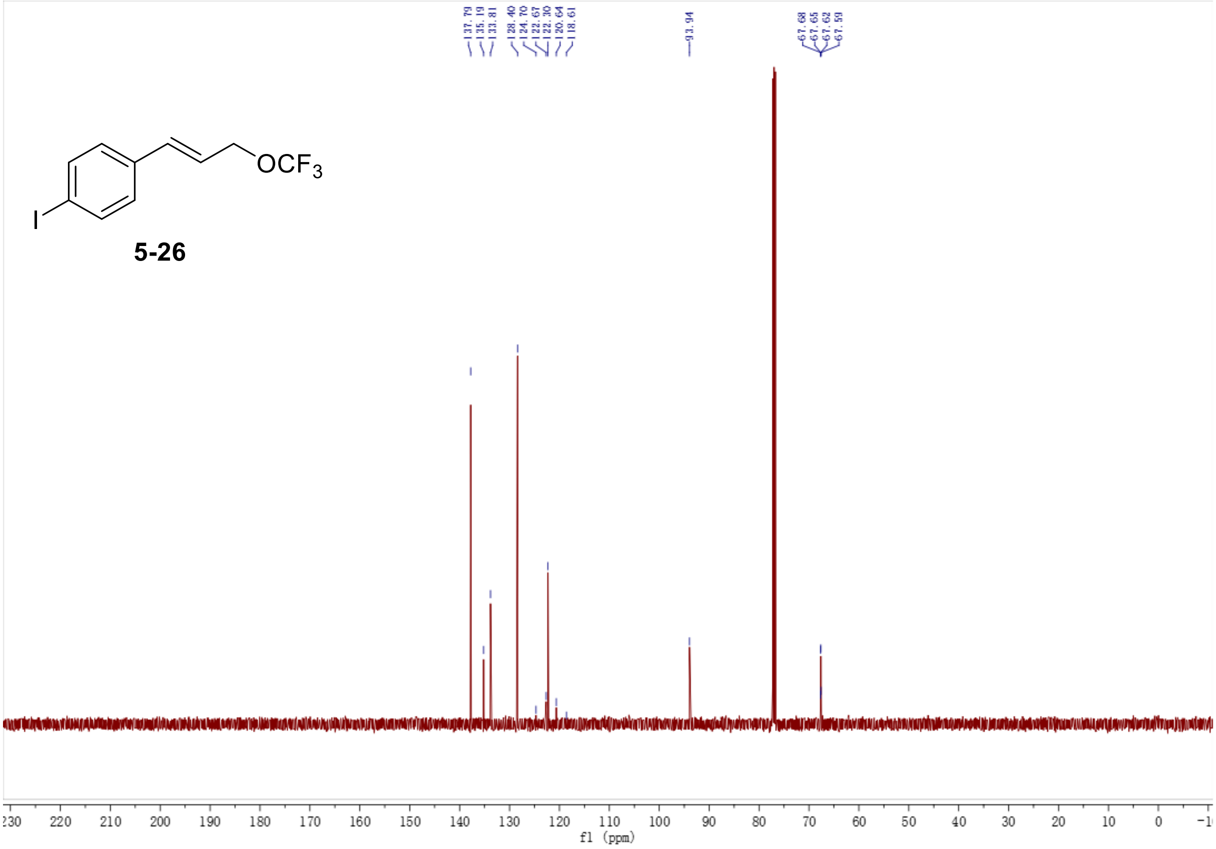

Supplementary Figure 97. ^{13}C NMR spectrum (101 MHz, $CDCl_3$) of **5-26**

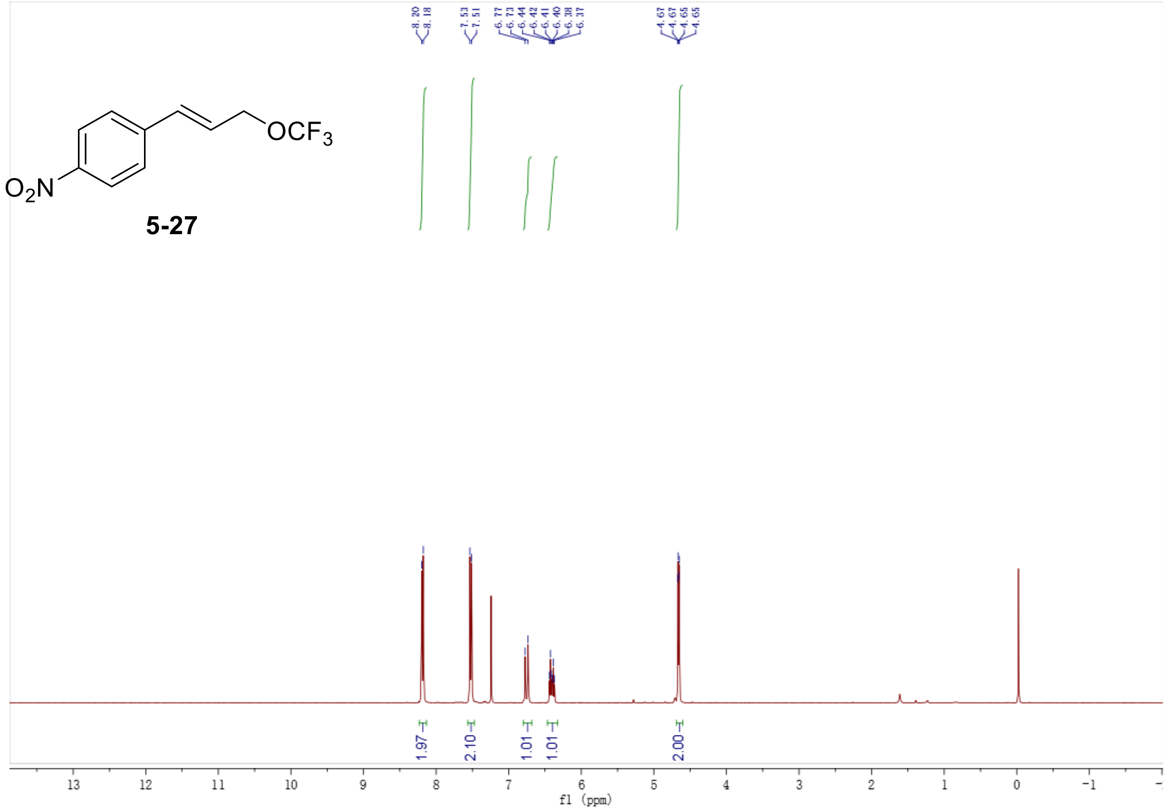

Supplementary Figure 98. ^{1}H NMR spectrum (400 MHz, $CDCl_3$) of **5-27**

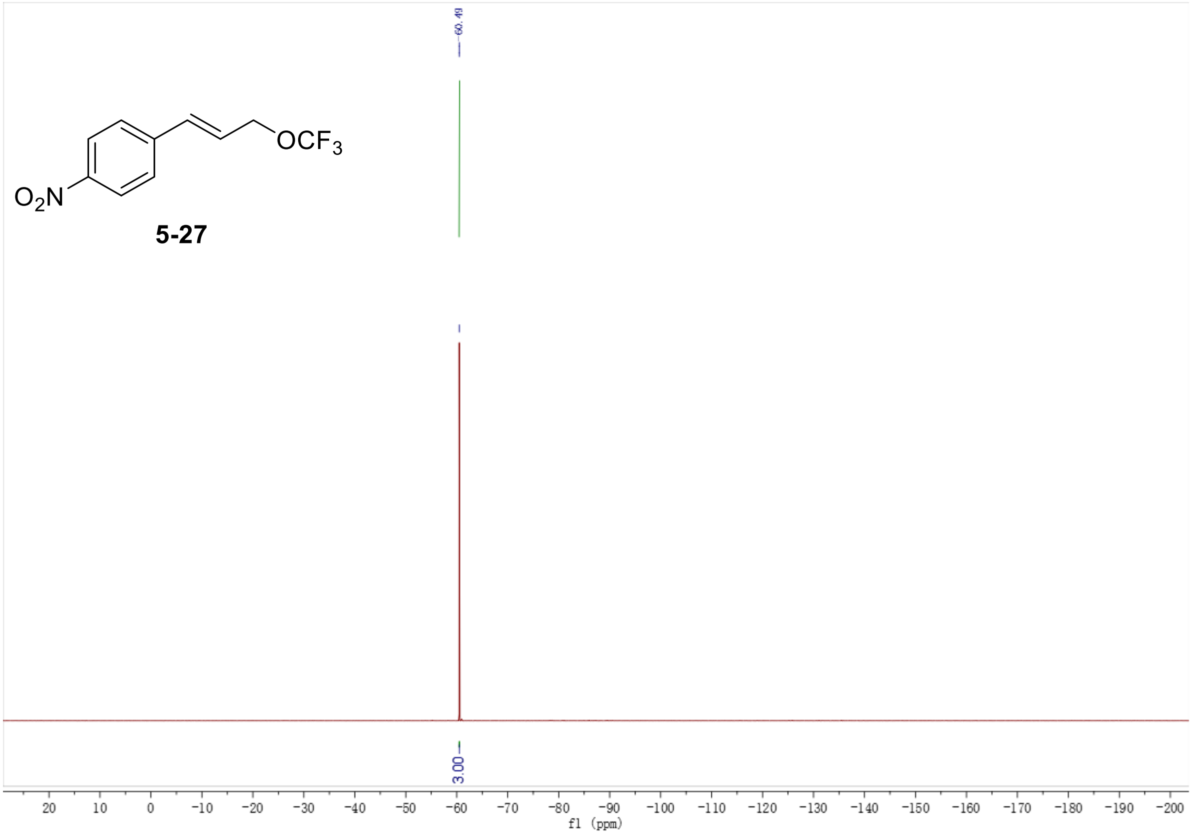

Supplementary Figure 99. ^{19}F NMR spectrum (376 MHz, $CDCl_3$) of **5-27**

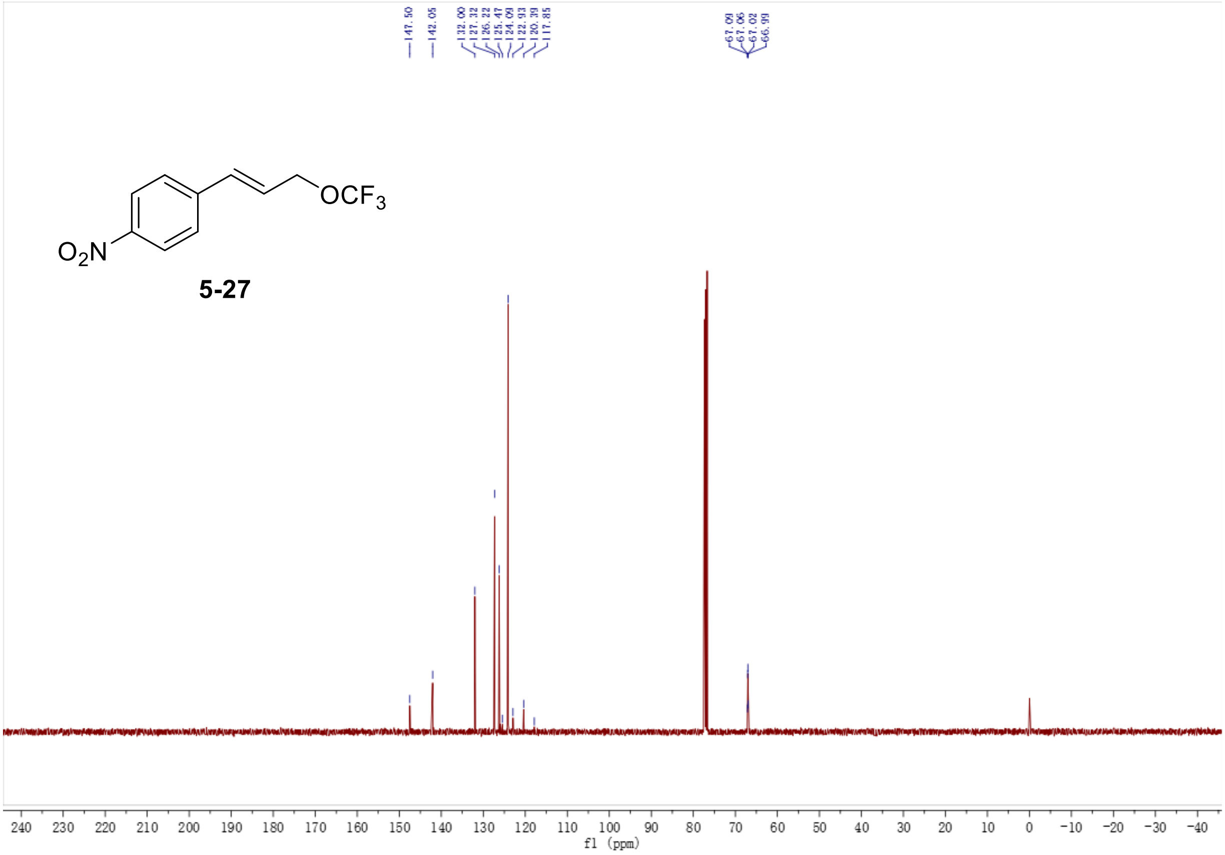

Supplementary Figure 100. ^{13}C NMR spectrum (101 MHz, $CDCl_3$) of **5-27**

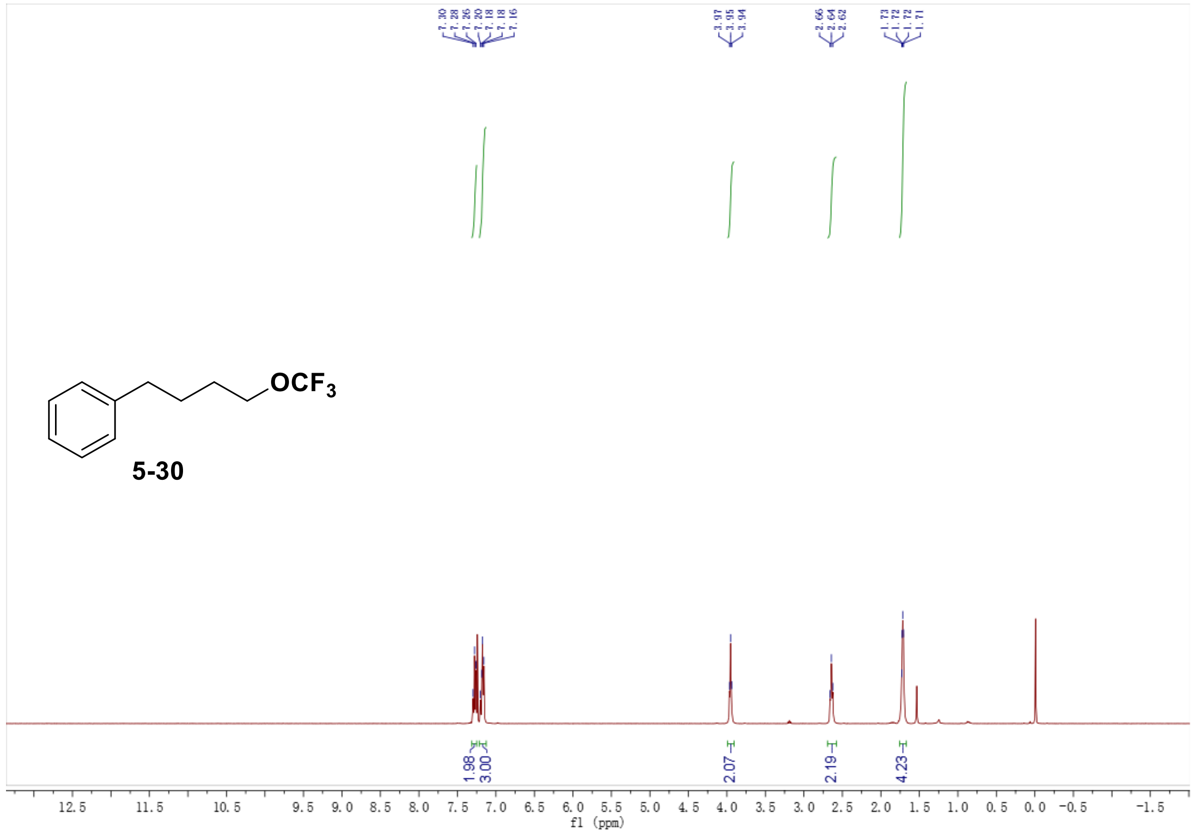

Supplementary Figure 101. ^{1}H NMR spectrum (400 MHz, $CDCl_3$) of **5-30**

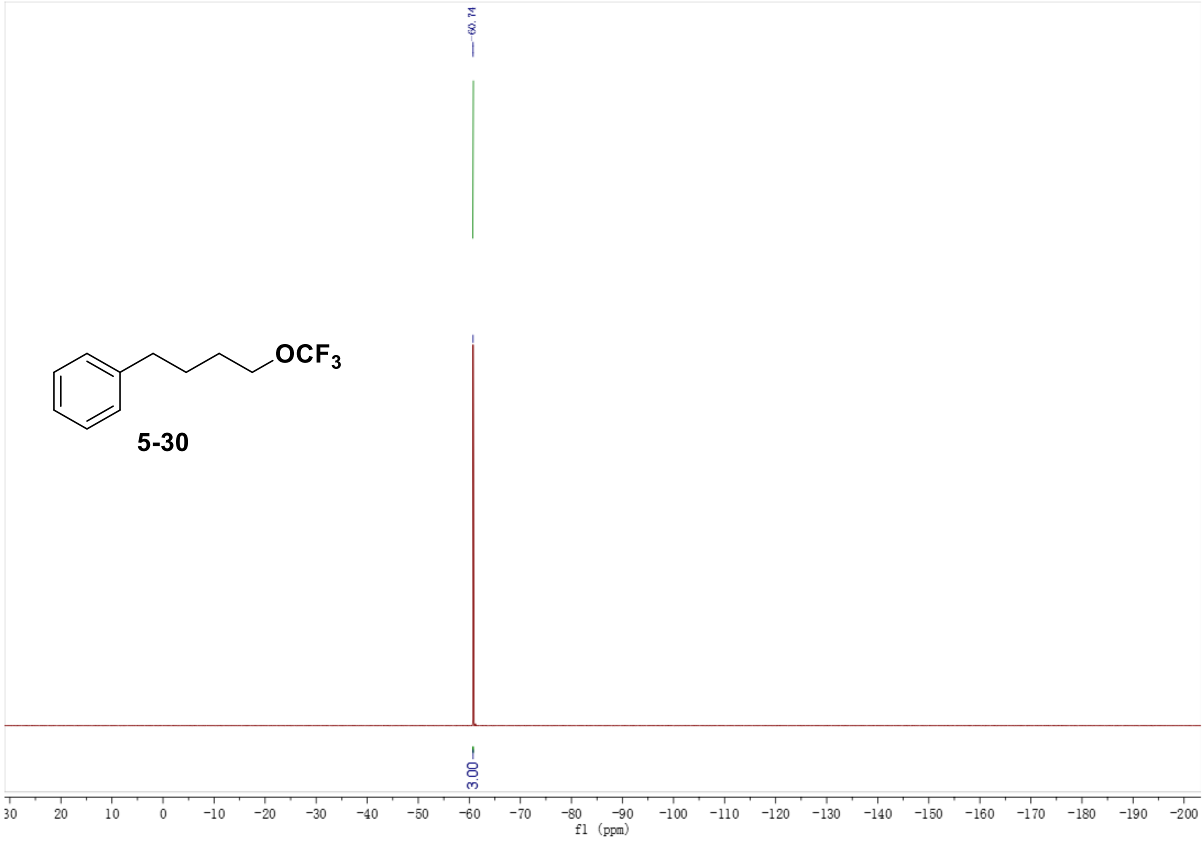

Supplementary Figure 102. ^{19}F NMR spectrum (376 MHz, $CDCl_3$) of **5-30**

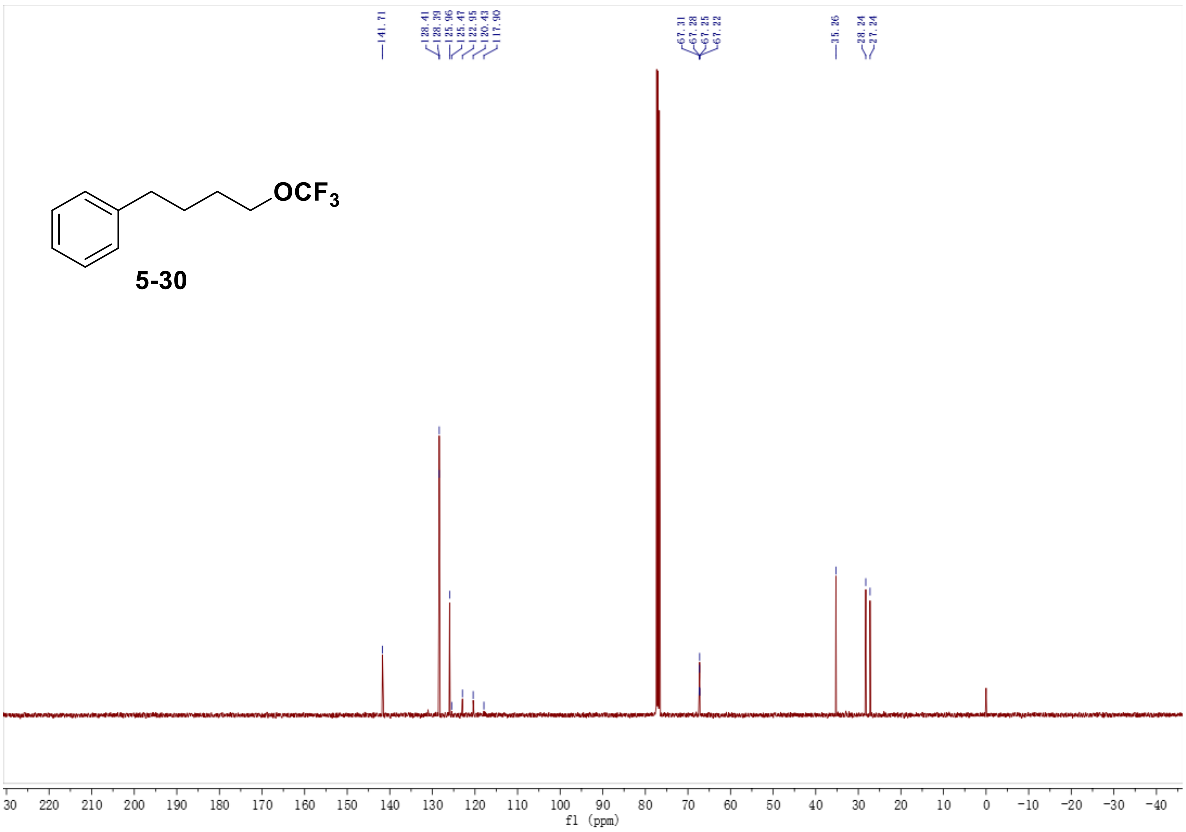

Supplementary Figure 103. ^{13}C NMR spectrum (101 MHz, $CDCl_3$) of **5-30**

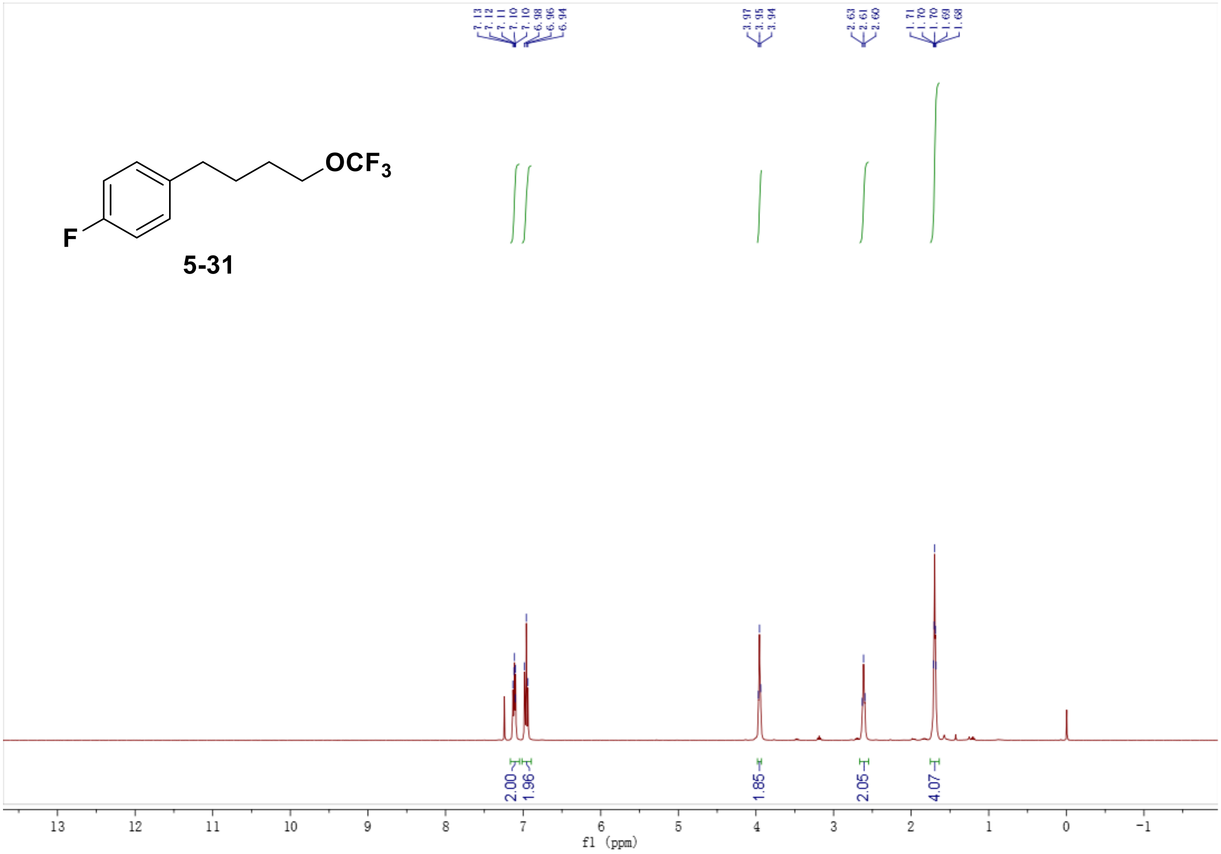

Supplementary Figure 104. ^{1}H NMR spectrum (400 MHz, $CDCl_3$) of **5-31**

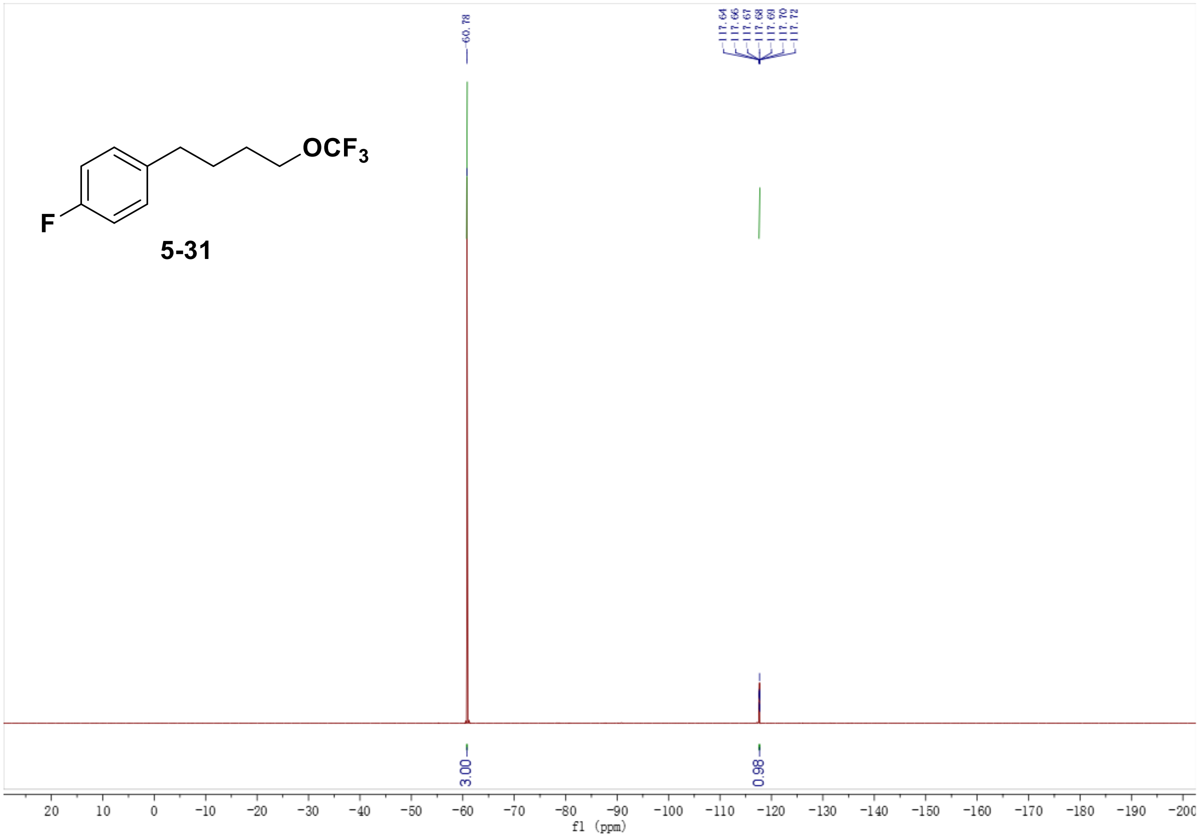

Supplementary Figure 105. ^{19}F NMR spectrum (376 MHz, $CDCl_3$) of **5-31**

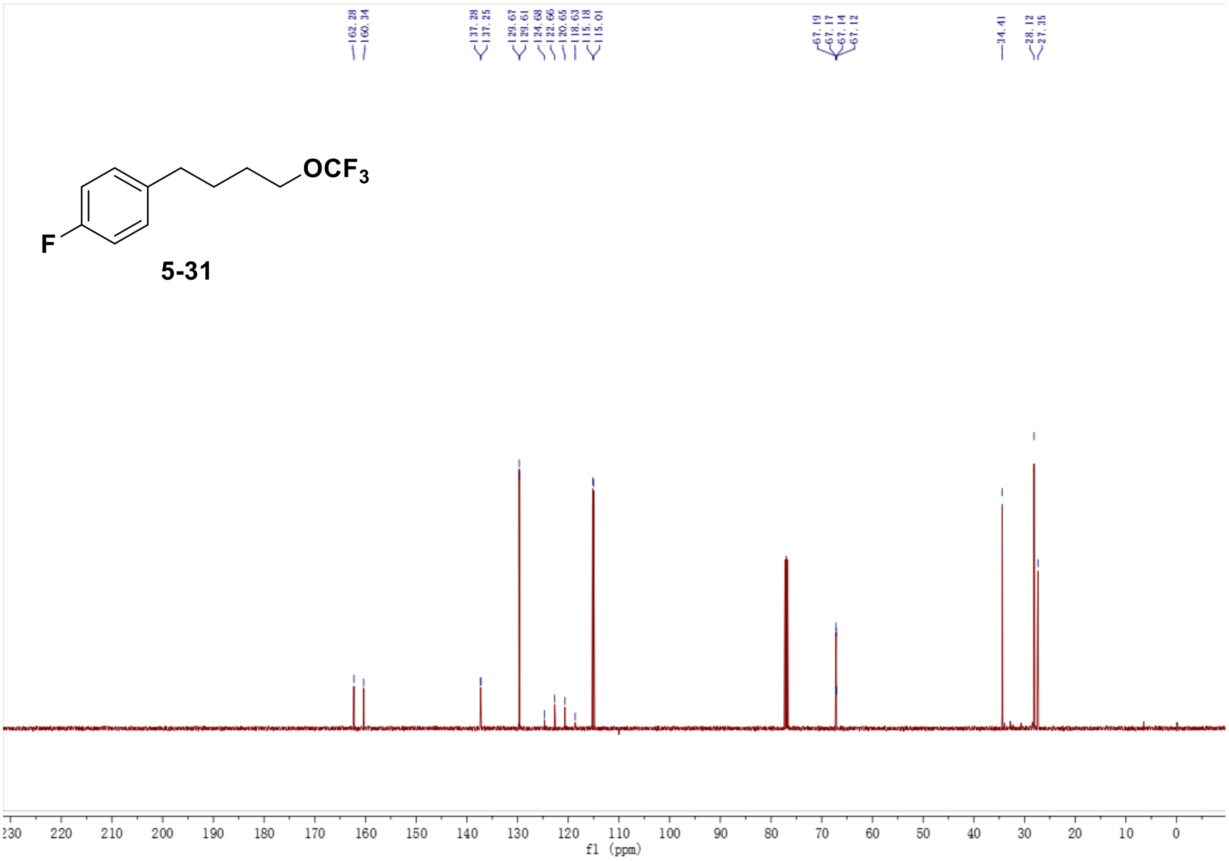

Supplementary Figure 106. ^{13}C NMR spectrum (101 MHz, $CDCl_3$) of **5-31**

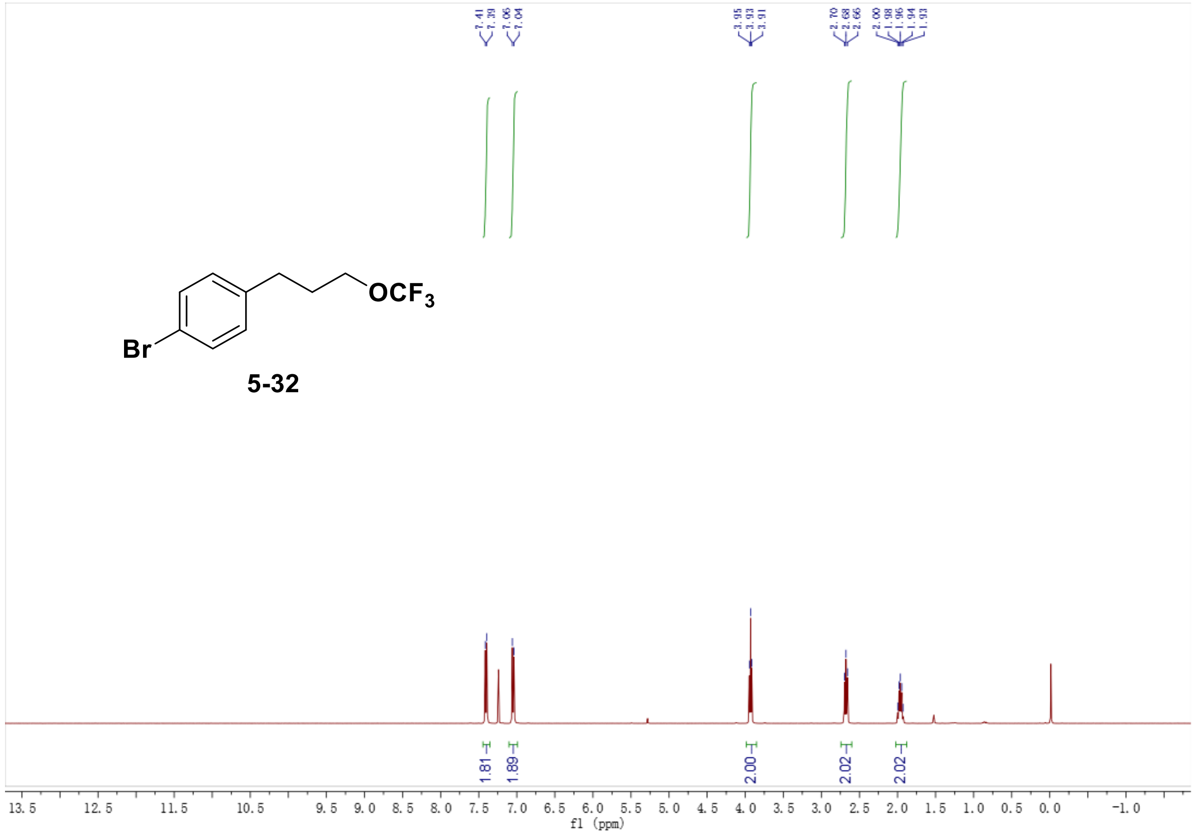

Supplementary Figure 107. ^{1}H NMR spectrum (400 MHz, $CDCl_3$) of **5-32**

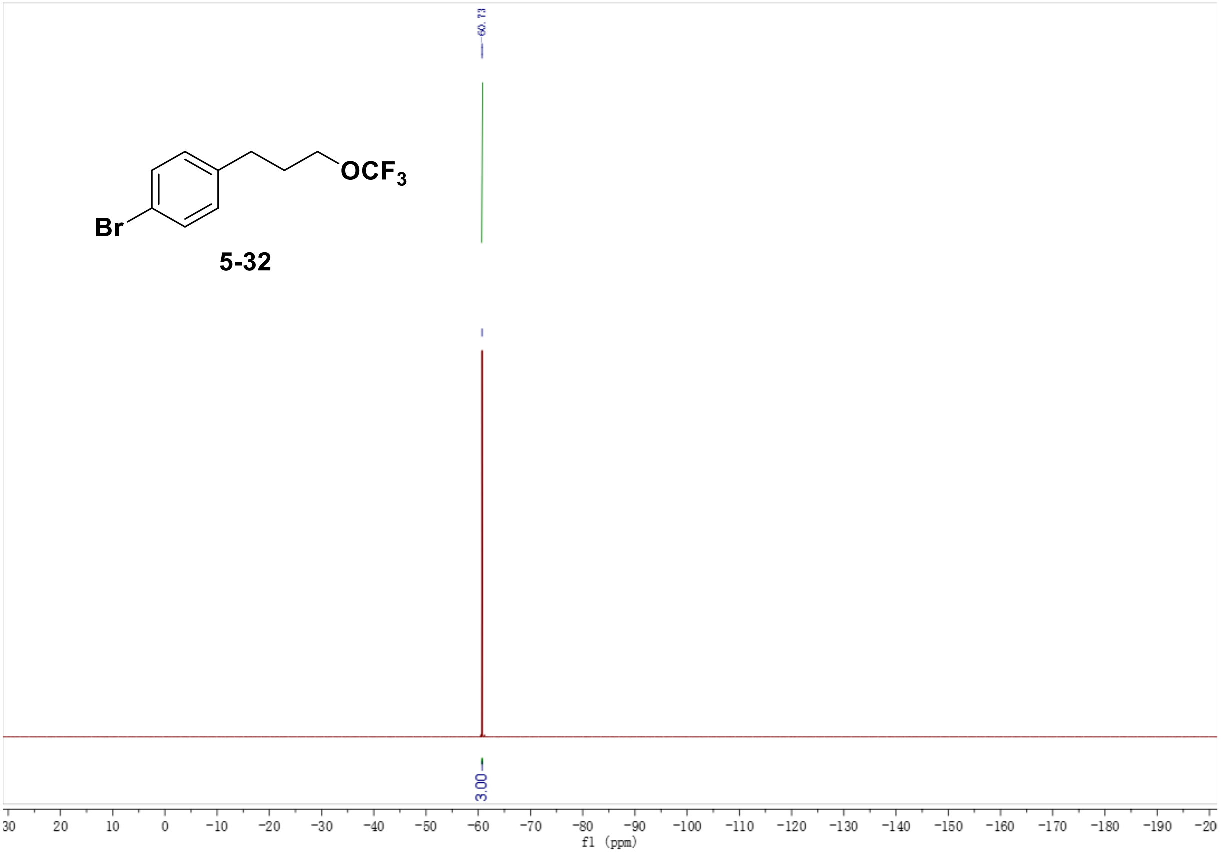

Supplementary Figure 108. ^{19}F NMR spectrum (376 MHz, $CDCl_3$) of **5-32**

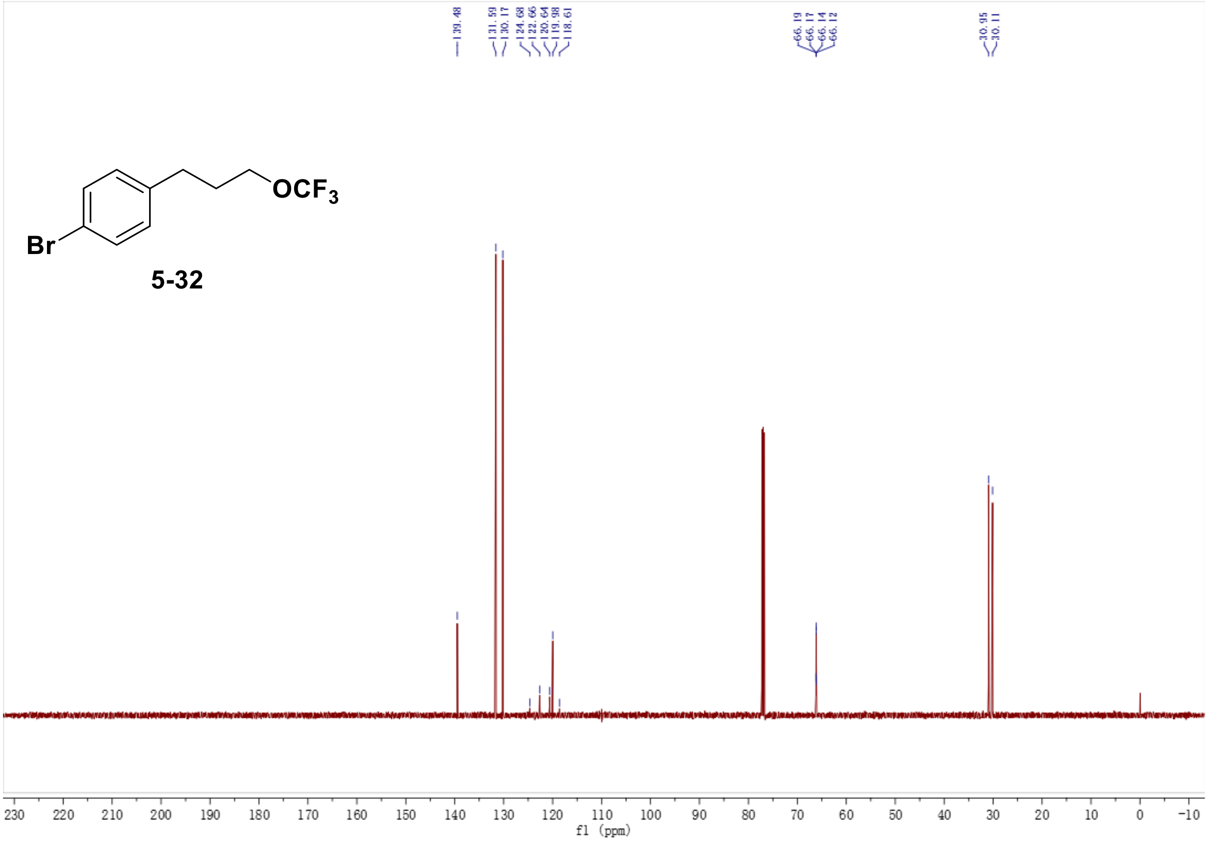

Supplementary Figure 109. ^{13}C NMR spectrum (101 MHz, $CDCl_3$) of **5-32**

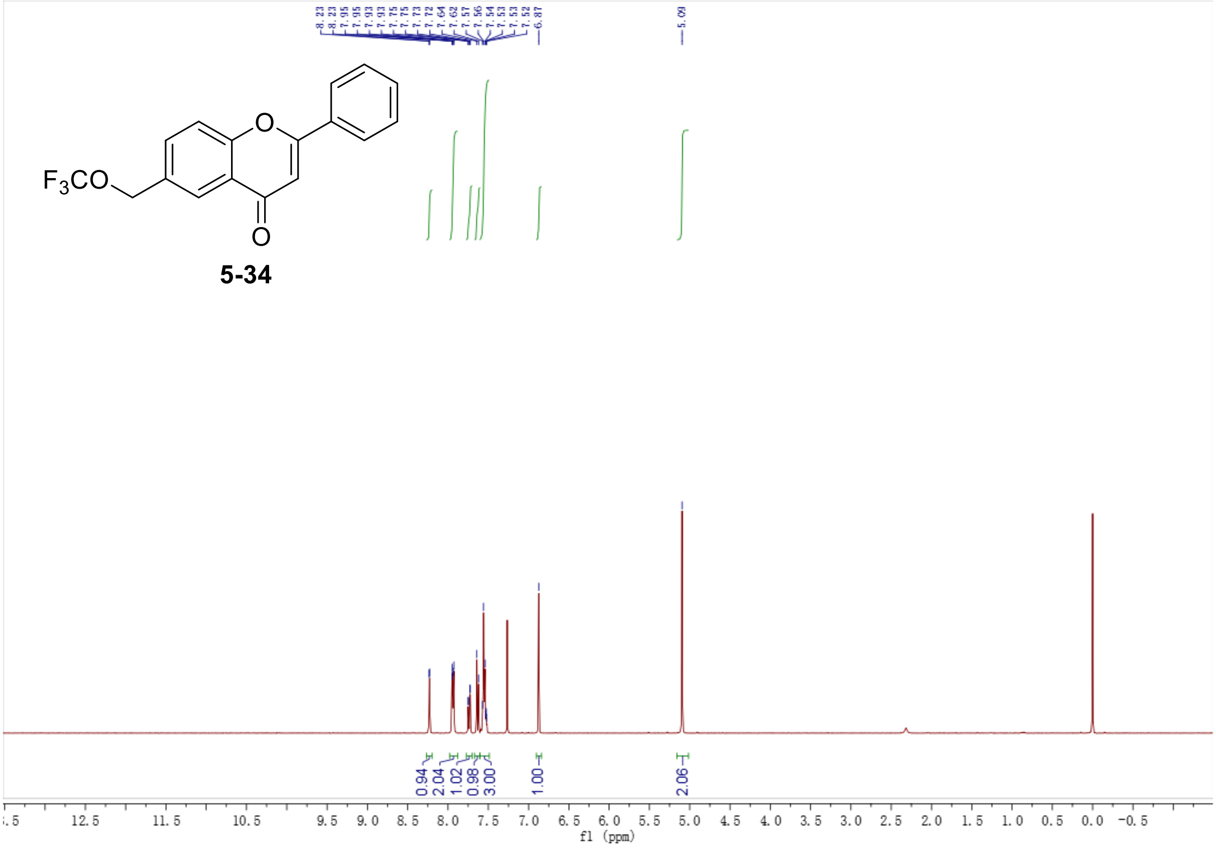

Supplementary Figure 110. ^{1}H NMR spectrum (400 MHz, $CDCl_3$) of **5-34**

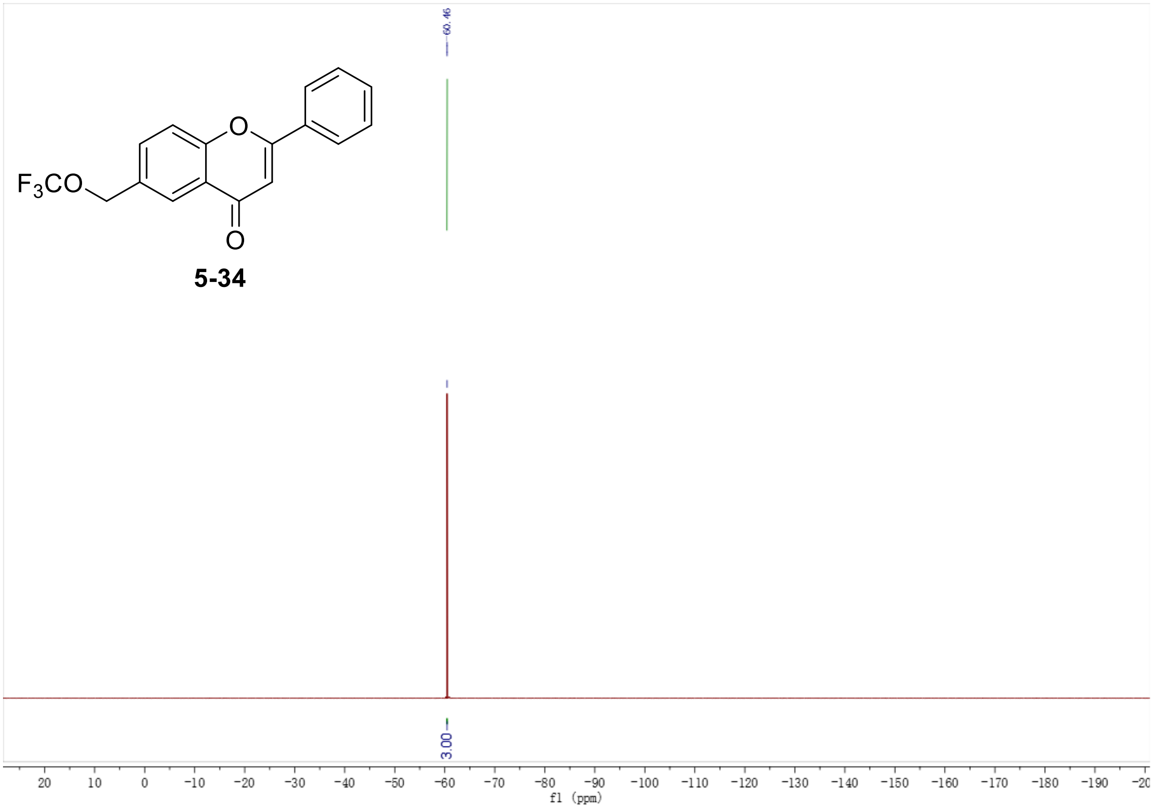

Supplementary Figure 111. ^{19}F NMR spectrum (376 MHz, $CDCl_3$) of **5-34**

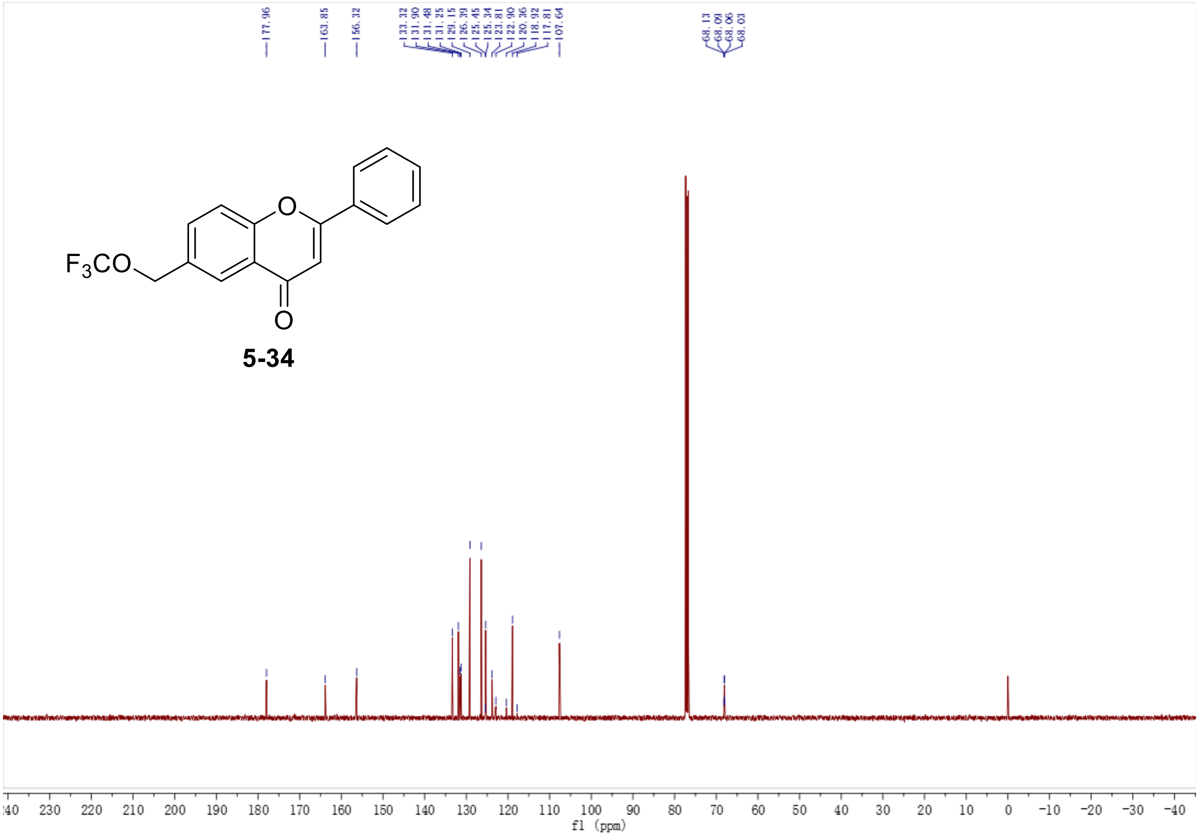

Supplementary Figure 112. ^{13}C NMR spectrum (101 MHz, $CDCl_3$) of **5-34**

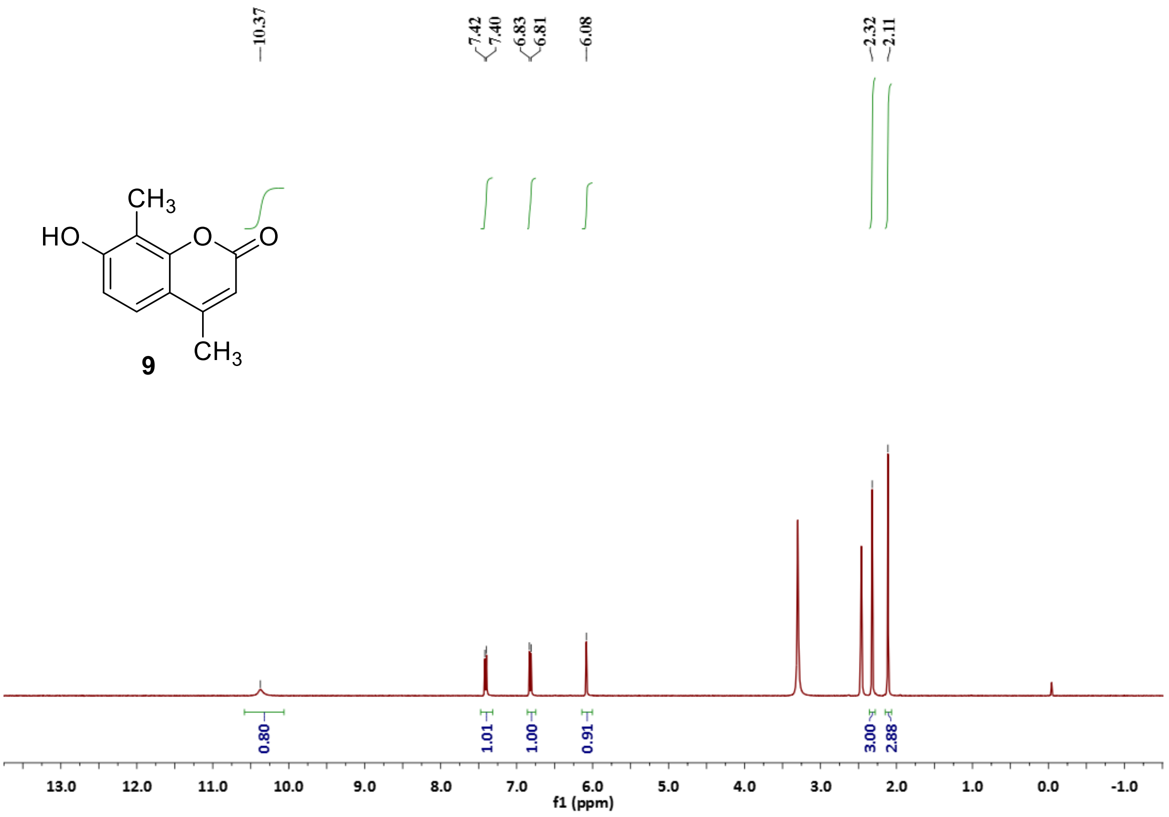

Supplementary Figure 113. ^{1}H NMR spectrum (400 MHz, DMSO) of **9**

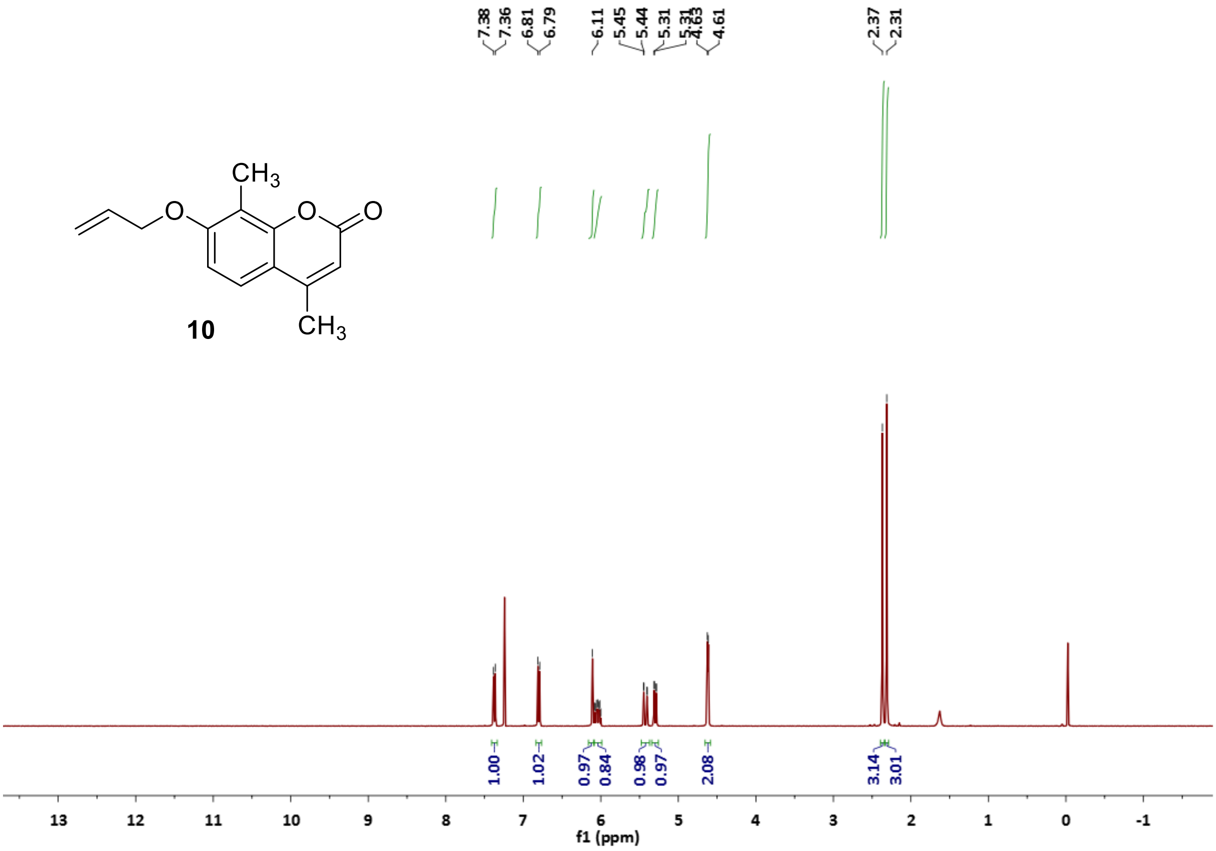

Supplementary Figure 114. ^{1}H NMR spectrum (400 MHz, $CDCl_3$) of **10**

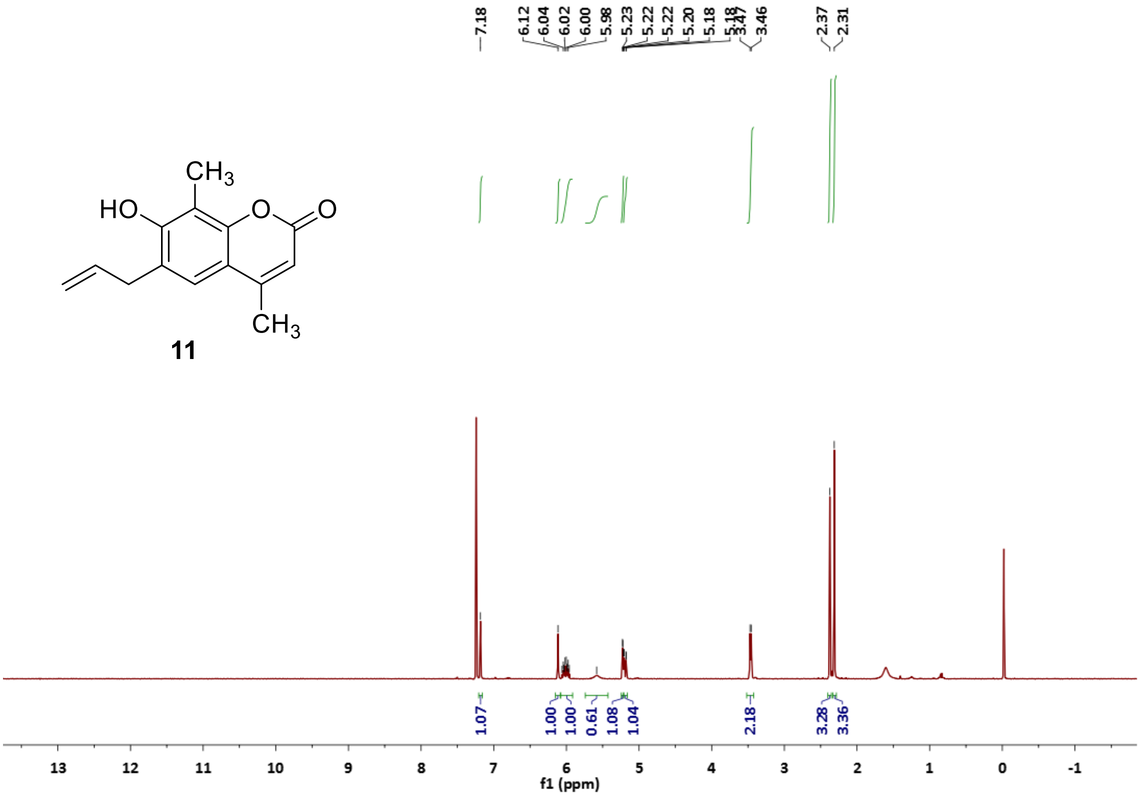

Supplementary Figure 115. ^{1}H NMR spectrum (400 MHz, $CDCl_3$) of **11**

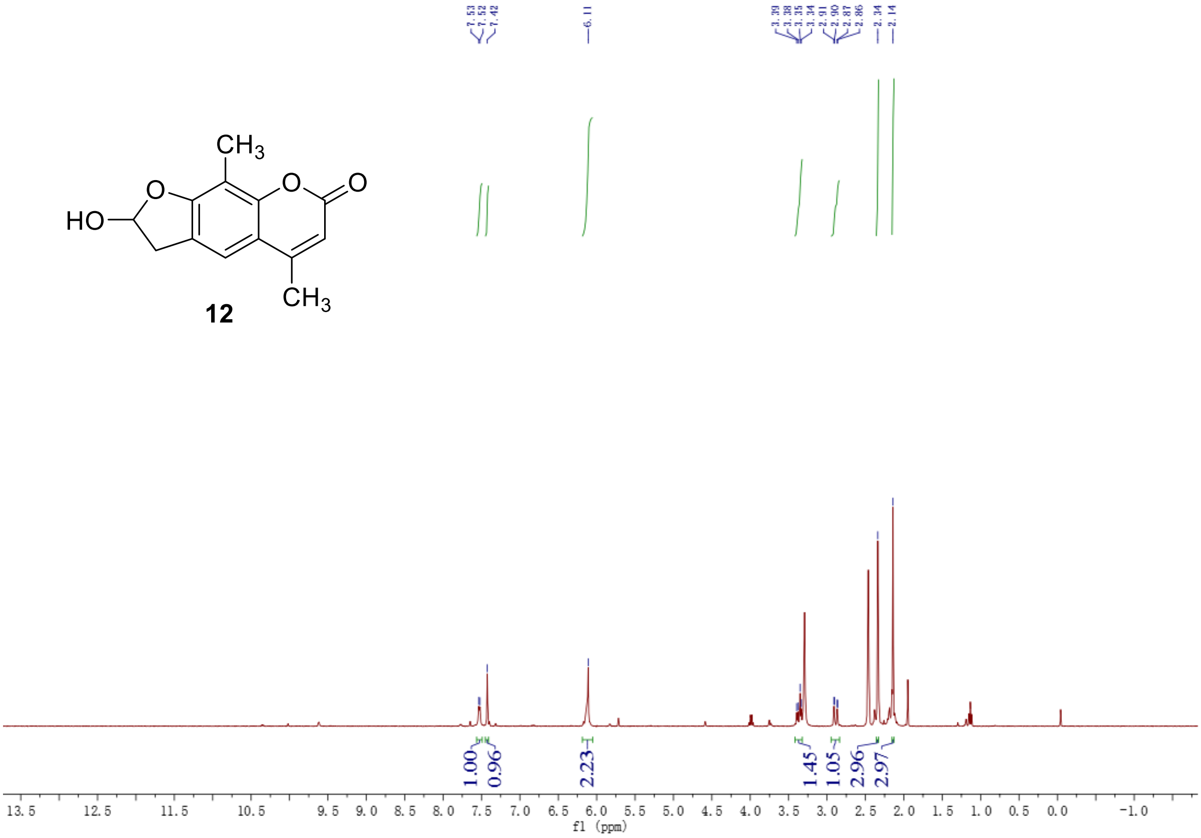

Supplementary Figure 116. ^{1}H NMR spectrum (400 MHz, DMSO) of **12**

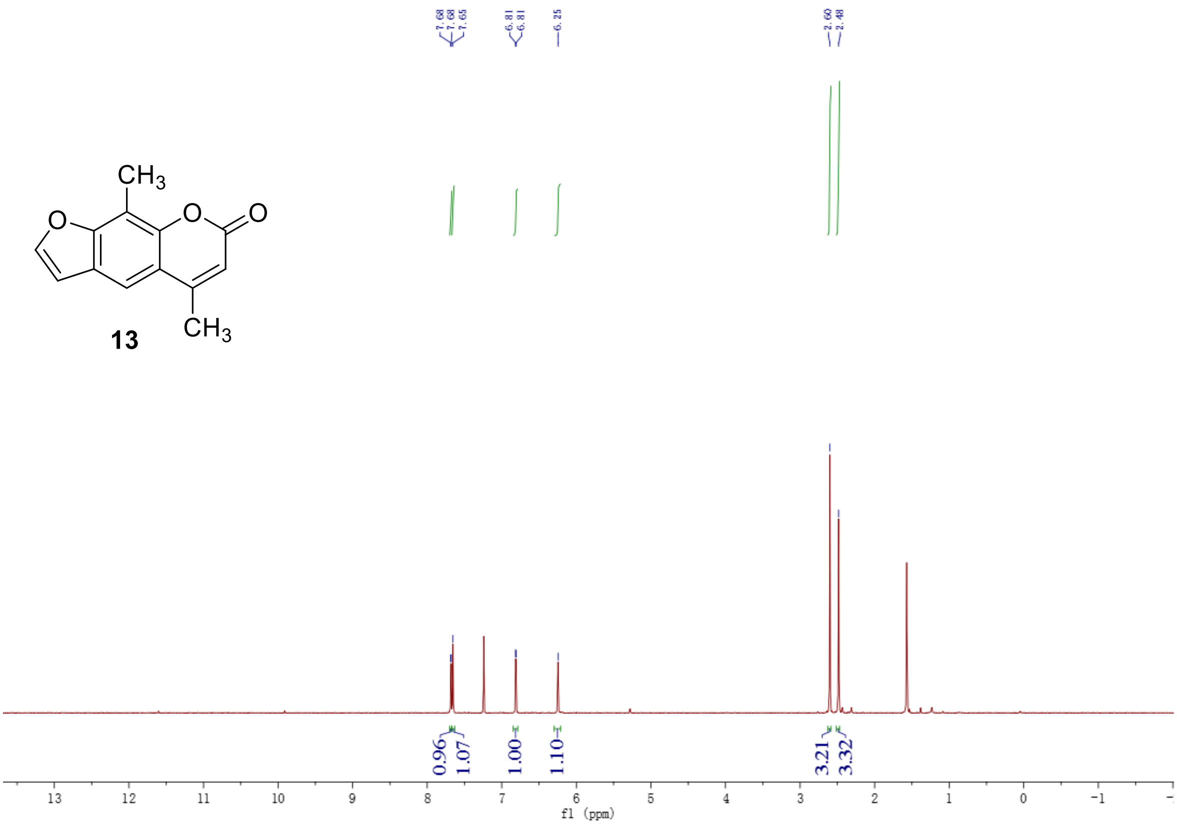

Supplementary Figure 117. ^{1}H NMR spectrum (400 MHz, $CDCl_3$) of **13**

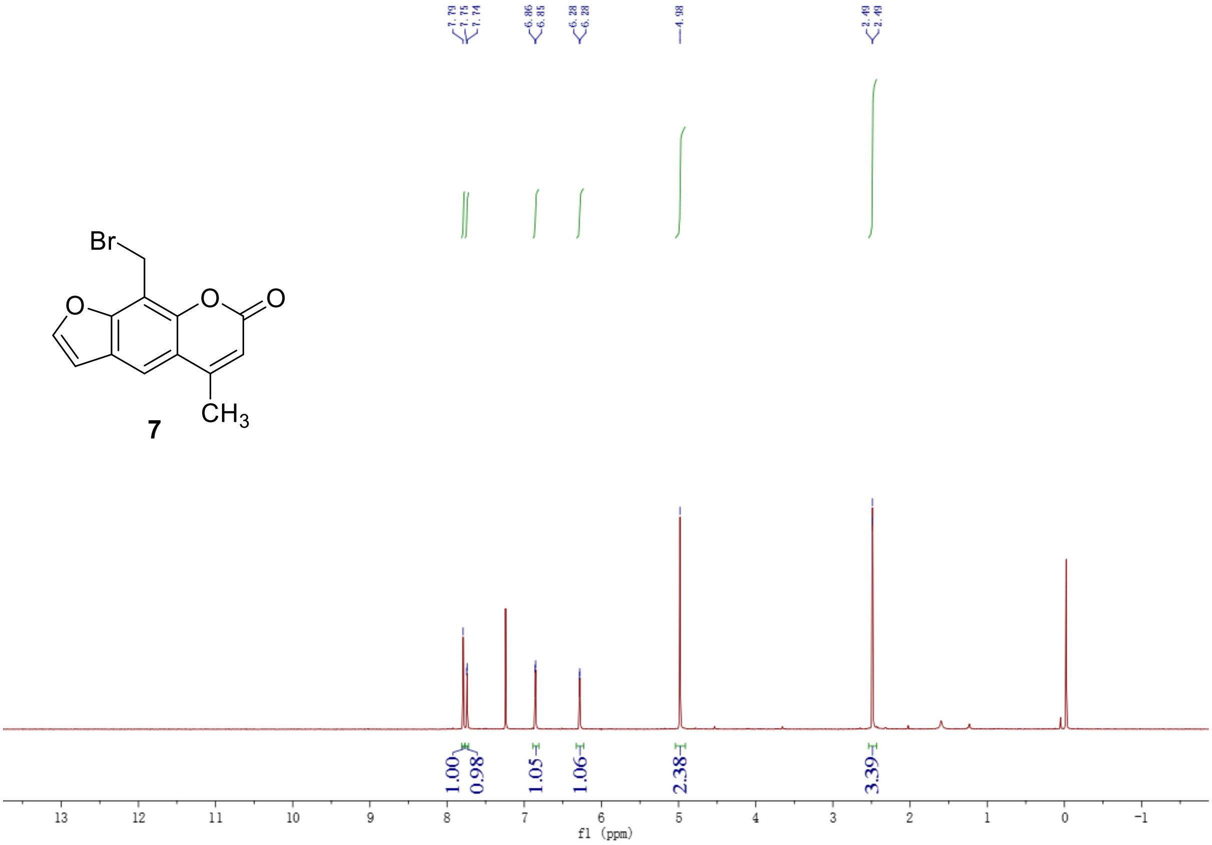

Supplementary Figure 118. ^{1}H NMR spectrum (400 MHz, $CDCl_3$) of **7**

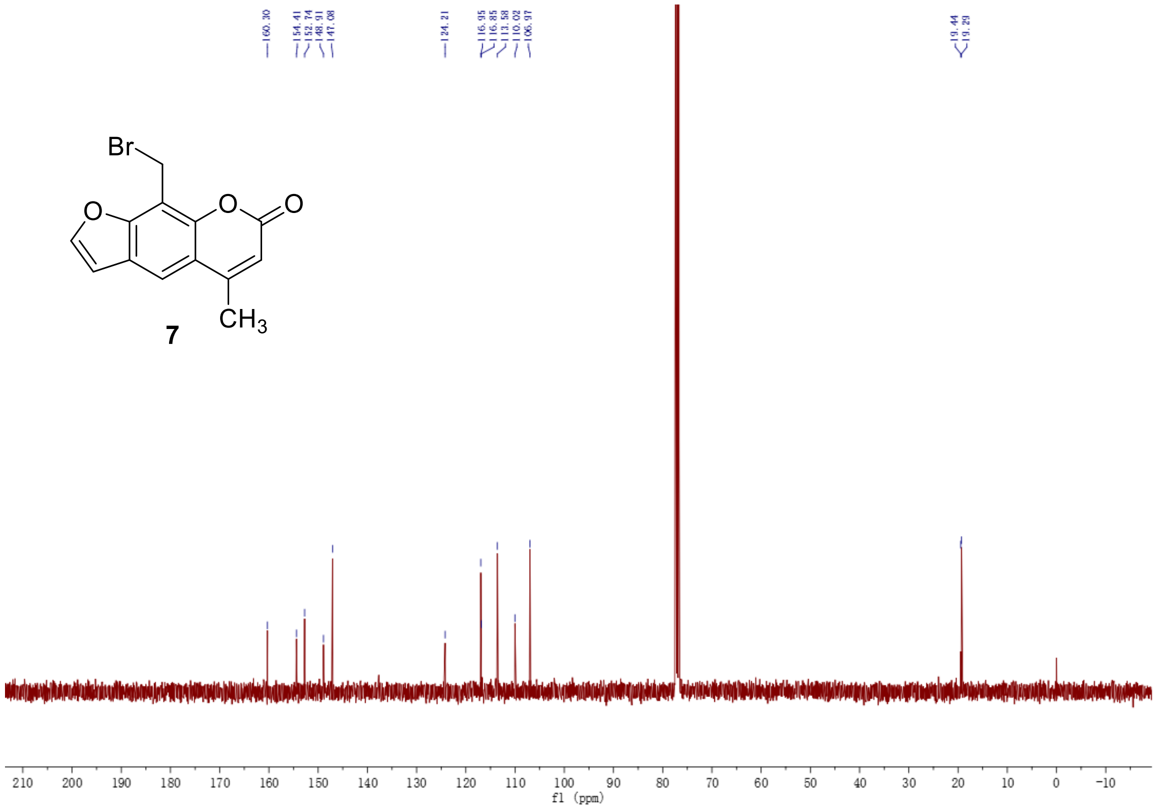

Supplementary Figure 119. ^{13}C NMR spectrum (101 MHz, $CDCl_3$) of **7**

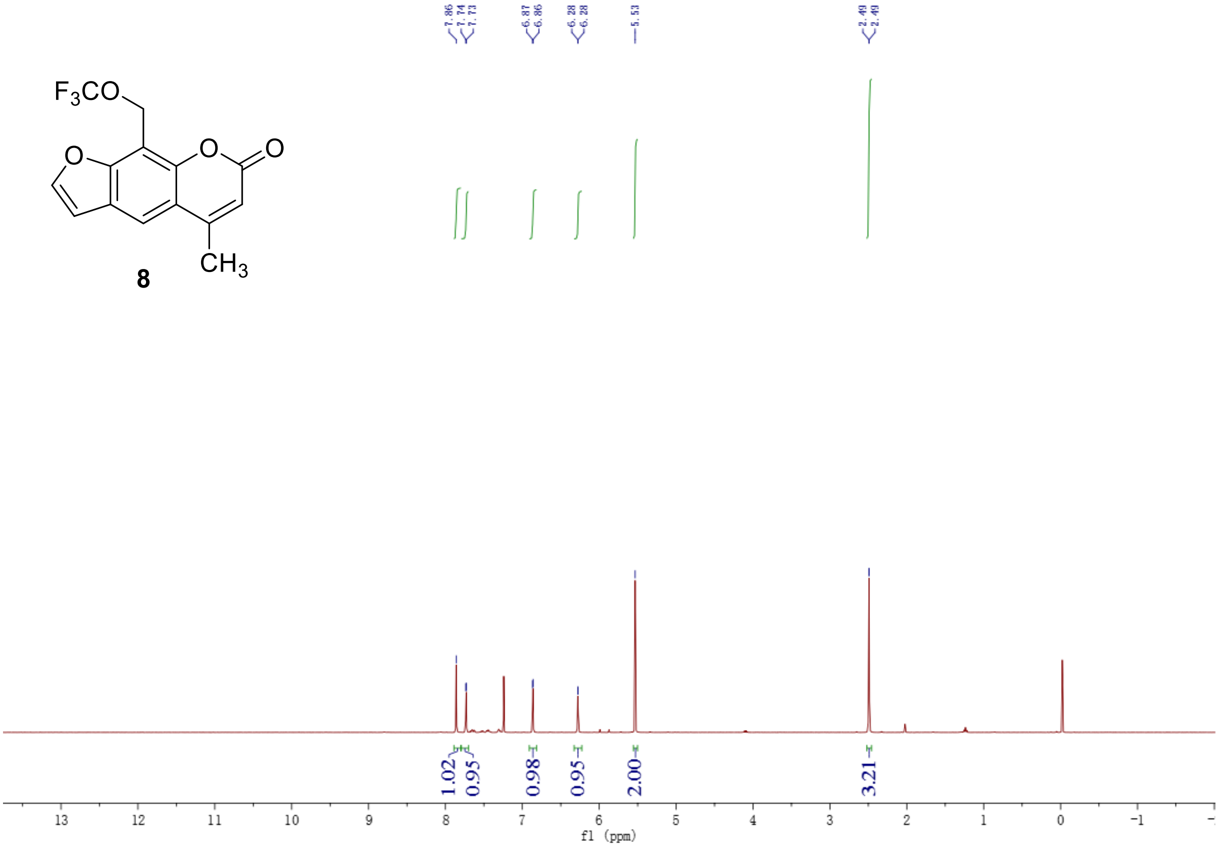

Supplementary Figure 120. ^{1}H NMR spectrum (400 MHz, $CDCl_3$) of **8**

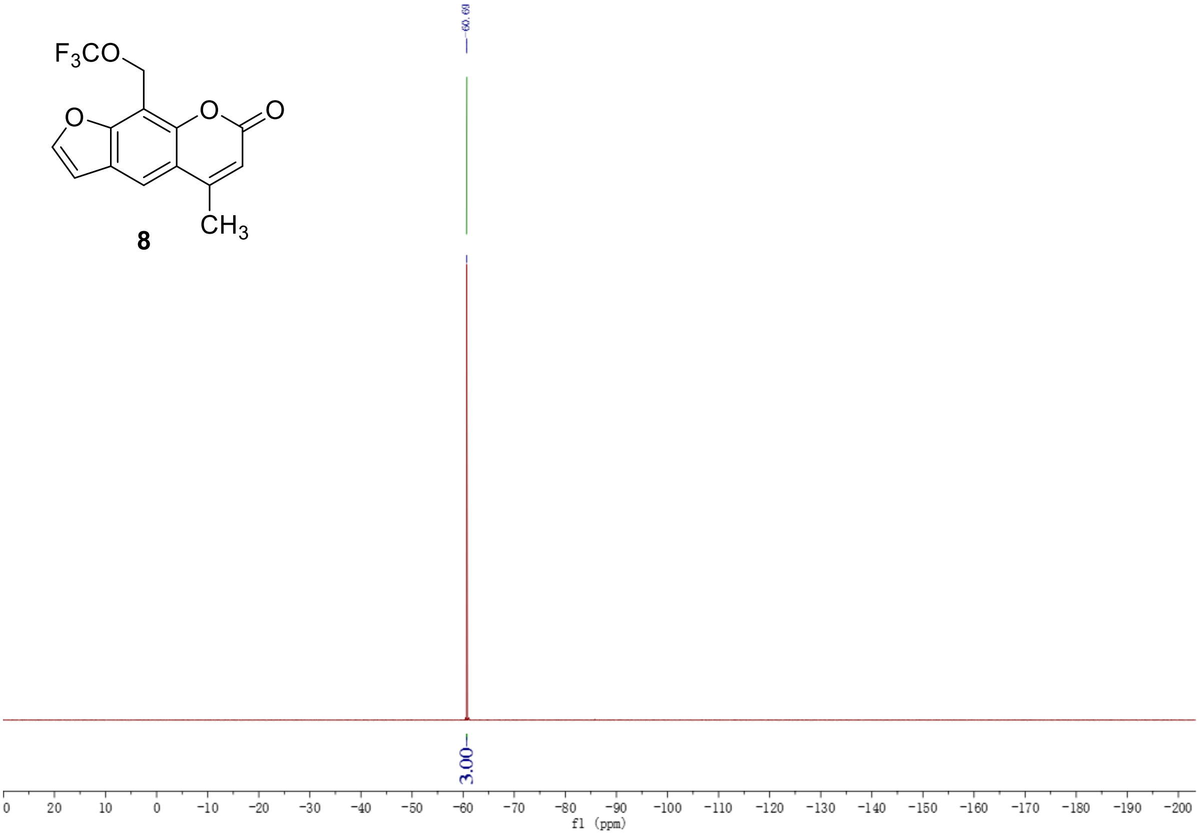

Supplementary Figure 121. ^{19}F NMR spectrum (376 MHz, $CDCl_3$) of **8**

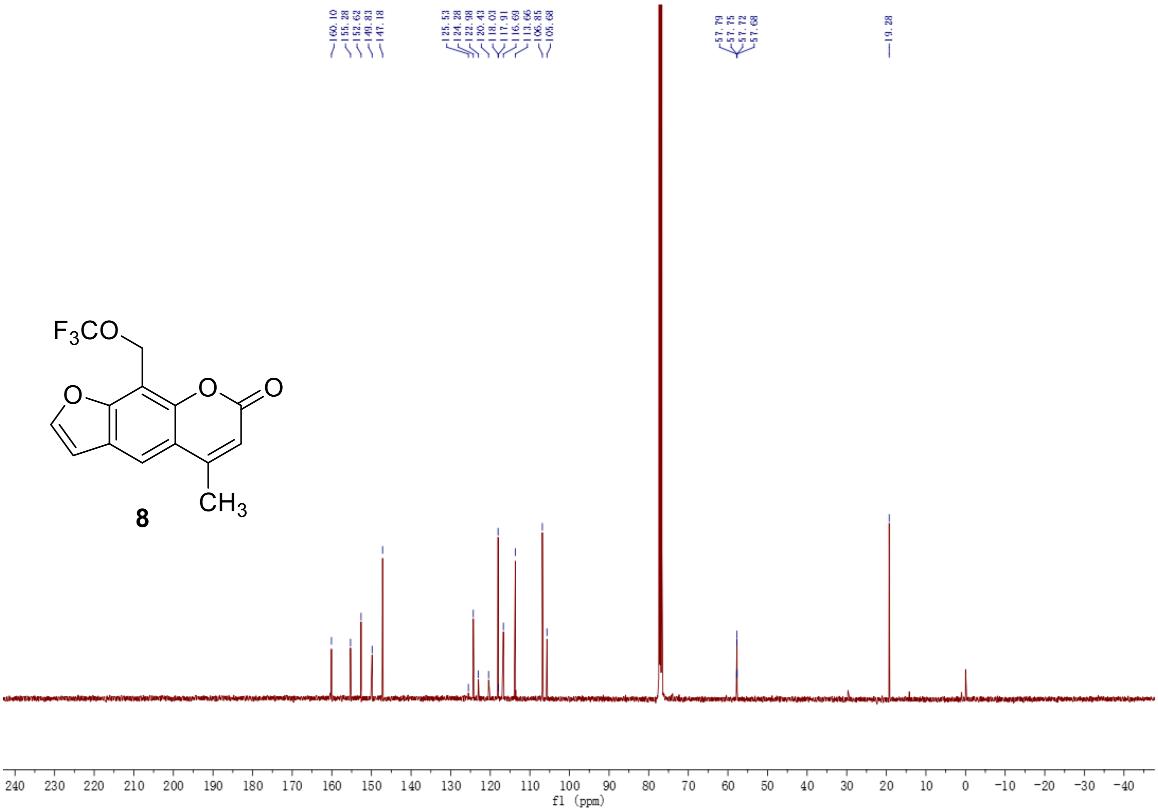

Supplementary Figure 122. ^{13}C NMR spectrum (101 MHz, $CDCl_3$) of **8**

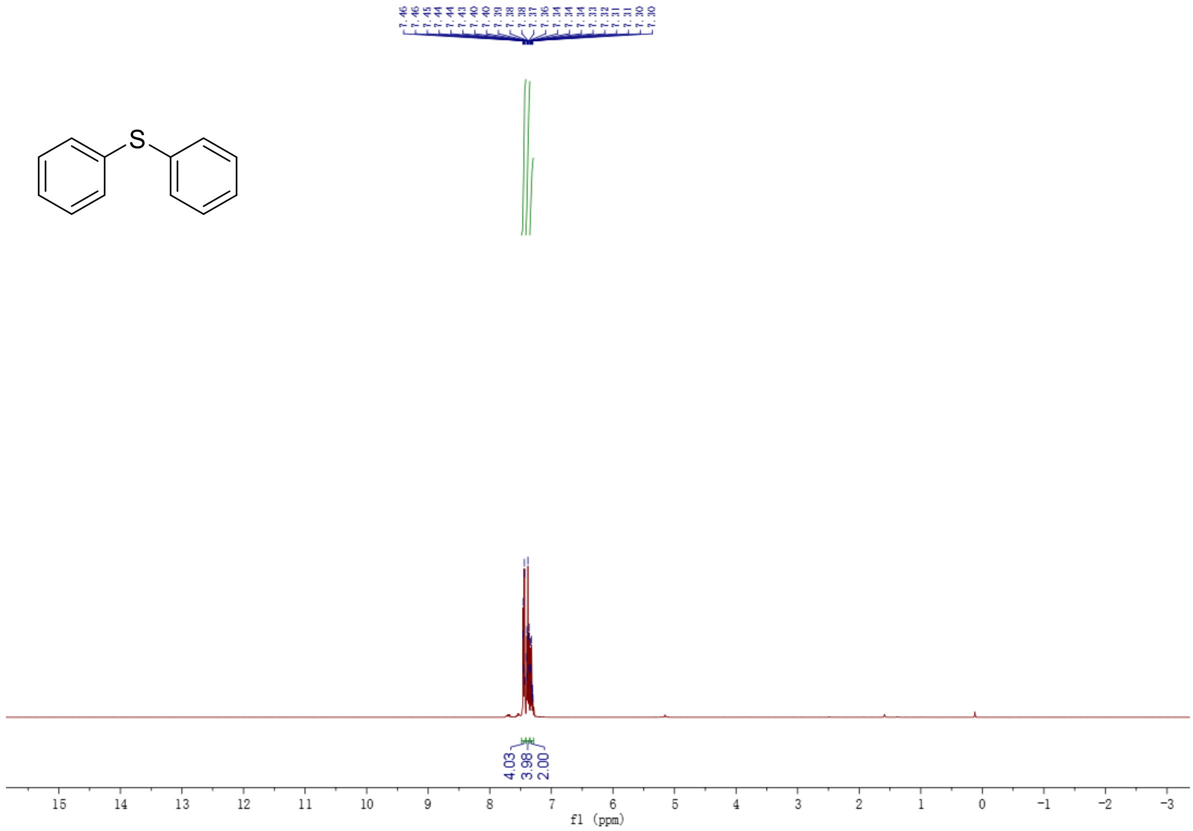

Supplementary Figure 123. ^{1}H NMR spectrum (400 MHz, $CDCl_3$) of **Ph_2S**

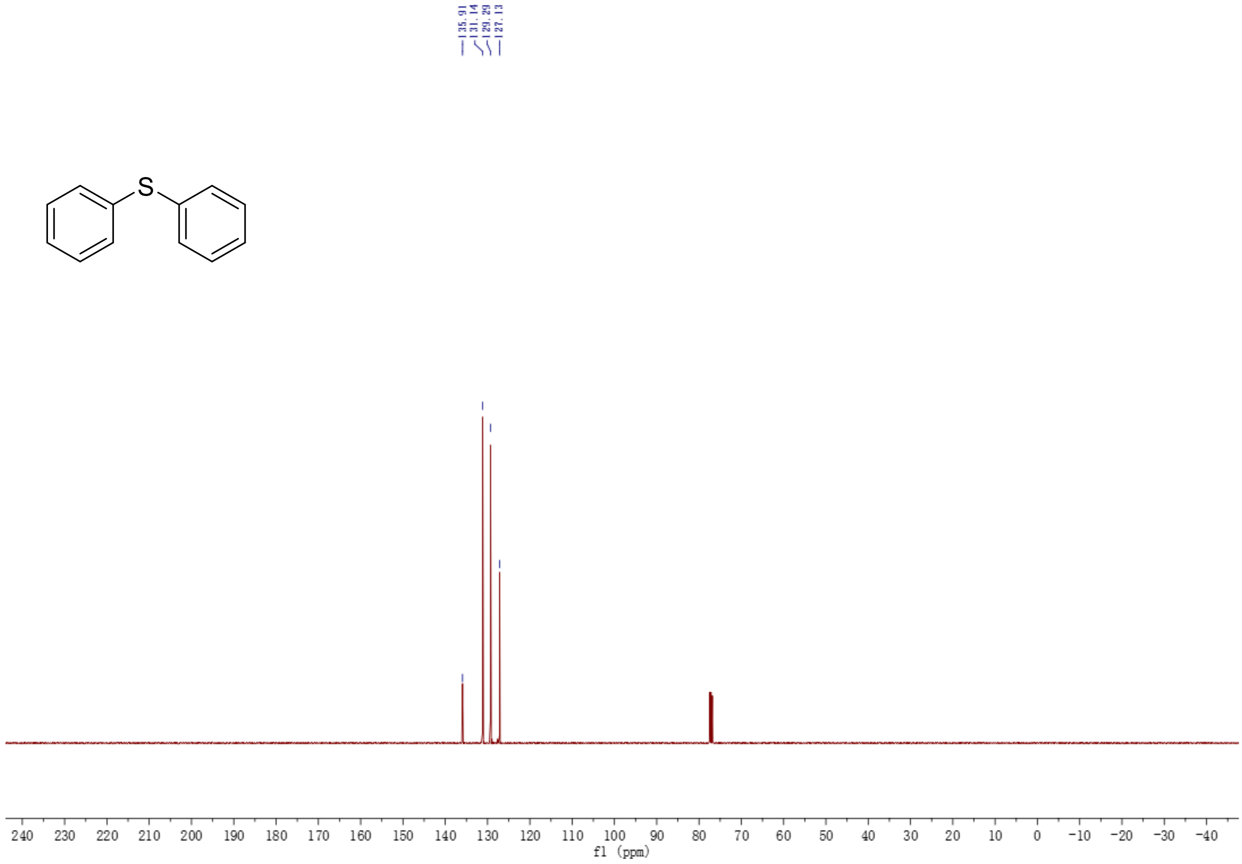

Supplementary Figure 124. ^{13}C NMR spectrum (101 MHz, $CDCl_3$) of **Ph_2S**

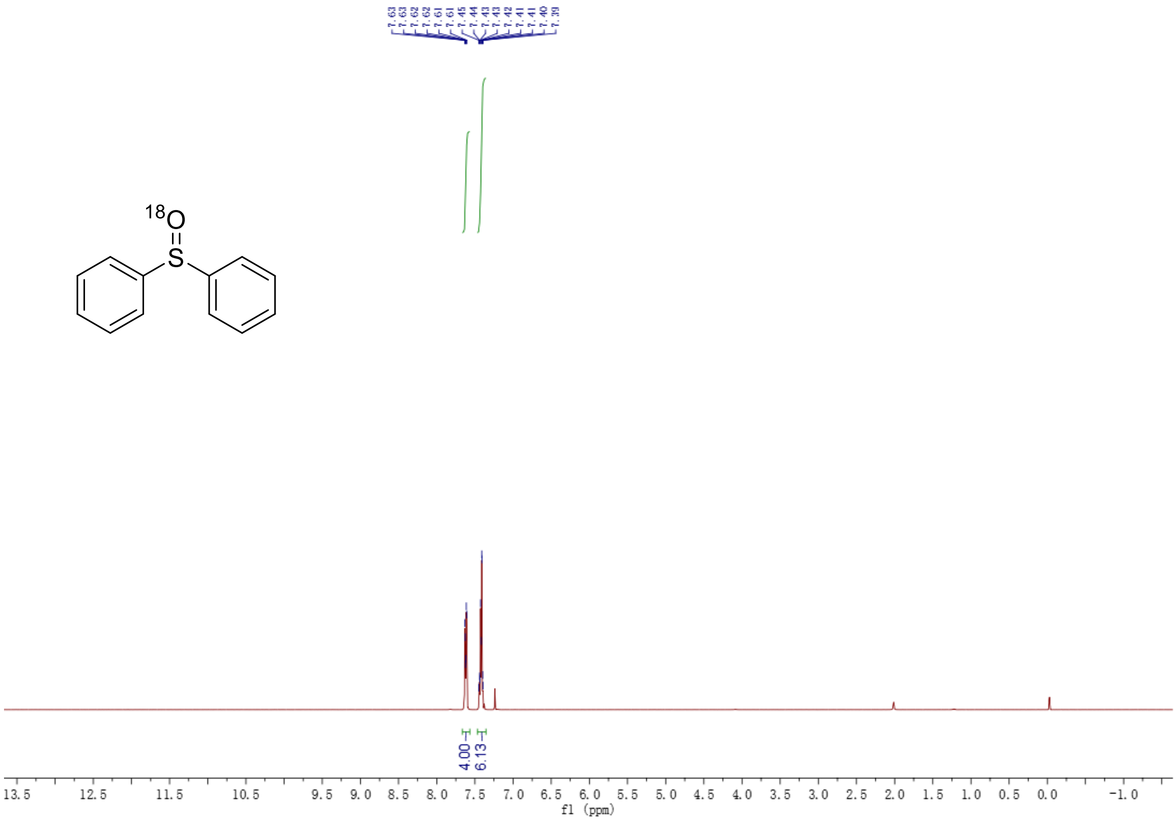

Supplementary Figure 125. ^{1}H NMR spectrum (400 MHz, $CDCl_3$) of **$Ph_2S{=}O^{18}$**

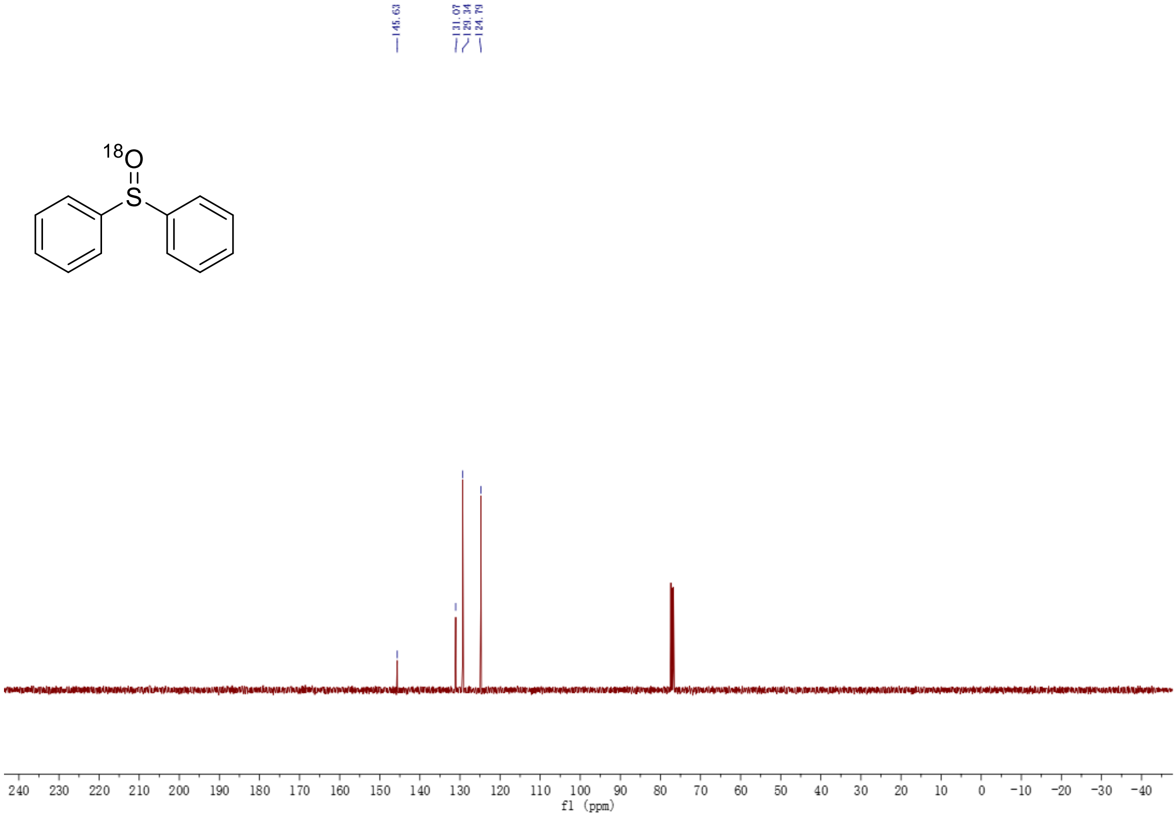

Supplementary Figure 126. ^{13}C NMR spectrum (101 MHz, $CDCl_3$) of **$Ph_2S{=}O^{18}$**

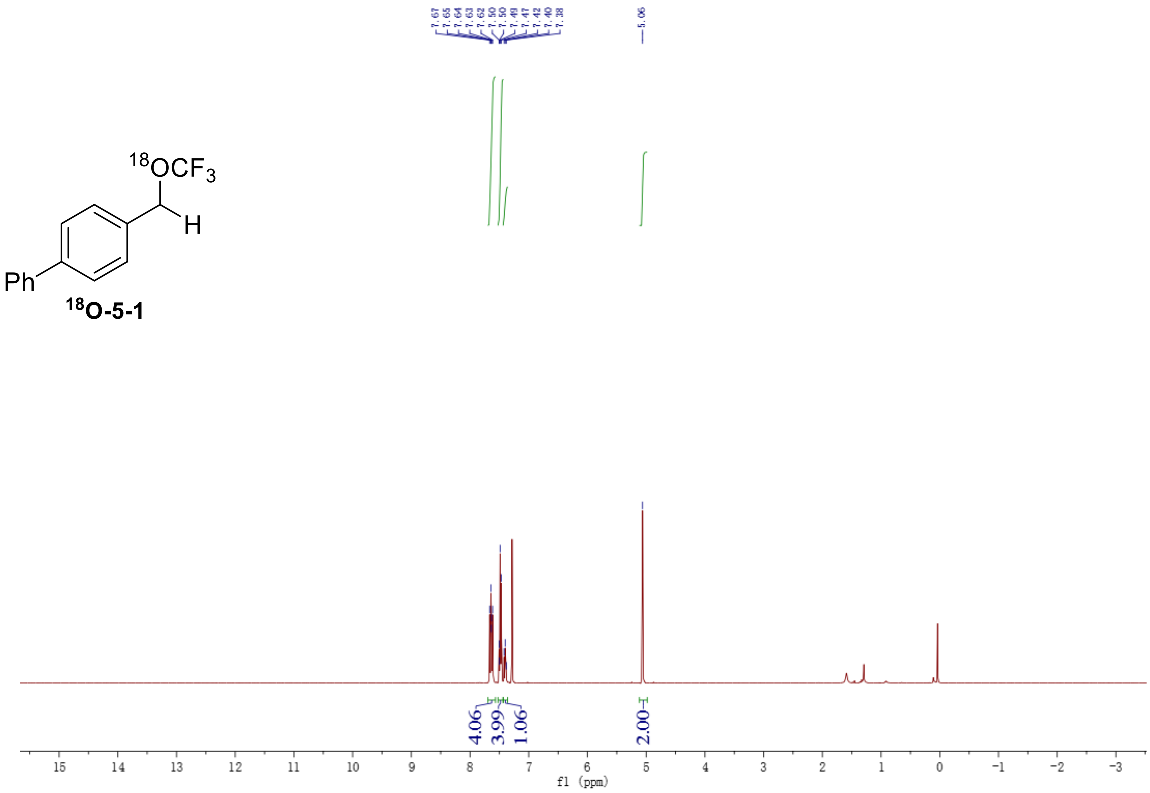

Supplementary Figure 127. ^{1}H NMR spectrum (400 MHz, $CDCl_3$) of **^{18}O-5-1**

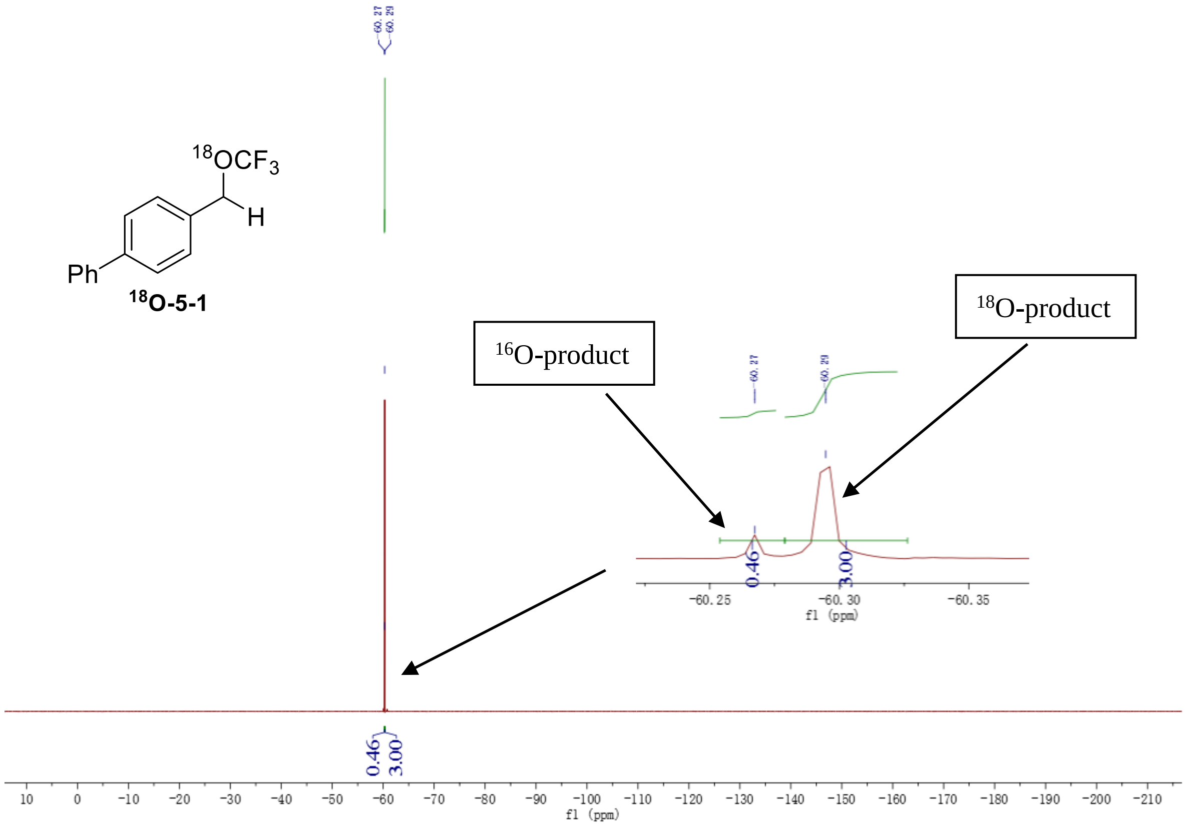

Supplementary Figure 128. ^{19}F NMR spectrum (376 MHz, $CDCl_3$) of **^{18}O-5-1**

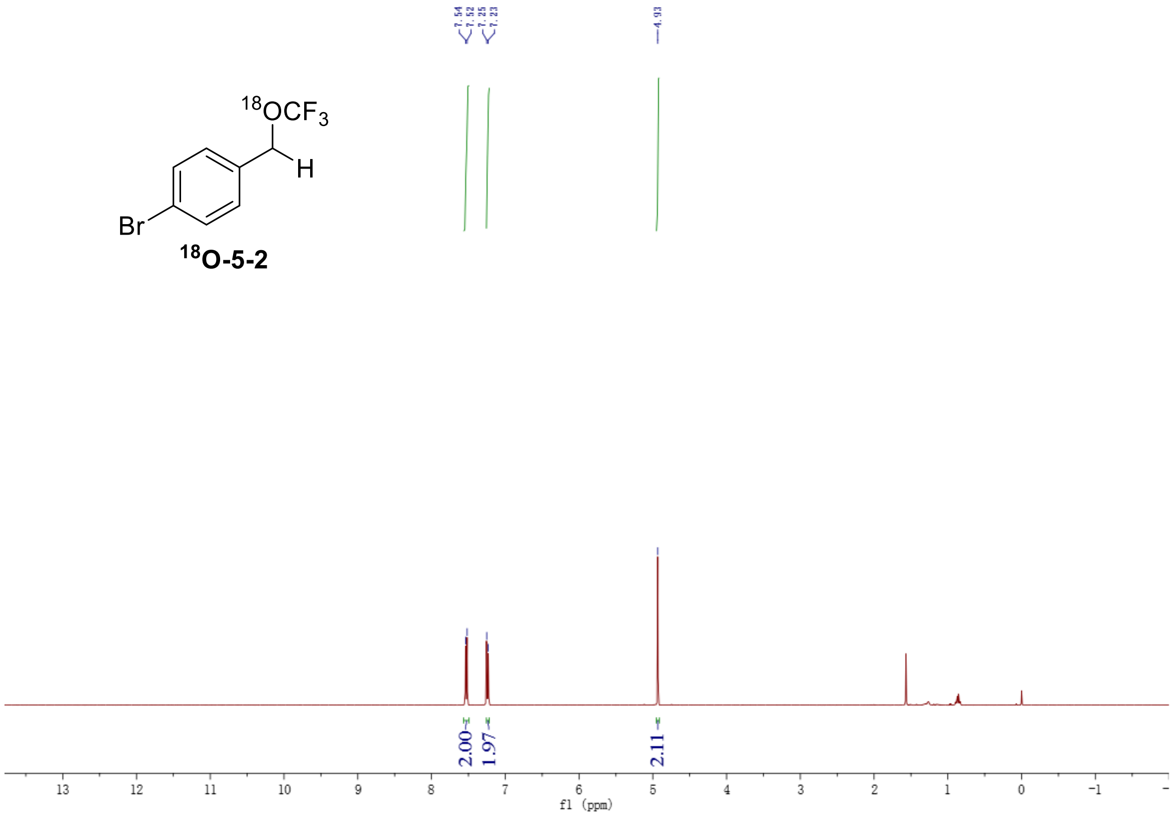

Supplementary Figure 129. ^{1}H NMR spectrum (400 MHz, $CDCl_3$) of **^{18}O-5-2**

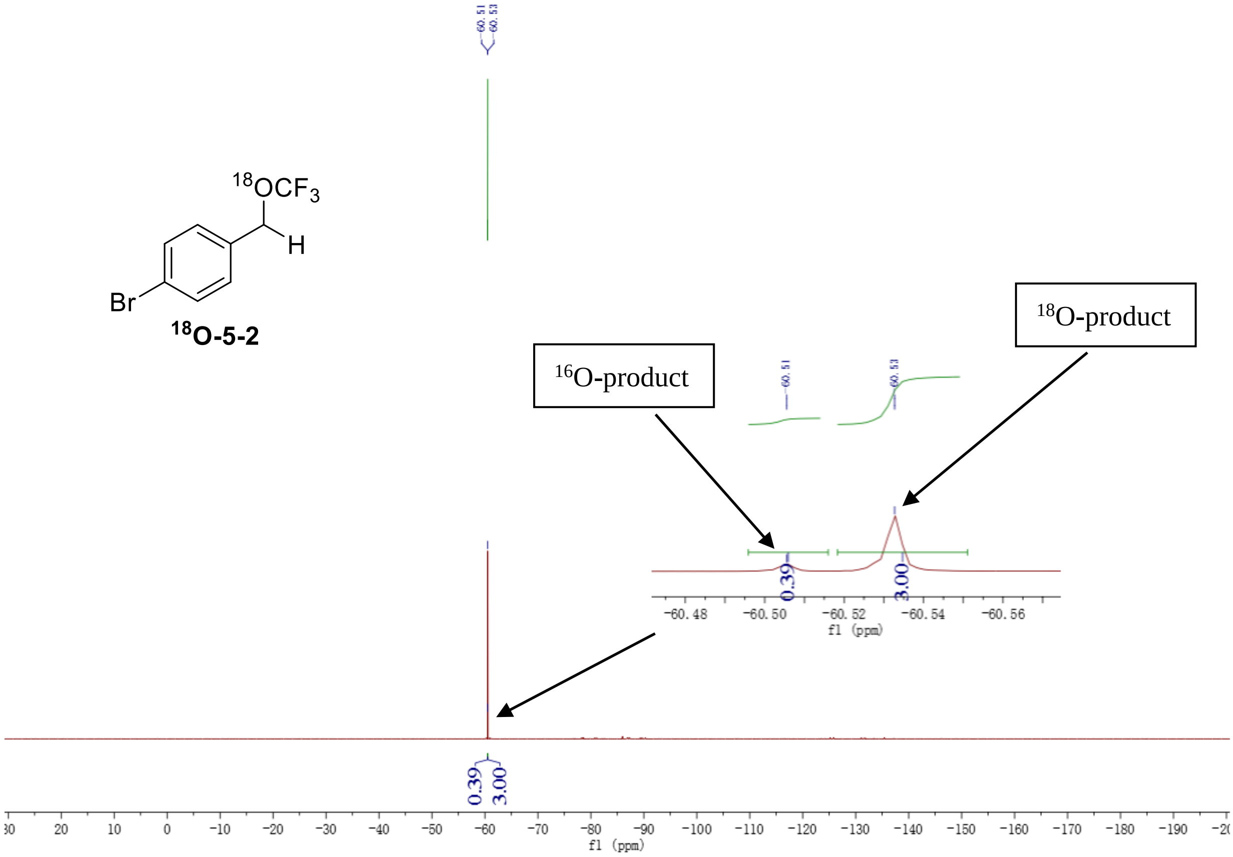

Supplementary Figure 130. ^{19}F NMR spectrum (376 MHz, $CDCl_3$) of **^{18}O-5-2**

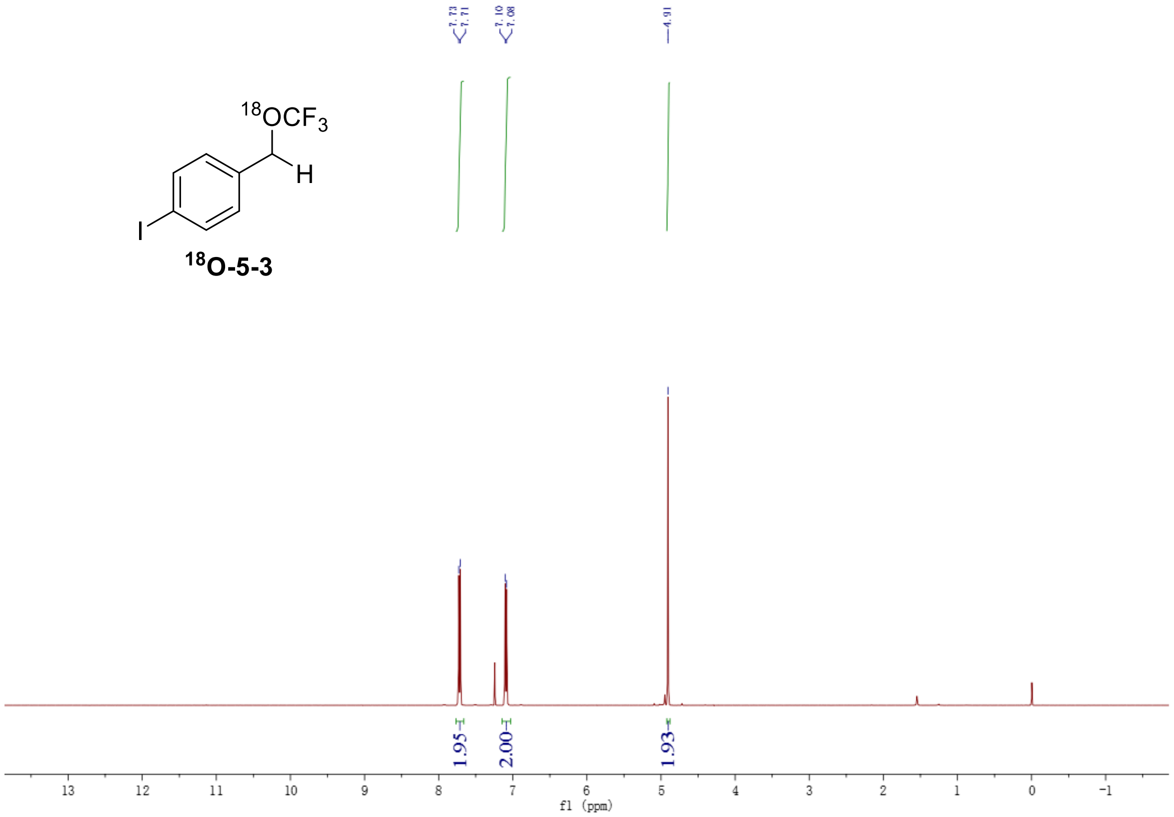

Supplementary Figure 131. ^{1}H NMR spectrum (400 MHz, $CDCl_3$) of **^{18}O-5-3**

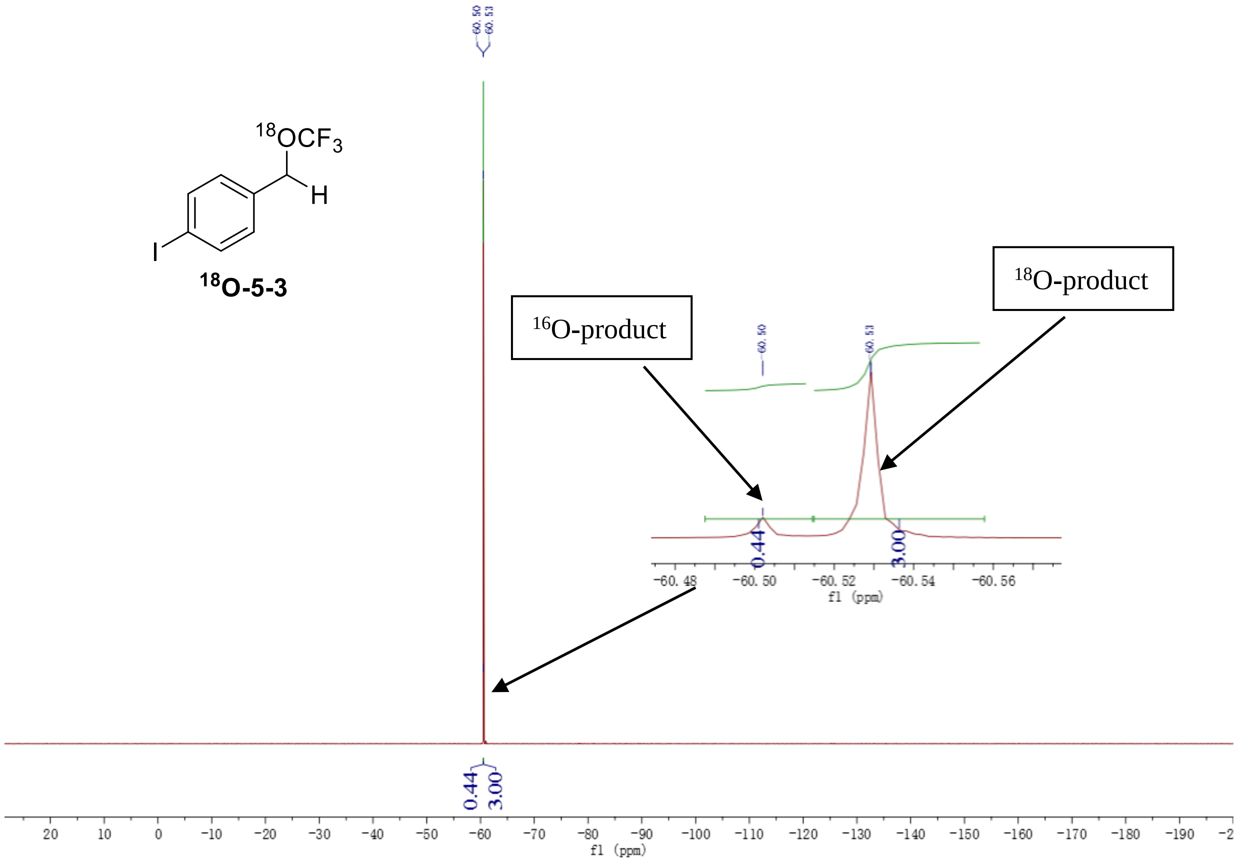

Supplementary Figure 132. ^{19}F NMR spectrum (376 MHz, $CDCl_3$) of **^{18}O-5-3**

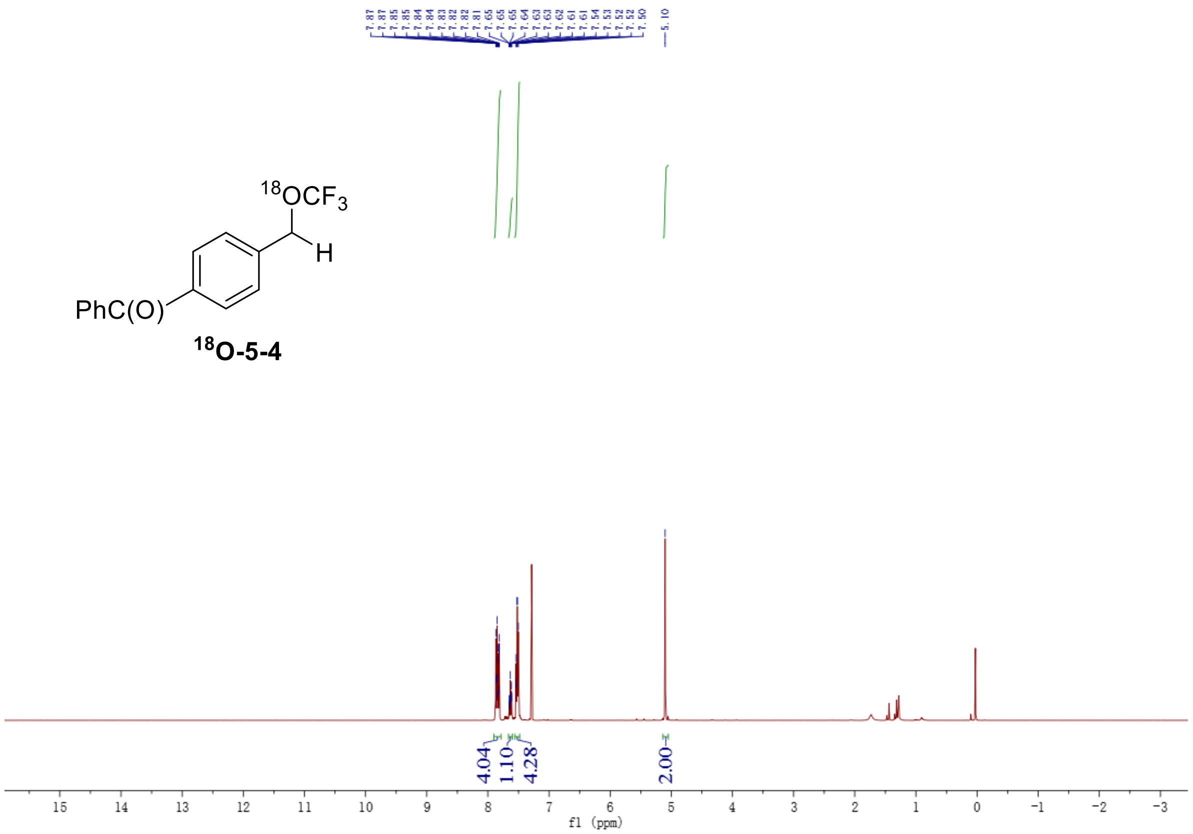

Supplementary Figure 133. ^{1}H NMR spectrum (400 MHz, $CDCl_3$) of **^{18}O-5-4**

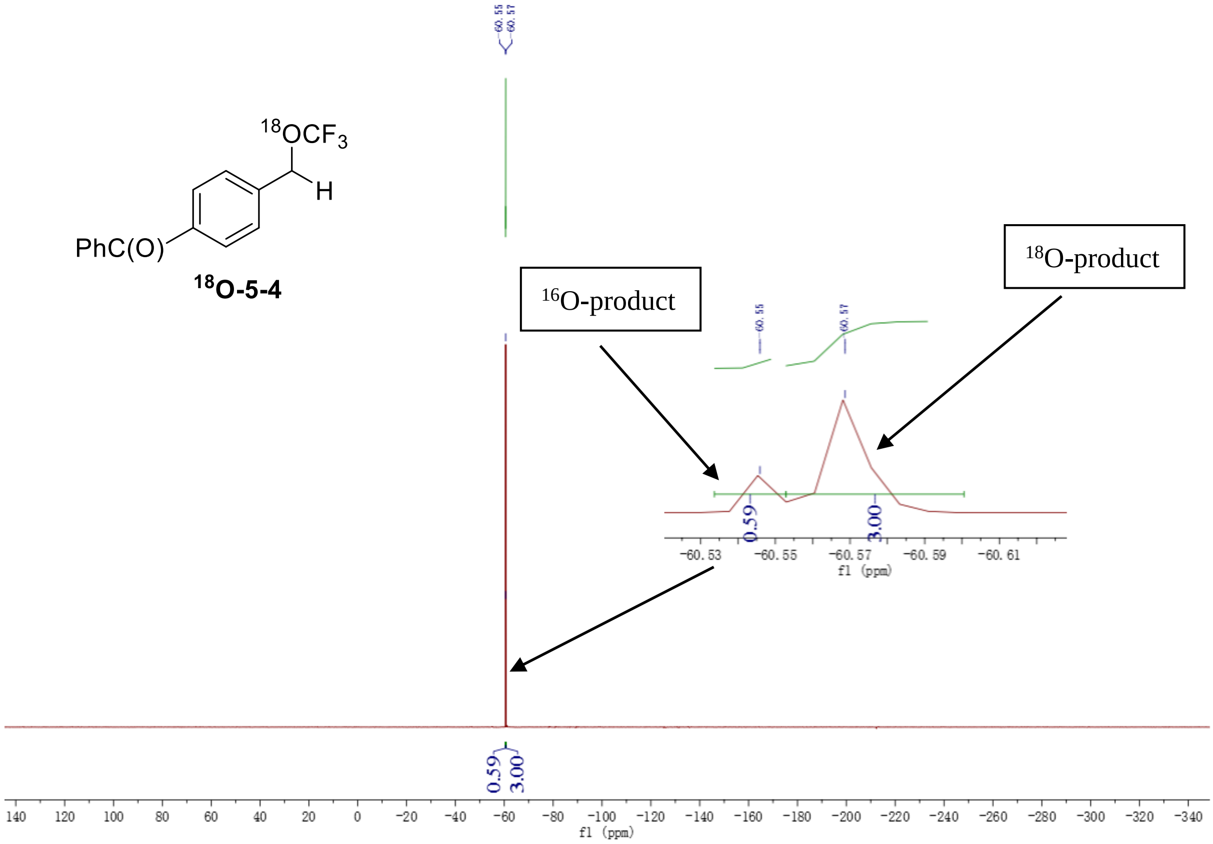

Supplementary Figure 134. ^{19}F NMR spectrum (376 MHz, $CDCl_3$) of **^{18}O-5-4**

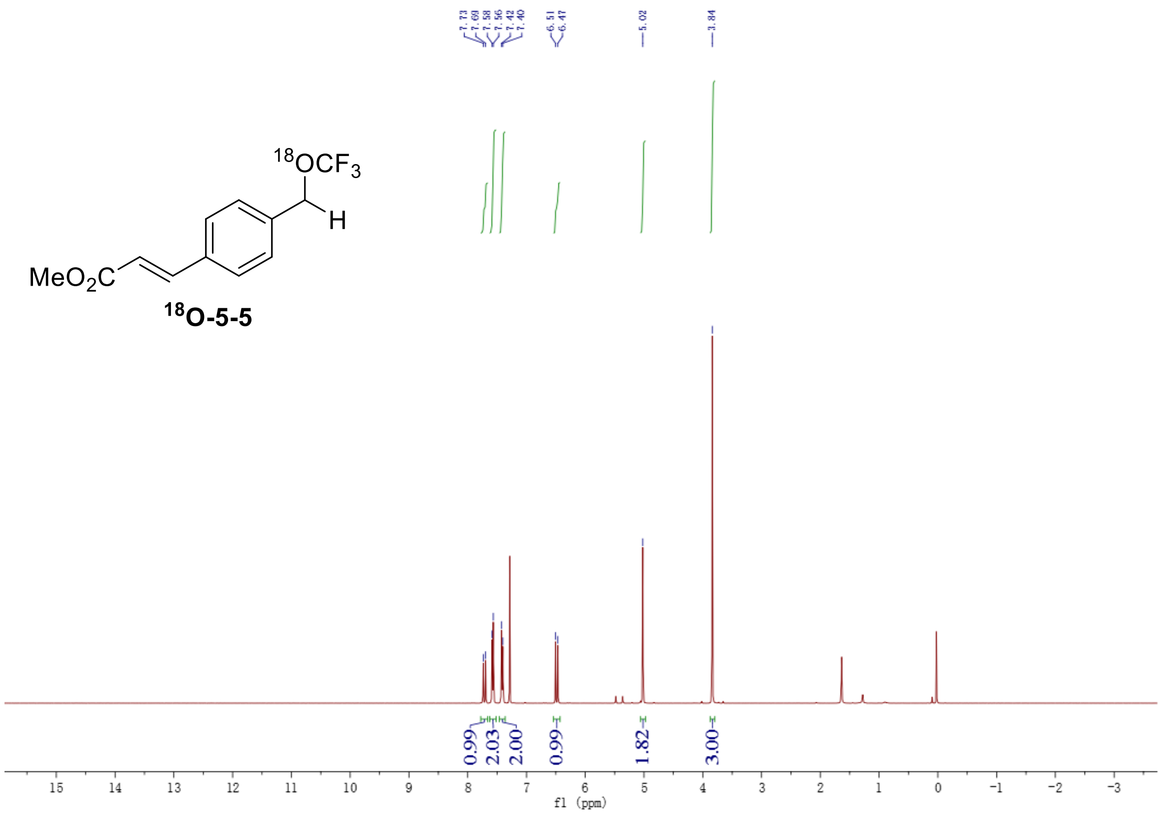

Supplementary Figure 135. ^{1}H NMR spectrum (400 MHz, $CDCl_3$) of **^{18}O-5-5**

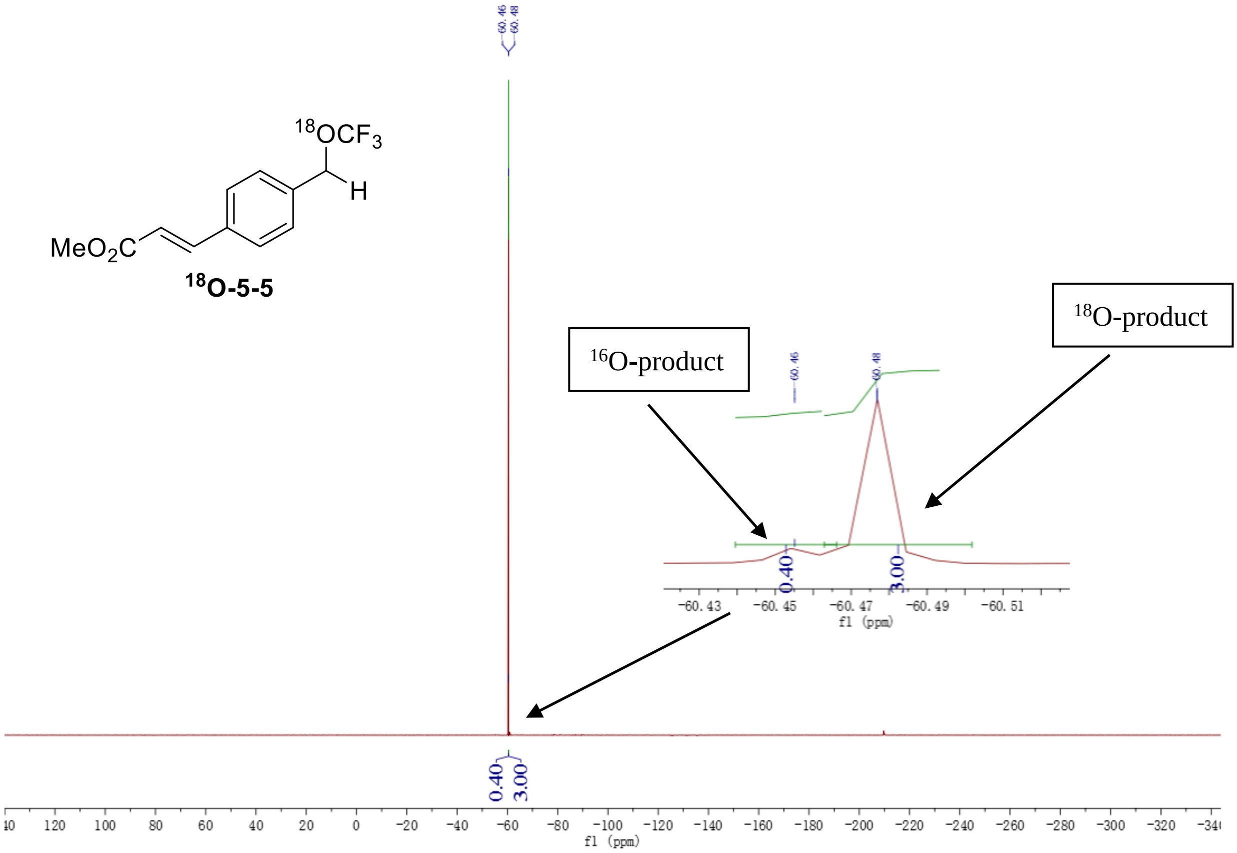

Supplementary Figure 136. ^{19}F NMR spectrum (376 MHz, $CDCl_3$) of **^{18}O-5-5**

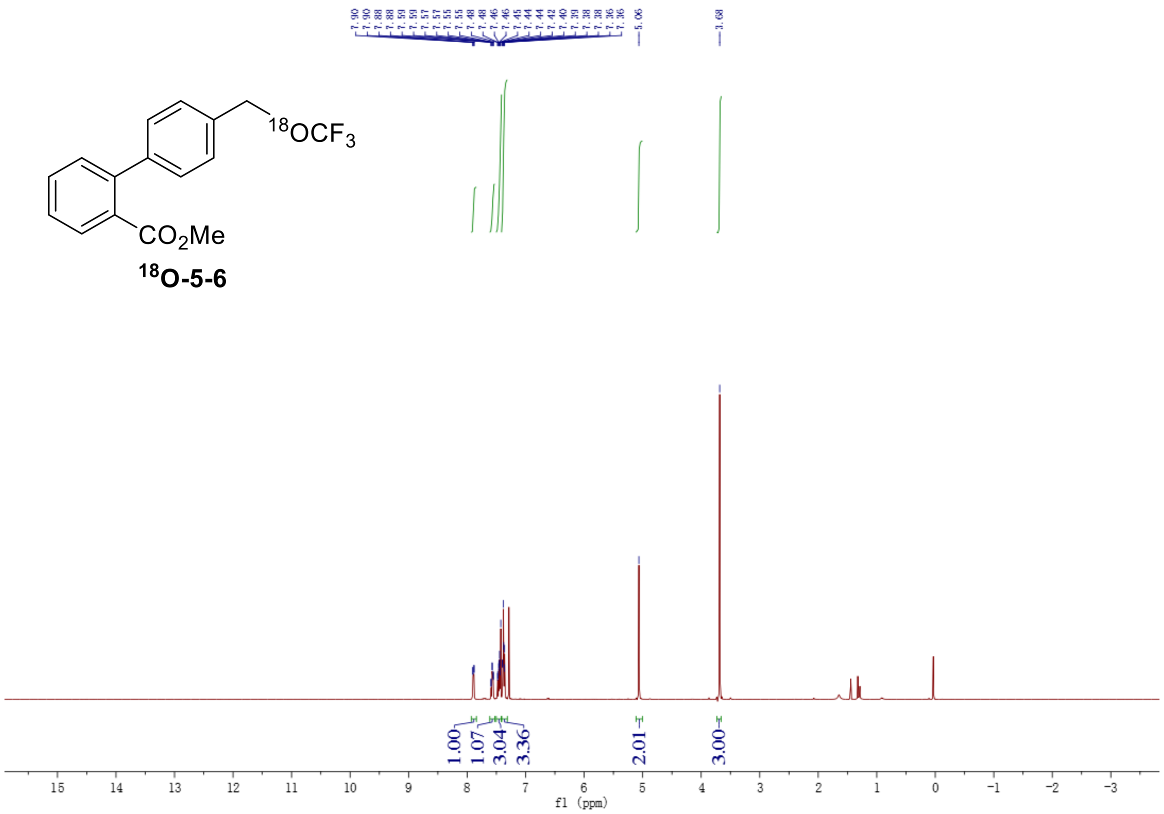

Supplementary Figure 137. ^{1}H NMR spectrum (400 MHz, $CDCl_3$) of **^{18}O-5-6**

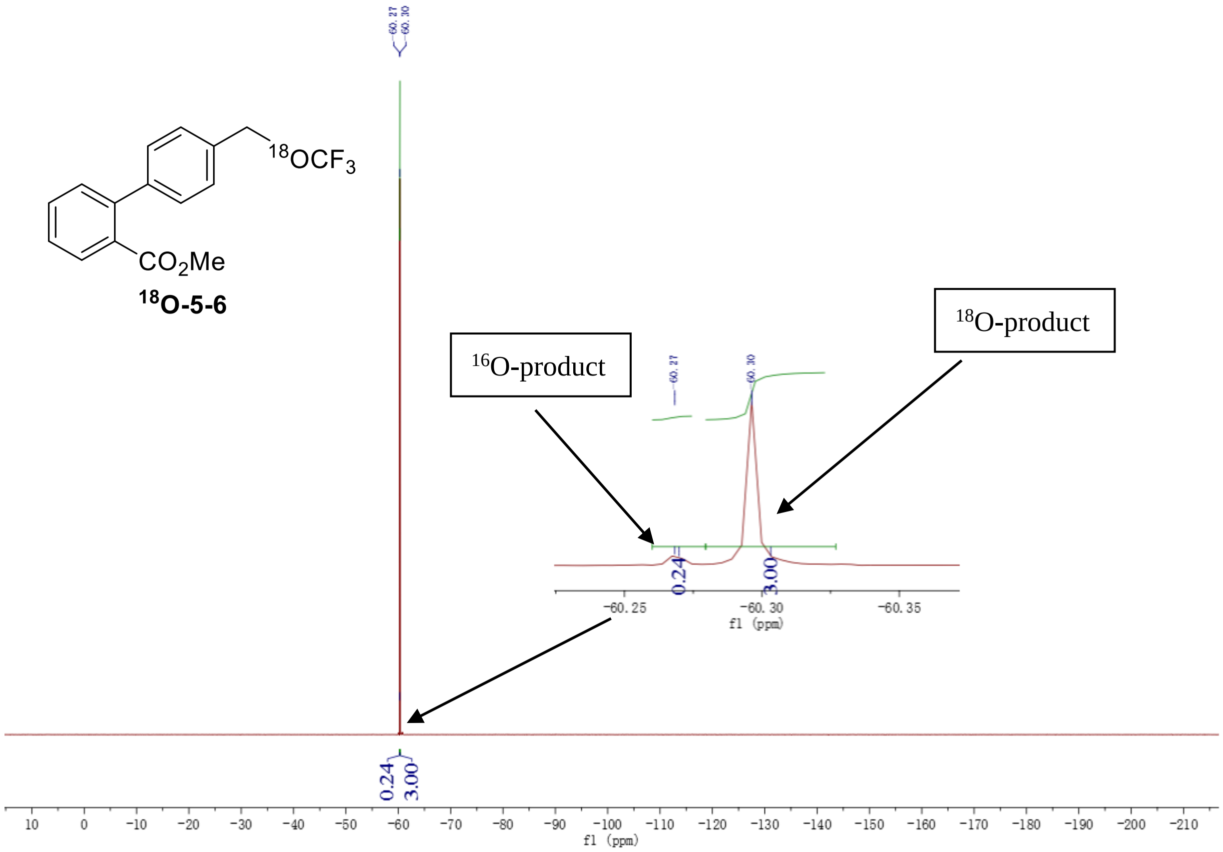

Supplementary Figure 138. ^{19}F NMR spectrum (376 MHz, $CDCl_3$) of **^{18}O-5-6**

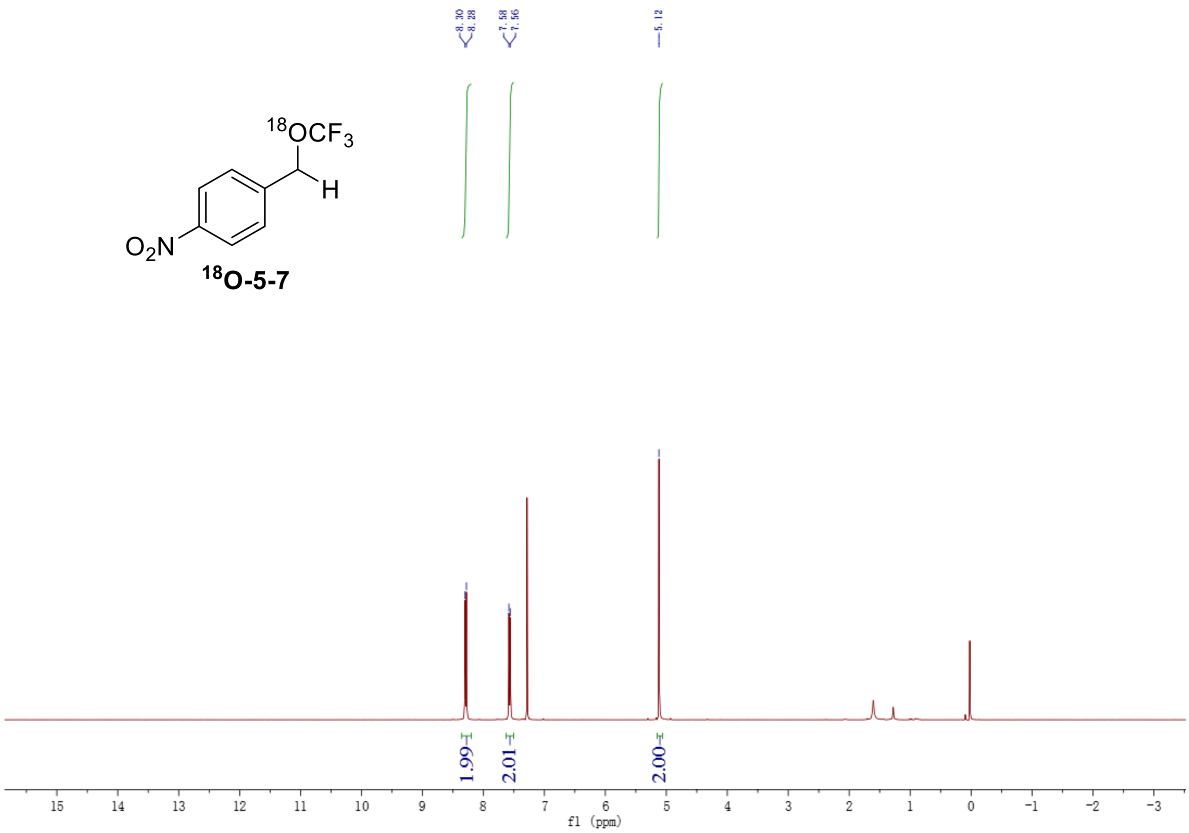

Supplementary Figure 139. ^{1}H NMR spectrum (400 MHz, $CDCl_3$) of **^{18}O-5-7**

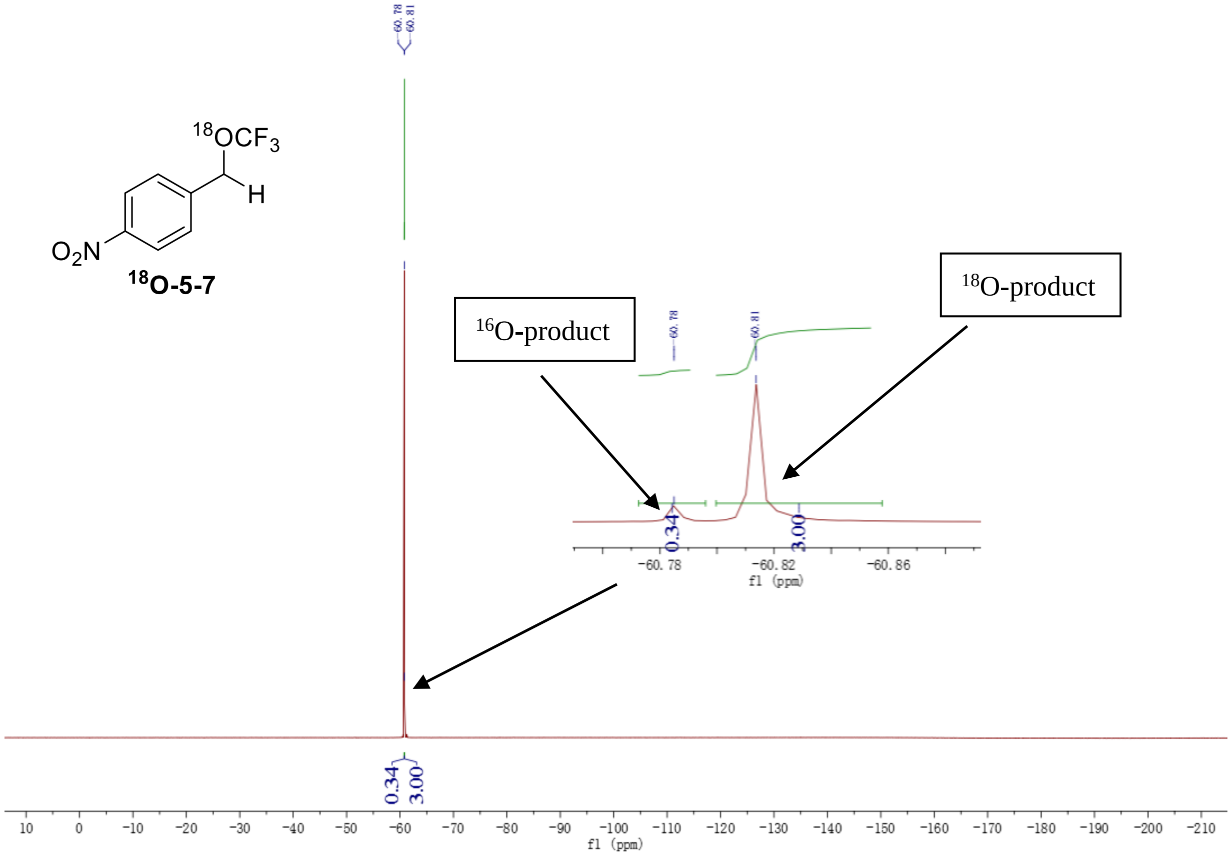

Supplementary Figure 140. ^{19}F NMR spectrum (376 MHz, $CDCl_3$) of **^{18}O-5-7**

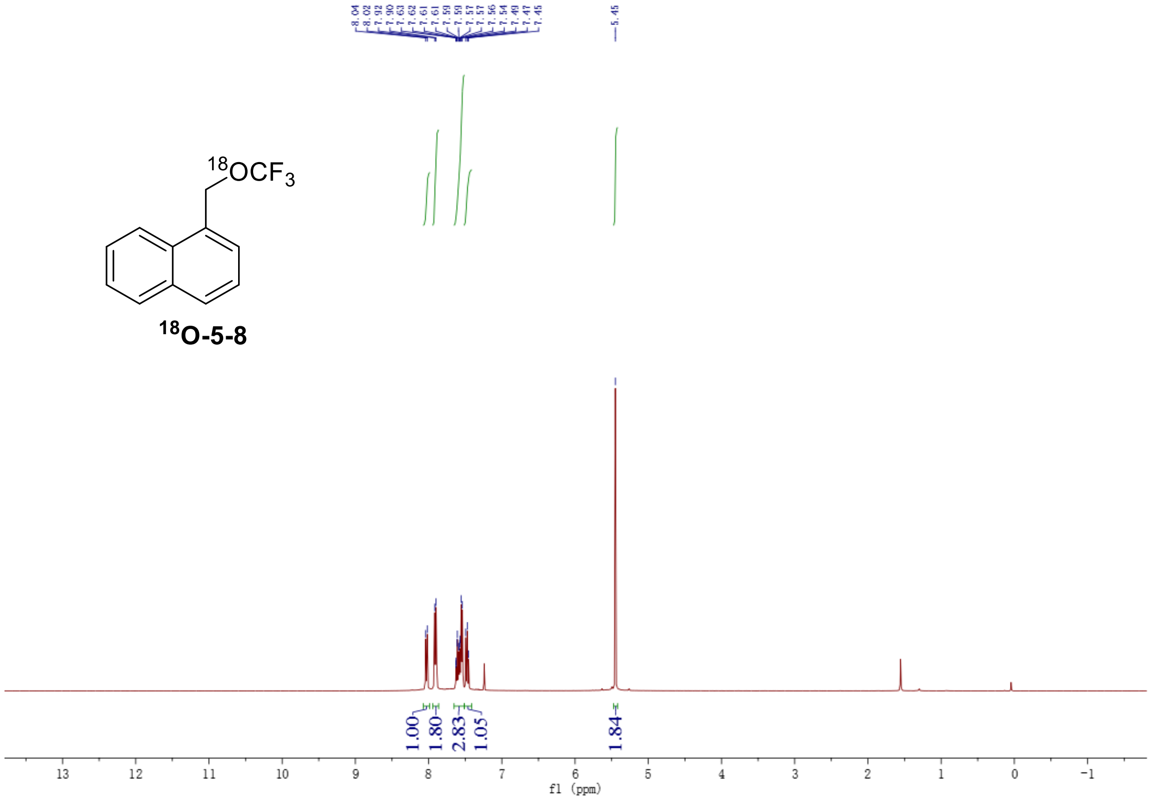

Supplementary Figure 141. ^{1}H NMR spectrum (400 MHz, $CDCl_3$) of **^{18}O-5-8**

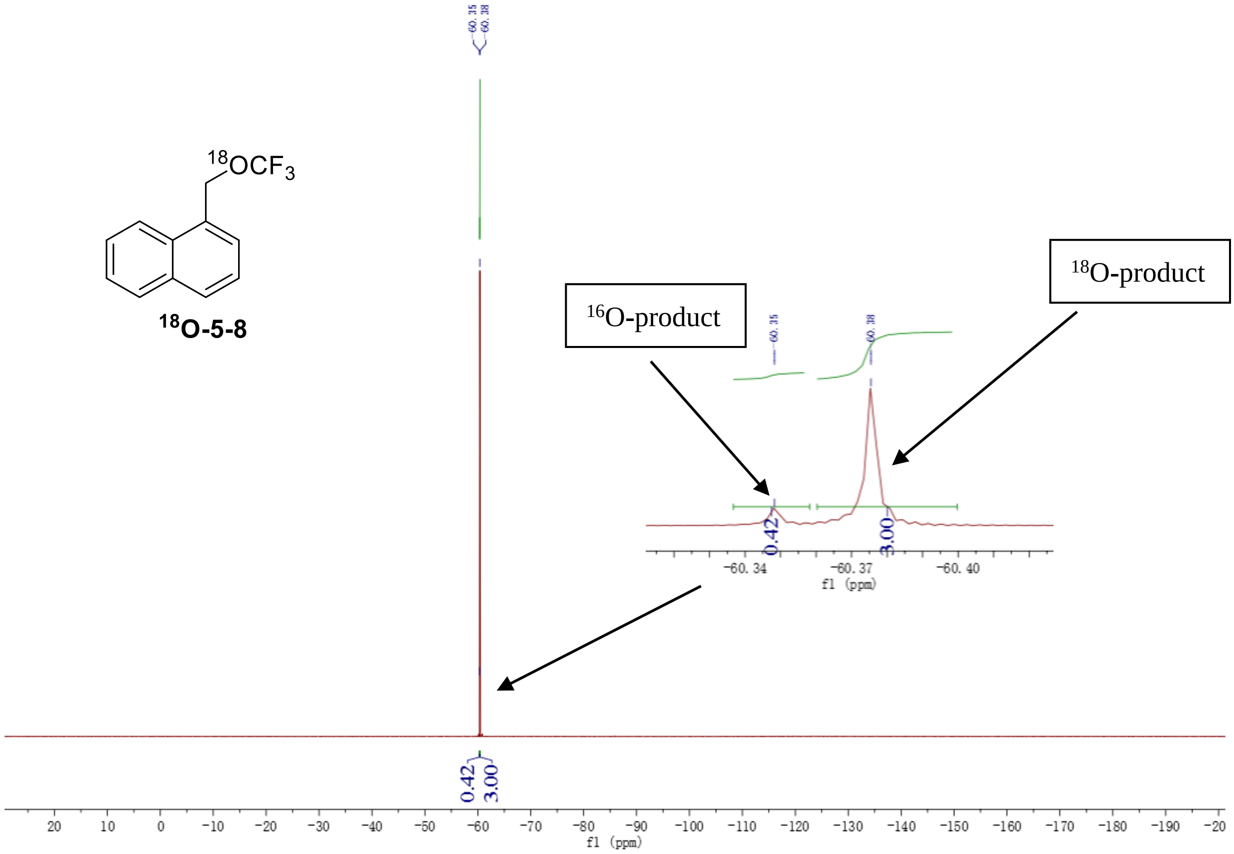

Supplementary Figure 142. ^{19}F NMR spectrum (376 MHz, $CDCl_3$) of **^{18}O-5-8**

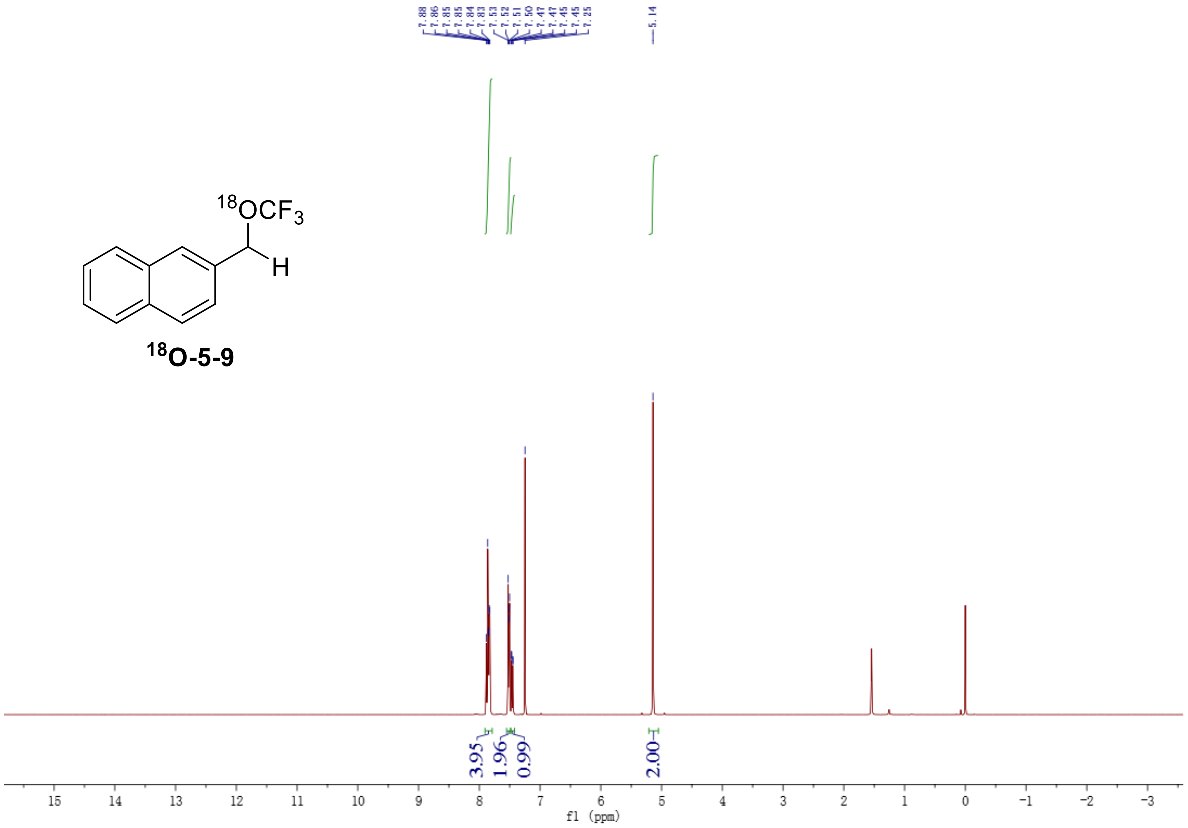

Supplementary Figure 143. ^{1}H NMR spectrum (400 MHz, $CDCl_3$) of **^{18}O-5-9**

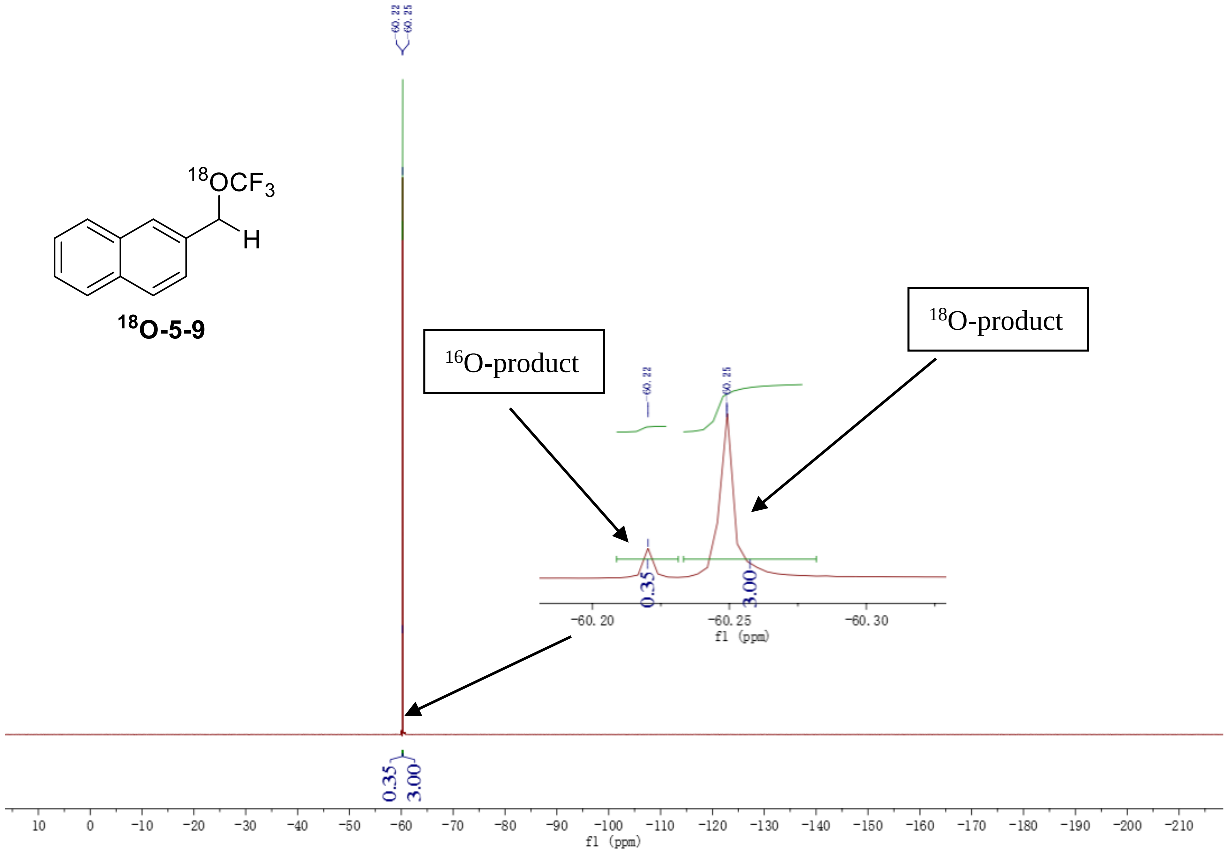

Supplementary Figure 144. ^{19}F NMR spectrum (376 MHz, $CDCl_3$) of **^{18}O-5-9**

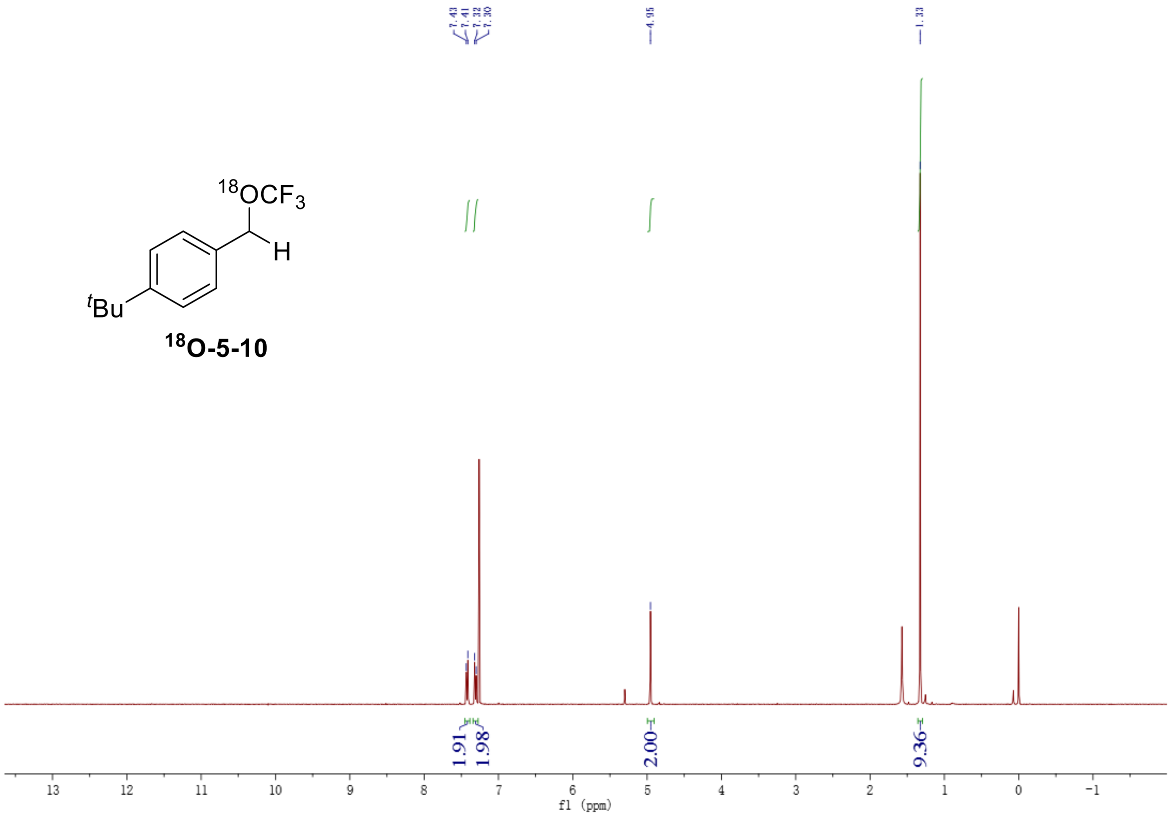

Supplementary Figure 145. ^{1}H NMR spectrum (400 MHz, $CDCl_3$) of **^{18}O-5-10**

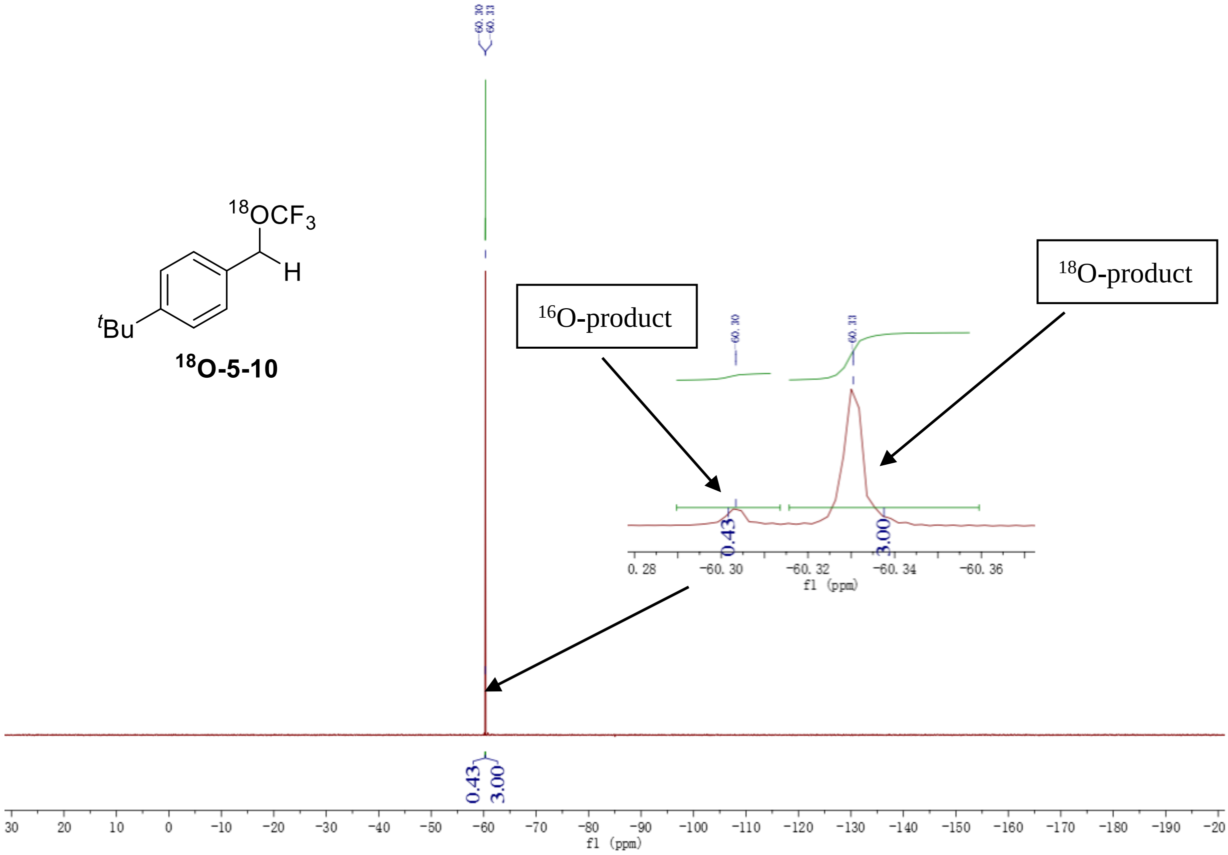

Supplementary Figure 146. ^{19}F NMR spectrum (376 MHz, $CDCl_3$) of **^{18}O-5-10**

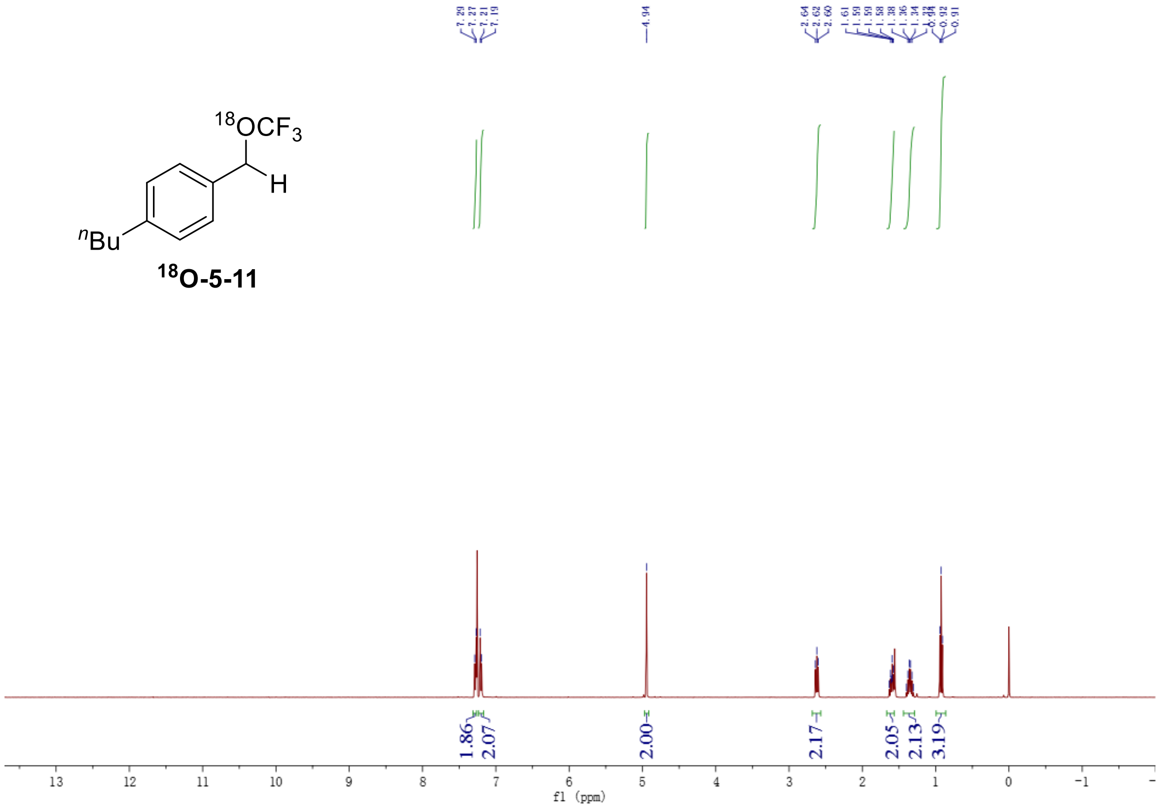

Supplementary Figure 147. ^{1}H NMR spectrum (400 MHz, $CDCl_3$) of **^{18}O-5-11**

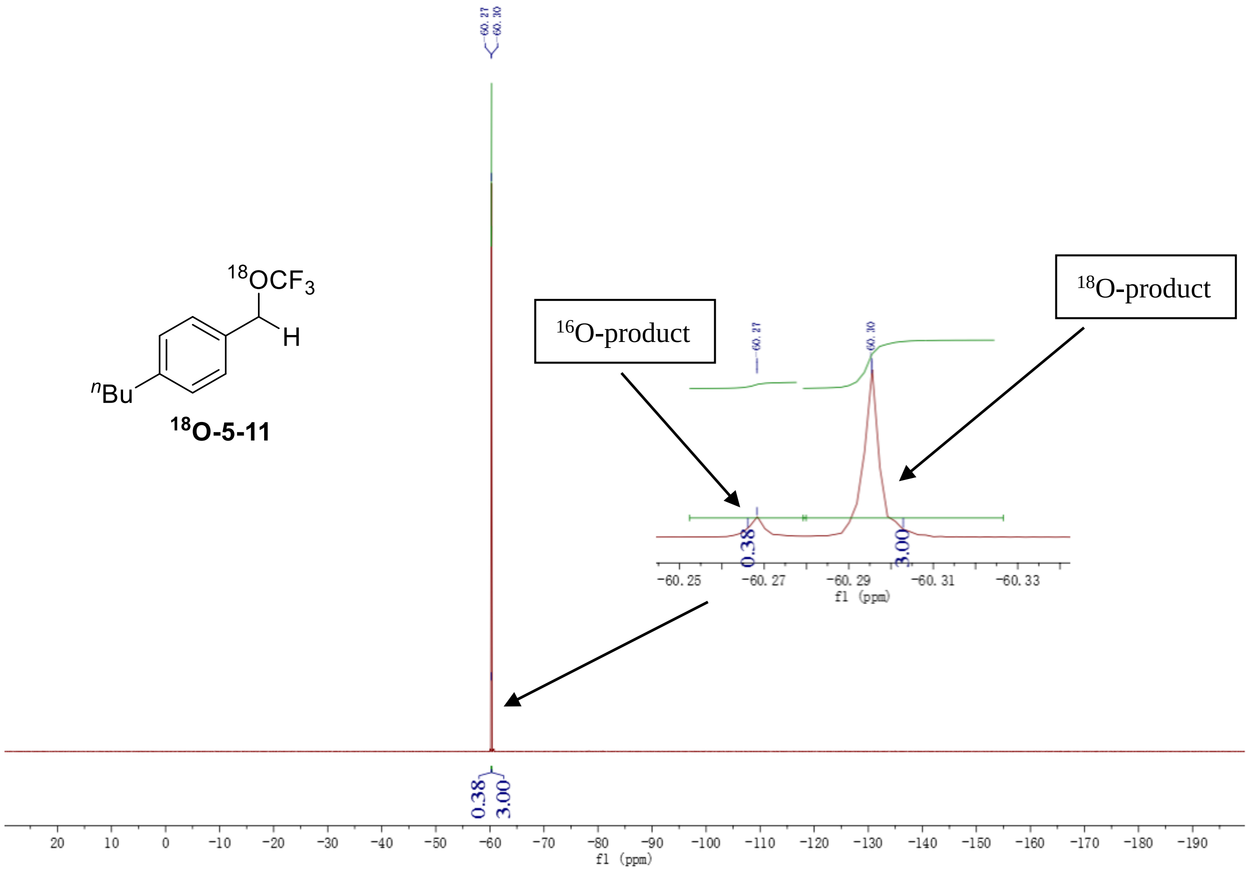

Supplementary Figure 148. ^{19}F NMR spectrum (376 MHz, $CDCl_3$) of **^{18}O-5-11**

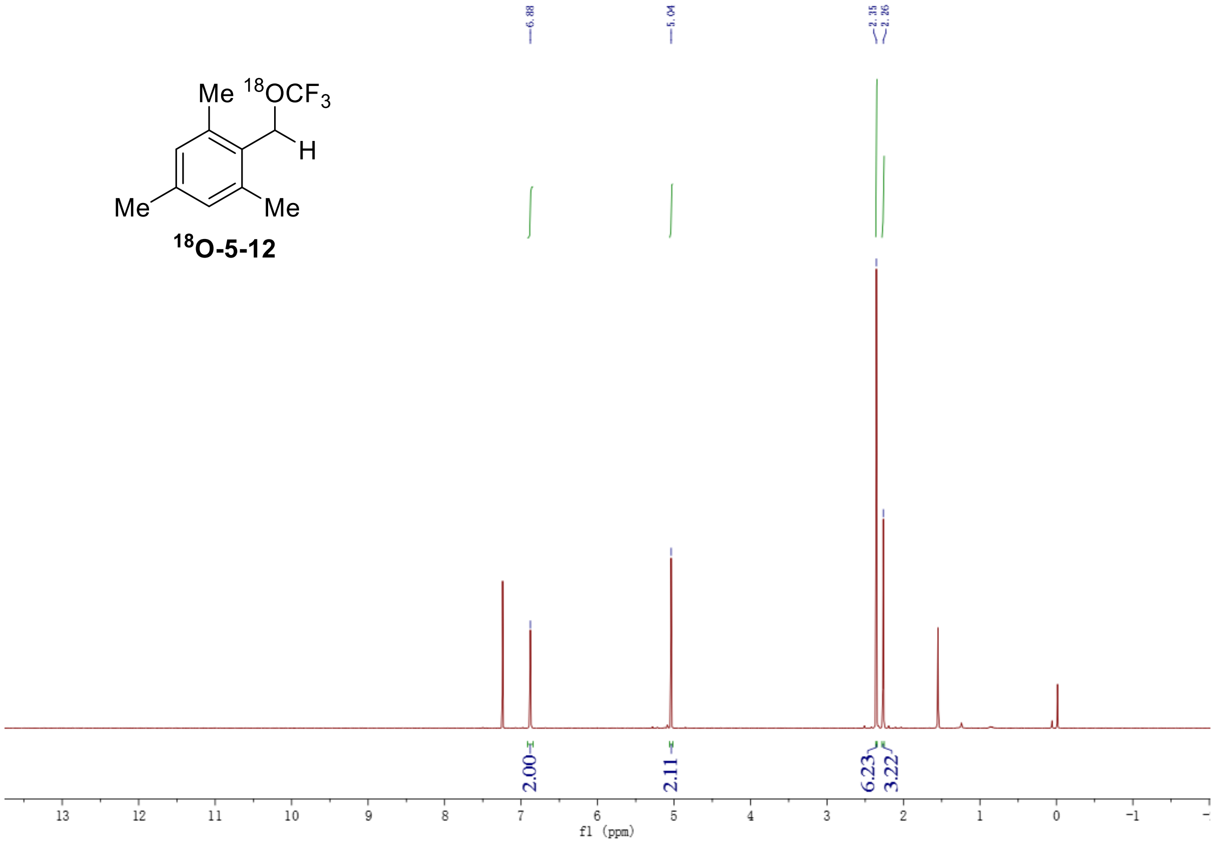

Supplementary Figure 149. ^{1}H NMR spectrum (400 MHz, $CDCl_3$) of **^{18}O-5-12**

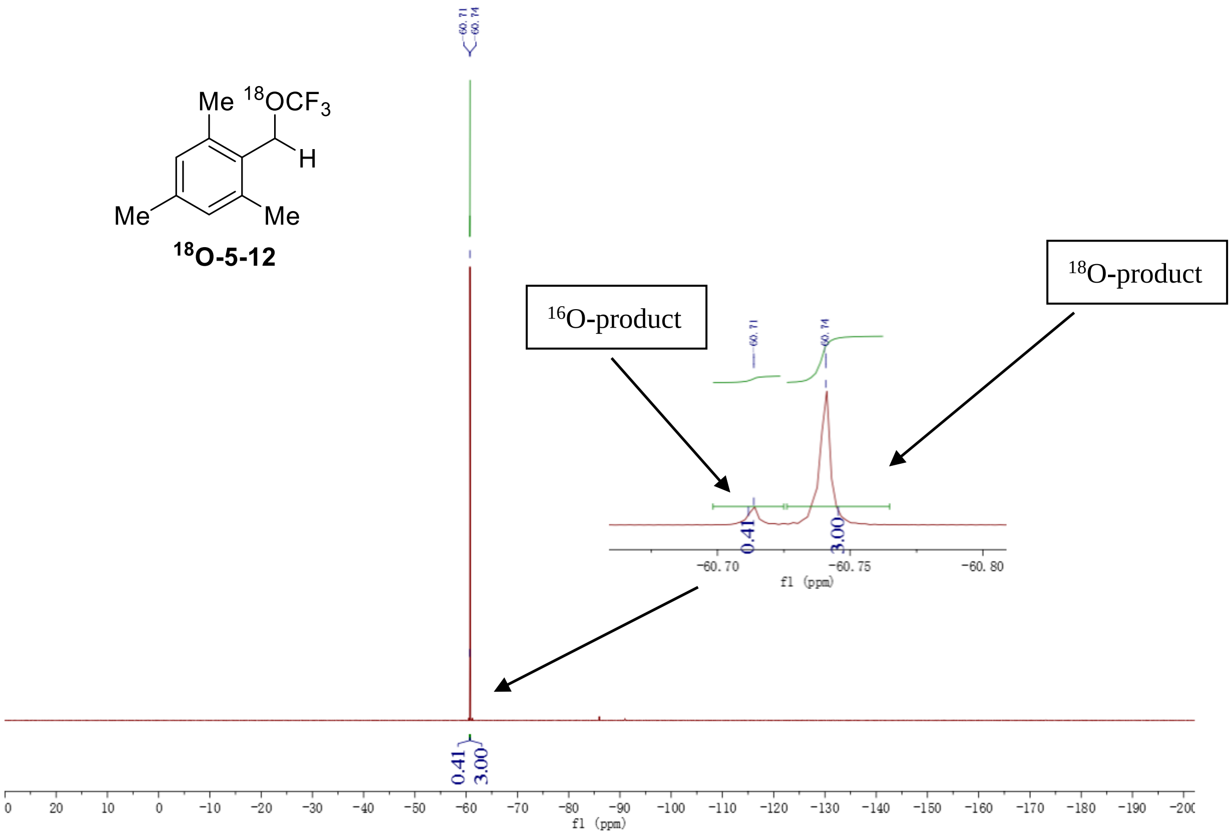

Supplementary Figure 150. ^{19}F NMR spectrum (376 MHz, $CDCl_3$) of **^{18}O-5-12**

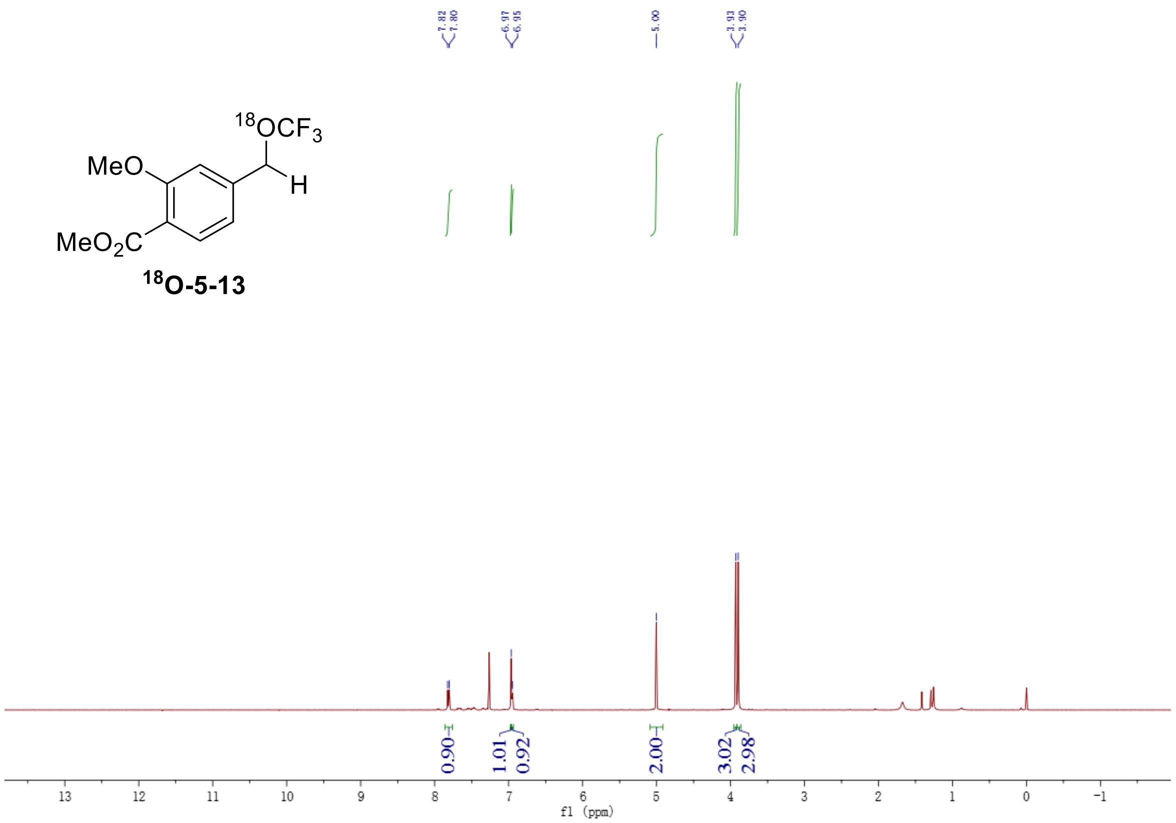

Supplementary Figure 151. ^{1}H NMR spectrum (400 MHz, CDCl$_3$) of **^{18}O-5-13**

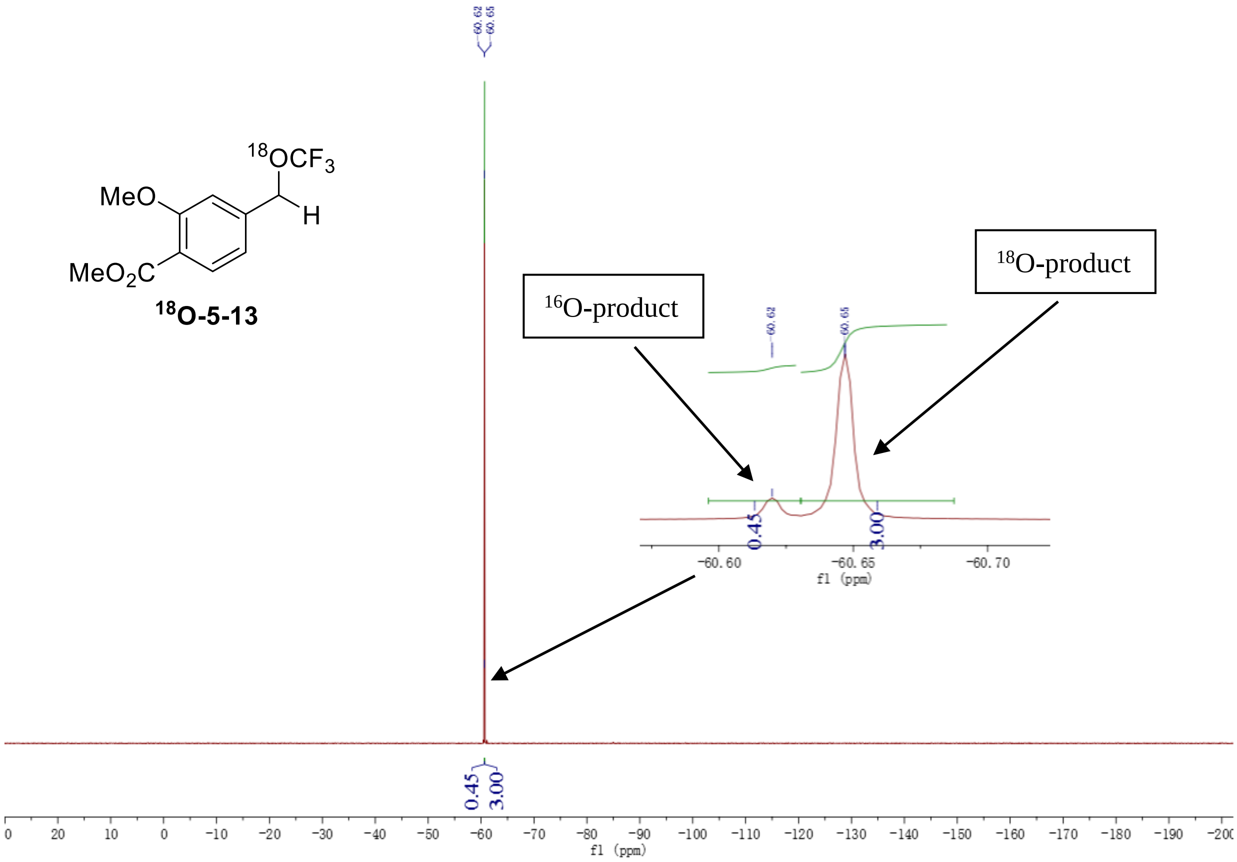

Supplementary Figure 152. ^{19}F NMR spectrum (376 MHz, CDCl$_3$) of **^{18}O-5-13**

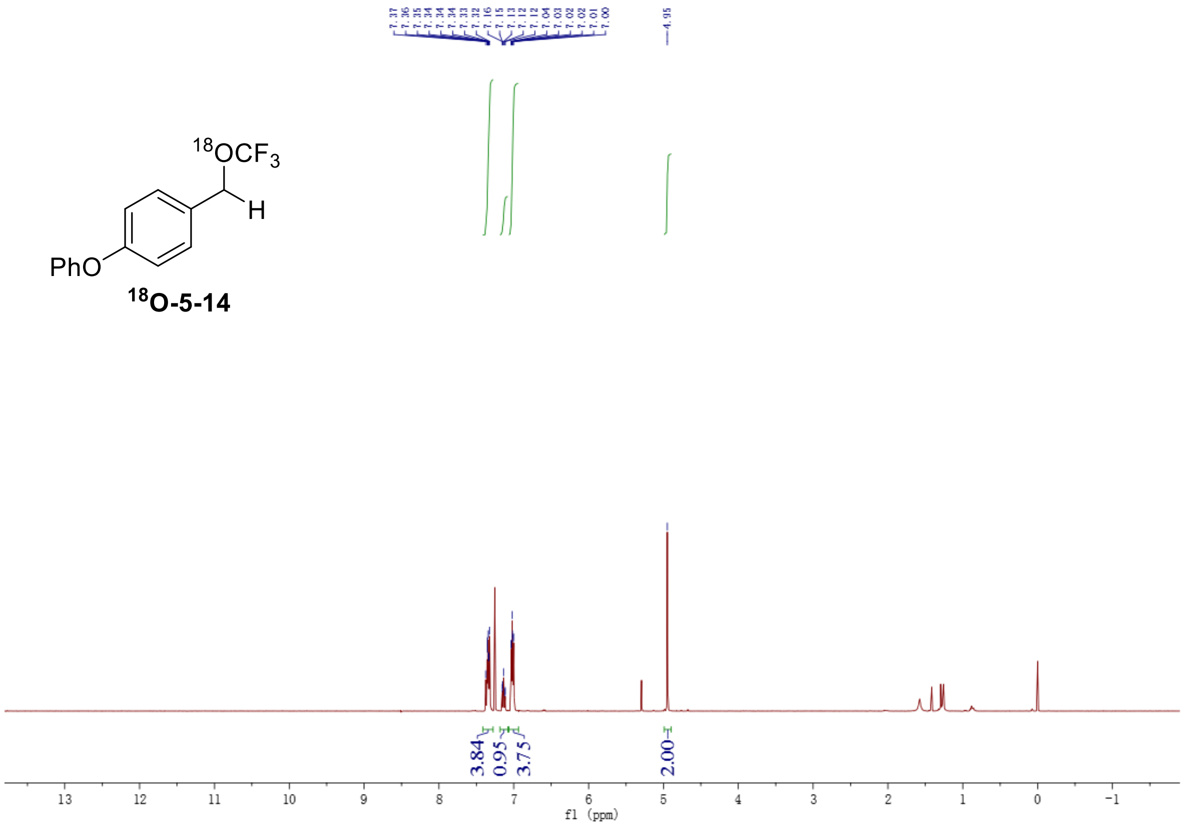

Supplementary Figure 153. ^{1}H NMR spectrum (400 MHz, $CDCl_3$) of **^{18}O-5-14**

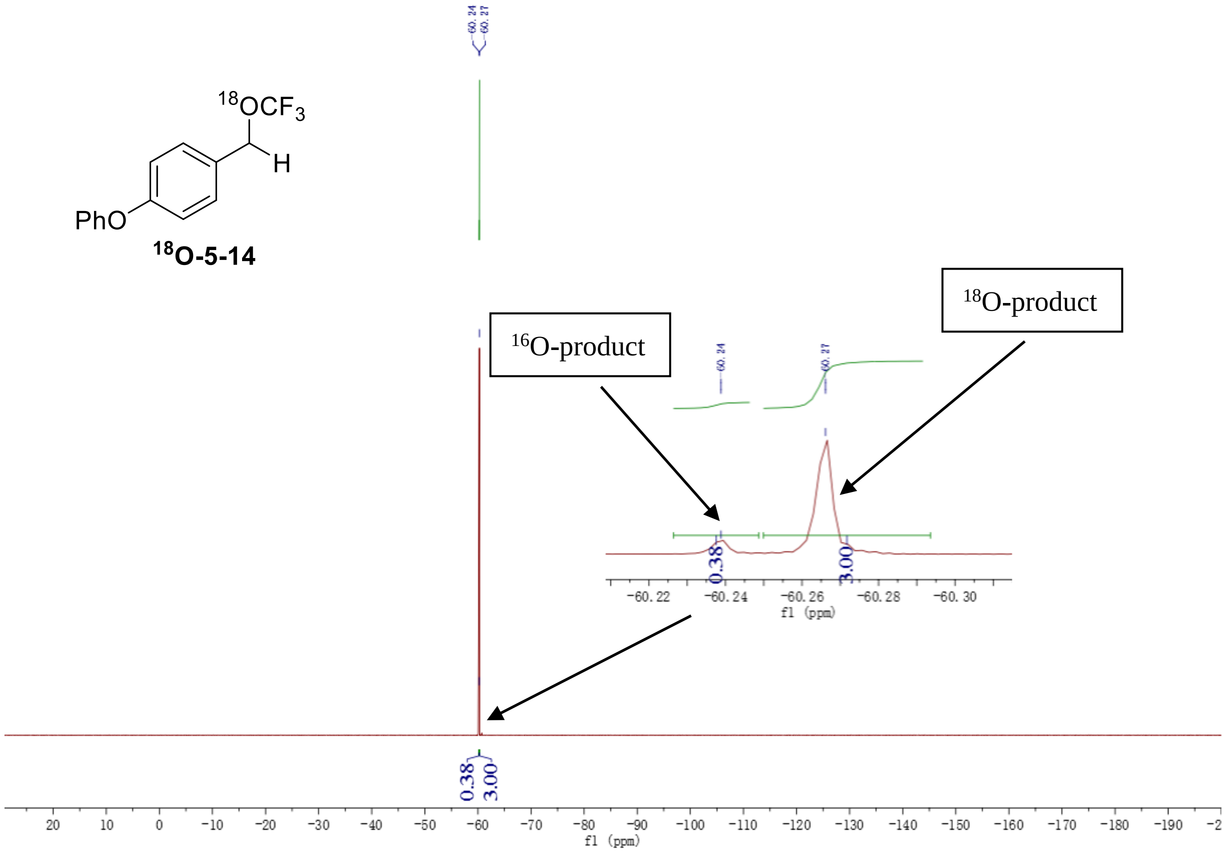

Supplementary Figure 154. ^{19}F NMR spectrum (376 MHz, $CDCl_3$) of **^{18}O-5-14**

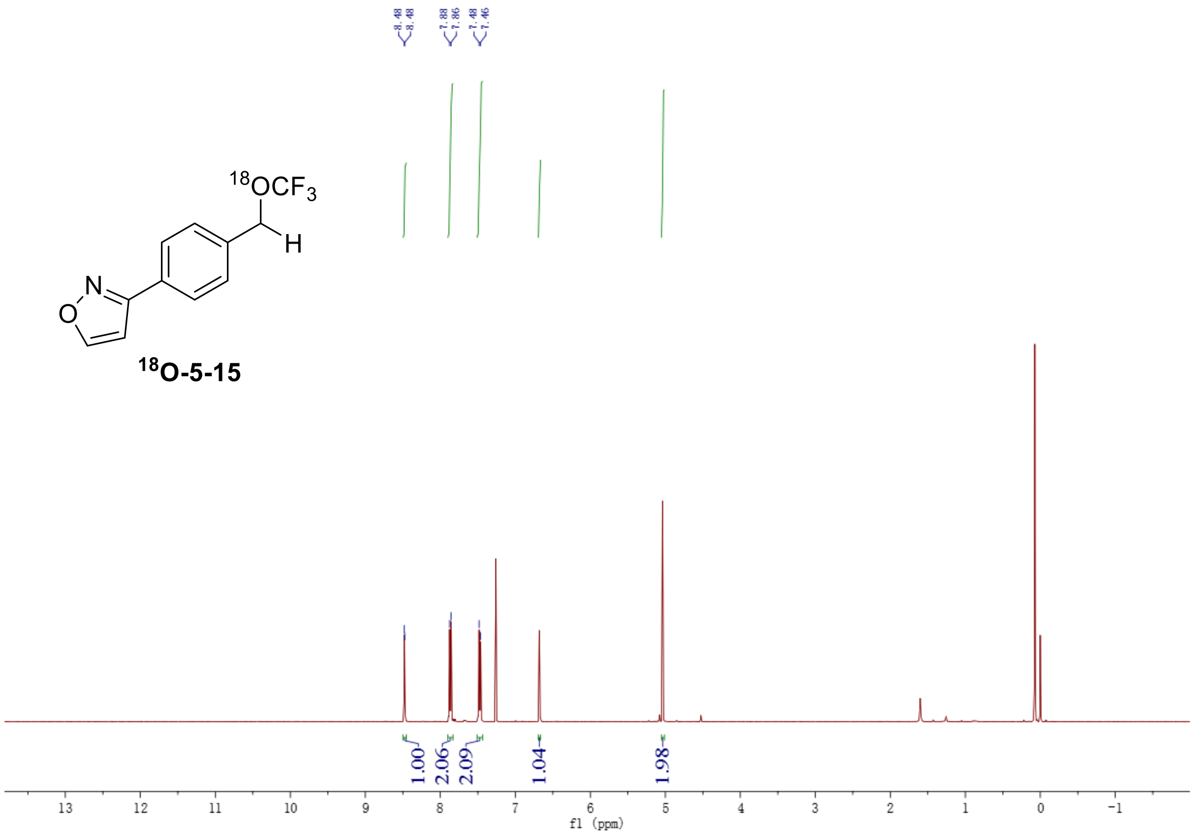

Supplementary Figure 155. ^{1}H NMR spectrum (400 MHz, $CDCl_3$) of **^{18}O-5-15**

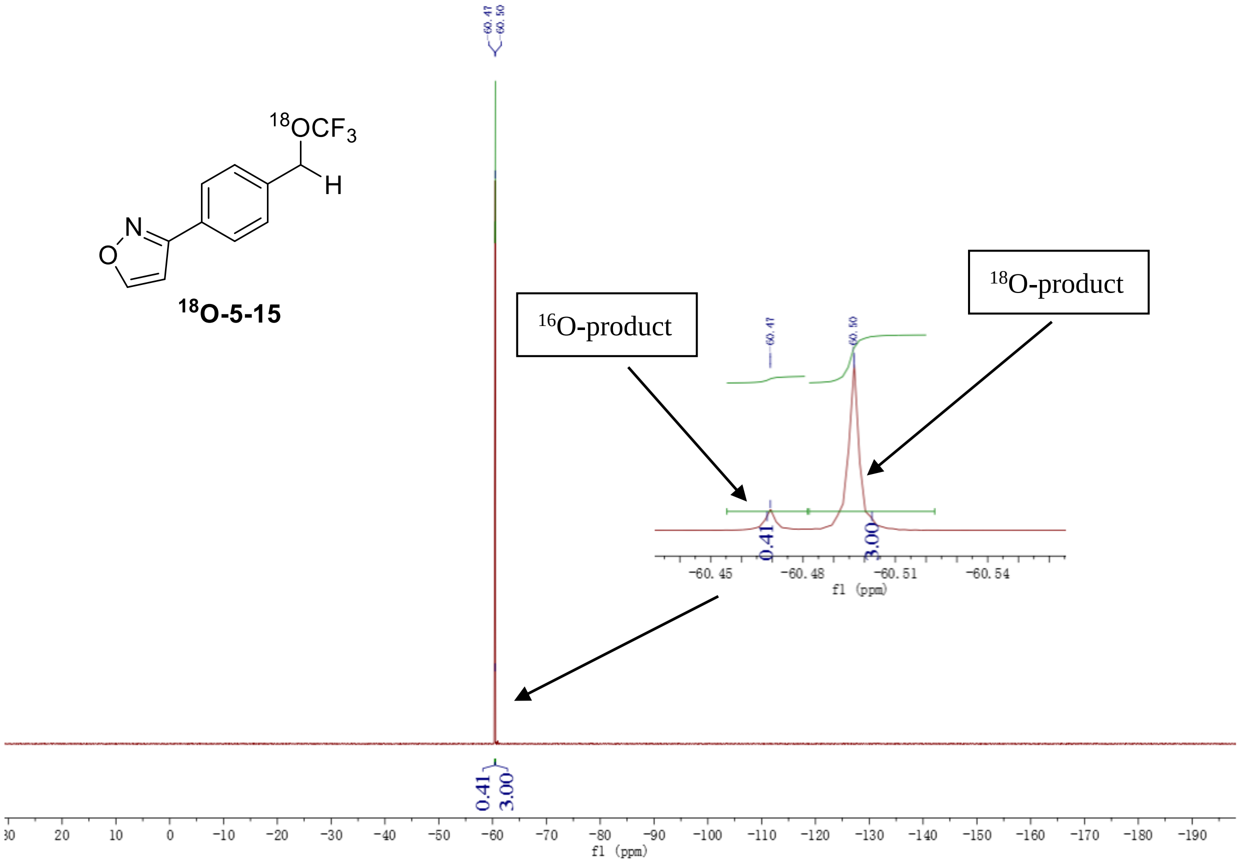

Supplementary Figure 156. ^{19}F NMR spectrum (376 MHz, $CDCl_3$) of **^{18}O-5-15**

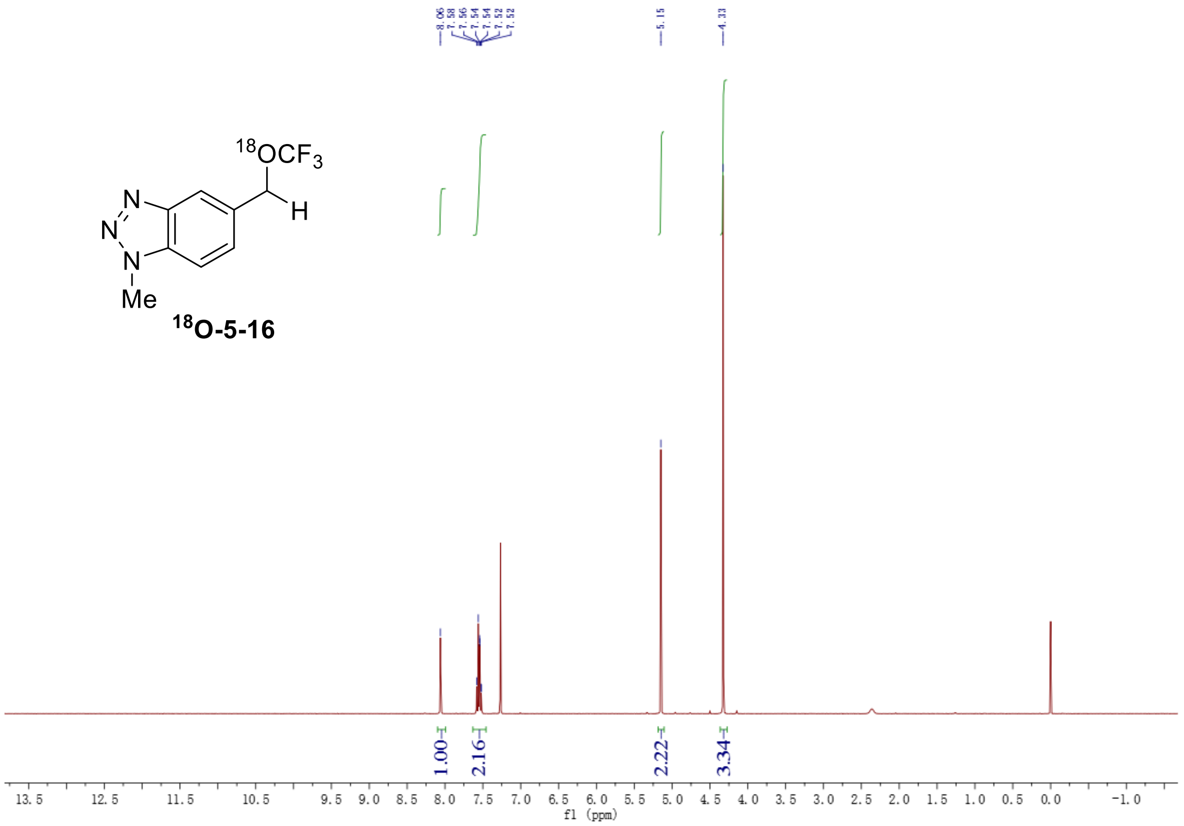

Supplementary Figure 157. ^{1}H NMR spectrum (400 MHz, $CDCl_3$) of **^{18}O-5-16**

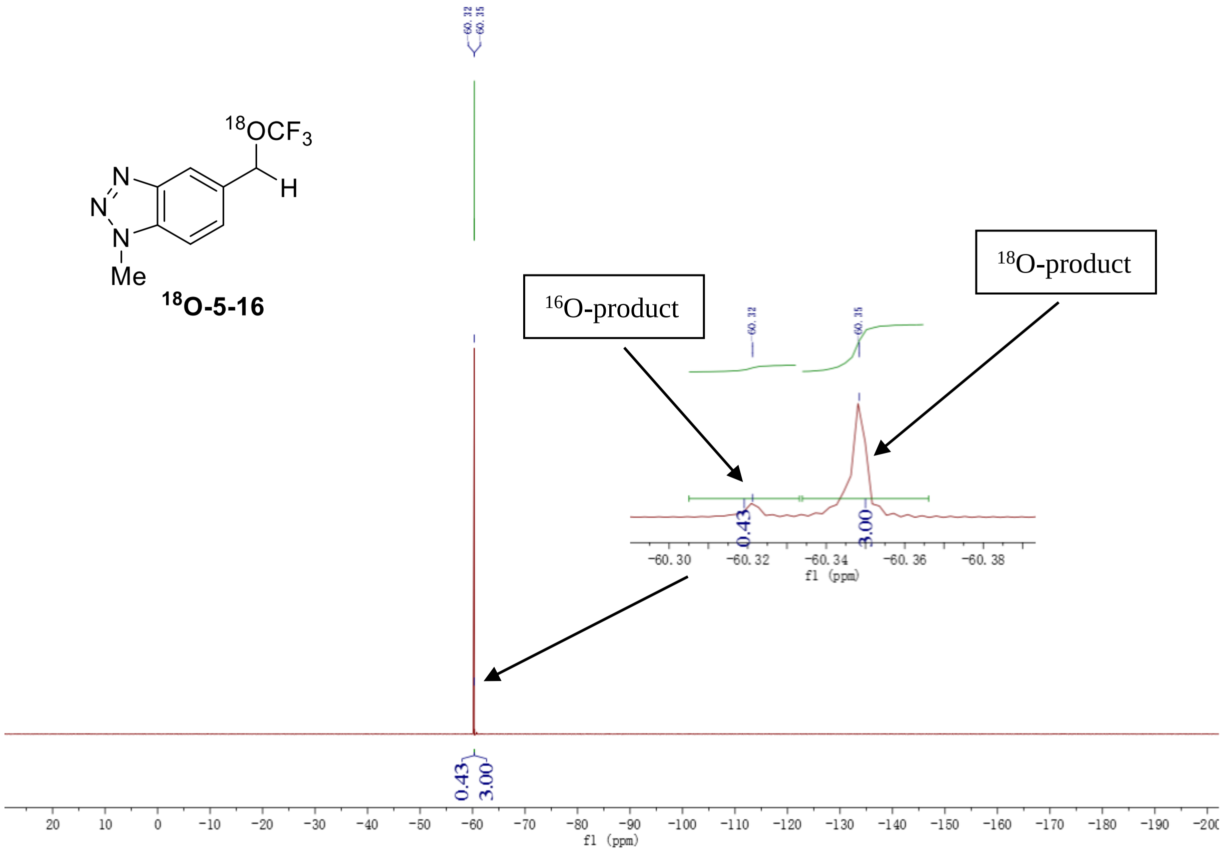

Supplementary Figure 158. ^{19}F NMR spectrum (376 MHz, $CDCl_3$) of **^{18}O-5-16**

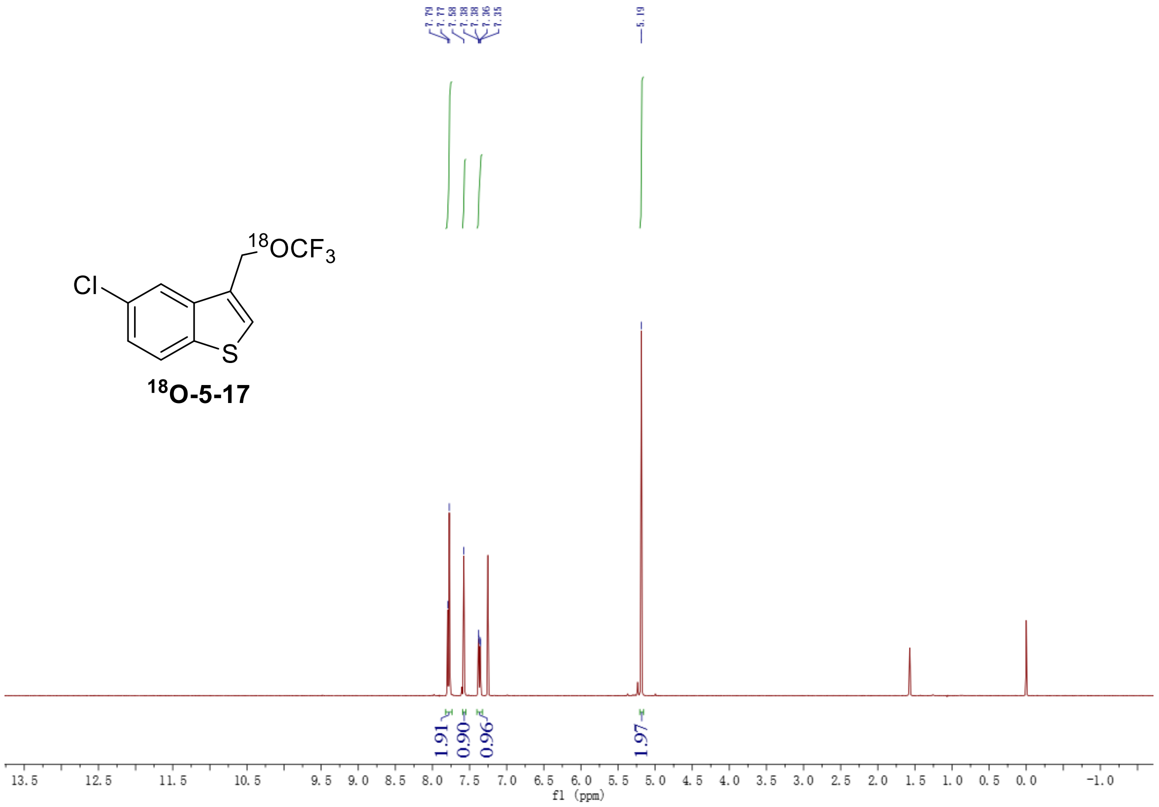

Supplementary Figure 159. ^{1}H NMR spectrum (400 MHz, $CDCl_3$) of **^{18}O-5-17**

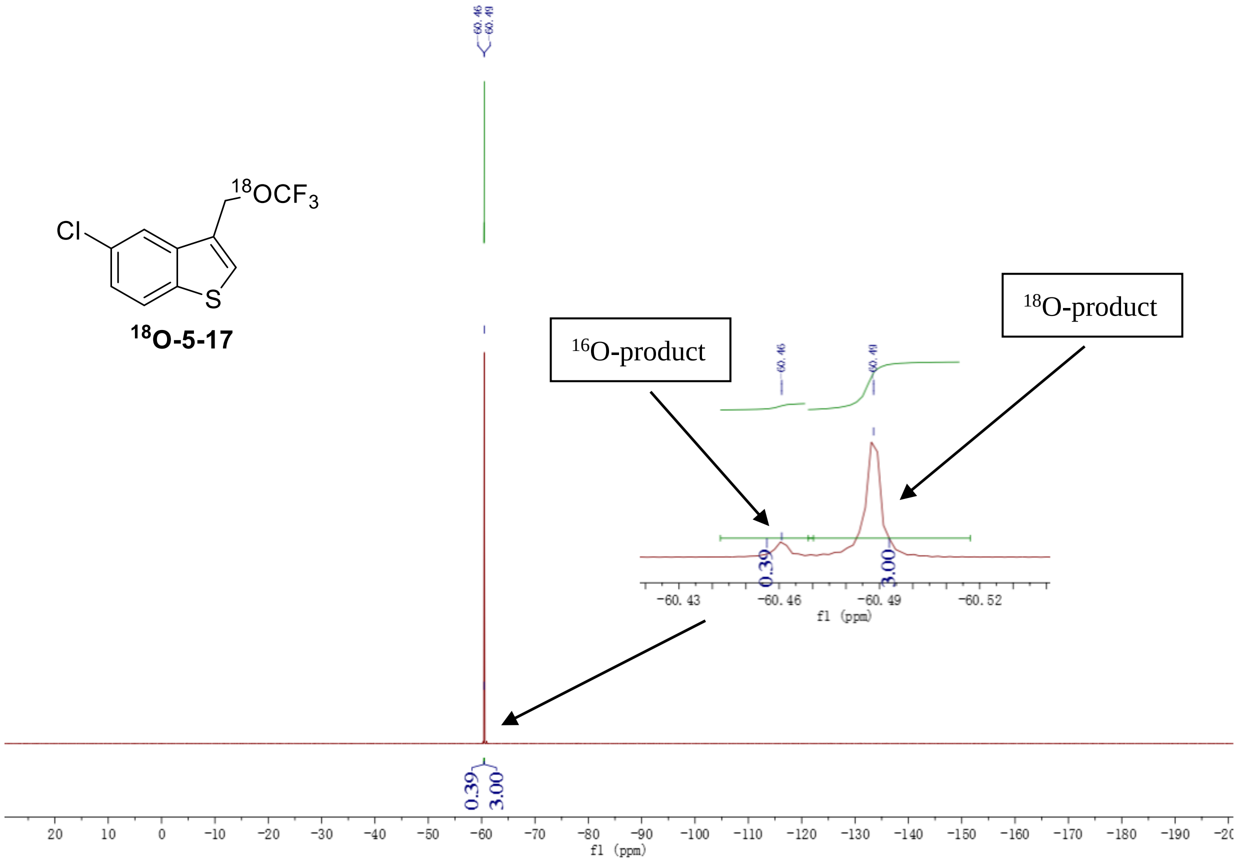

Supplementary Figure 160. ^{19}F NMR spectrum (376 MHz, $CDCl_3$) of **^{18}O-5-17**

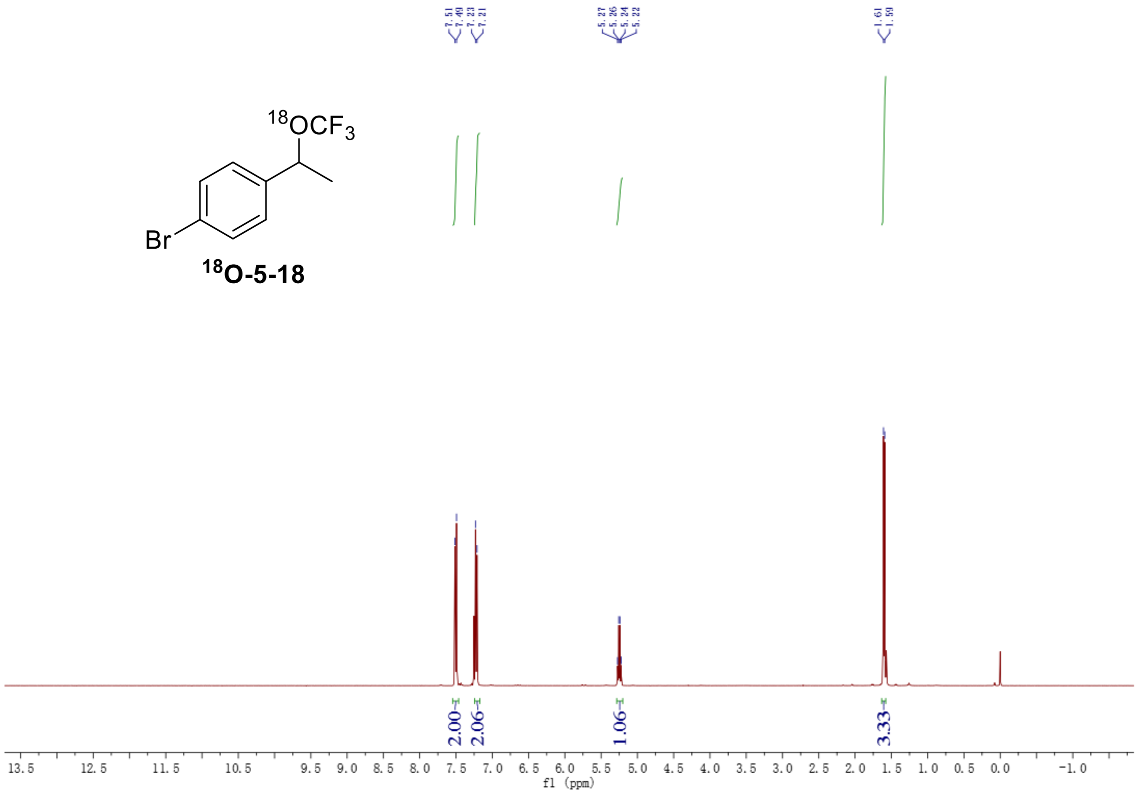

Supplementary Figure 161. ^{1}H NMR spectrum (400 MHz, $CDCl_3$) of **^{18}O-5-18**

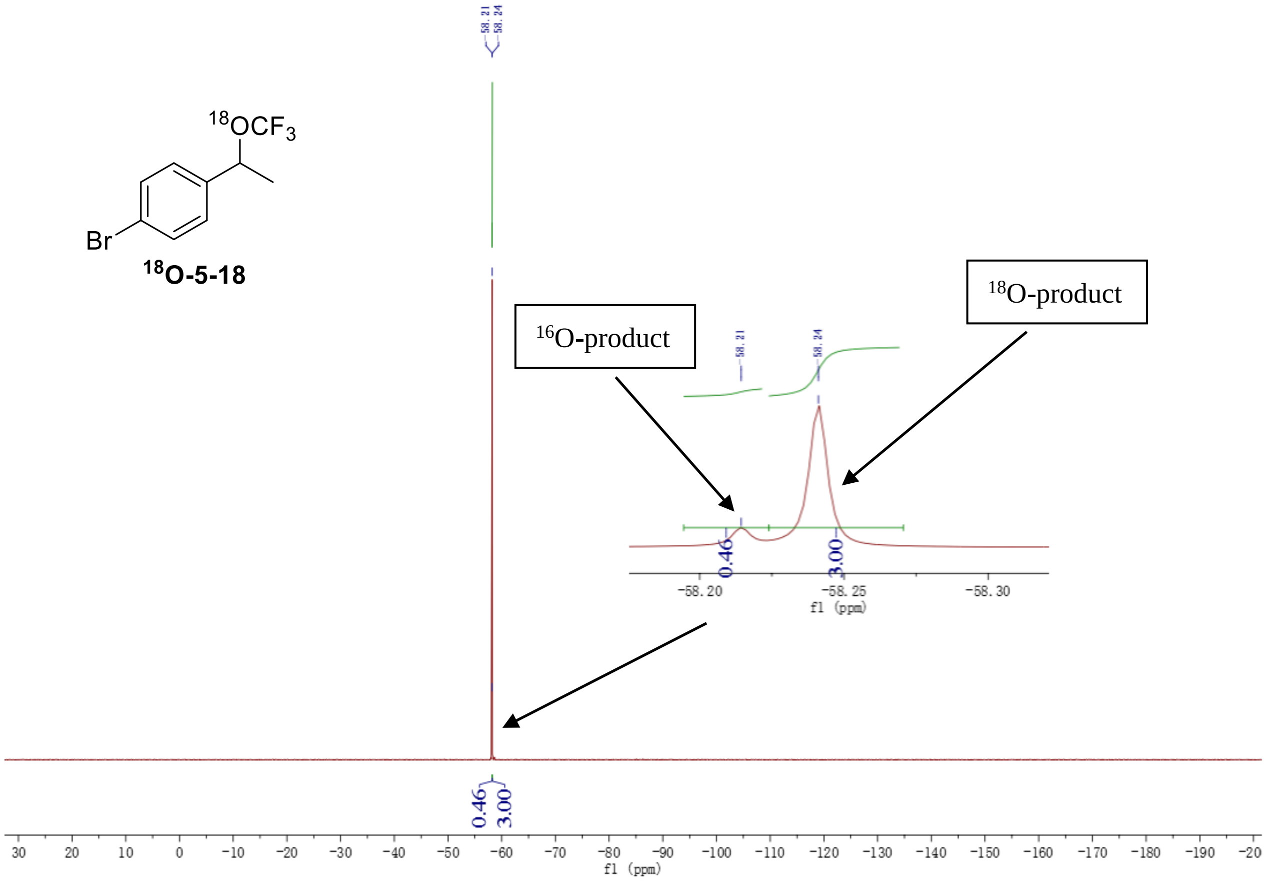

Supplementary Figure 162. ^{19}F NMR spectrum (376 MHz, $CDCl_3$) of **^{18}O-5-18**

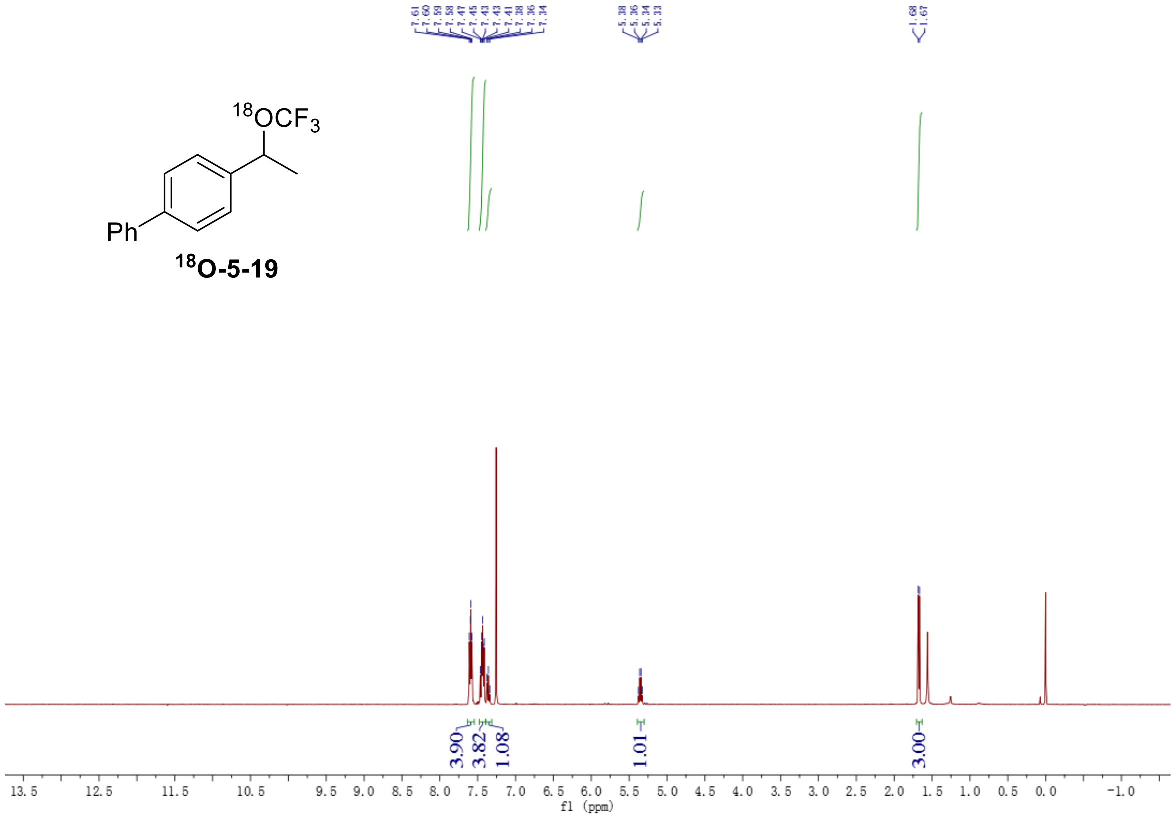

Supplementary Figure 163. ^{1}H NMR spectrum (400 MHz, $CDCl_3$) of **^{18}O-5-19**

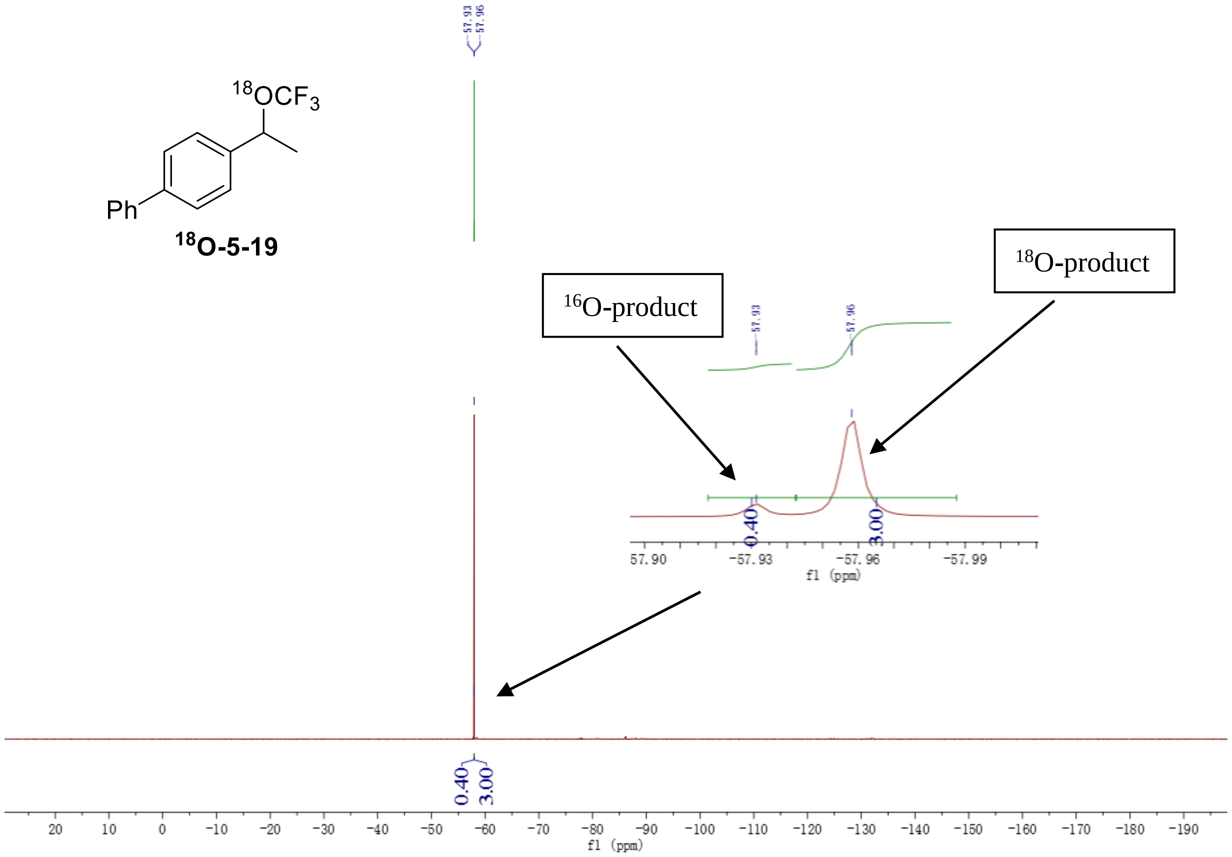

Supplementary Figure 164. ^{19}F NMR spectrum (376 MHz, $CDCl_3$) of **^{18}O-5-19**

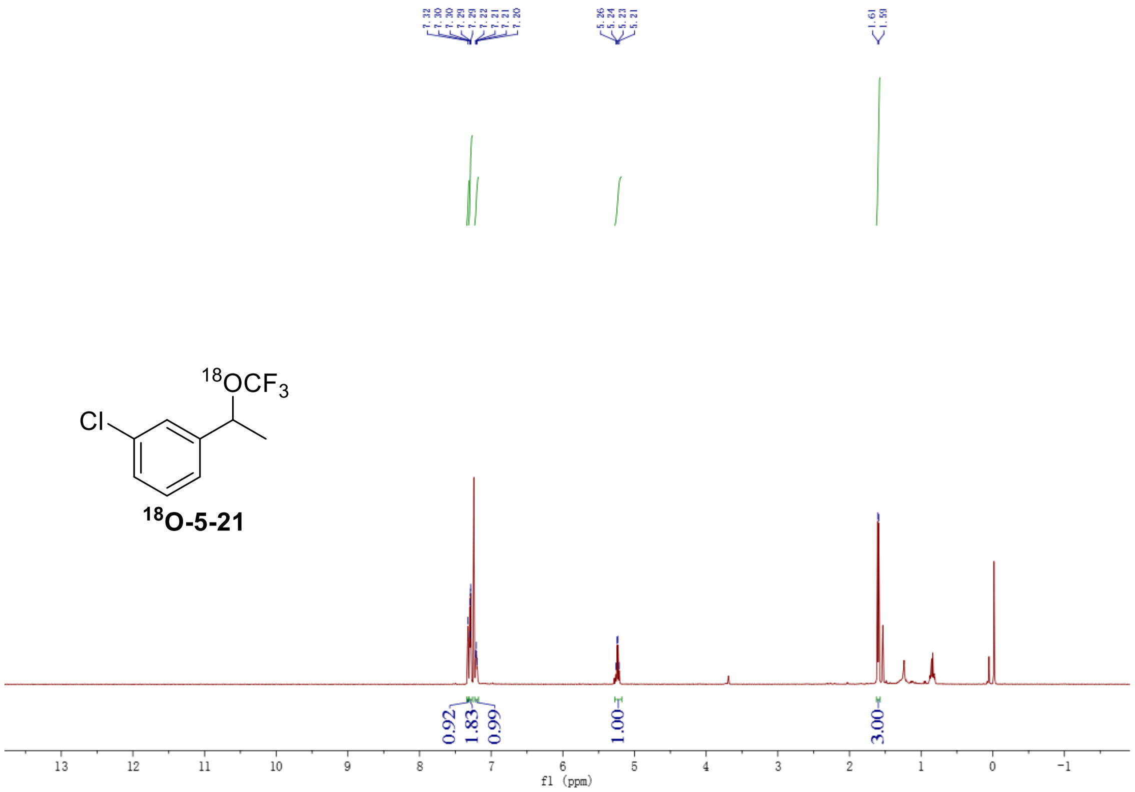

Supplementary Figure 165 ^{1}H NMR spectrum (400 MHz, $CDCl_3$) of **^{18}O-5-21**

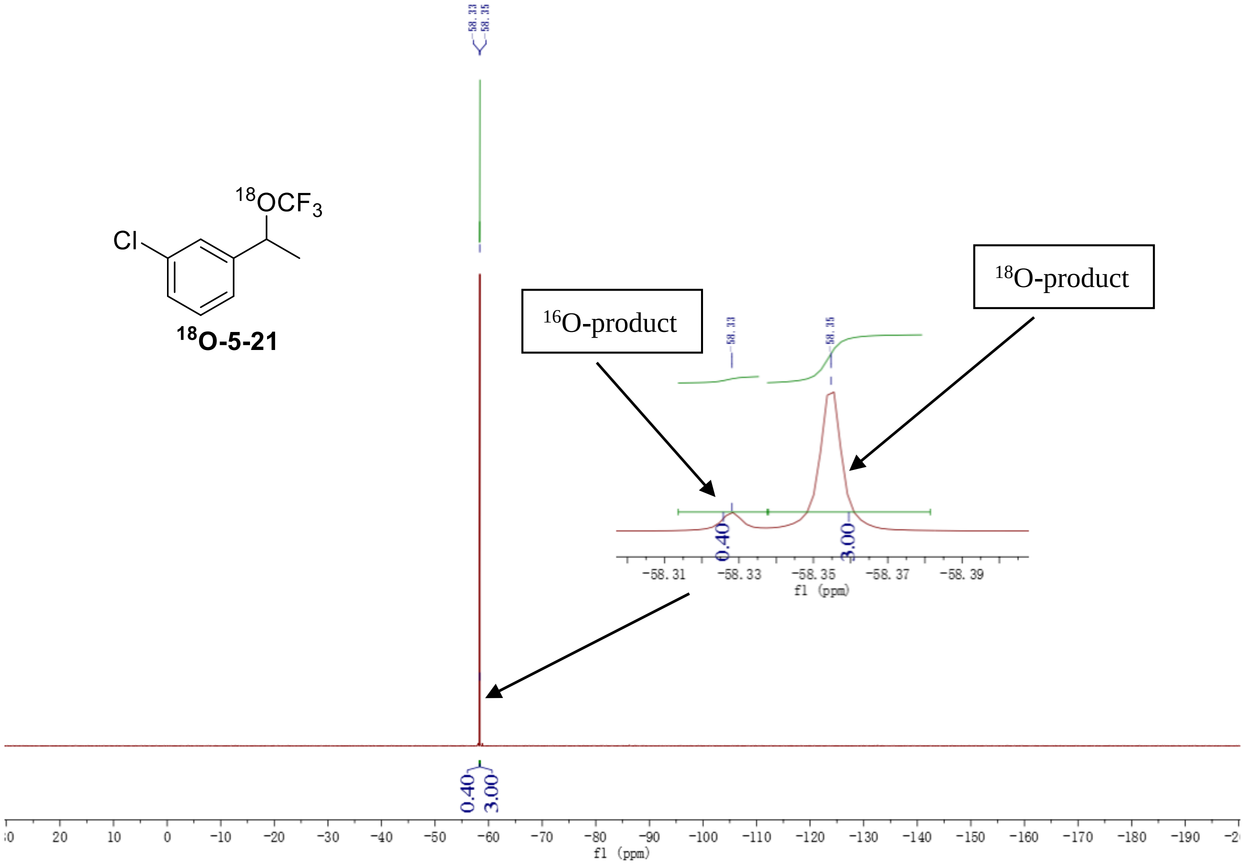

Supplementary Figure 166. ^{19}F NMR spectrum (376 MHz, $CDCl_3$) of **^{18}O-5-21**

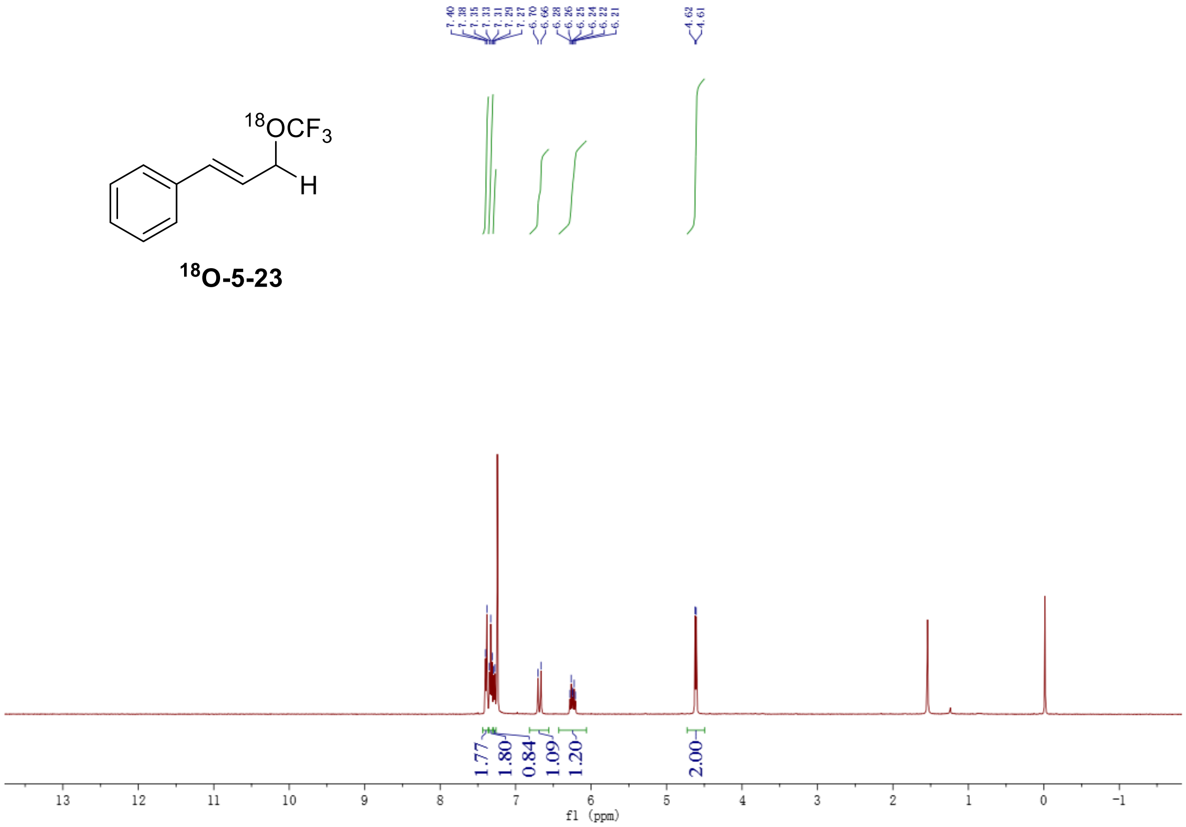

Supplementary Figure 167. ^{1}H NMR spectrum (400 MHz, $CDCl_3$) of **^{18}O-5-23**

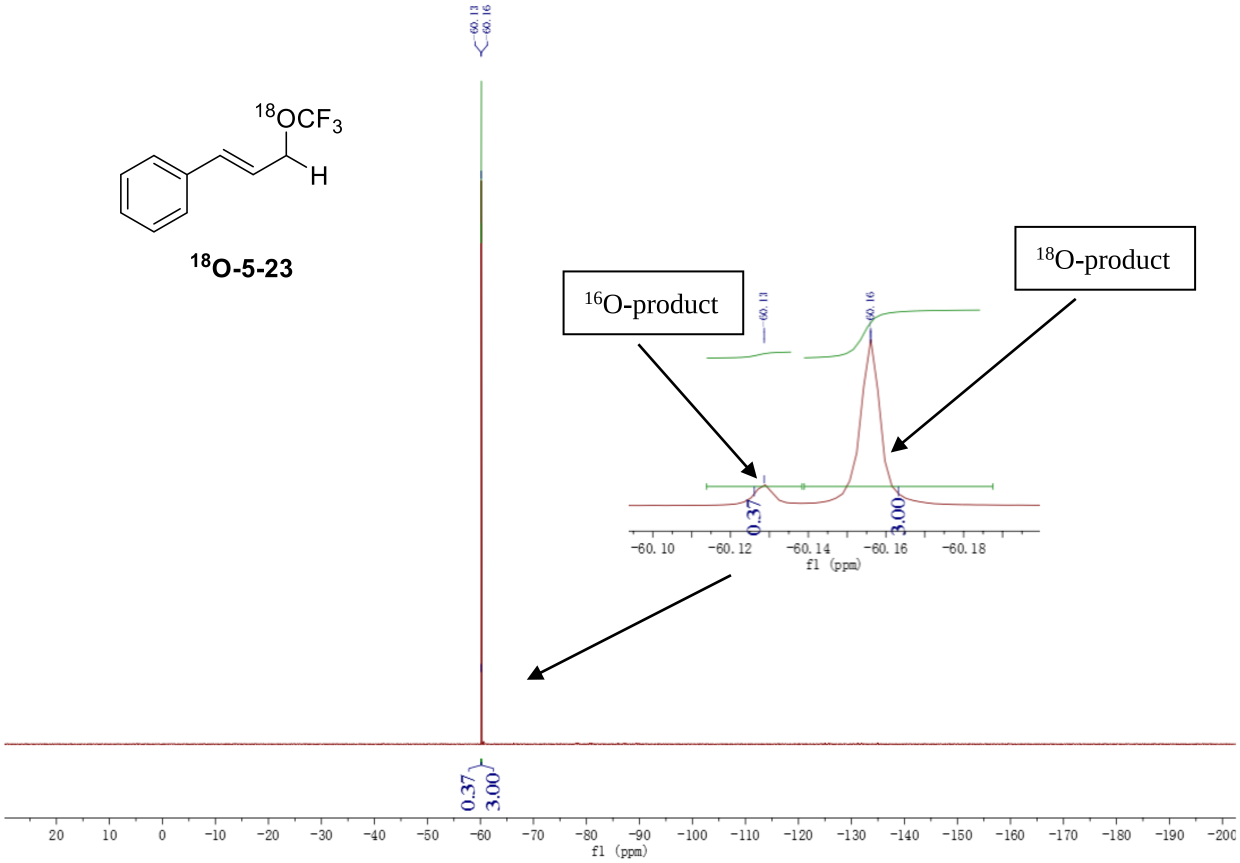

Supplementary Figure 168. ^{19}F NMR spectrum (376 MHz, $CDCl_3$) of **^{18}O-5-23**

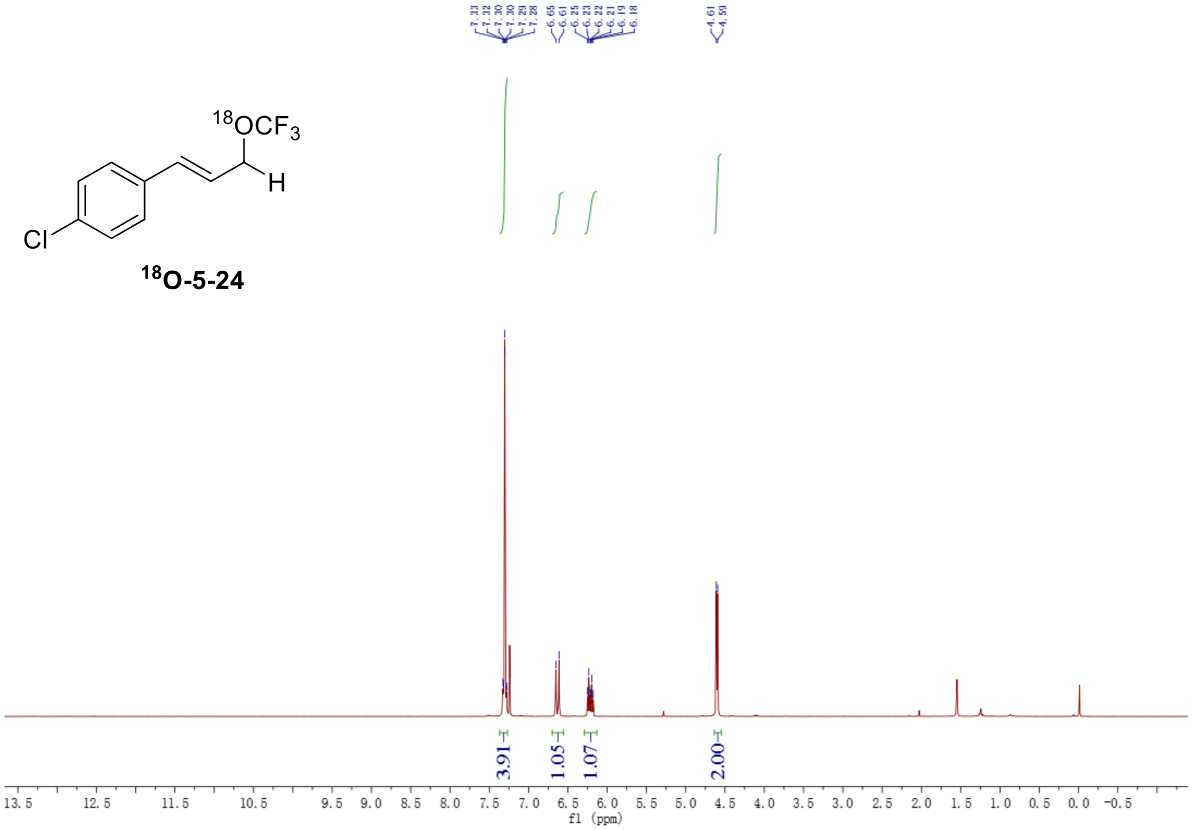

Supplementary Figure 169. ^{1}H NMR spectrum (400 MHz, $CDCl_3$) of **^{18}O-5-24**

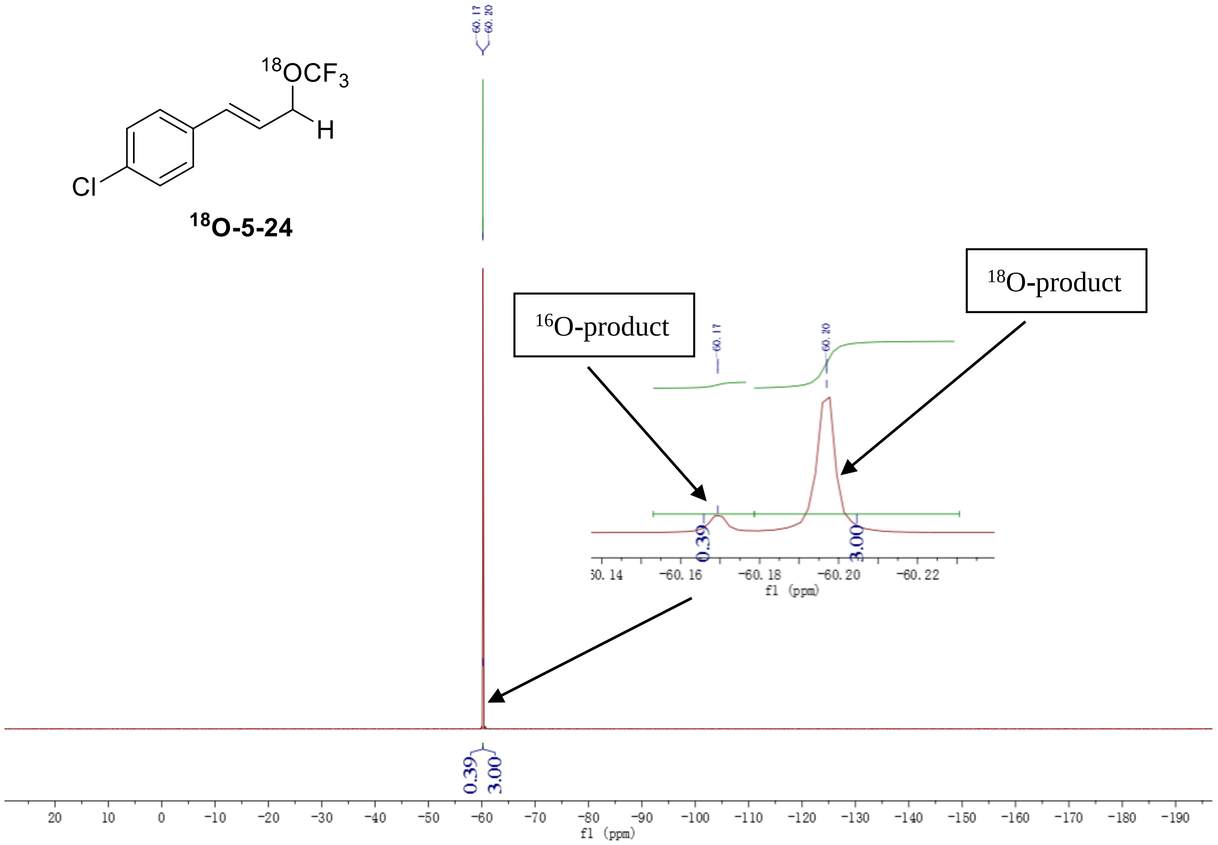

Supplementary Figure 170. ^{19}F NMR spectrum (376 MHz, $CDCl_3$) of **^{18}O-5-24**

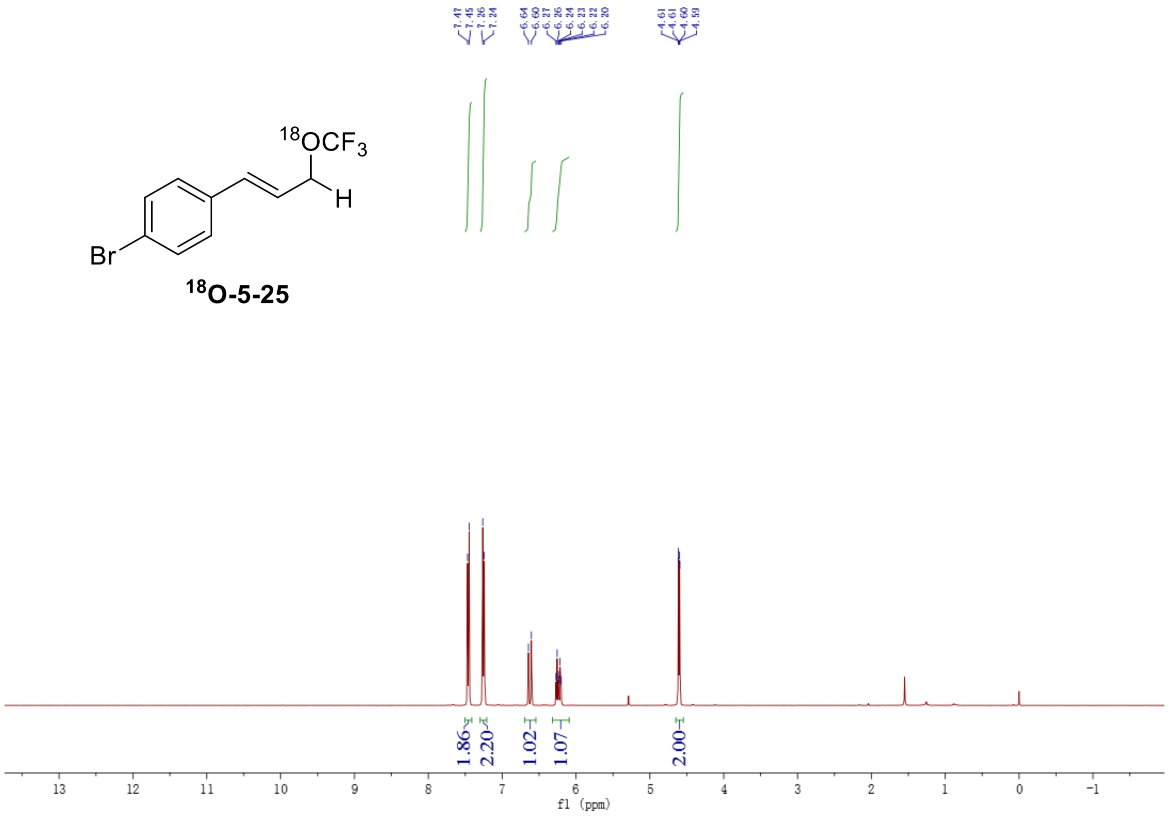

Supplementary Figure 171. ^{1}H NMR spectrum (400 MHz, $CDCl_3$) of **^{18}O-5-25**

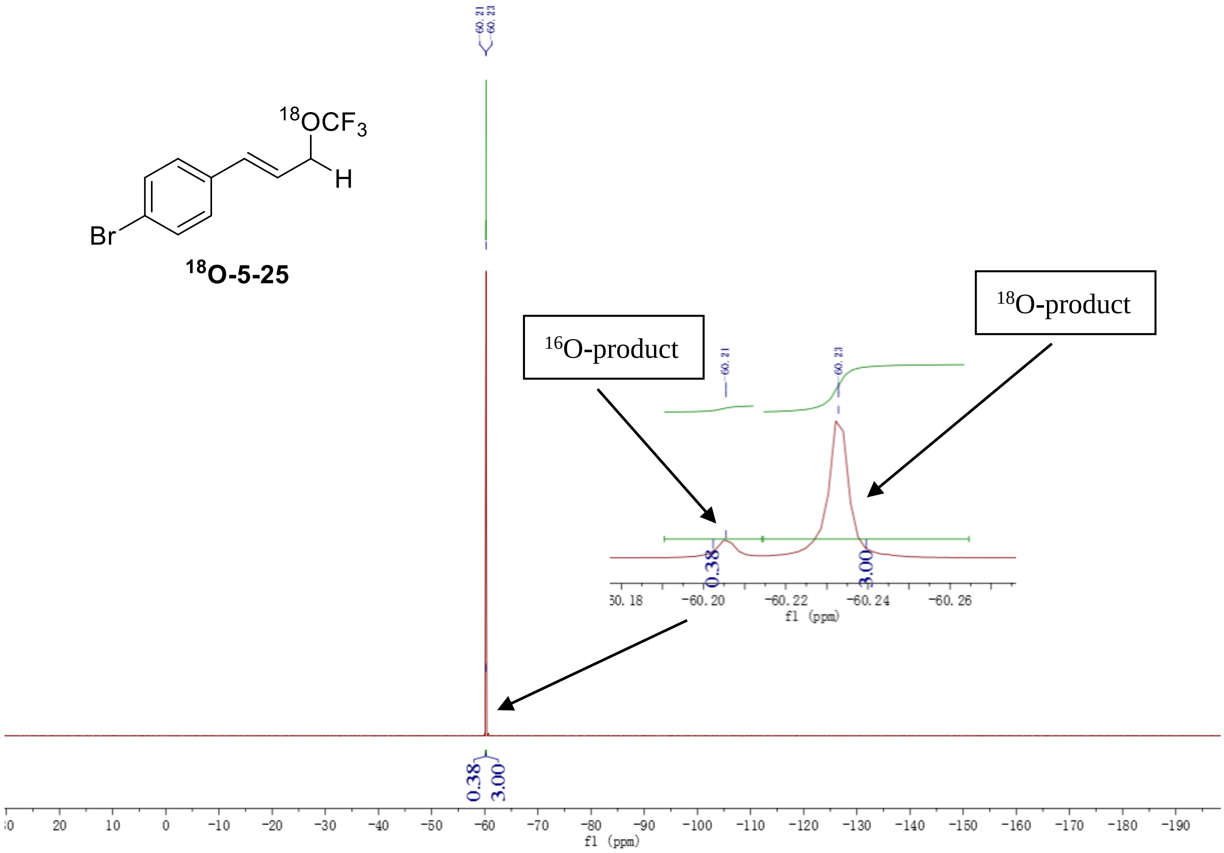

Supplementary Figure 172. ^{19}F NMR spectrum (376 MHz, $CDCl_3$) of **^{18}O-5-25**

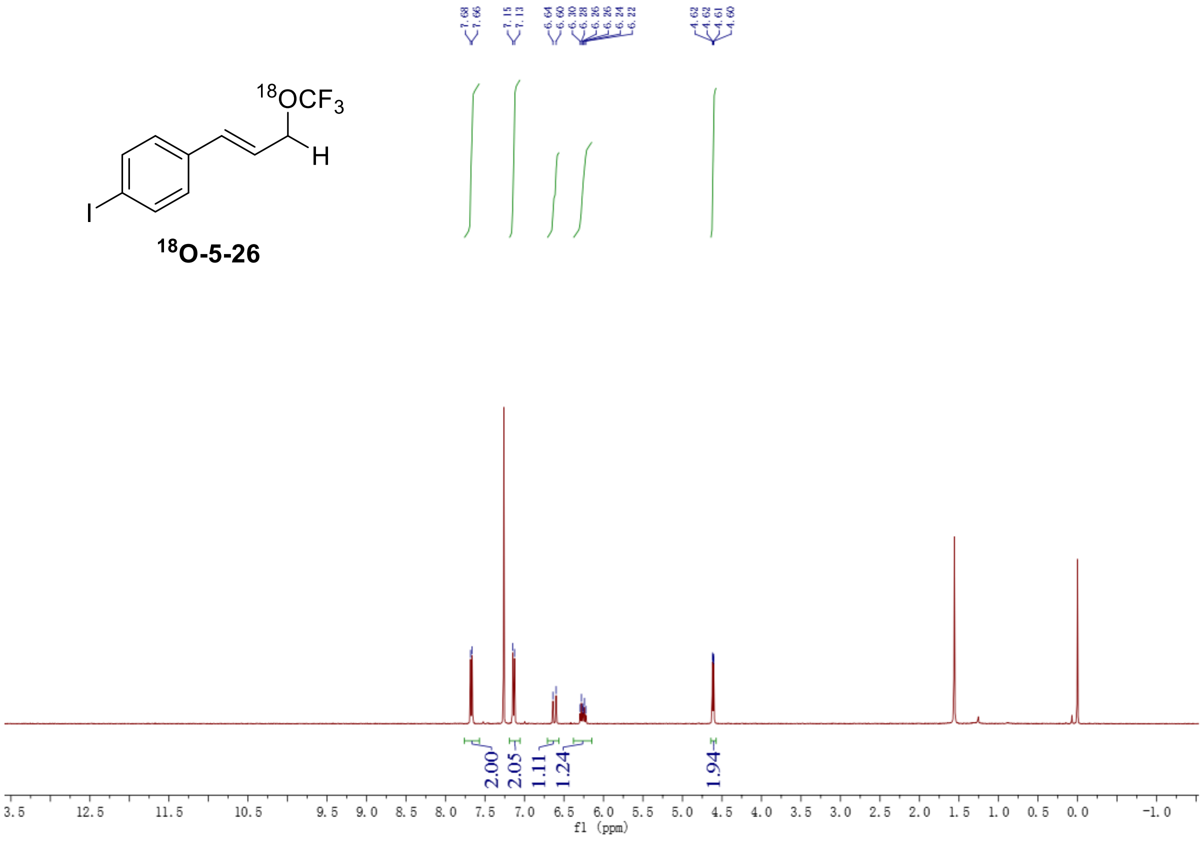

Supplementary Figure 173. ^{1}H NMR spectrum (400 MHz, $CDCl_3$) of **^{18}O-5-26**

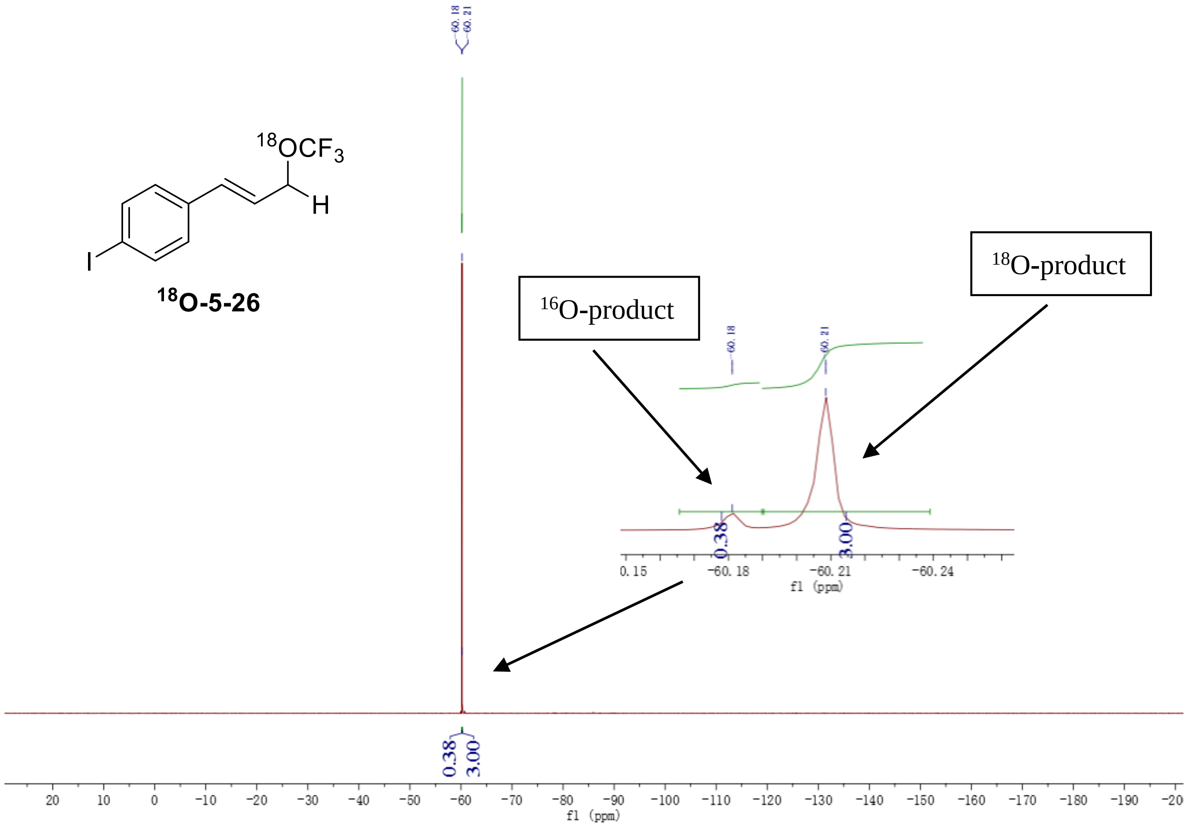

Supplementary Figure 174. ^{19}F NMR spectrum (376 MHz, $CDCl_3$) of **^{18}O-5-26**

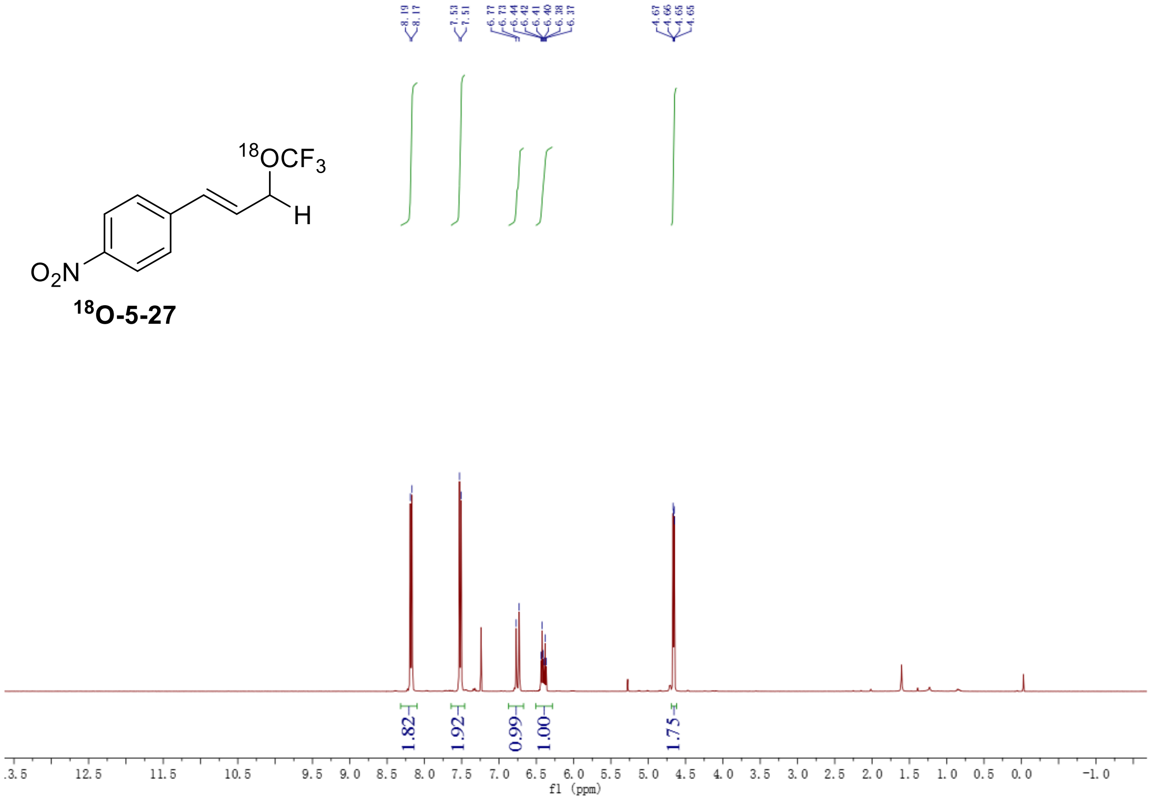

Supplementary Figure 175. ^{1}H NMR spectrum (400 MHz, $CDCl_3$) of **^{18}O-5-27**

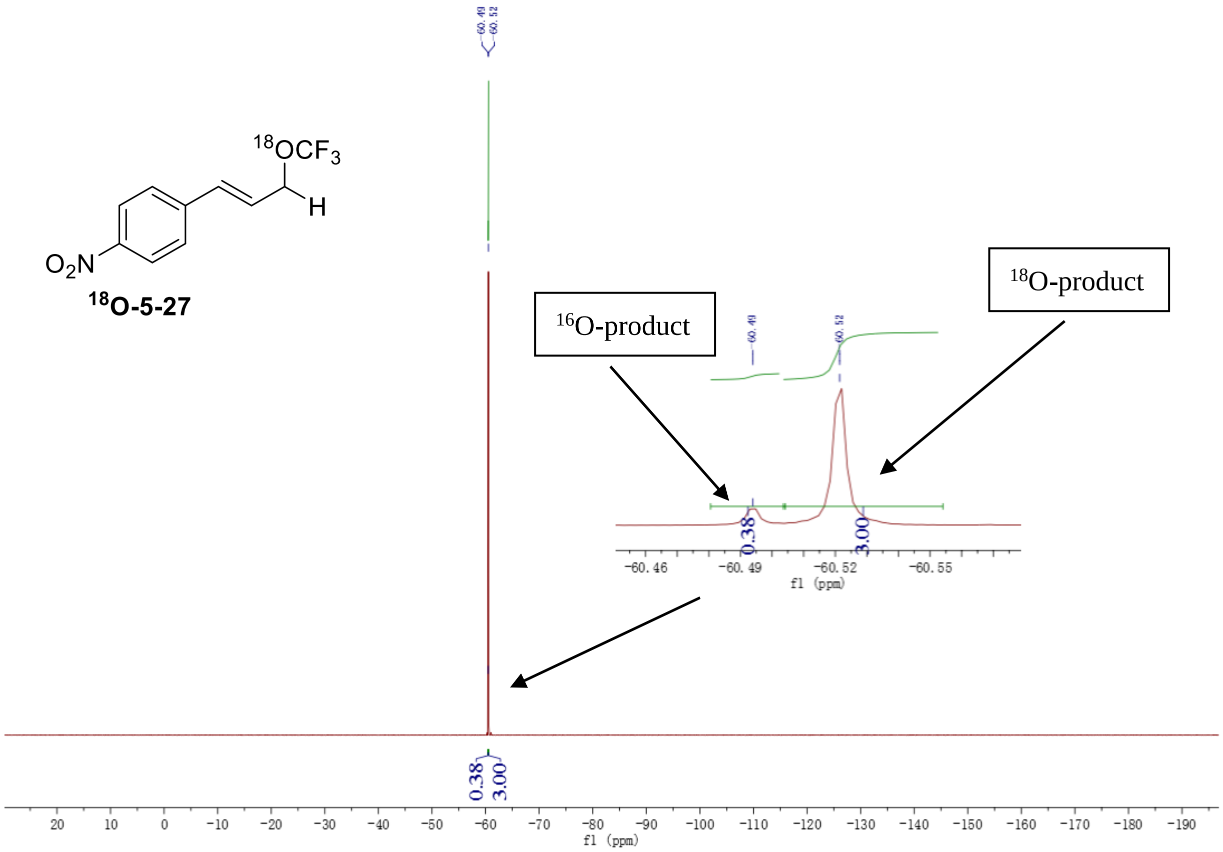

Supplementary Figure 176. ^{19}F NMR spectrum (376 MHz, $CDCl_3$) of **^{18}O-5-27**

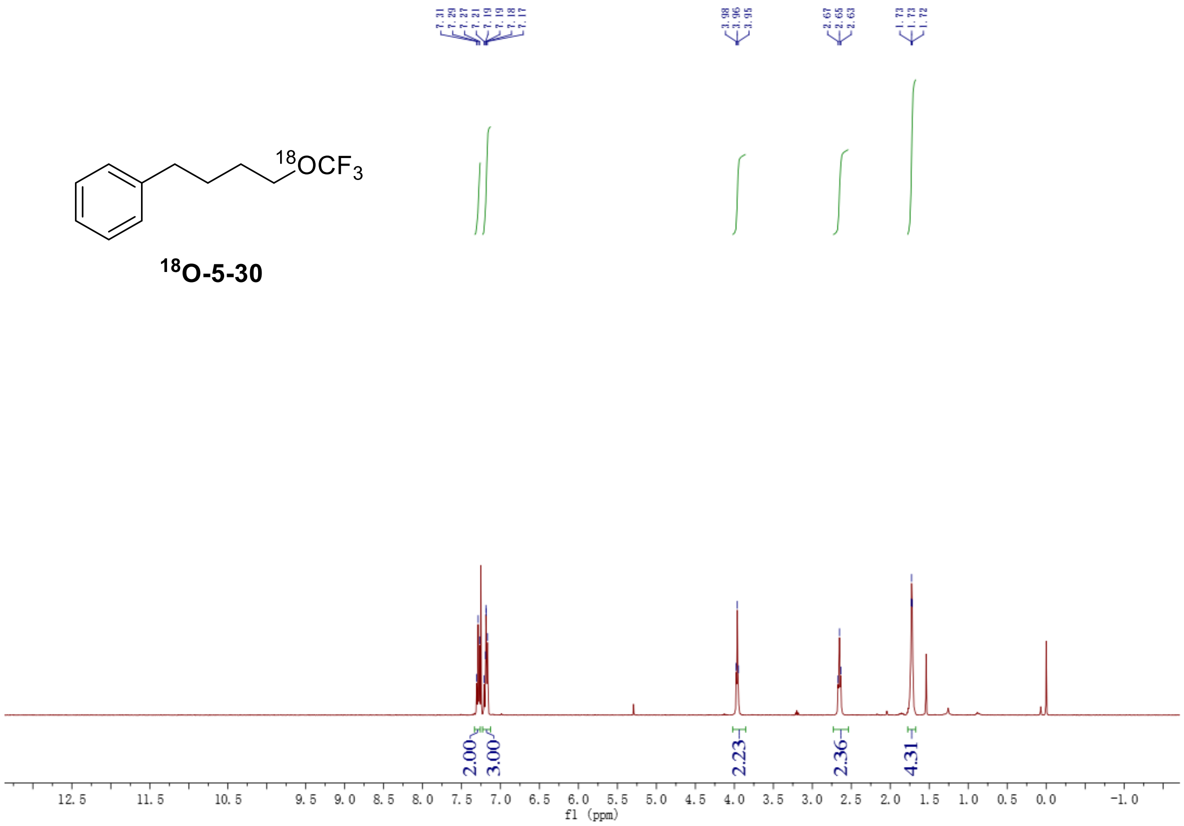

Supplementary Figure 177. ^{1}H NMR spectrum (400 MHz, $CDCl_3$) of **^{18}O-5-30**

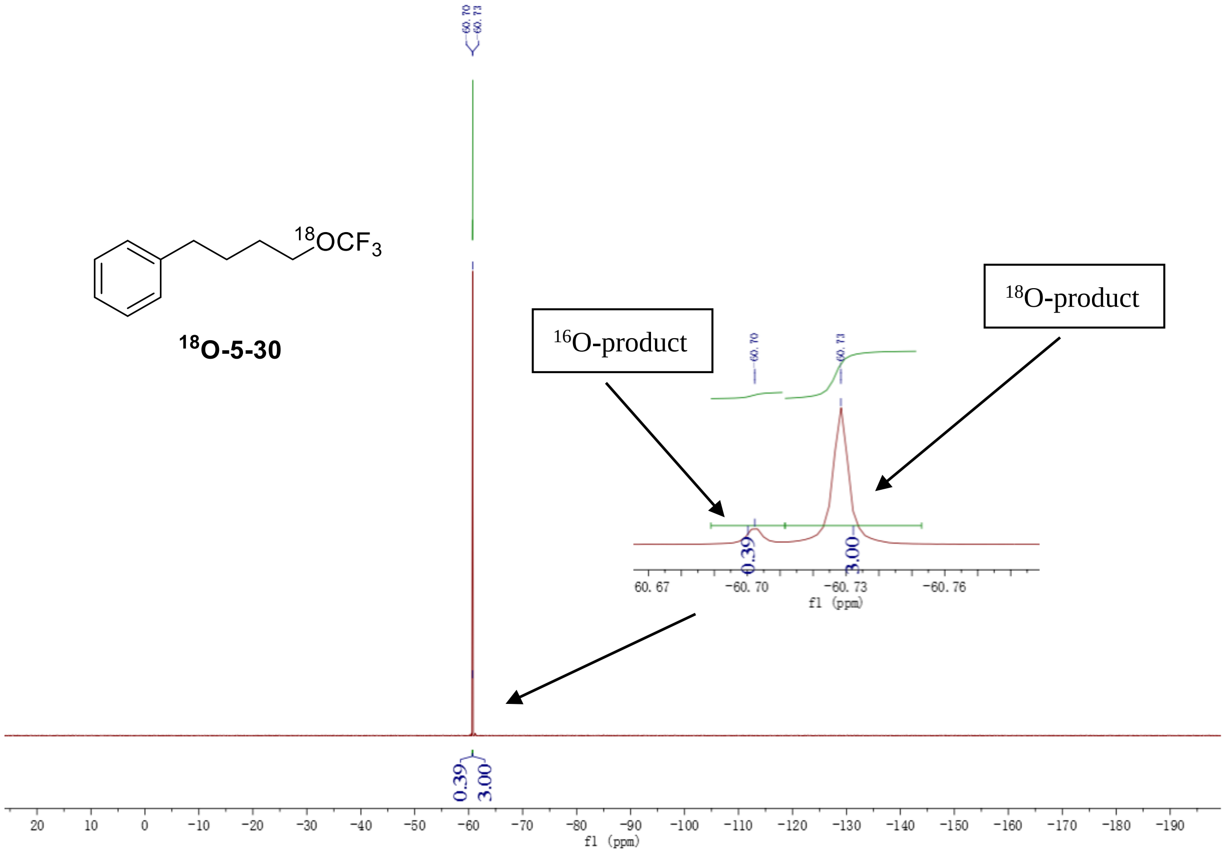

Supplementary Figure 178. ^{19}F NMR spectrum (376 MHz, $CDCl_3$) of **^{18}O-5-30**

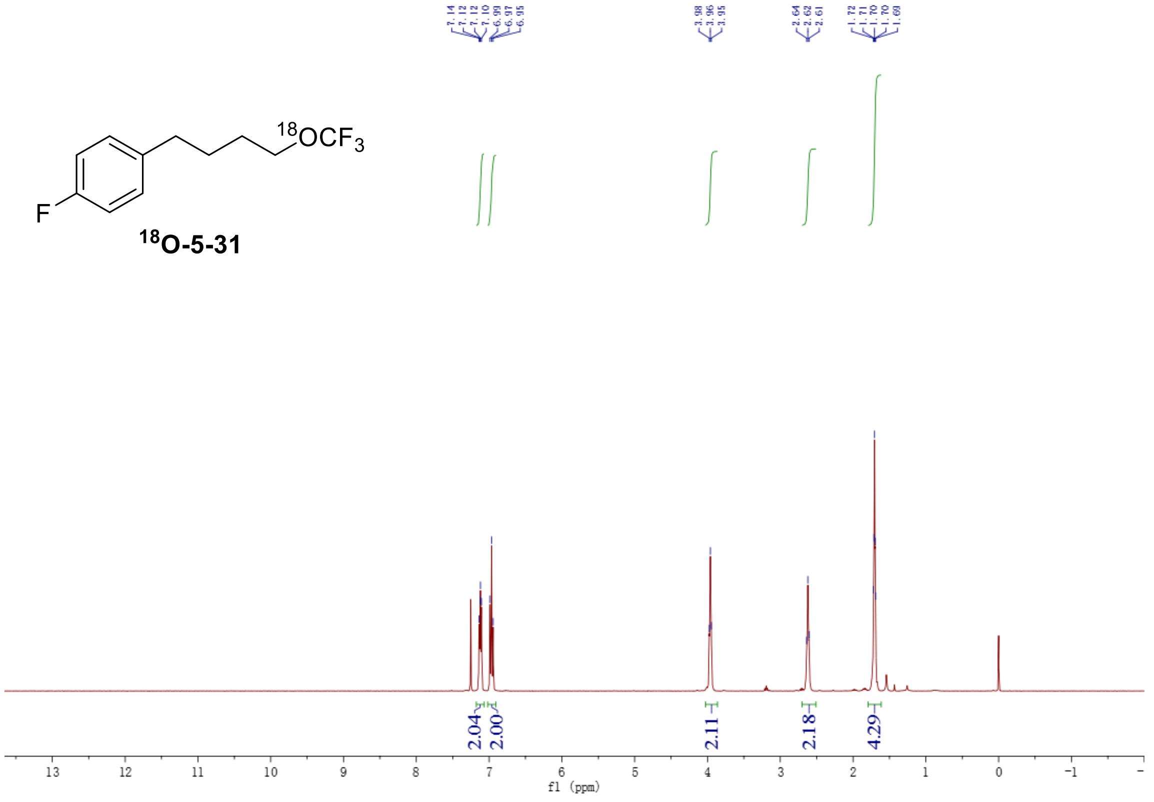

Supplementary Figure 179. ^{1}H NMR spectrum (400 MHz, $CDCl_3$) of **^{18}O-5-31**

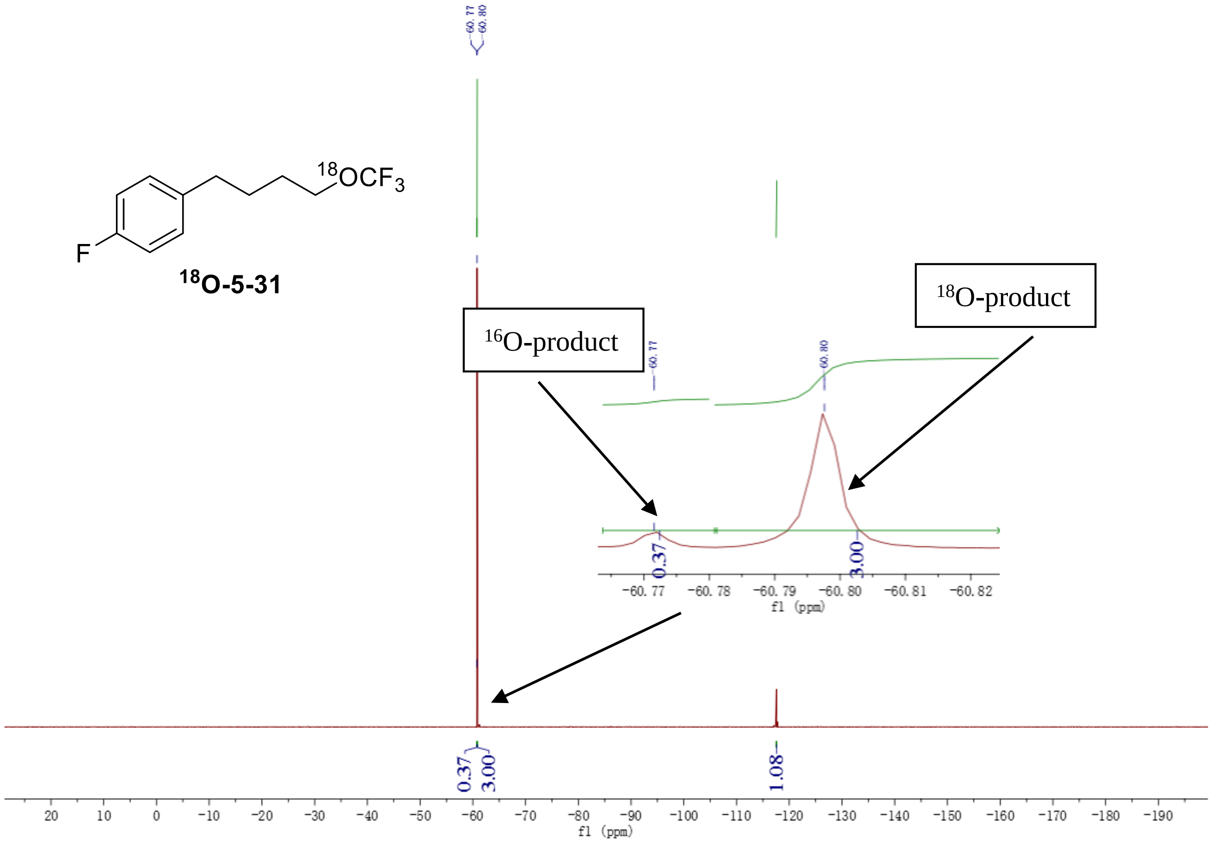

Supplementary Figure 180. ^{19}F NMR spectrum (376 MHz, $CDCl_3$) of **^{18}O-5-31**

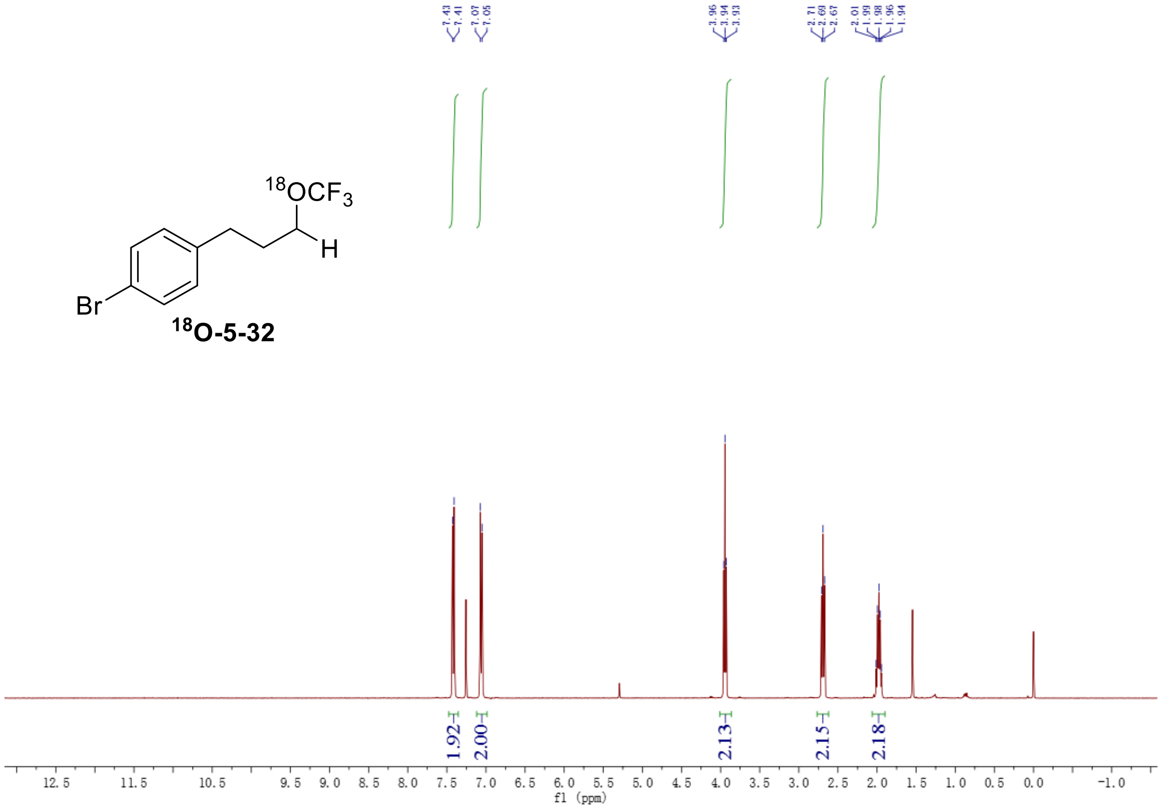

Supplementary Figure 181. ^{1}H NMR spectrum (400 MHz, $CDCl_3$) of **^{18}O-5-32**

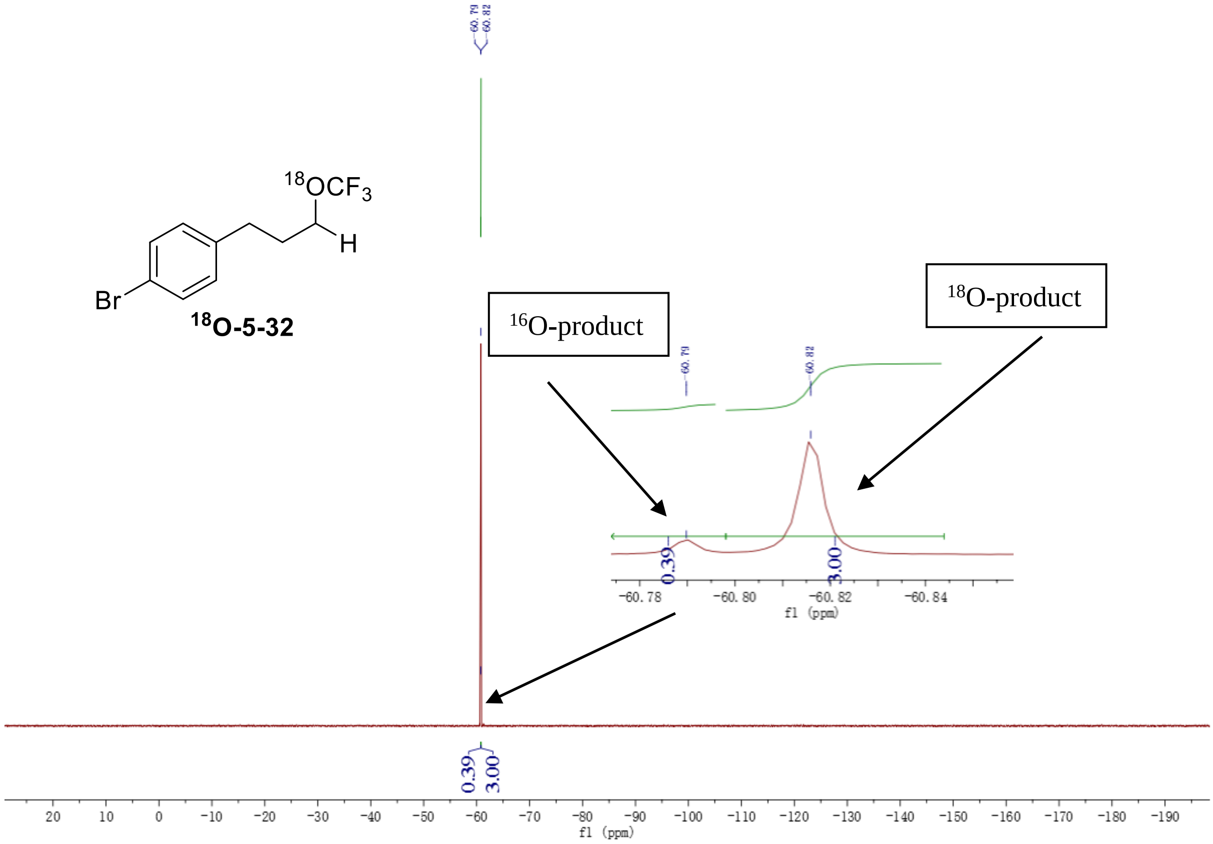

Supplementary Figure 182. ^{19}F NMR spectrum (376 MHz, $CDCl_3$) of **^{18}O-5-32**

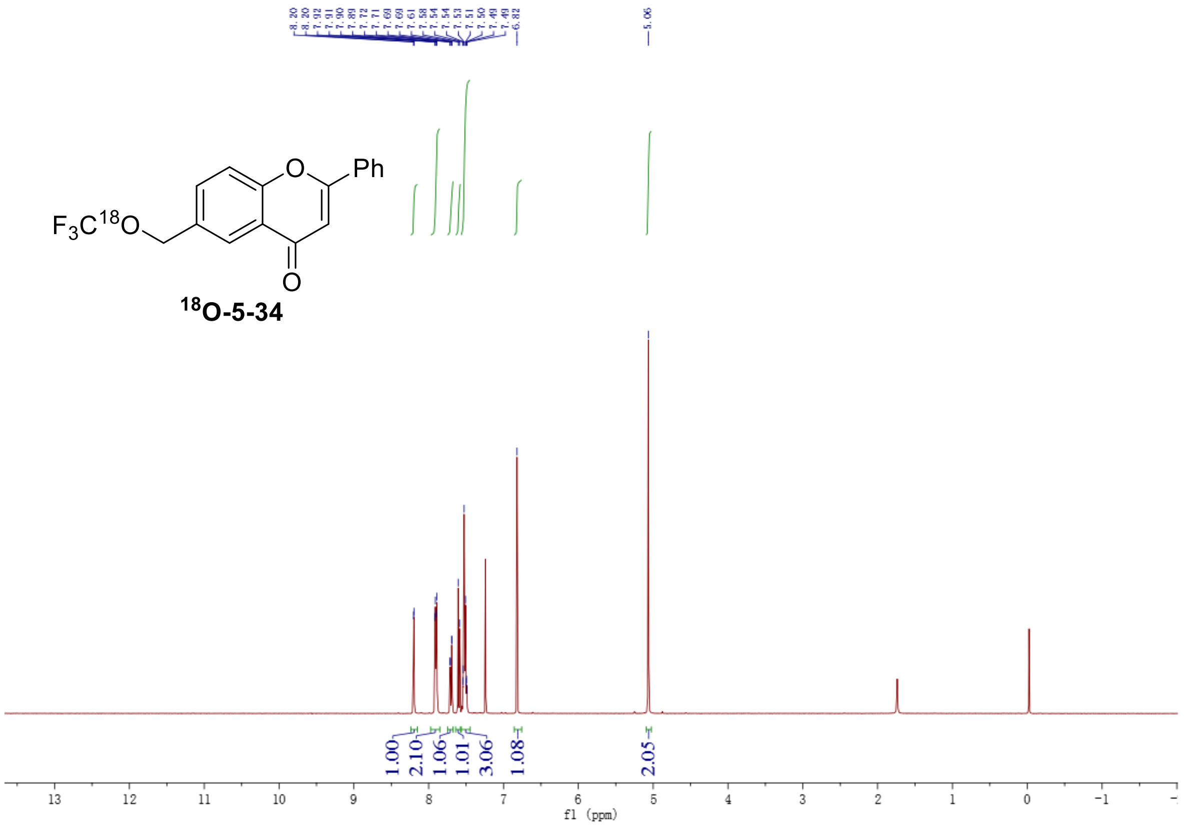

Supplementary Figure 183. ^{1}H NMR spectrum (400 MHz, $CDCl_3$) of **^{18}O-5-34**

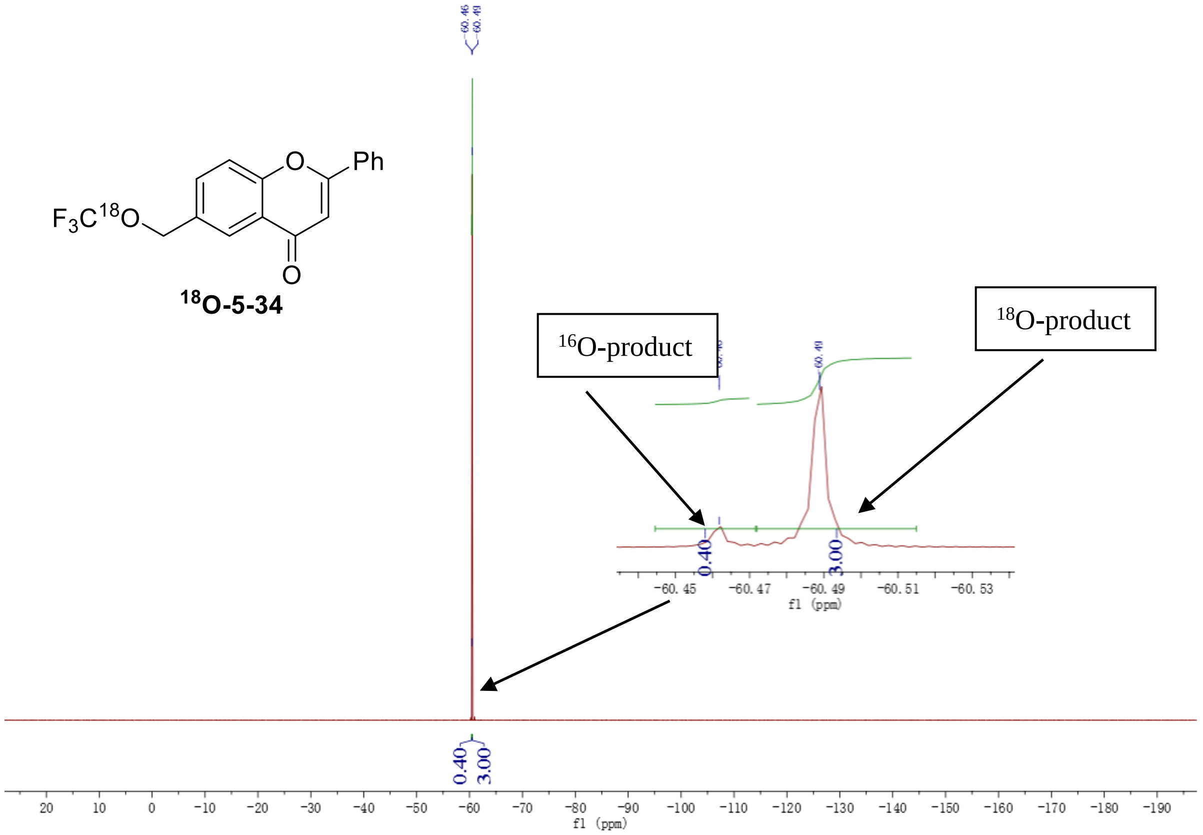

Supplementary Figure 184. ^{19}F NMR spectrum (376 MHz, $CDCl_3$) of **^{18}O-5-34**

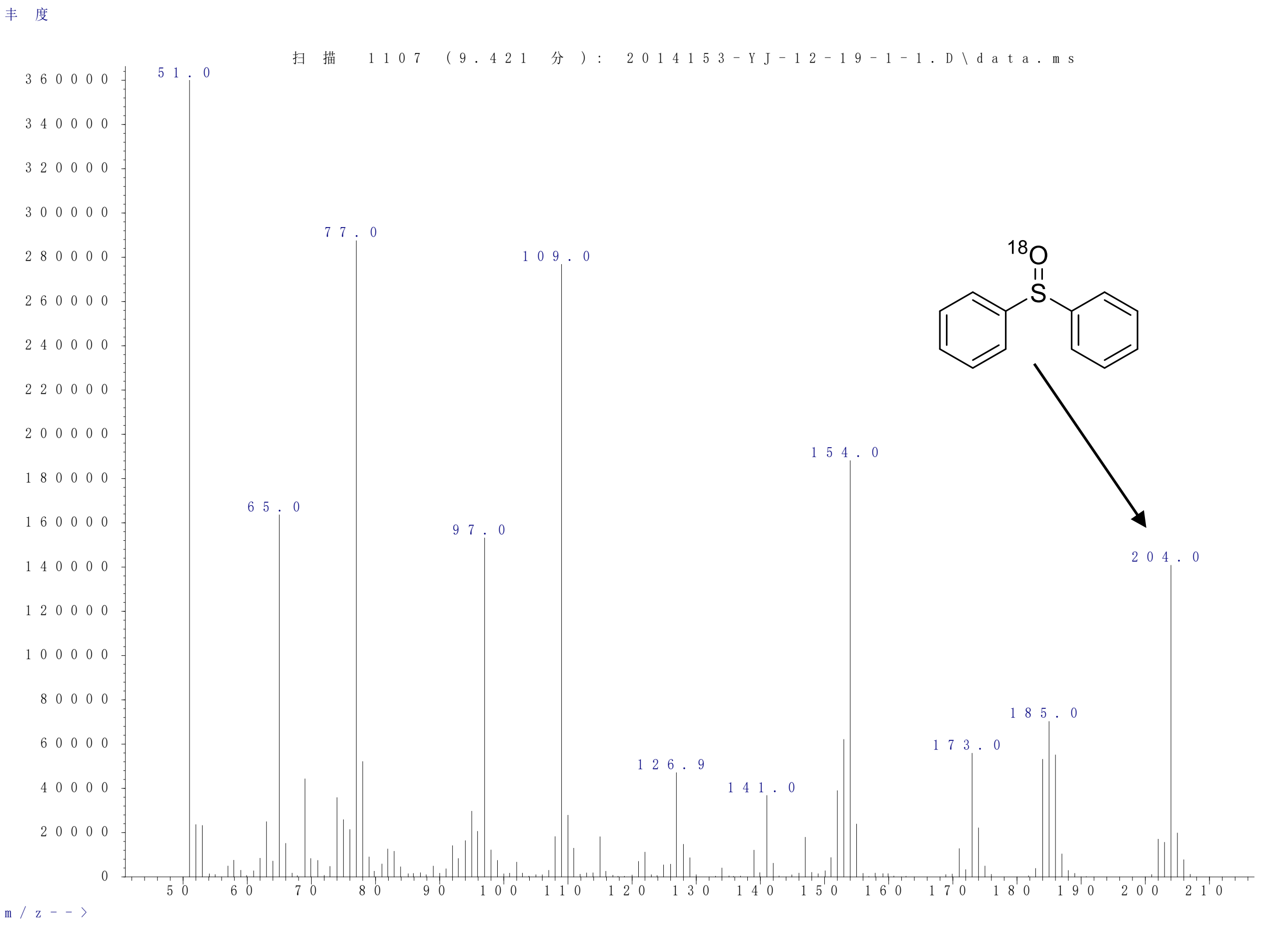

Supplementary Figure 185. EI spectrum of products **^{18}O-diphenyl sulfoxide**

m/z	Abs. Int.
185.00	70264.0
186.00	55104.0
187.00	10395.0
188.00	2892.0
189.00	1597.0
190.80	184.0
201.00	1086.0
202.00	17048.0
203.00	15618.0
204.00	140800.0
205.00	19816.0
206.00	7753.0
207.00	1180.0

^{18}O : ^{16}O = 140800.0 : 17048.0 = 89 : 11

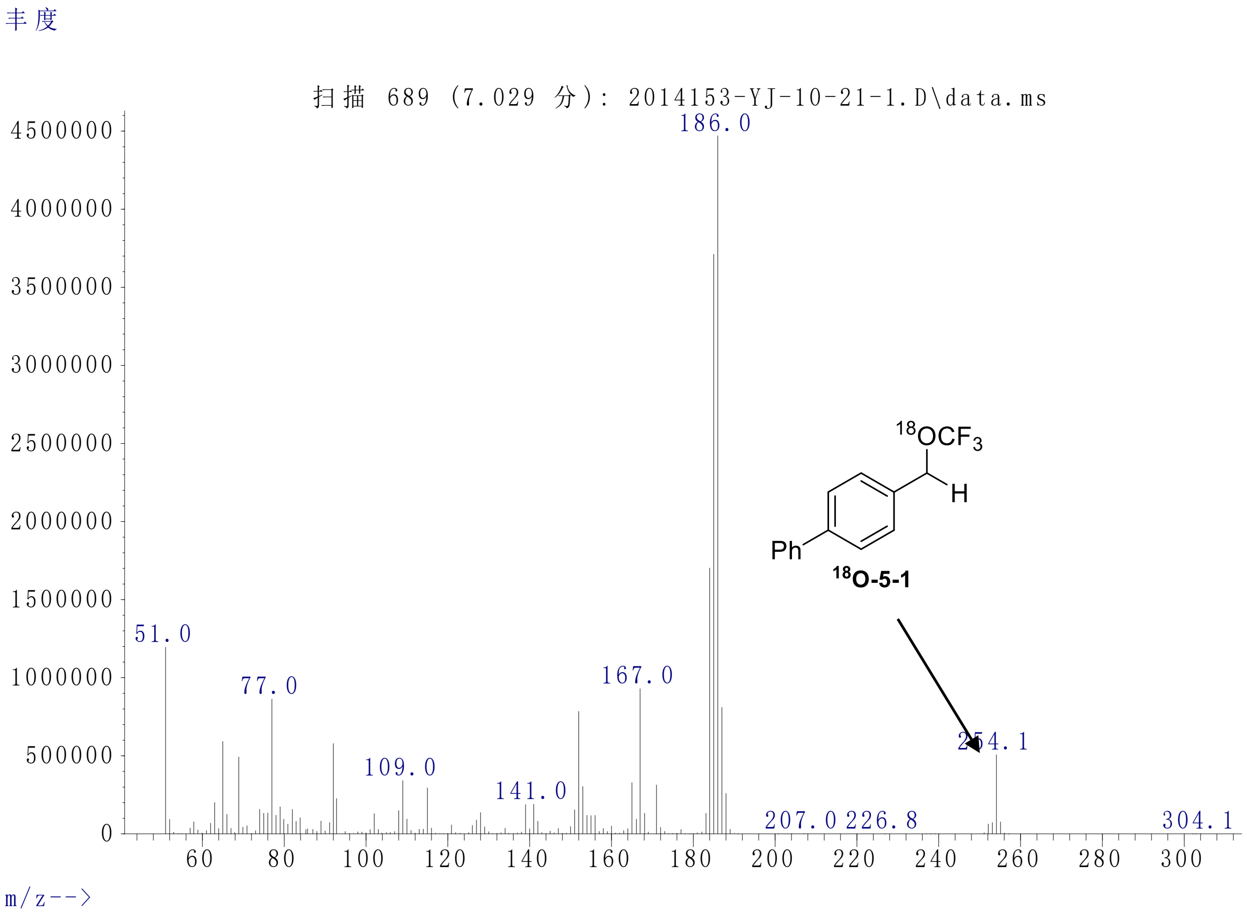

Supplementary Figure 186. EI spectrum of products **^{18}O-5-1**

m/z	Abs. Int.
203.90	533.0
206.90	594.0
208.10	186.0
227.00	221.0
236.90	164.0
239.00	758.0
251.10	6392.0
252.10	58584.0
253.10	68896.0
254.10	471744.0
255.10	71656.0
256.10	5163.0
257.10	300.0
302.10	389.0
303.90	454.0

^{18}O : ^{16}O = 471744.0 : 58584.0 = 89 : 11

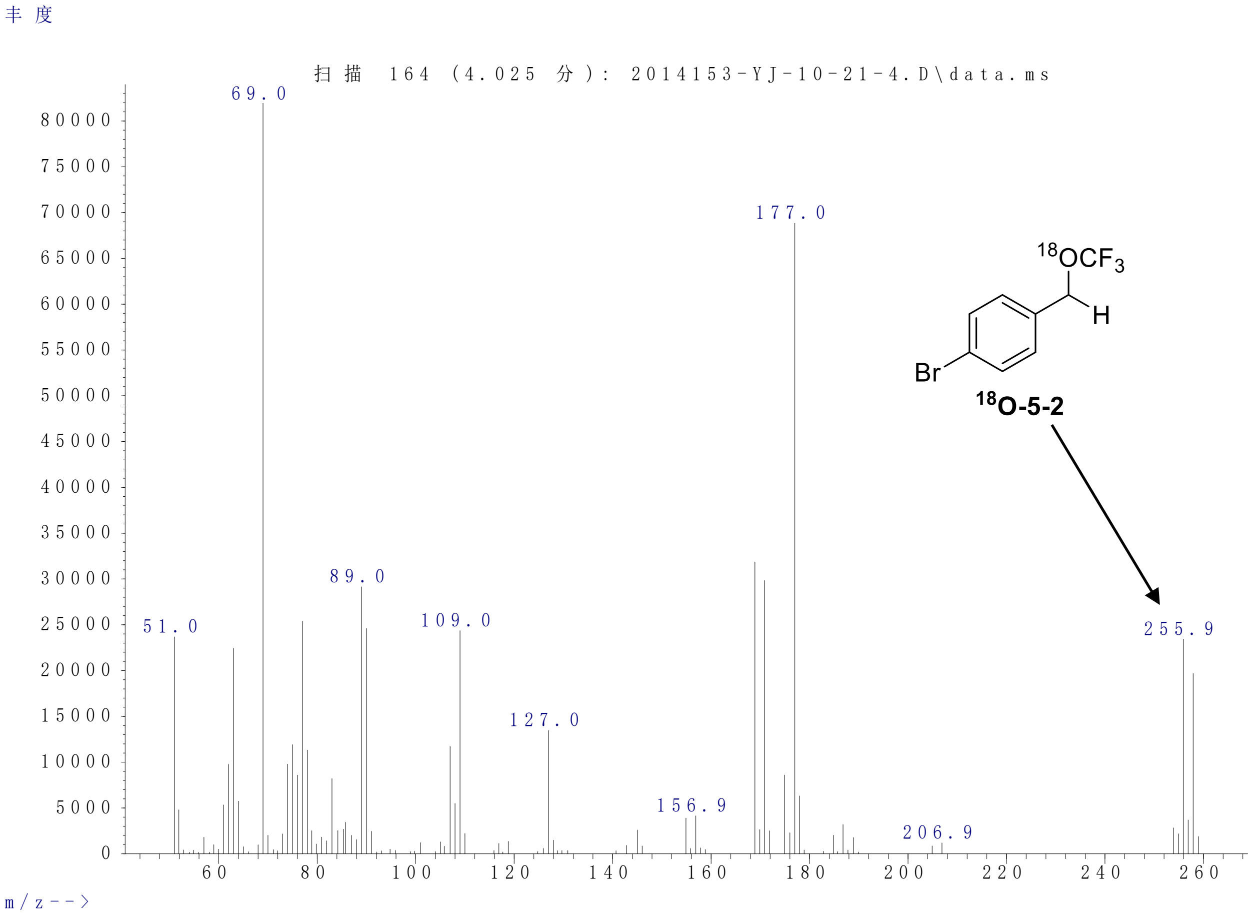

Supplementary Figure 187. EI spectrum of products ^{18}**O-5-2**

m/z	Abs. Int.
187.70	217.0
189.00	1592.0
204.80	701.0
207.00	1117.0
252.80	157.0
253.90	2315.0
254.80	1938.0
255.90	23992.0
256.90	3425.0
257.90	18368.0
258.90	1680.0

^{18}O : ^{16}O = 23992.0 : 2315.0 = 91 : 9

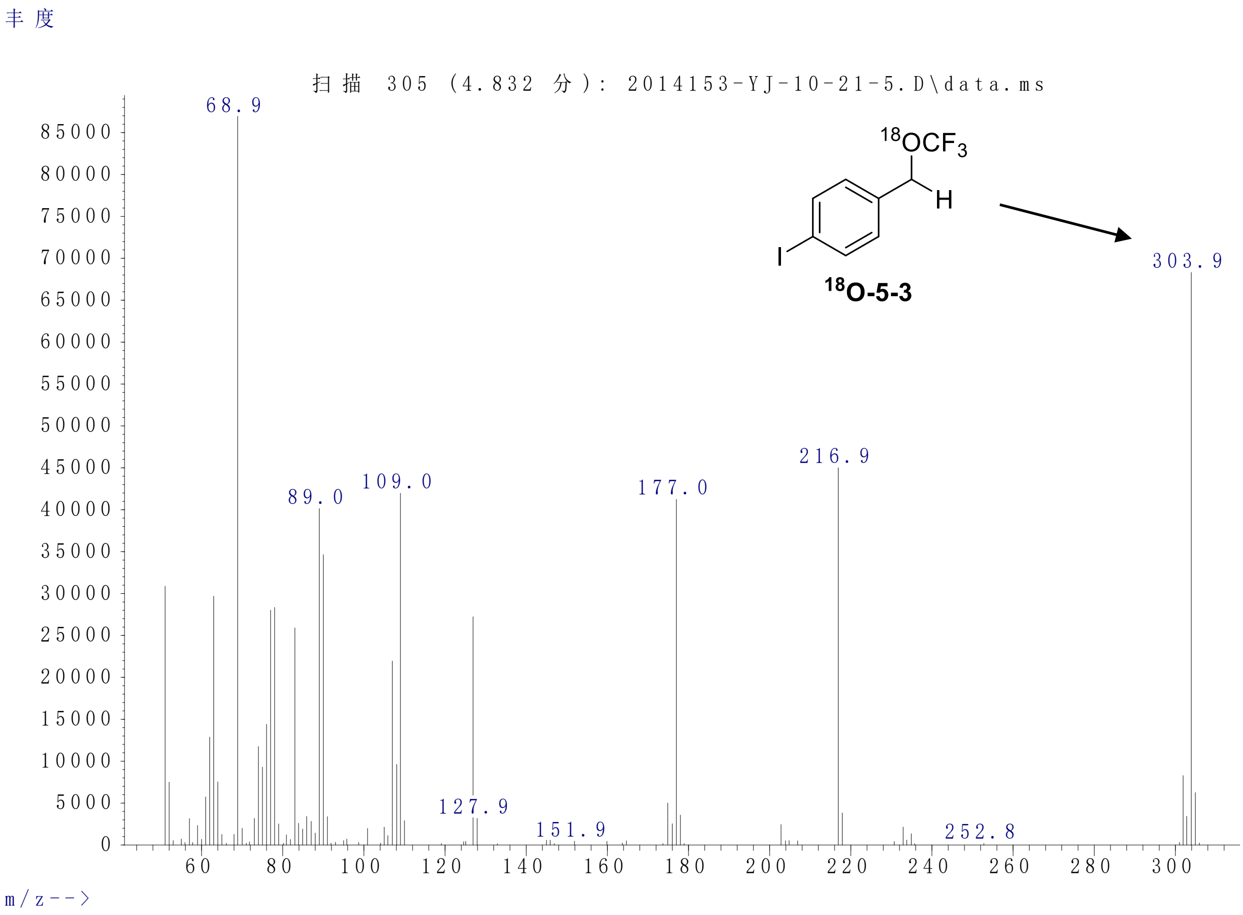

Supplementary Figure 188. EI spectrum of products ^{18}**O-5-3**

m/z	Abs. Int.
216.90	57488.0
217.90	4083.0
218.80	197.0
230.70	425.0
231.90	202.0
232.90	3163.0
233.90	740.0
234.90	1797.0
235.80	203.0
252.80	202.0
300.80	363.0
301.90	11003.0
302.90	5083.0
303.90	87528.0
304.90	7566.0
305.80	416.0

^{18}O : ^{16}O = 87528.0 : 11003.0 = 89 : 11

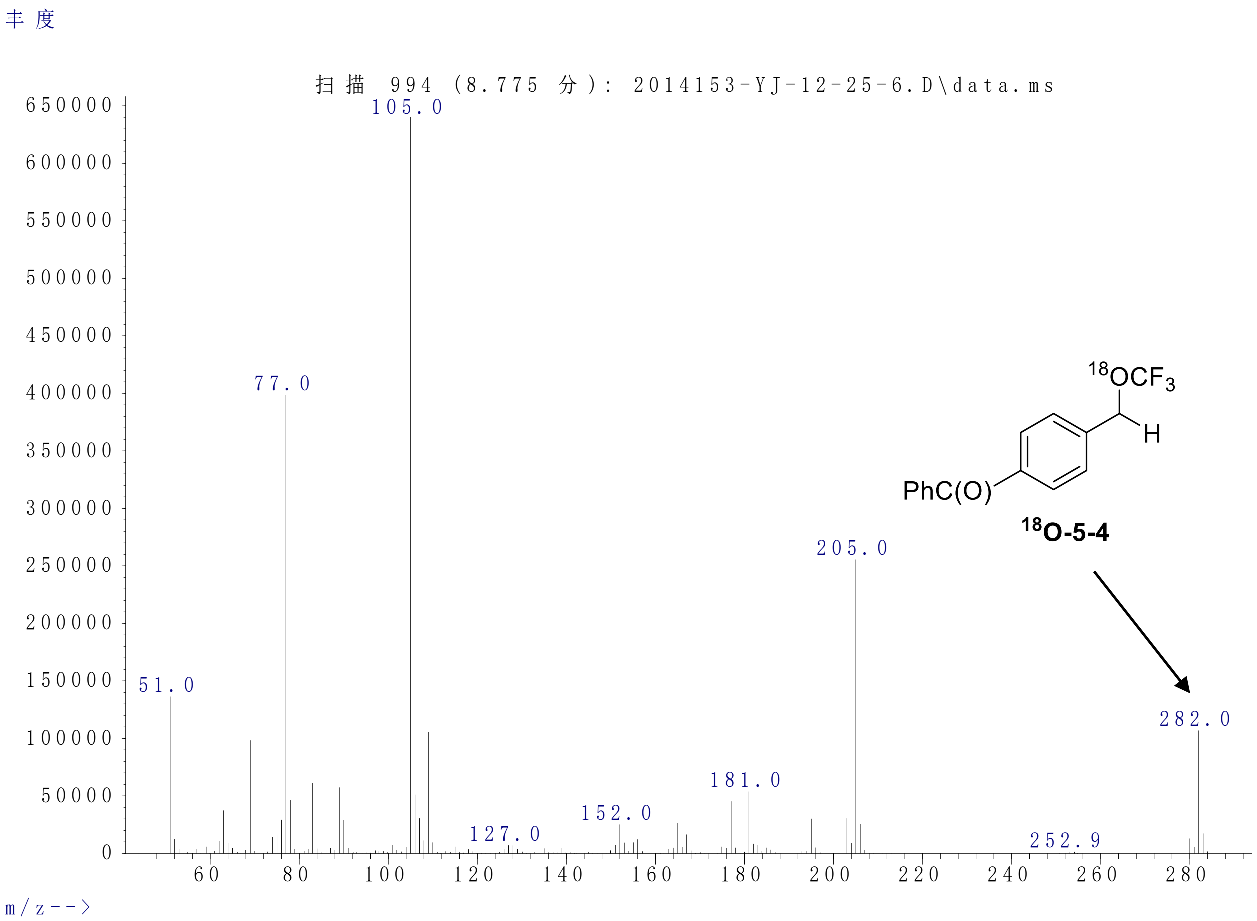

Supplementary Figure 189. EI spectrum of products **^{18}O-5-4**

m/z	Abs. Int.
209.10	156.0
210.80	321.0
213.10	219.0
213.80	203.0
251.00	220.0
252.00	253.0
253.00	1326.0
254.00	1262.0
255.10	279.0
280.00	13914.0
281.00	5729.0
282.00	111912.0
283.10	18136.0
283.90	1697.0
285.20	193.0

^{18}O : ^{16}O = 111912.0 : 13914.0 = 89 : 11

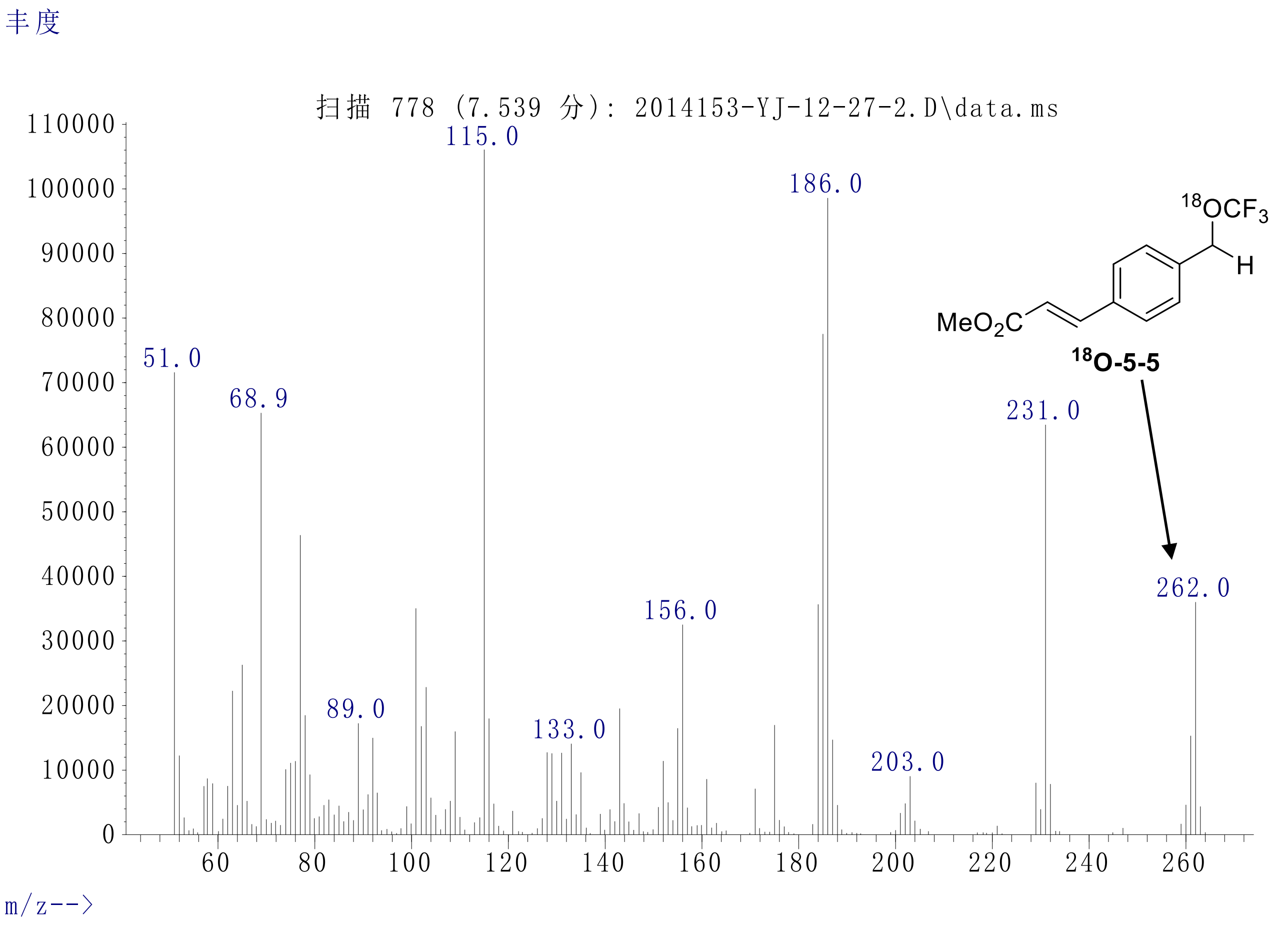

Supplementary Figure 190. EI spectrum of products **^{18}O-5-5**

m/z	Abs. Int.
222.00	165.0
229.00	8013.0
230.00	3907.0
231.00	63440.0
232.00	7814.0
233.10	546.0
233.90	505.0
244.90	325.0
247.00	1008.0
259.00	1652.0
260.00	4607.0
261.00	15295.0
262.00	35984.0
263.00	4341.0
264.00	348.0

^{18}O : ^{16}O = 35984.0 : 4607.0 = 89 : 11

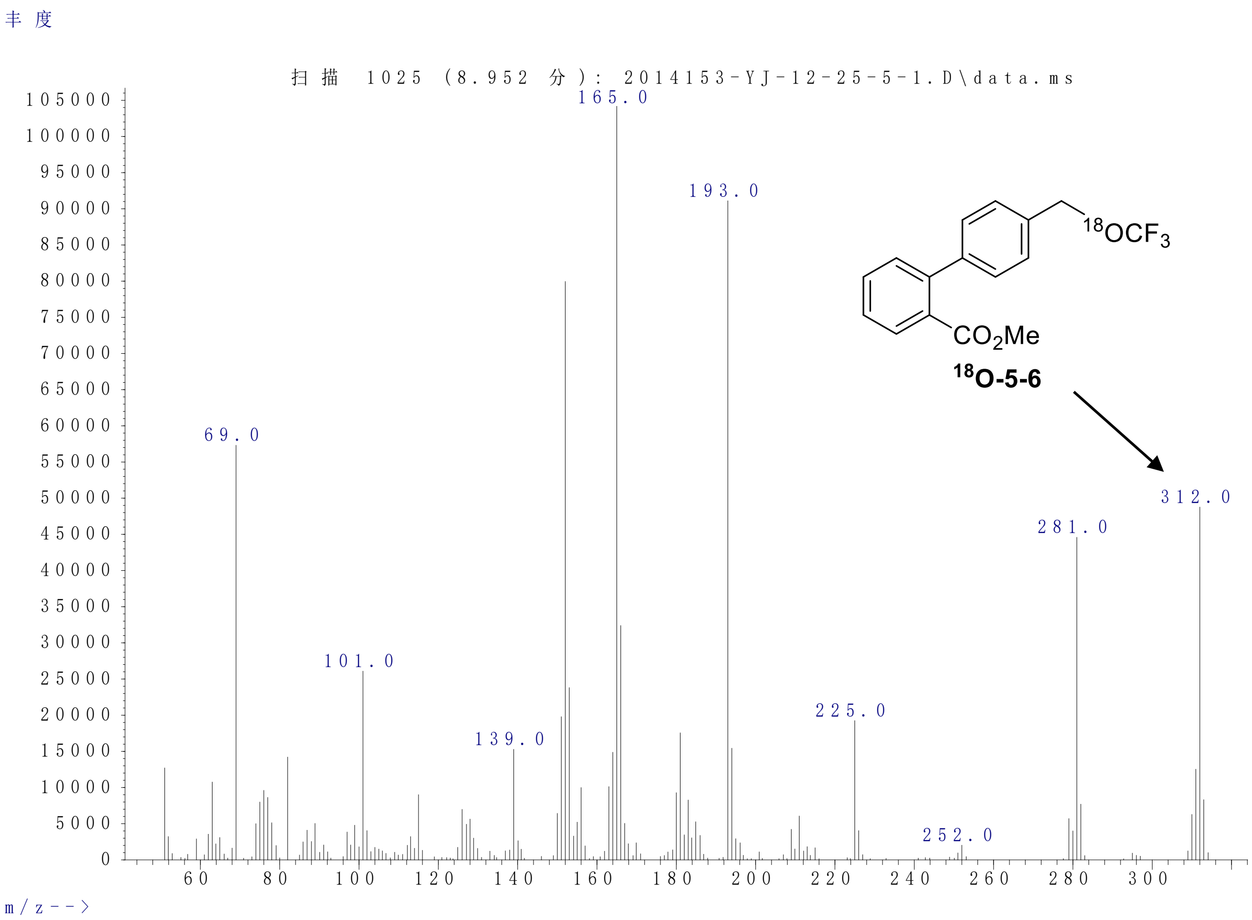

Supplementary Figure 191. EI spectrum of products **^{18}O-5-6**

m/z	Abs. Int.
279.00	5709.0
280.00	4001.0
281.00	44584.0
282.00	7702.0
283.00	597.0
292.80	151.0
295.00	935.0
296.00	631.0
297.00	507.0
309.00	1235.0
310.00	6287.0
311.00	12528.0
312.00	48768.0
313.00	8314.0
314.10	1000.0

$^{18}O : {}^{16}O = 48768.0 : 6287.0 = 89 : 11$

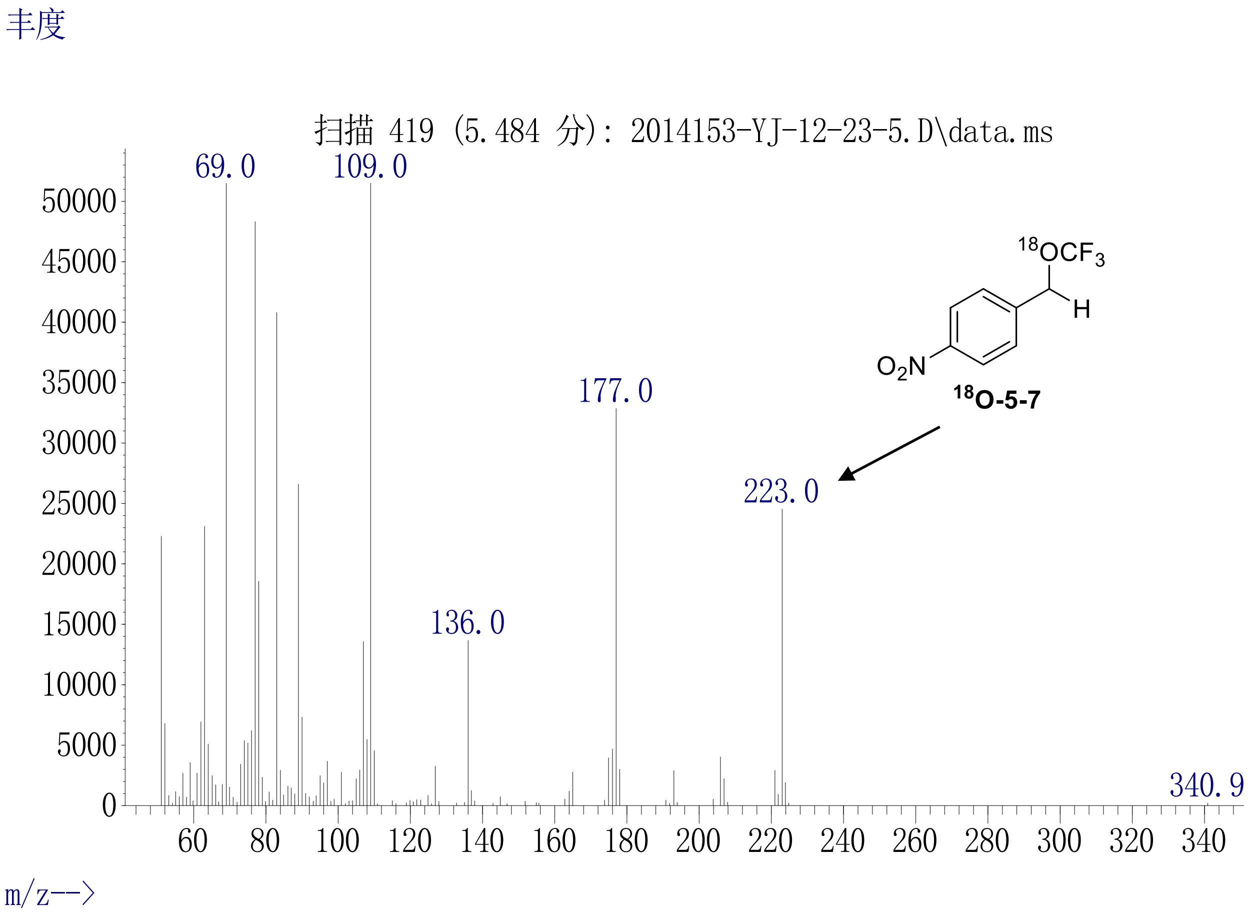

Supplementary Figure 192. EI spectrum of products **^{18}O-5-7**

m/z	Abs. Int.
192.10	271.0
192.90	2367.0
193.90	212.0
204.10	566.0
204.90	312.0
205.90	3965.0
207.00	2276.0
208.00	409.0
221.00	2791.0
222.00	823.0
223.00	23984.0
224.00	2293.0
224.90	196.0
325.10	212.0

$^{18}O : ^{16}O = 23984.0 : 2791.0 = 90 : 10$

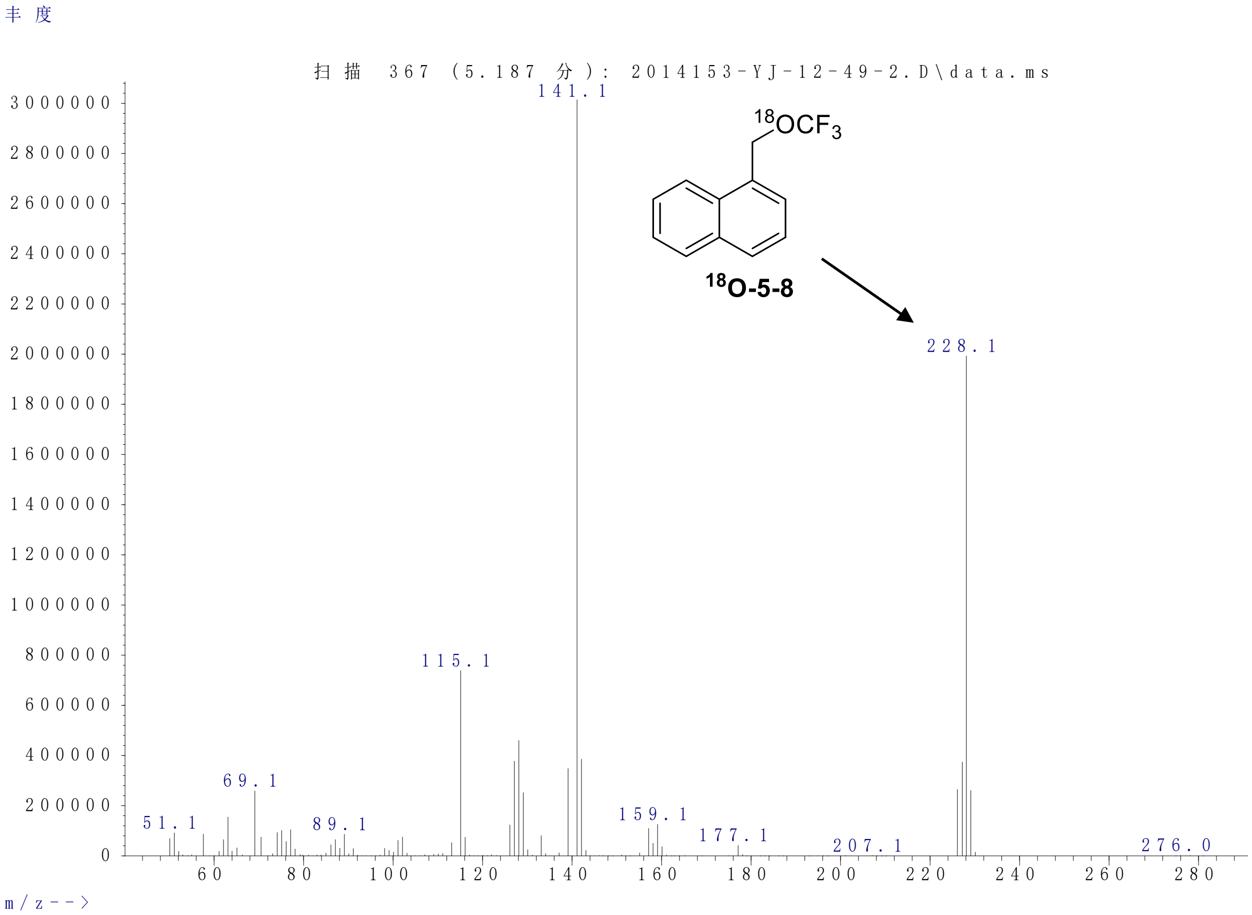

Supplementary Figure 193. EI spectrum of products **^{18}O-5-8**

m/z	Abs. Int.
197.00	157.0
201.10	313.0
207.10	1306.0
208.10	336.0
209.20	151.0
210.20	207.0
226.10	264896.0
227.20	373824.0
228.10	1992192.0
229.10	260544.0
230.10	14811.0
231.10	573.0
274.90	248.0
276.00	1965.0
277.10	632.0
278.10	1372.0
281.00	179.0

$^{18}O : ^{16}O = 1992192.0 : 264896.0 = 88 : 12$

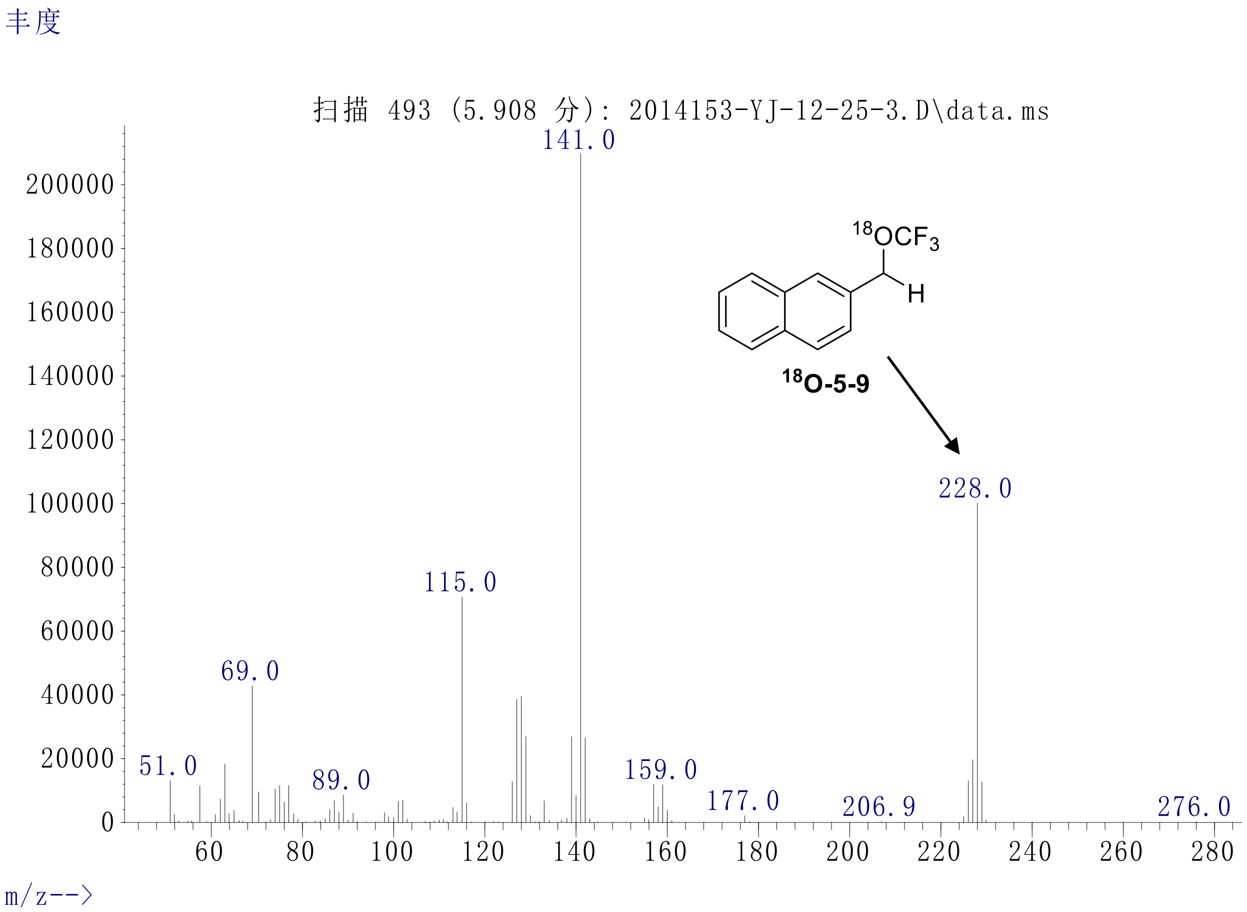

Supplementary Figure 194. EI spectrum of products **^{18}O-5-9**

m/z	Abs. Int.
158.00	6251.0
159.00	13525.0
160.00	4098.0
161.00	413.0
177.00	2670.0
177.80	379.0
206.80	253.0
225.10	2129.0
226.00	14558.0
227.00	22144.0
228.00	118392.0
229.00	15405.0
230.10	743.0

^{18}O : ^{16}O = 118392.0 : 14558.0 = 89 : 11

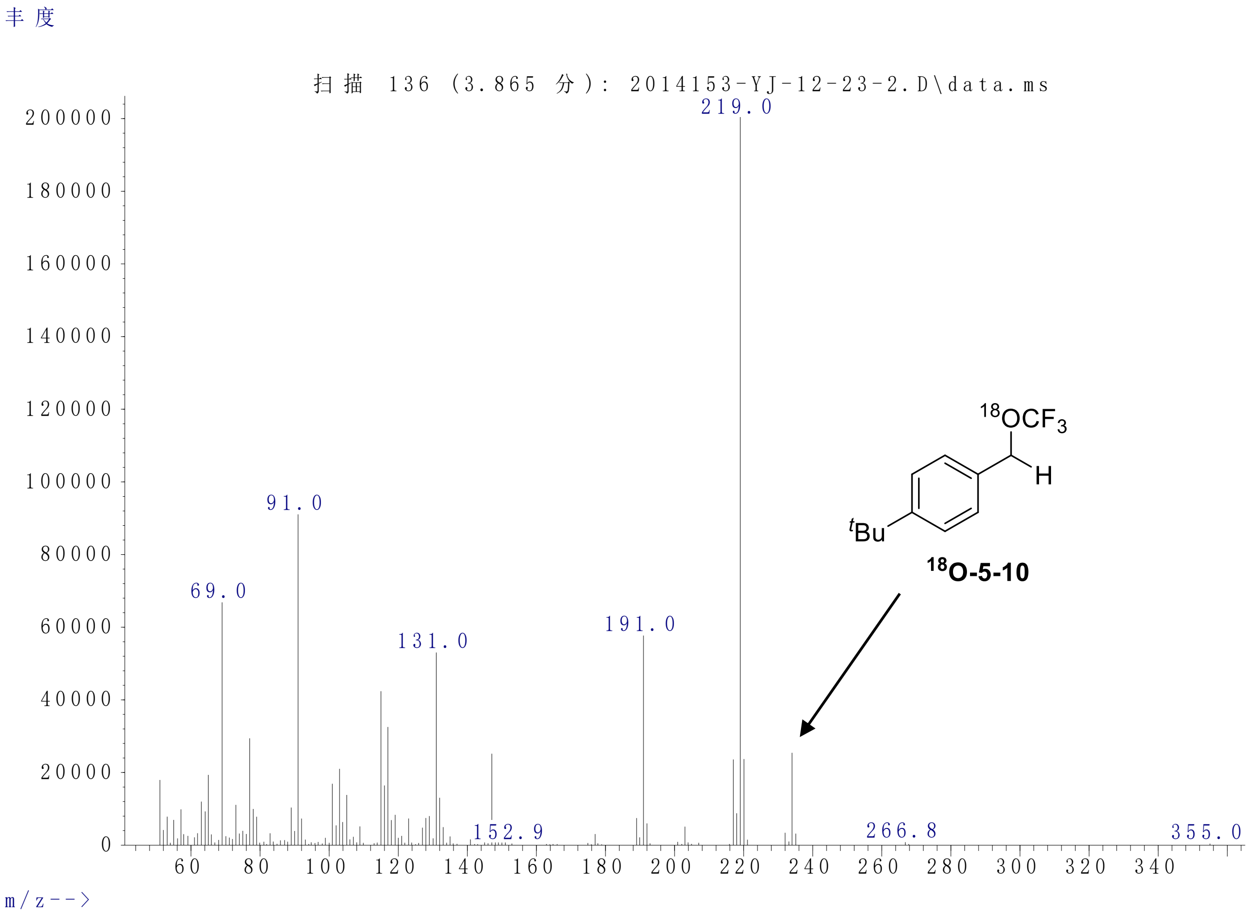

Supplementary Figure 195. EI spectrum of products **^{18}O-5-10**

m/z	Abs. Int.
204.80	205.0
205.20	203.0
206.80	384.0
217.00	24720.0
218.00	8552.0
219.00	189376.0
220.10	23088.0
221.00	963.0
232.00	2784.0
233.10	1001.0
234.00	21544.0
235.00	2883.0
236.00	203.0
266.90	695.0
267.70	197.0
354.90	187.0

^{18}O : ^{16}O = 21544.0 : 2784.0 = 89 : 11

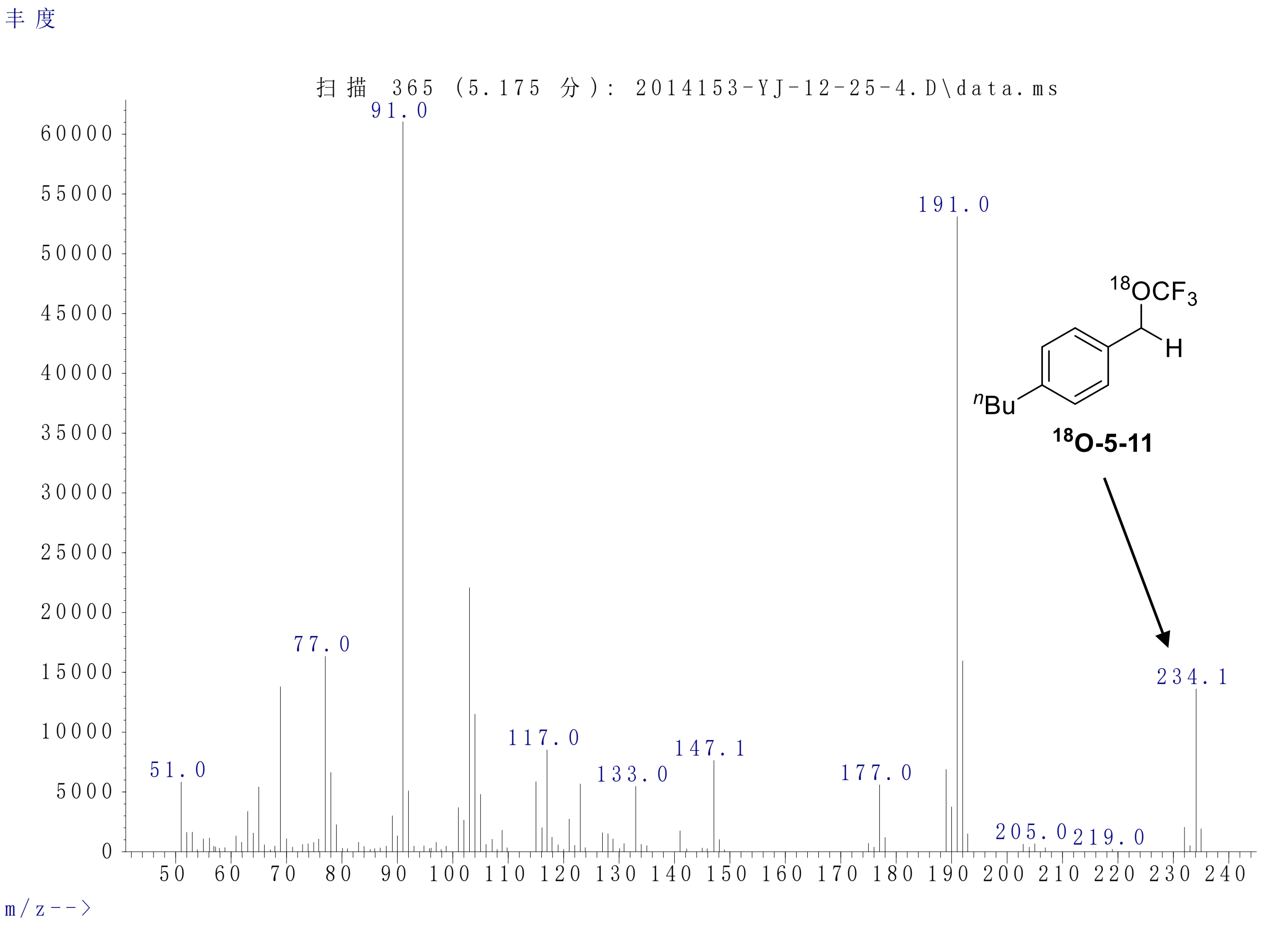

Supplementary Figure 196. EI spectrum of products **^{18}O-5-11**

m/z	Abs. Int.
190.00	3736.0
191.00	56176.0
192.00	15717.0
193.00	1234.0
202.90	495.0
203.90	463.0
205.10	778.0
207.00	268.0
218.80	180.0
219.10	203.0
232.00	2115.0
233.00	701.0
234.10	13952.0
235.00	1983.0

^{18}O : ^{16}O = 13952.0 : 2115.0 = 87 : 13

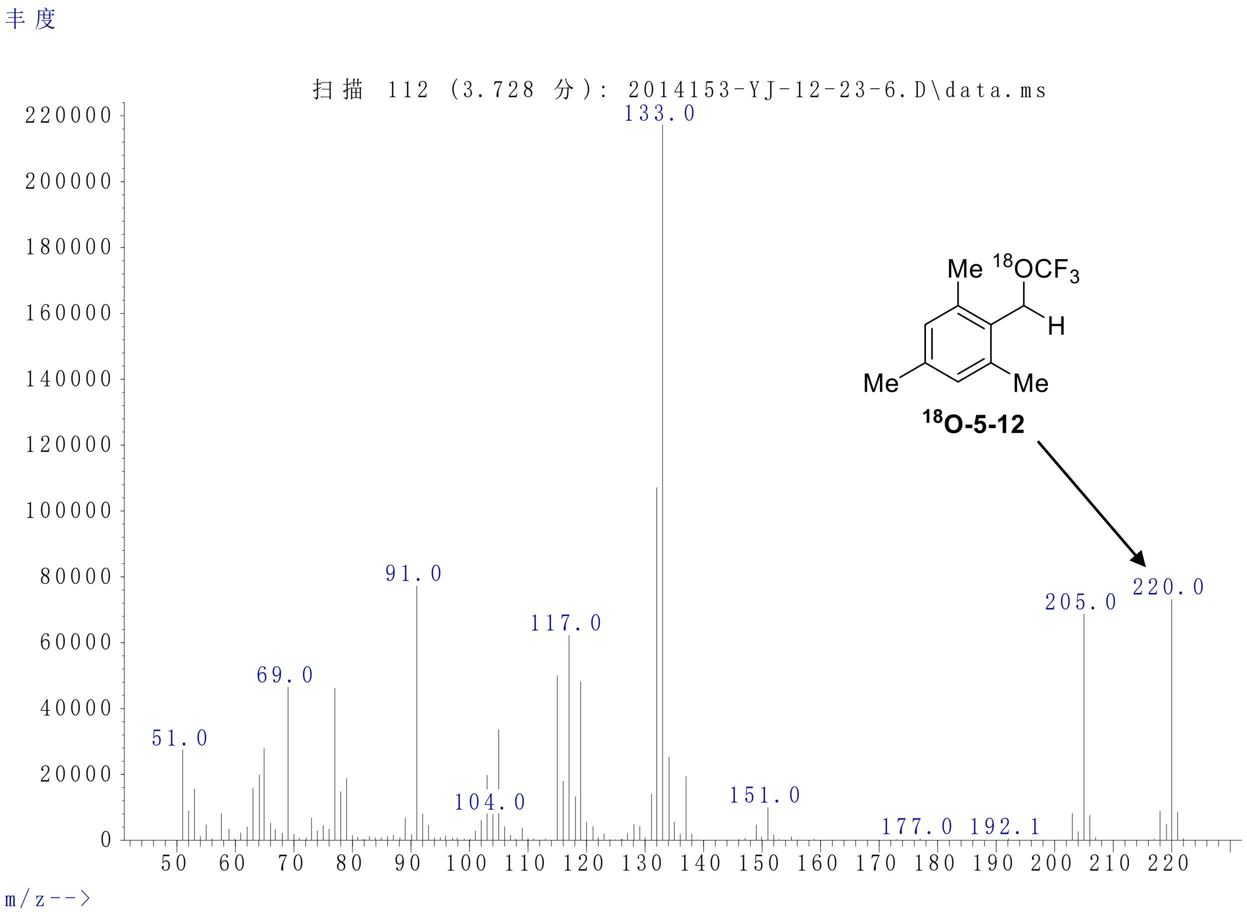

Supplementary Figure 197. EI spectrum of products **^{18}O-5-12**

m/z	Abs. Int.
182.80	161.0
188.90	220.0
190.80	241.0
192.00	504.0
203.00	8558.0
203.90	2196.0
205.00	69656.0
206.00	7513.0
207.10	797.0
217.00	247.0
218.00	9371.0
219.10	5445.0
220.00	74464.0
221.00	8671.0
222.00	400.0

^{18}O : ^{16}O = 74464.0 : 9371.0 = 89 : 11

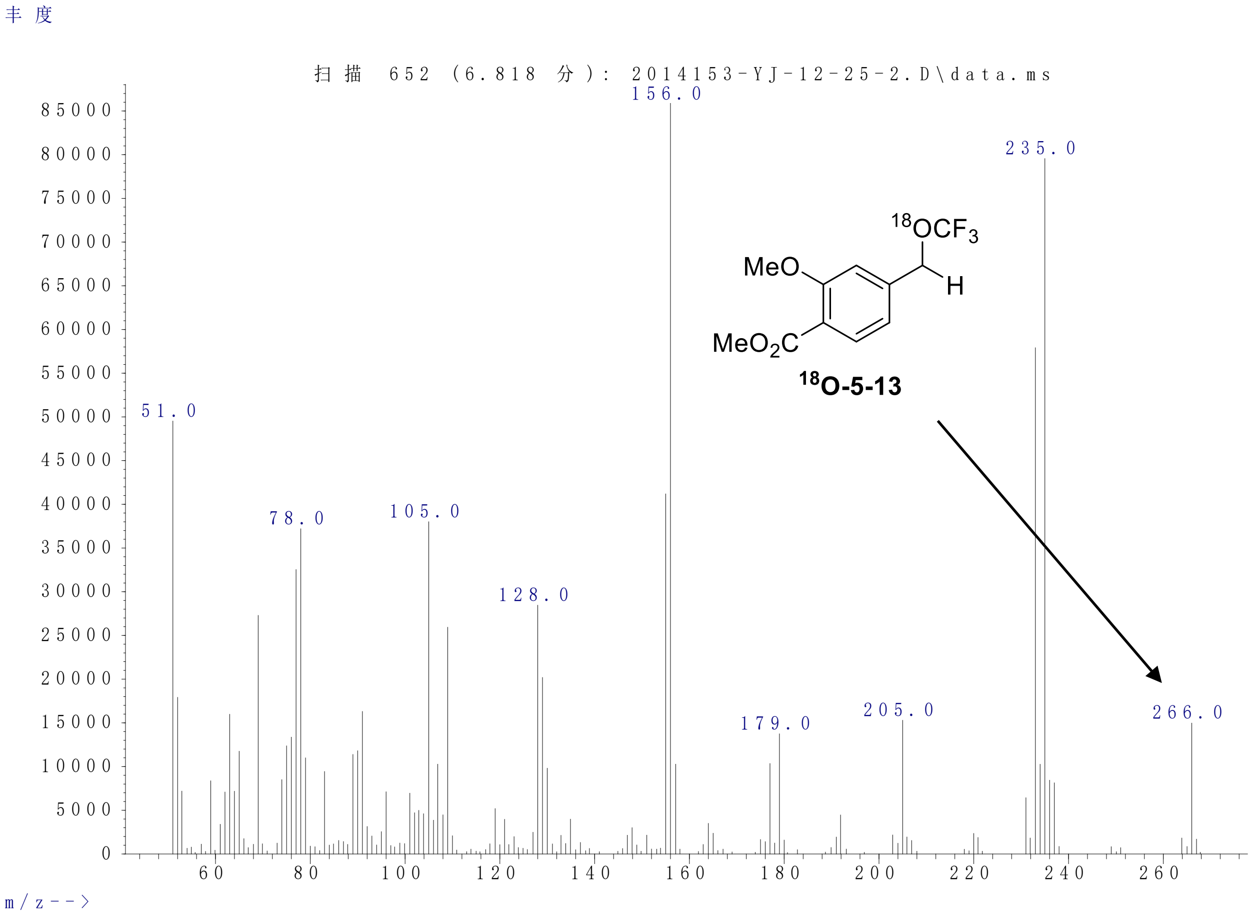

Supplementary Figure 198. EI spectrum of products **^{18}O-5-13**

m/z	Abs. Int.
231.00	7539.0
231.90	2147.0
233.00	68800.0
234.00	12442.0
235.00	95568.0
236.00	10118.0
237.00	9414.0
238.00	1074.0
249.00	996.0
251.00	801.0
264.00	2557.0
265.00	1261.0
266.00	17376.0
267.00	2247.0
267.90	356.0

$^{18}O : ^{16}O$ = 17376.0 : 2557.0 = 87 : 13

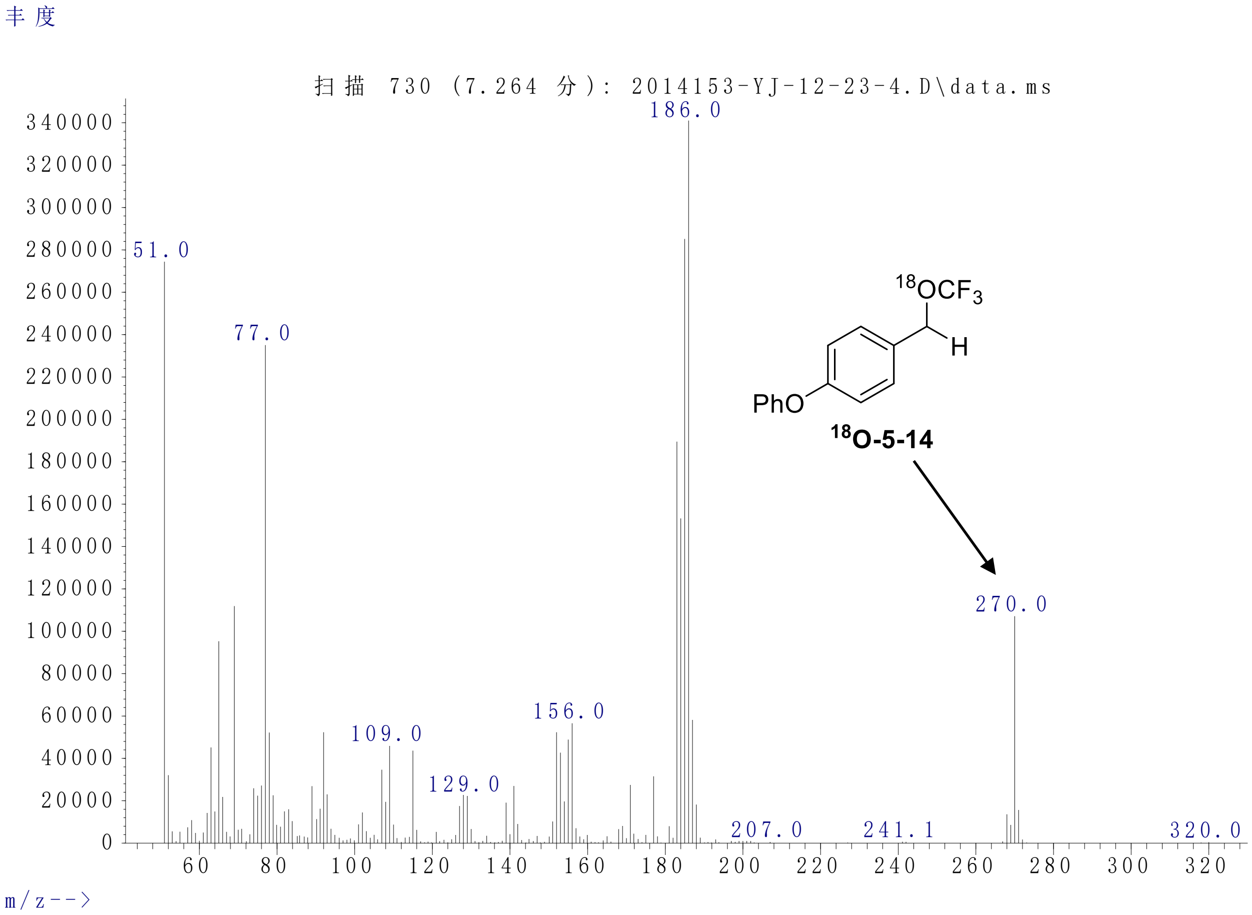

Supplementary Figure 199. EI spectrum of products **^{18}O-5-14**

m/z	Abs. Int.
202.00	1432.0
203.00	318.0
206.90	398.0
227.10	179.0
240.00	161.0
241.00	719.0
242.10	748.0
267.00	699.0
268.00	13611.0
269.00	9589.0
270.00	113576.0
271.00	17992.0
272.00	1449.0
273.10	173.0
319.90	236.0

^{18}O : ^{16}O = 113576.0 : 13611.0 = 89 : 11

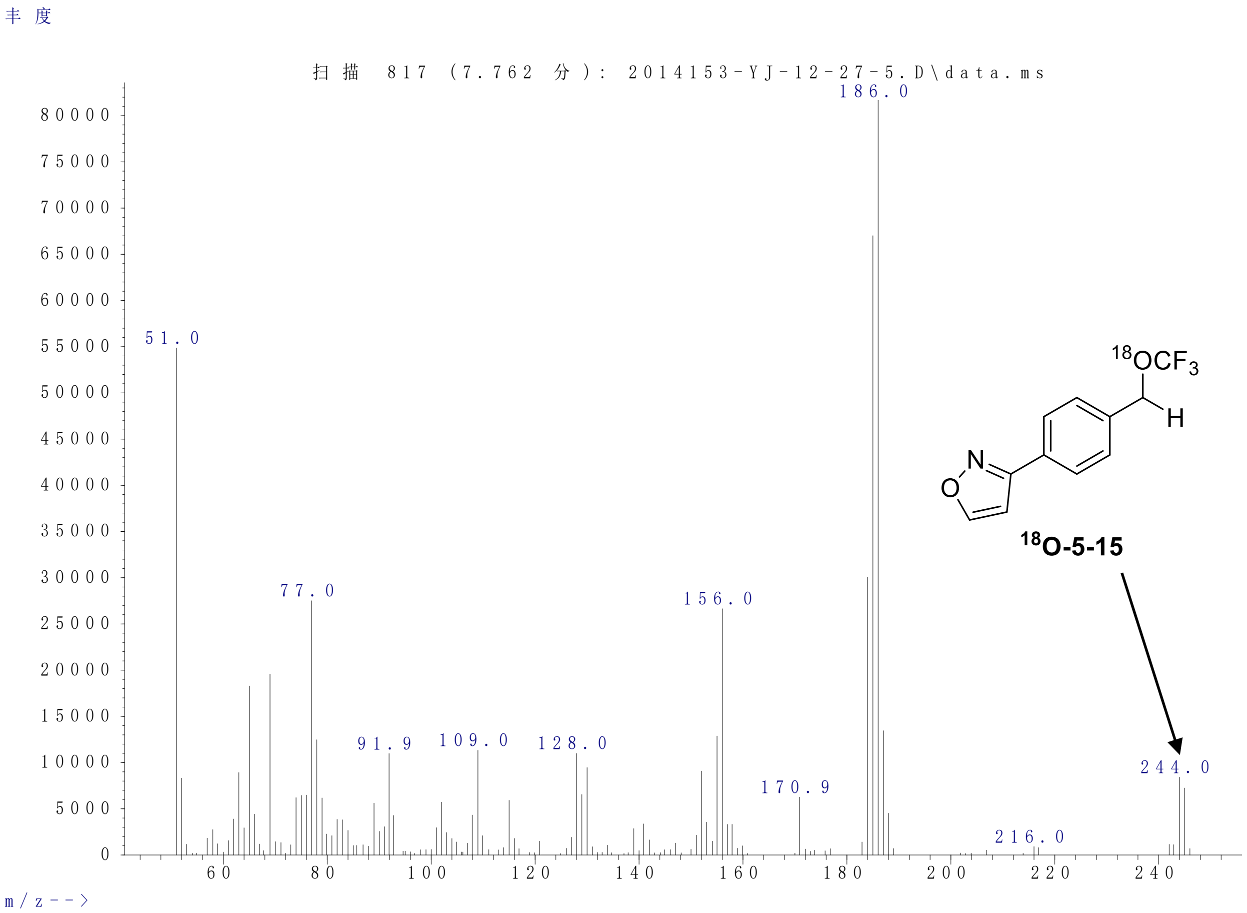

Supplementary Figure 200. EI spectrum of products **^{18}O-5-15**

m/z	Abs. Int.
202.80	157.0
203.90	200.0
206.80	526.0
213.90	183.0
216.00	899.0
216.90	794.0
242.00	1150.0
242.90	1115.0
244.00	8413.0
245.00	7240.0
246.00	689.0

^{18}O : ^{16}O = 7240.0 : 1115.0 = 86 : 14

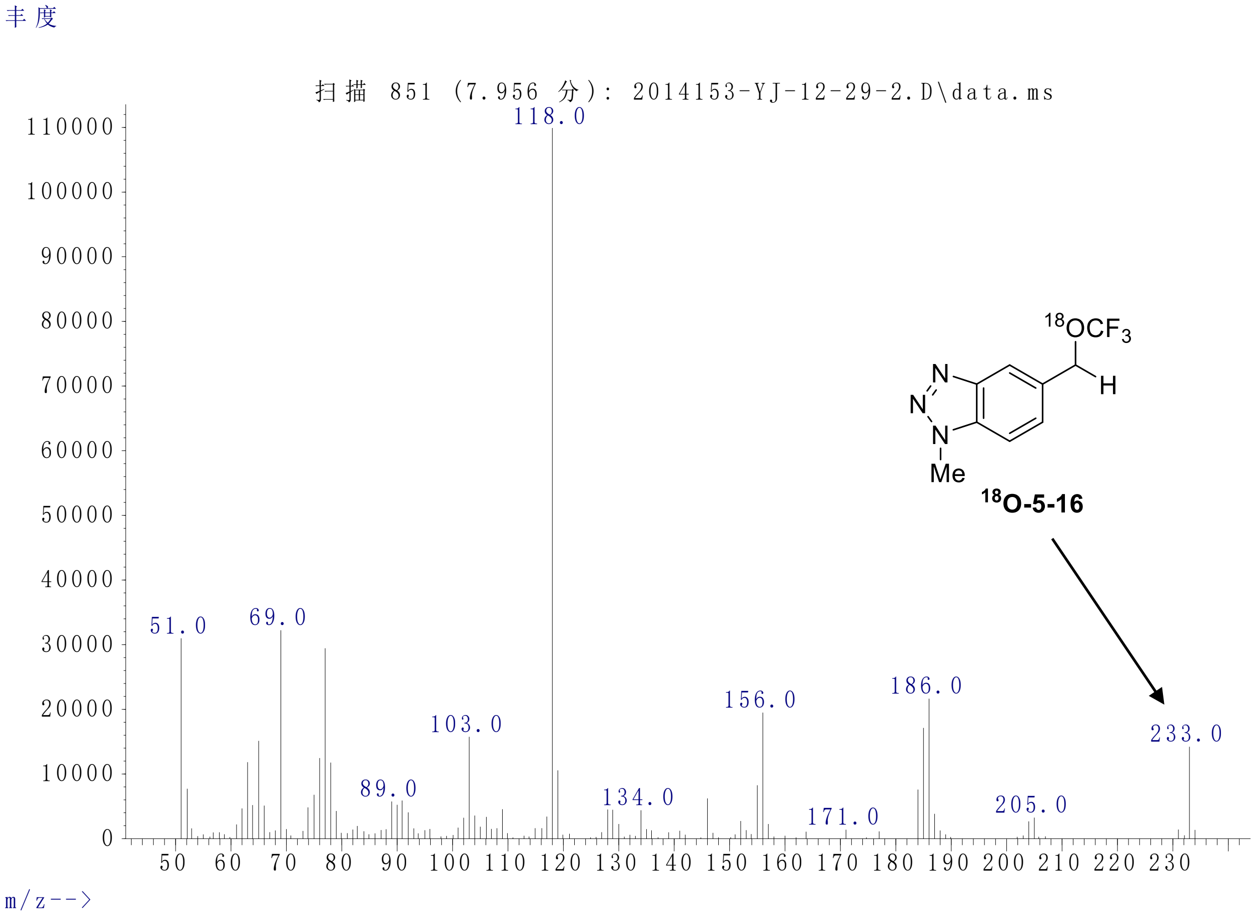

Supplementary Figure 201. EI spectrum of products **^{18}O-5-16**

m/z	Abs. Int.
186.00	29320.0
187.00	4199.0
188.00	1665.0
188.90	782.0
190.00	371.0
201.70	274.0
202.80	546.0
204.00	2445.0
205.00	3843.0
206.00	245.0
207.10	461.0
230.90	1849.0
232.00	502.0
233.00	14839.0
234.10	1469.0

^{18}O : ^{16}O = 14839.0 : 1849.0 = 89 : 11

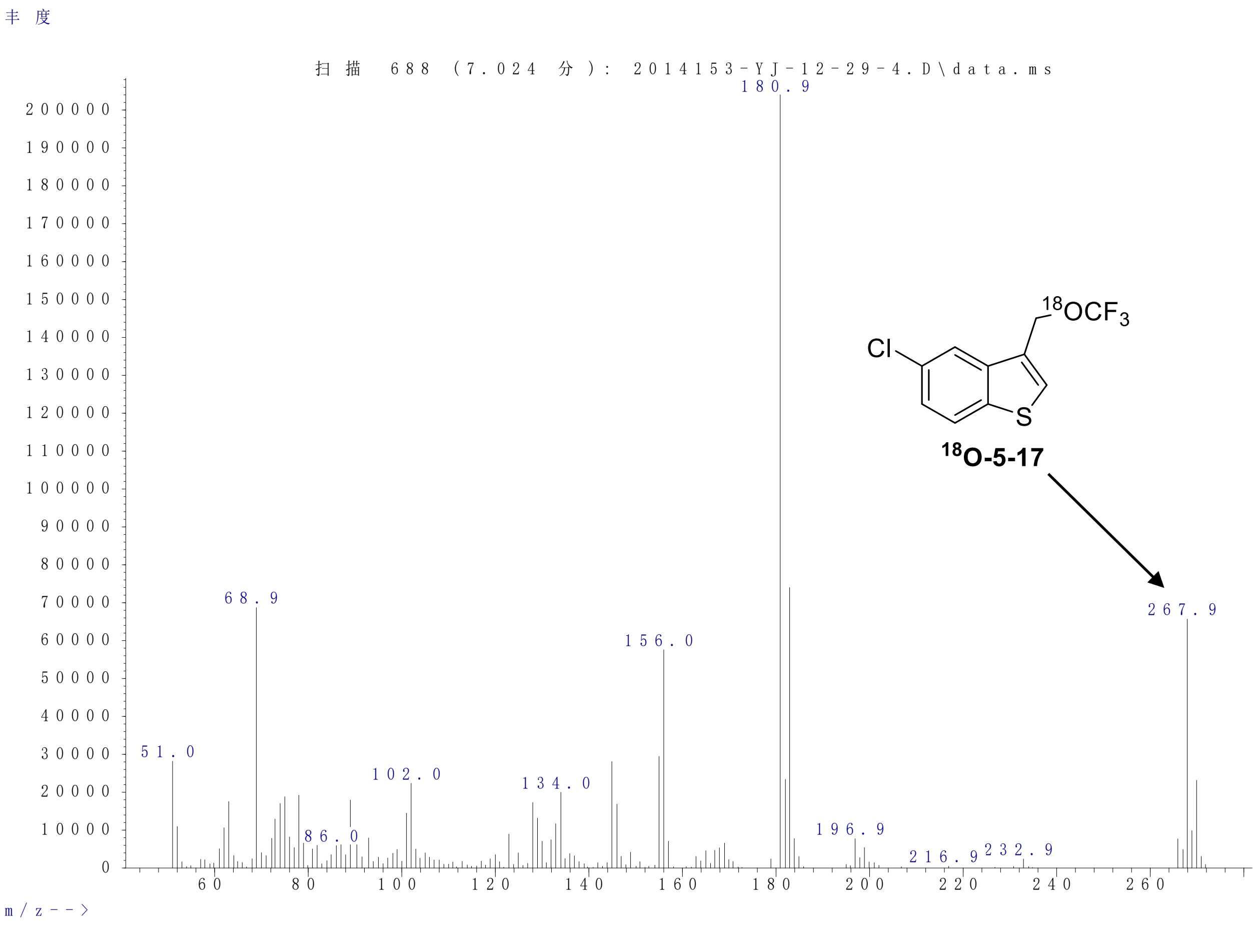

^{18}O-5-17

Supplementary Figure 202. EI spectrum of products **^{18}O-5-17**

m/z	Abs. Int.
216.70	313.0
217.00	271.0
218.90	205.0
226.90	178.0
230.90	272.0
232.90	2030.0
233.80	220.0
234.80	226.0
265.00	489.0
265.90	7934.0
266.90	5223.0
267.90	62296.0
269.00	8177.0
269.90	22368.0
270.90	2564.0
272.00	997.0

$^{18}O : ^{16}O = 62296.0 : 7934.0 = 89 : 11$

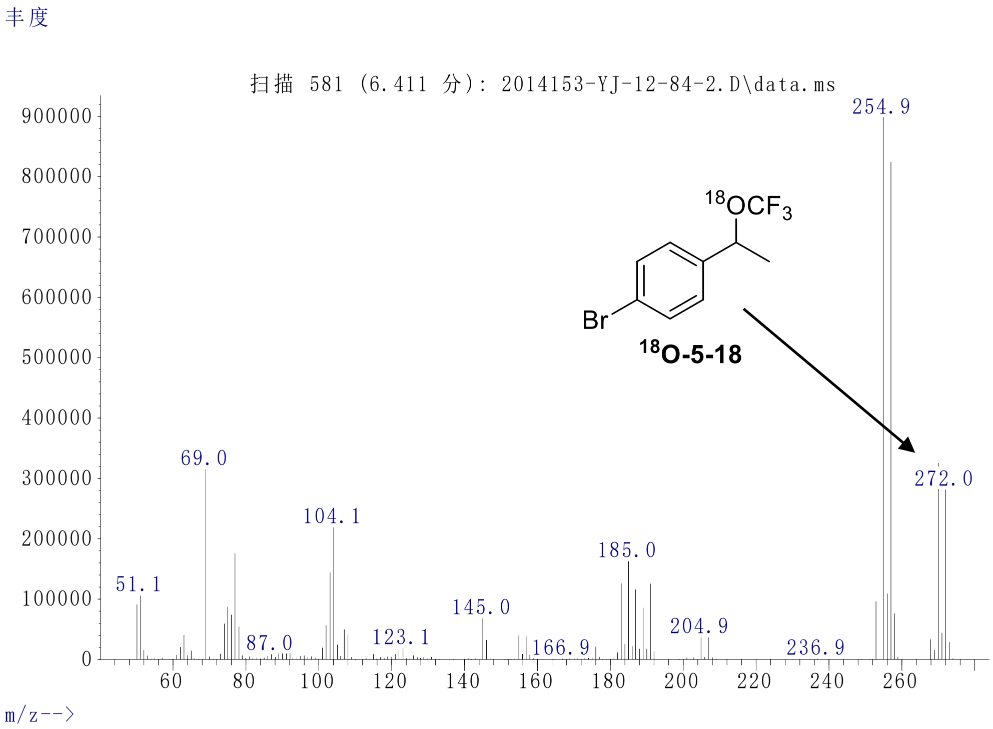

Supplementary Figure 203. EI spectrum of products **^{18}O-5-18**

m/z	丰度
209.10	395.0
234.80	466.0
236.90	667.0
249.00	203.0
251.10	199.0
252.90	96088.0
254.90	934208.0
256.00	108848.0
257.00	823872.0
258.00	75848.0
258.90	3162.0
267.90	32824.0
269.00	15000.0
270.00	324992.0
271.00	43760.0
272.00	280960.0
273.00	28320.0
274.00	1161.0

$^{18}O : ^{16}O$ = 324992.0 : 32824.0 = 91 : 9

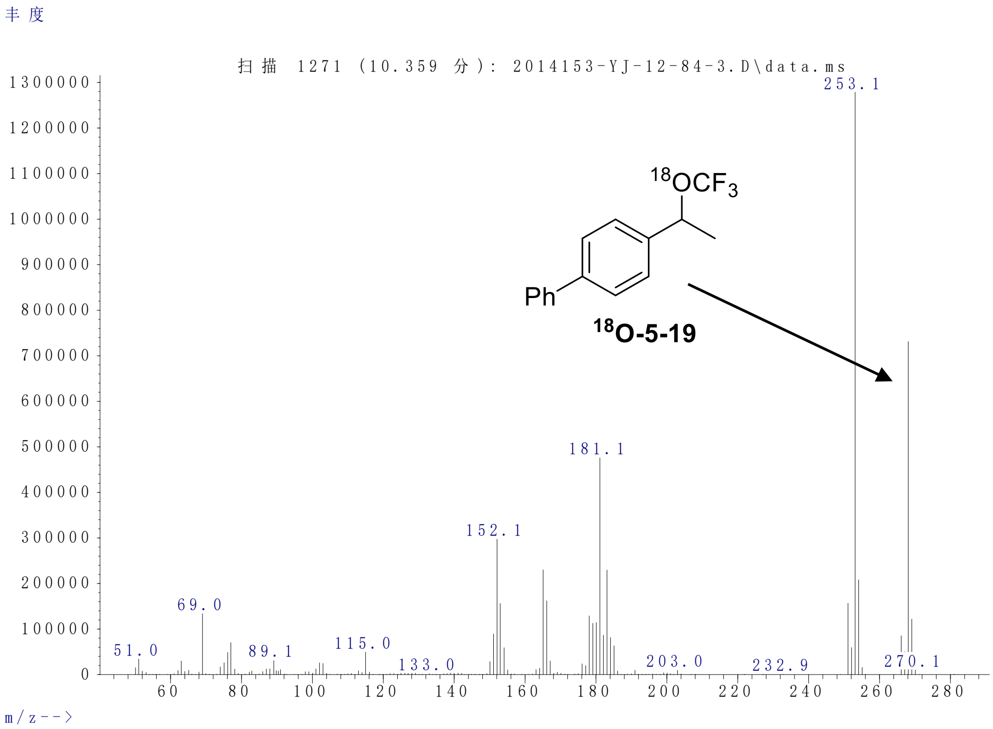

Supplementary Figure 204. EI spectrum of products **^{18}O-5-19**

m/z	丰度
251.10	156480.0
252.10	59168.0
253.10	1315328.0
254.10	207872.0
255.10	15578.0
256.00	566.0
264.10	315.0
266.10	85128.0
267.10	50136.0
268.10	731072.0
269.10	121728.0
270.10	9743.0
271.20	314.0
281.00	203.0

^{18}O : ^{16}O = 731072.0 : 85128.0 = 90 : 10

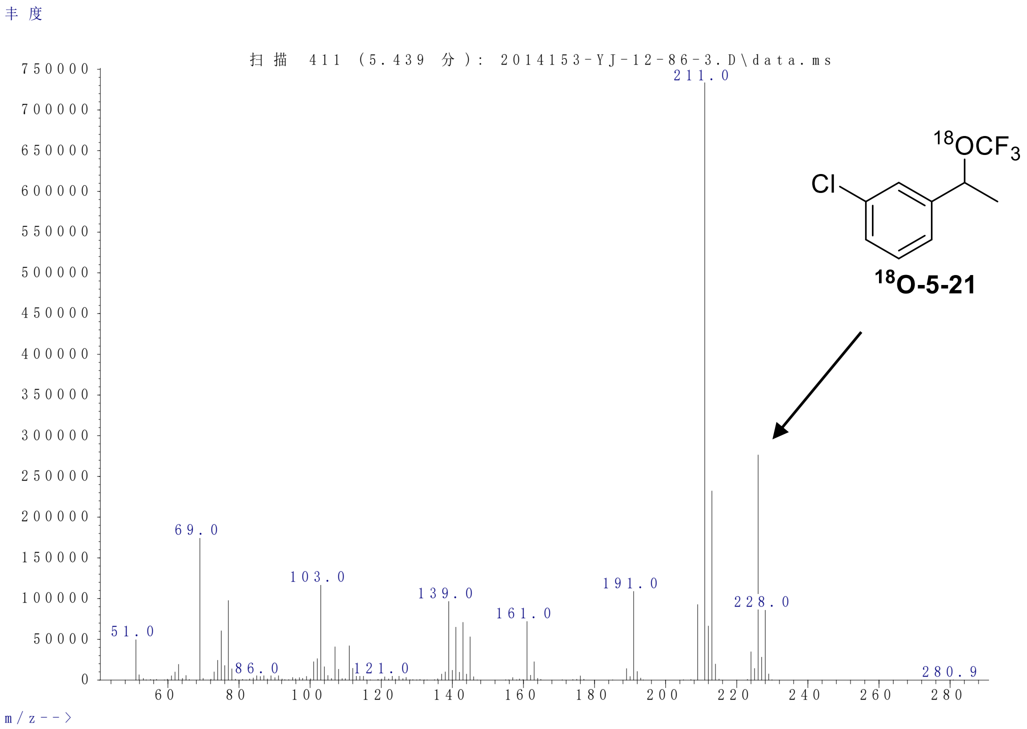

Supplementary Figure 205. EI spectrum of products **^{18}O-5-21**

m/z	丰度
213.00	232128.0
214.00	19592.0
215.00	1105.0
223.00	803.0
224.00	34648.0
225.00	14209.0
226.00	276352.0
227.00	28056.0
228.00	85664.0
229.00	7459.0
229.90	350.0
280.90	173.0

$^{18}O : ^{16}O$ = 276352.0 : 34648.0 = 89 : 11

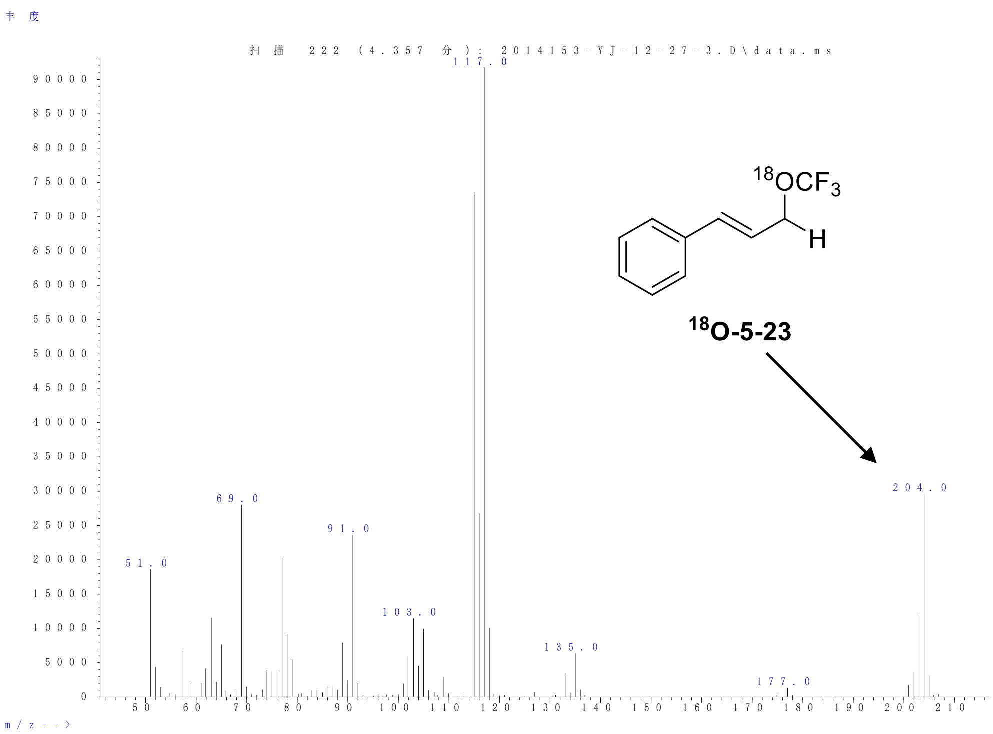

Supplementary Figure 206. EI spectrum of products **^{18}O-5-23**

m/z	丰度
134.00	594.0
135.00	6360.0
136.00	1049.0
136.90	200.0
174.90	244.0
177.00	1336.0
178.00	233.0
200.90	1724.0
202.00	3652.0
203.00	12121.0
204.00	29600.0
205.00	3072.0
205.90	238.0
206.90	380.0

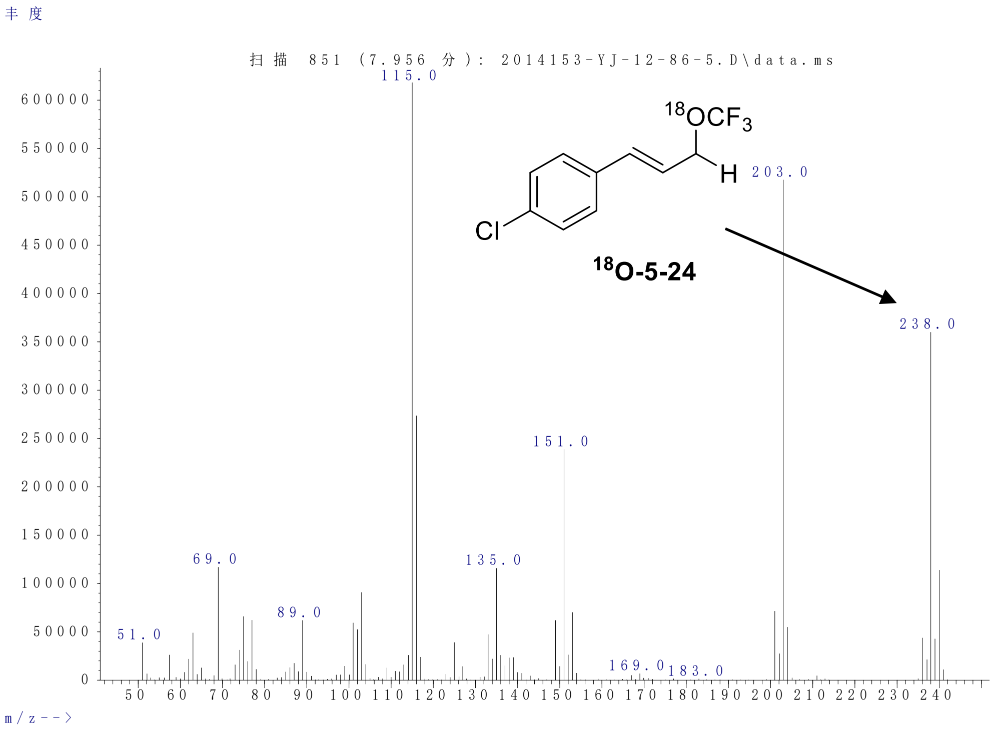

Supplementary Figure 207. EI spectrum of products **^{18}O-5-24**

m/z	丰度
204.00	54688.0
205.10	2239.0
206.90	449.0
209.00	592.0
211.00	4419.0
211.90	288.0
212.90	1254.0
213.80	171.0
235.00	1448.0
236.00	43528.0
237.10	21152.0
238.00	359744.0
239.00	42600.0
240.00	113680.0
241.00	10808.0
241.90	493.0

^{18}O : ^{16}O = 359744.0 : 43528.0 = 89 : 11

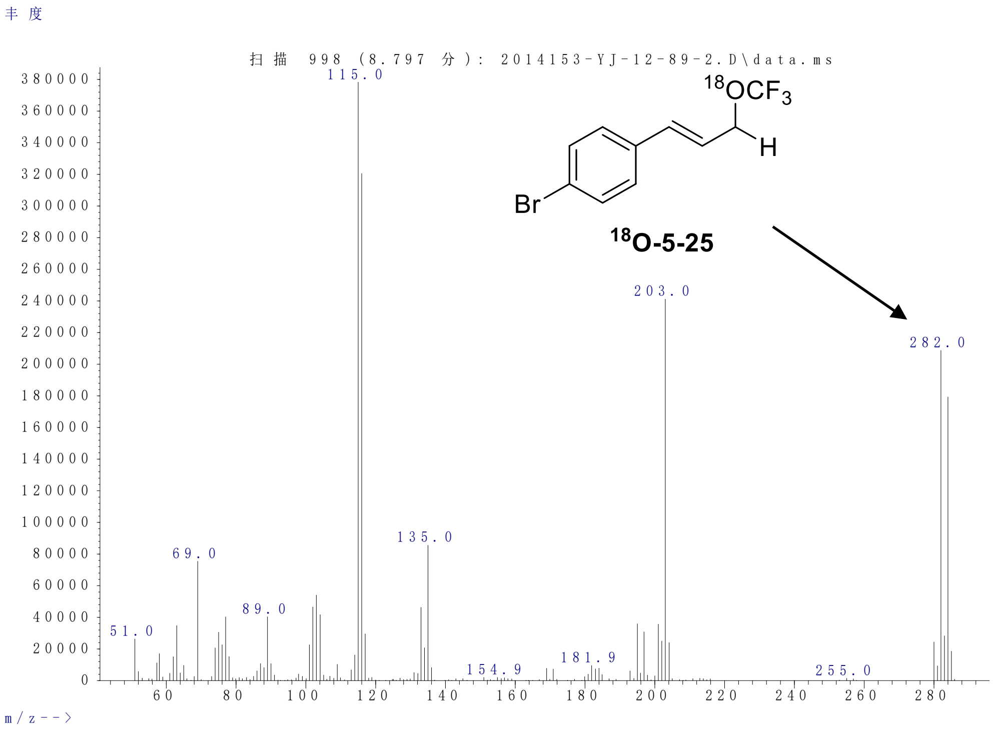

Supplementary Figure 208. EI spectrum of products **^{18}O-5-25**

m/z	丰度
208.90	399.0
210.90	1216.0
212.90	1457.0
213.90	1201.0
214.90	543.0
215.90	1107.0
252.90	291.0
255.00	1388.0
256.90	1110.0
257.80	174.0
280.00	24368.0
281.00	9284.0
282.00	208704.0
283.00	28272.0
284.00	179264.0
285.00	18536.0
285.90	852.0

^{18}O : ^{16}O = 208704.0 : 24368.0 = 90 : 10

丰度

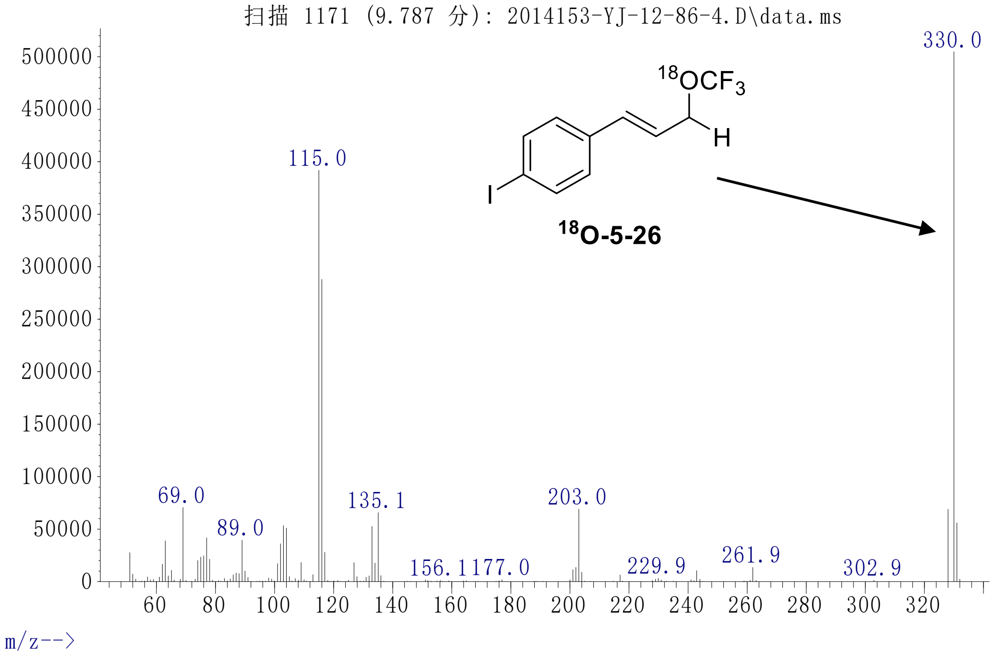

Supplementary Figure 209. EI spectrum of products **^{18}O-5-26**

m/z	丰度
244.00	2338.0
244.90	263.0
258.70	453.0
259.10	399.0
259.80	160.0
261.00	1241.0
261.90	13555.0
263.00	1130.0
302.90	1317.0
328.00	68832.0
330.00	526848.0
331.00	55888.0
332.00	2315.0

^{18}O : ^{16}O = 526848.0 : 68832.0 = 88 : 12

丰度

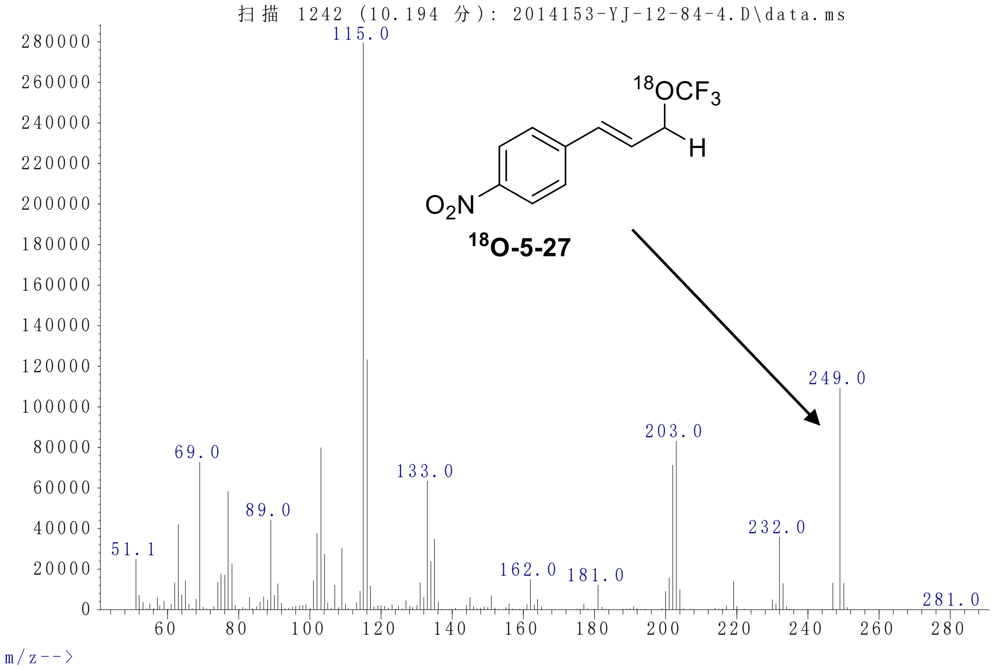

Supplementary Figure 210. EI spectrum of products **^{18}O-5-27**

m/z	丰度
222.10	339.0
229.10	193.0
230.00	4932.0
231.00	2934.0
232.00	35896.0
233.00	12989.0
234.00	1557.0
234.90	259.0
235.30	182.0
245.20	241.0
247.00	13231.0
249.00	109240.0
250.10	13064.0
251.00	1159.0
281.00	176.0

^{18}O : ^{16}O = 109240.0 : 13231.0 = 89 : 11

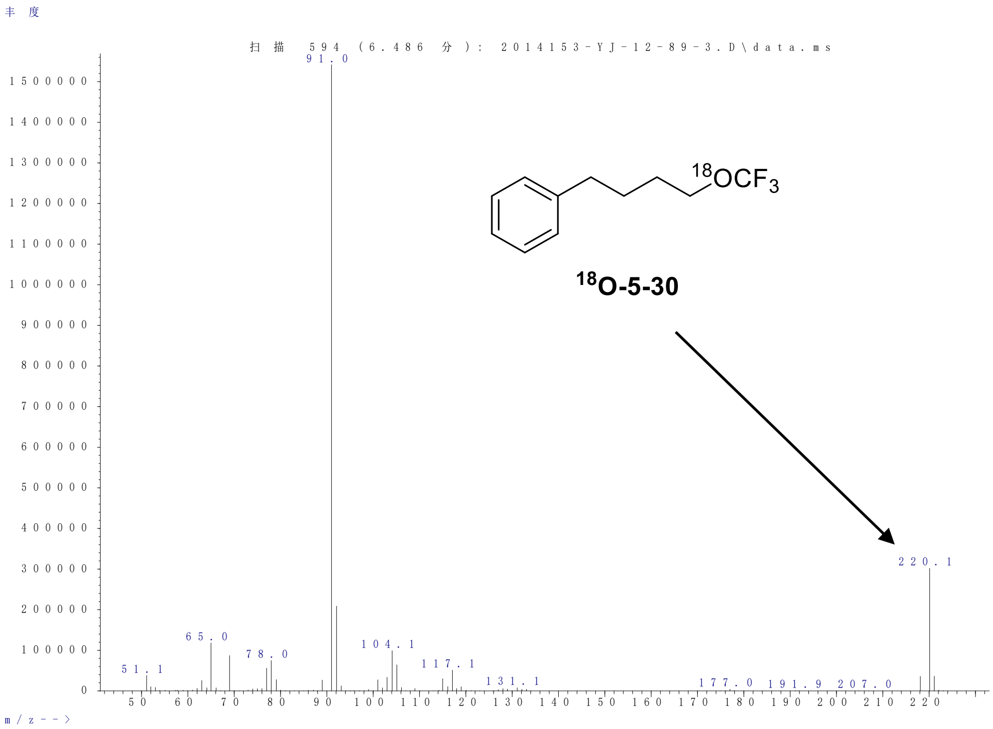

Supplementary Figure 211. EI spectrum of products **^{18}O-5-30**

m/z	丰度
174.90	432.0
176.00	189.0
177.00	4030.0
177.90	606.0
191.10	205.0
191.90	377.0
204.80	354.0
207.00	363.0
218.10	35608.0
220.10	301632.0
221.10	35976.0
222.10	1942.0
223.00	201.0

^{18}O : ^{16}O = 301632.0 : 35608.0 = 89 : 11

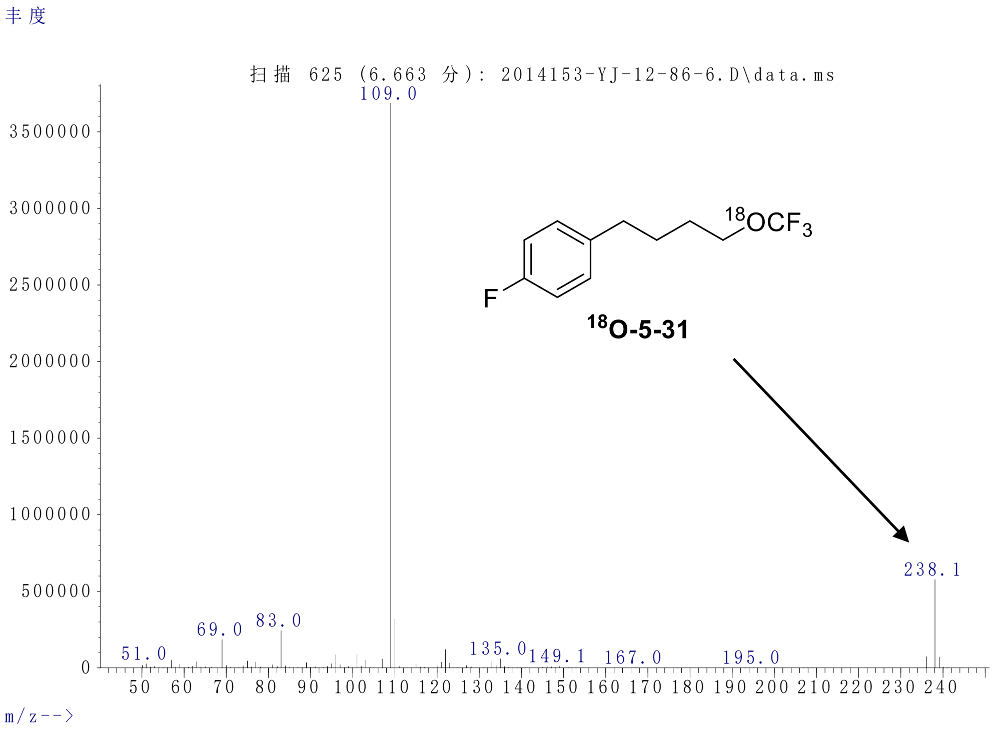

Supplementary Figure 212. EI spectrum of products **^{18}O-5-31**

m/z	丰度
149.10	9851.0
150.10	2734.0
151.10	2942.0
151.90	296.0
152.90	177.0
167.00	177.0
177.00	162.0
193.00	212.0
195.00	2148.0
196.00	185.0
206.90	371.0
207.90	350.0
208.90	165.0
236.10	72248.0
238.10	575680.0
239.10	70408.0
240.10	3758.0
241.00	180.0

$^{18}O : ^{16}O = 575680.0 : 72248.0 = 89 : 11$

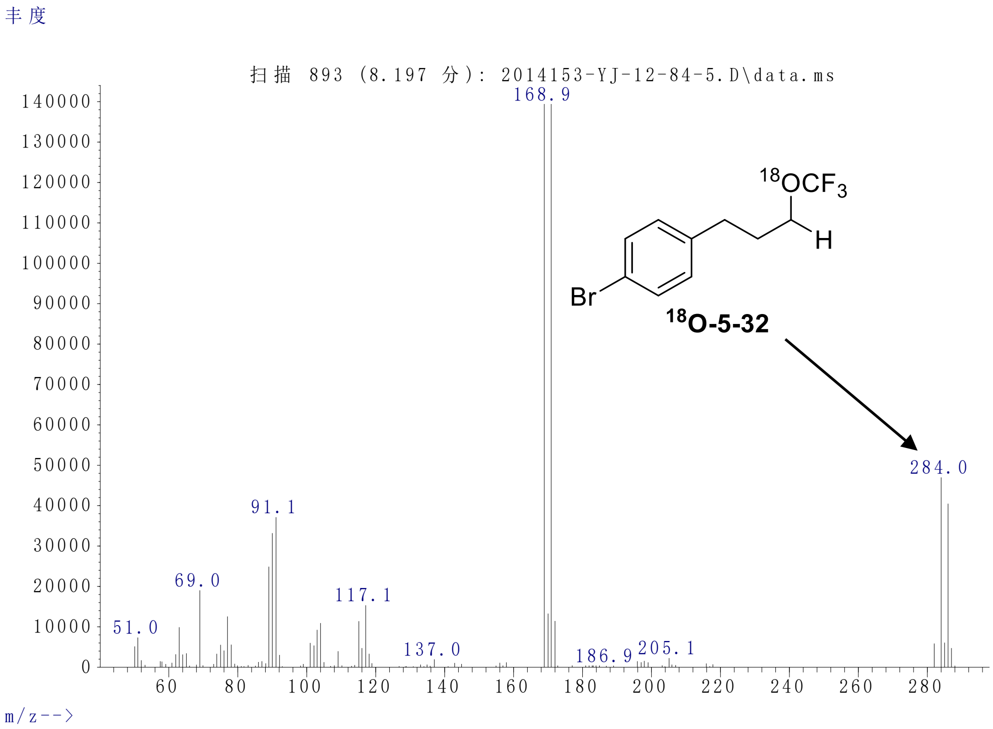

Supplementary Figure 213. EI spectrum of products **^{18}O-5-32**

m/z	丰度
203.10	400.0
203.90	154.0
205.10	2192.0
205.90	602.0
207.00	474.0
215.90	868.0
216.90	181.0
217.80	656.0
282.00	5809.0
284.00	46944.0
285.00	6041.0
286.00	40440.0
287.00	4677.0
288.00	321.0

^{18}O : ^{16}O = 46944.0 : 5809.0 = 89 : 11

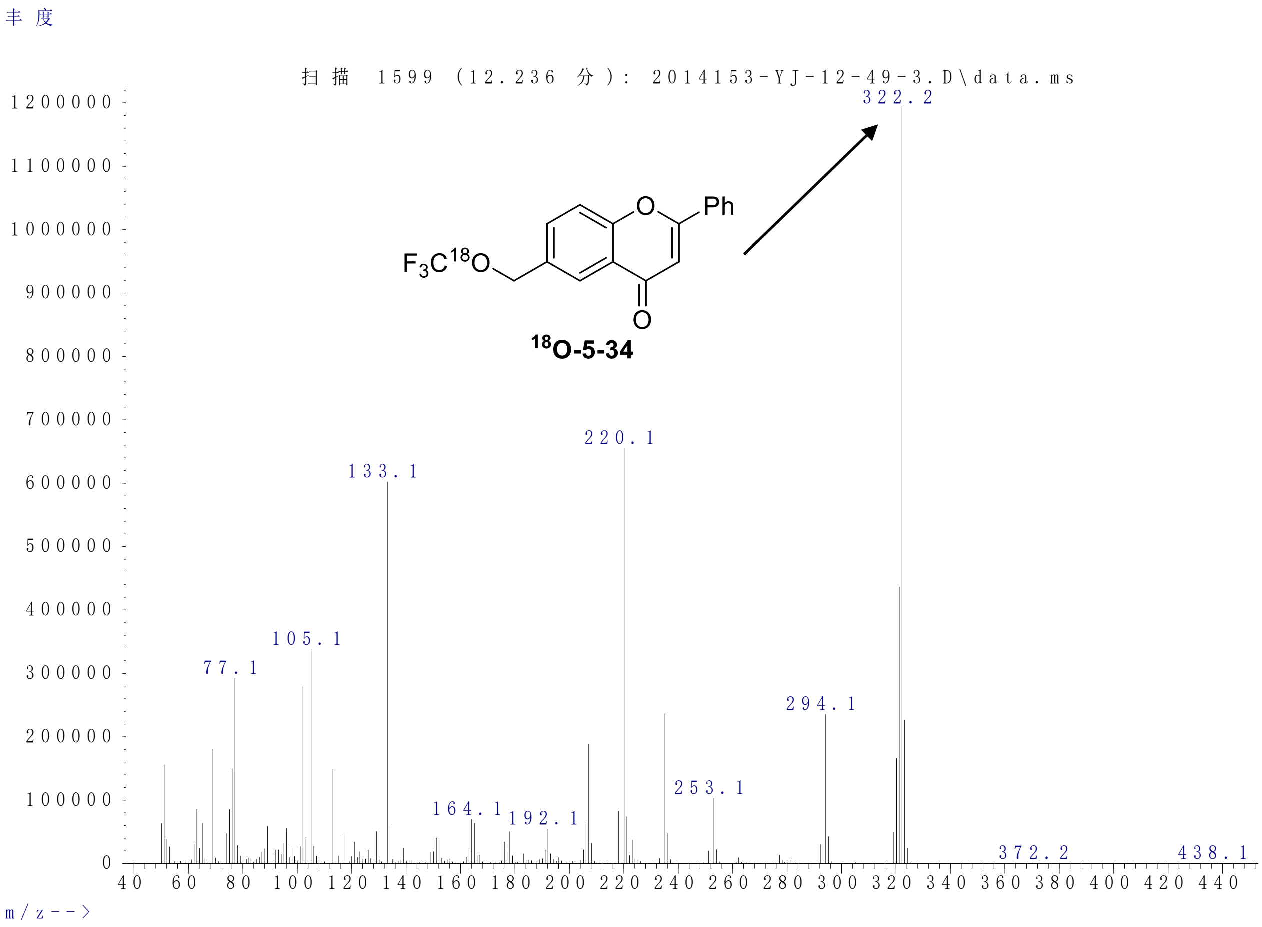

Supplementary Figure 214. EI spectrum of products **^{18}O-5-34**

m/z	Abs. Int.
305.20	1471.0
306.10	333.0
319.20	49008.0
320.20	165696.0
321.20	436032.0
322.20	1223680.0
323.20	225856.0
324.20	23832.0
325.20	2065.0
334.30	223.0
335.30	627.0
336.20	679.0
338.00	243.0
355.20	166.0
370.20	335.0
371.00	190.0

^{18}O : ^{16}O = 1223680.0 = 165696.0 = 88 : 12

Supplementary References

1. Roy SS, Chakraborty P, Bhattacharya S. Intervention in cyclophosphamide induced oxidative stress and DNA damage by a flavonyl-thiazolidinedione based organoselenocyanate and evaluation of its efficacy during adjuvant therapy in tumor bearing mice. *Eur J Med Chem* **73**, 195-209 (2014).
2. Brambilla M, Tredwell M. Palladium-Catalyzed Suzuki-Miyaura Cross-Coupling of Secondary alpha-(Trifluoromethyl)benzyl Tosylates. *Angew Chem Int Ed* **56**, 11981-11985 (2017).
3. Tanaka K, Hosokawa A, Yoshida K. A practical synthesis of indanofan via one-pot bromination of 3-chloroethylbenzene. *Synthesis*, 249-253 (1999).
4. Cho H, Shin JE, Lee S, Jeon H, Park S, Kim S. Asymmetric C alpha-Alkylation of Proline via Chirality Transfers of Conformationally Restricted Proline Derivative: Application to the Total Synthesis of (-)-Amathaspiramide F. *Org Lett* **20**, 6121-6125 (2018).
5. Kurauchi D, Hirano K, Kato H, Saito T, Miyamoto K, Uchiyama M. Dialkylzinc-mediated allylic polyfluoroarylation reaction. *Tetrahedron* **71**, 5849-5857 (2015).
6. West TH, Daniels DS, Slawin AM, Smith AD. An isothiourea-catalyzed asymmetric [2,3]-rearrangement of allylic ammonium ylides. *J Am Chem Soc* **136**, 4476-4479 (2014).
7. Smith SM, Takacs JM. Amide-Directed Catalytic Asymmetric Hydroboration of Trisubstituted Alkenes. *J Am Chem Soc* **132**, 1740-+ (2010).
8. Thornton AR, Martin VI, Blakey SB. π-Nucleophile Traps for Metallonitrene/Alkyne Cascade Reactions: A Versatile Process for the Synthesis of α-Aminocyclopropanes and β-Aminostyrenes. *J Am Chem Soc* **131**, 2434-2435 (2009).
9. J. X, D. Z, Tang P. Direct Dehydroxytrifluoromethoxylation of Alcohols. *Angew Chem Int Ed* **57**, 292-295 (2018).
10. Yang H, Wang F, Jiang X, Zhou Y, Xu X, Tang P. Silver-Promoted Oxidative Benzylic C-H Trifluoromethoxylation. *Angew Chem Int Ed* **57**, 13266-13270 (2018).
11. Zhang W, Chen J, Lin JH, Xiao JC, Gu YC. Rapid Dehydroxytrifluoromethoxylation of Alcohols. *iScience* **5**, 110-117 (2018).
12. Kamijo S, Tao K, Takao G, Tonoda H, Murafuji T. Photoinduced Oxidation of Secondary Alcohols Using 4-Benzoylpyridine as an Oxidant. *Org Lett* **17**, 3326-3329 (2015).
13. Qi X, Chen P, Liu G. Catalytic Oxidative Trifluoromethoxylation of Allylic C-H Bonds Using a Palladium Catalyst. *Angew Chem Int Ed* **56**, 9517-9521 (2017).
14. Oian JH, Suo JS. $KHSO_4$ catalysed Pechmann condensation under solvent-free conditions. *J Chem Res*, 486-487 (2005).
15. Kaufman KD, *et al.* Reactions of furocoumarins. II. Synthetic aminomethyl psoralens via chloromethylation or benzylic bromination. *J Heterocycl Chem* **19**, 1051-1056 (1982).
16. Fascione MA, Adshead SJ, Mandal PK, Kilner CA, Leach AG, Turnbull WB. Mechanistic studies on a sulfoxide transfer reaction mediated by diphenyl sulfoxide/triflic anhydride. *Chem Eur J* **18**, 2987-2997 (2012).
17. Frisch MJ, Trucks GW, Schlegel HB, Scuseria GE, Robb MA, Cheeseman JR, Scalmani G, Barone V, Mennucci B, Petersson GA, Nakatsuji H, Caricato M, Li X, Hratchian HP, Izmaylov AF, Bloino J, Zheng G, Sonnenberg JL, Hada M, Ehara M, Toyota K, Fukuda R, Hasegawa J, Ishida M, Nakajima T, Honda Y, Kitao O, Nakai H, Vreven T, Montgomery JA, Jr, Peralta J, Ogliaro EF, Bearpark M, Heyd JJ, Brothers E, Kudin KN, Staroverov VN, Keith T, Kobayashi R, Normand J, Raghavachari K, Rendell A, Burant JC, Iyengar SS, Tomasi J, Cossi M, Rega N, Millam JM, Klene M, Knox JE, Cross JB, Bakken V, Adamo C, Jaramillo J, Gomperts R, Stratmann RE, Yazyev O, Austin AJ, Cammi R, Pomelli C, Ochterski JW, Martin RL, Morokuma K, Zakrzewski VG, Voth GA, Salvador P, Dannenberg JJ, Dapprich S, Daniels AD, Farkas O, Foresman JB, Ortiz JV, Cioslowski J, and Fox DJ. Gaussian 09,

Revision D.01, Gaussian, Inc., Wallingford CT, (2013).
