# Supplementary material for: Oxidation of difluorocarbene and subsequent trifluoromethoxylation
Source: Nat Commun. 2019 Nov 25;10:5362. doi: 10.1038/s41467-019-13359-z (PMC6877537; doi:10.1038/s41467-019-13359-z)
Supplement: Supplementary file 3 — Description of Additional Supplementary Files [file 41467_2019_13359_MOESM3_ESM.pdf]

Description of Additional Supplementary Files

File Name: Supplementary Data 1
Description: Relative free energies for the generation of difluorocarbene

File Name: Supplementary Data 2
Description: Relative free energies for trifluoromethoxylation
