# Supplementary material for: Oxidation of difluorocarbene and subsequent trifluoromethoxylation
Source: Nat Commun. 2019 Nov 25;10:5362. doi: 10.1038/s41467-019-13359-z (PMC6877537; doi:10.1038/s41467-019-13359-z)
Supplement: Supplementary file 4 — Supplementary Data 1 [file 41467_2019_13359_MOESM4_ESM.pdf]

DFT calculations: optimized geometrical coordinates and calculated total energies. All energies are presented with atomic unit.

Relative free energies for the generation of difluorocarbene

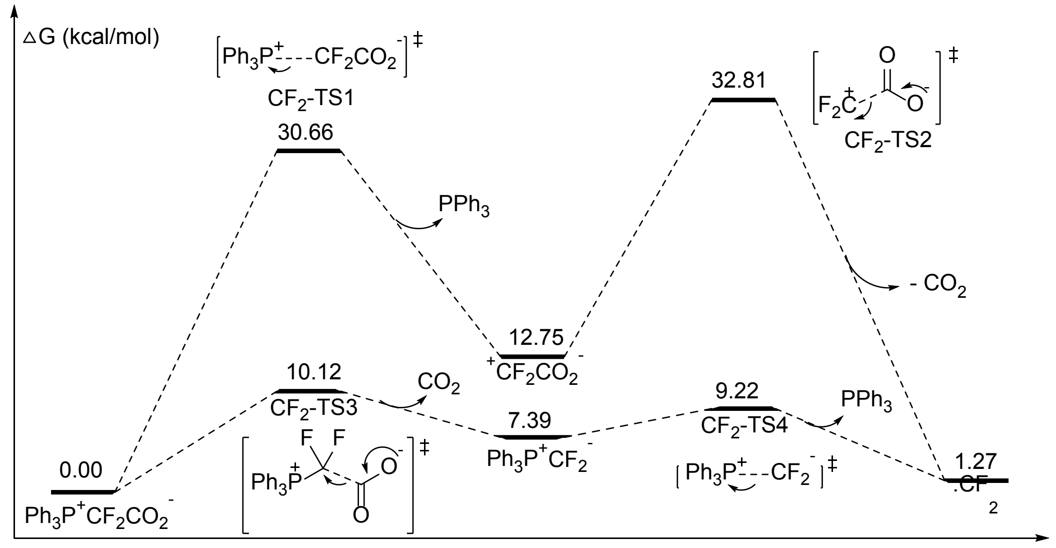

$Ph_3P^+CF_2CO_2^-$

C	1.87007800	-0.21322200	0.62982100
F	2.67854800	0.56870100	-0.11942900
F	2.28781300	-1.49742100	0.43064300
P	0.10234900	-0.06521000	-0.05902900
C	-0.76949100	1.13863400	0.94873500
C	-2.00124000	0.81445600	1.51719600
C	-0.18965600	2.39564400	1.15239800
C	-2.65678700	1.75943300	2.29963300
H	-2.44424200	-0.16294400	1.35947100
C	-0.85302500	3.32828800	1.93926400
H	0.76445300	2.64960600	0.69892500
C	-2.08325200	3.00964900	2.51148300
H	-3.61367000	1.51484000	2.74587500
H	-0.40939600	4.30315200	2.10466800
H	-2.59671500	3.74122100	3.12532400
C	-0.70588700	-1.67406500	-0.05476700
C	-1.52646900	-2.00796600	-1.13704000
C	-0.55338700	-2.54826700	1.02679300
C	-2.19431800	-3.22749000	-1.13468400
H	-1.64914300	-1.32959400	-1.97410700
C	-1.22314100	-3.76573500	1.01069800
H	0.07469700	-2.26901400	1.86536300

C	-2.03981700	-4.10506400	-0.06563200
H	-2.83106600	-3.49036300	-1.97134600
H	-1.10487500	-4.45154700	1.84163500
H	-2.55777800	-5.05763100	-0.07060400
C	0.24311700	0.52259800	-1.75623500
C	1.03652400	-0.20280100	-2.65382800
C	-0.45727700	1.65349600	-2.17770800
C	1.13098000	0.21769300	-3.97296100
H	1.57257300	-1.09023000	-2.33008900
C	-0.35716000	2.06287400	-3.50450900
H	-1.08148200	2.20969500	-1.48758400
C	0.43529000	1.34946700	-4.39702300
H	1.74632600	-0.33815900	-4.67076100
H	-0.90139300	2.93943400	-3.83618100
H	0.51119800	1.67270700	-5.42919000
C	1.92879500	0.11737100	2.16467300
O	2.76034000	0.95561200	2.50520100
O	1.10448000	-0.55714000	2.80278300

Sum of electronic and zero-point Energies= -1462.219326
Sum of electronic and thermal Energies= -1462.197632
Sum of electronic and thermal Enthalpies= -1462.196688
Sum of electronic and thermal Free Energies= -1462.272371

CF_2-TS1

C	2.87034700	-0.31195500	0.99401200
F	3.28084200	0.76950000	0.41520000
F	3.12269000	-1.36705700	0.28909300
P	0.32212500	-0.04997300	0.01094800
C	-0.75264200	1.12454900	0.91417400
C	-2.09673600	0.87821500	1.20056600
C	-0.17161900	2.33003500	1.32302700
C	-2.85158000	1.83110300	1.88182000
H	-2.56116300	-0.05292500	0.89242900
C	-0.93009000	3.28375100	1.99338700
H	0.87739600	2.52437400	1.11572900
C	-2.27190400	3.03386600	2.27528500
H	-3.89489200	1.63242100	2.10175500
H	-0.47243600	4.21680300	2.30342700
H	-2.86217000	3.77417100	2.80421800
C	-0.65509600	-1.59872900	-0.03105700
C	-1.47327400	-1.96666800	-1.10187600
C	-0.56477700	-2.43805500	1.08500300
C	-2.19706200	-3.15580500	-1.05138500
H	-1.55174800	-1.32819400	-1.97582700

C	-1.29672300	-3.62000800	1.13680300
H	0.07535400	-2.16363200	1.91877900
C	-2.11274200	-3.98095200	0.06690700
H	-2.83009500	-3.43488800	-1.88669400
H	-1.22411200	-4.26159600	2.00808300
H	-2.67866800	-4.90533100	0.10370700
C	0.25005400	0.54896300	-1.71965900
C	1.16749200	-0.00334500	-2.62095900
C	-0.66196000	1.50616800	-2.17028100
C	1.16030000	0.38274700	-3.95729000
H	1.88708400	-0.74195300	-2.27815500
C	-0.65982600	1.89873200	-3.50699900
H	-1.37851900	1.94575500	-1.48411200
C	0.24699700	1.33676200	-4.40140400
H	1.87171600	-0.05459400	-4.64923800
H	-1.37118800	2.64246100	-3.84929300
H	0.24486500	1.64328700	-5.44166300
C	2.58556800	-0.38231900	2.44433100
O	3.85577500	-0.46890800	2.47940000
O	1.65290400	-0.37356600	3.18559000

Sum of electronic and zero-point Energies= -1462.150543
Sum of electronic and thermal Energies= -1462.128232
Sum of electronic and thermal Enthalpies= -1462.127288
Sum of electronic and thermal Free Energies= -1462.206305

Ph_3P

P	0.49961500	-0.12313900	0.04850300
C	-0.61047400	1.04249000	0.94947400
C	-1.95383300	0.78983800	1.23488900
C	-0.05191100	2.26039300	1.35147800
C	-2.72312800	1.74115800	1.90045800
H	-2.40515400	-0.15029500	0.93614300
C	-0.82341700	3.21497400	2.00375200
H	0.99549000	2.46310300	1.14706600
C	-2.16276300	2.95548700	2.28156300
H	-3.76486500	1.53179500	2.11766200
H	-0.37744500	4.15697800	2.30315500
H	-2.76474800	3.69518500	2.79748800
C	-0.56553200	-1.62707700	-0.03021100
C	-1.40015400	-1.94451100	-1.10384700
C	-0.52970900	-2.48489500	1.07427900
C	-2.18892400	-3.09161300	-1.06729800
H	-1.43854800	-1.29551200	-1.97220900

C	-1.32710900	-3.62272100	1.11615000
H	0.12606800	-2.25623100	1.90949400
C	-2.15835400	-3.92947000	0.04231600
H	-2.83145300	-3.32770500	-1.90850000
H	-1.29289300	-4.27437500	1.98222500
H	-2.77533900	-4.82072600	0.06902100
C	0.33570900	0.50833000	-1.67699500
C	1.21474400	-0.01938600	-2.62842200
C	-0.60970300	1.45278100	-2.08281700
C	1.13718000	0.37009400	-3.96064500
H	1.96351100	-0.74429300	-2.32231300
C	-0.67871500	1.85233800	-3.41503700
H	-1.29741600	1.87809100	-1.35964500
C	0.18941600	1.30986500	-4.35646600
H	1.82178700	-0.05286000	-4.68750400
H	-1.41649100	2.58770500	-3.71693200
H	0.13179900	1.62121700	-5.39351100

Sum of electronic and zero-point Energies= -1035.909282
Sum of electronic and thermal Energies= -1035.894316
Sum of electronic and thermal Enthalpies= -1035.893372
Sum of electronic and thermal Free Energies= -1035.953899

$^{+}CF_2CO_2^{-}$

C	0.04074600	0.02059200	-0.49073900
F	-0.71032200	0.72389600	-1.30987100
F	0.86472800	-0.74015400	-1.17832400
C	-0.25389900	-0.17202600	0.91062700
O	0.64983600	0.76864000	0.55717700
O	-0.73206200	-0.60452100	1.89086100

Sum of electronic and zero-point Energies= -426.251113
Sum of electronic and thermal Energies= -426.246470
Sum of electronic and thermal Enthalpies= -426.245526
Sum of electronic and thermal Free Energies= -426.279444

CF_2-TS2

C	0.00231000	-0.35560600	-0.62046100
F	-1.03412900	-0.32663100	-1.34749900
F	1.04303000	-0.32142200	-1.34118300
C	-0.00355400	0.22218600	1.05156100
O	-0.01178400	1.39659900	0.76969300
O	0.00253800	-0.58953900	1.91463100

Sum of electronic and zero-point Energies= -426.221321
Sum of electronic and thermal Energies= -426.215819
Sum of electronic and thermal Enthalpies= -426.214874

Sum of electronic and thermal Free Energies= -426.251808

CO_2

C	0.00000000	0.00000000	0.00000000
O	0.00000000	0.00000000	1.15477400
O	0.00000000	0.00000000	-1.15477400

Sum of electronic and zero-point Energies= -188.564636
Sum of electronic and thermal Energies= -188.562013
Sum of electronic and thermal Enthalpies= -188.561068
Sum of electronic and thermal Free Energies= -188.585321

CF_2-TS3

C	1.79283800	-0.18636900	0.39787000
F	2.46329800	0.97156000	-0.03103800
F	2.44414900	-1.19634200	-0.33298600
P	0.10169800	-0.04925300	-0.37654600
C	-0.83244200	1.12351400	0.62746600
C	-2.22884800	1.07700700	0.61126600
C	-0.16425600	2.08953200	1.38359800
C	-2.95601300	2.00002600	1.35401400
H	-2.74751500	0.32498400	0.02468700
C	-0.90097600	3.00810000	2.12510200
H	0.91885600	2.13170400	1.39814400
C	-2.29227300	2.96358000	2.11022500
H	-4.03940900	1.96464400	1.34439500
H	-0.38550200	3.75774700	2.71450600
H	-2.86168900	3.68090200	2.69083000
C	-0.66053600	-1.67997300	-0.26854800
C	-1.54884100	-2.12433400	-1.25102100
C	-0.37226700	-2.48139100	0.84115400
C	-2.14378200	-3.37515800	-1.12306600
H	-1.77974300	-1.50288800	-2.10935300
C	-0.96944600	-3.73164900	0.95661100
H	0.30939500	-2.13086000	1.60831900
C	-1.85278800	-4.17803300	-0.02345200
H	-2.83393000	-3.72113800	-1.88402000
H	-0.74444400	-4.35625900	1.81362900
H	-2.31737900	-5.15329100	0.07113000
C	0.08327300	0.50658500	-2.09611700
C	0.62686700	-0.33522000	-3.07506600
C	-0.34551300	1.79253600	-2.43206500
C	0.72092500	0.10908700	-4.38792700
H	0.96480500	-1.33384700	-2.81645200
C	-0.24687100	2.22800800	-3.75062000

H	-0.75846200	2.45112900	-1.67533500
C	0.28330200	1.38883600	-4.72572500
H	1.13594600	-0.54364000	-5.14749000
H	-0.58549700	3.22372400	-4.01348600
H	0.35752300	1.73139400	-5.75186600
C	2.32358000	-0.22288400	2.85837900
O	3.34389800	0.31364800	2.70138400
O	1.38863200	-0.75494300	3.30487000

Sum of electronic and zero-point Energies= -1462.184854

Sum of electronic and thermal Energies= -1462.162405

Sum of electronic and thermal Enthalpies= -1462.161461

Sum of electronic and thermal Free Energies= -1462.239402

$Ph_3P^+CF_2^-$

C	2.36539900	-0.01101000	0.02074400
F	2.71886300	1.21875200	-0.58049000
F	2.69144500	-0.93362800	-0.99833700
P	0.49828400	0.00524400	0.01079300
C	-0.04192000	1.12818500	1.31595500
C	-1.35732300	1.03841500	1.78092100
C	0.82977200	2.08647100	1.83652000
C	-1.80012700	1.91403300	2.76460400
H	-2.03214900	0.28944400	1.37695900
C	0.37765400	2.95737500	2.82407700
H	1.85246100	2.15516600	1.48449600
C	-0.93243700	2.87234000	3.28551900
H	-2.81970000	1.84645200	3.12646600
H	1.05273400	3.70059200	3.23276800
H	-1.27922000	3.55249400	4.05560700
C	-0.03193300	-1.66821700	0.43217100
C	-1.17193700	-2.23344200	-0.14410200
C	0.69851900	-2.37514600	1.39403300
C	-1.57303800	-3.51050700	0.23541700
H	-1.74816300	-1.68491800	-0.88107400
C	0.29163600	-3.65235600	1.76246400
H	1.57492400	-1.93030800	1.85247000
C	-0.84134100	-4.21975000	1.18370700
H	-2.45814700	-3.94924600	-0.21095000
H	0.85857600	-4.20267400	2.50470400
H	-1.15636500	-5.21546100	1.47573500
C	-0.26515500	0.50458200	-1.54985300
C	-0.10192500	-0.32410100	-2.66800100
C	-0.88757500	1.74834100	-1.68258400

C	-0.58018000	0.08794800	-3.90564100
H	0.38686900	-1.28850100	-2.57279100
C	-1.36305500	2.15171700	-2.92702000
H	-1.00655400	2.39975100	-0.82338100
C	-1.21160800	1.32383400	-4.03508300
H	-0.45891100	-0.55564600	-4.76950500
H	-1.85257900	3.11369900	-3.02803900
H	-1.58351300	1.64184900	-5.00274500

Sum of electronic and zero-point Energies= -1273.614211
Sum of electronic and thermal Energies= -1273.595000
Sum of electronic and thermal Enthalpies= -1273.594056
Sum of electronic and thermal Free Energies= -1273.663721

CF_2-TS4

C	2.90840600	1.00292200	-0.63538300
F	3.32158200	0.35322100	-1.70744900
F	3.71095600	0.57368300	0.31584200
P	0.64742100	-0.14812900	-0.07367800
C	0.16362100	0.84873300	1.38741600
C	-0.52980100	0.31885000	2.47739100
C	0.55012700	2.19537500	1.41308200
C	-0.83319400	1.12183000	3.57409700
H	-0.84087900	-0.71999800	2.47345300
C	0.23856200	2.99333400	2.50684700
H	1.08932200	2.61538700	0.57096000
C	-0.45181200	2.45819200	3.59143800
H	-1.37378200	0.69917600	4.41375900
H	0.53865800	4.03528800	2.51356000
H	-0.69136100	3.08184900	4.44543300
C	-0.20406400	-1.75011900	0.22644400
C	-1.48741800	-2.04416500	-0.23917200
C	0.48814500	-2.71052400	0.97067500
C	-2.07117100	-3.27453000	0.04810000
H	-2.03224900	-1.31371500	-0.82749300
C	-0.10169300	-3.93432400	1.26730100
H	1.49324900	-2.49629800	1.32224900
C	-1.38307200	-4.21820700	0.80430000
H	-3.06742300	-3.49474200	-0.31920800
H	0.44267800	-4.66898800	1.84995800
H	-1.84161900	-5.17508500	1.02721800
C	-0.34365600	0.59815400	-1.42199000
C	0.05478900	0.32413100	-2.73297200
C	-1.47054700	1.39364100	-1.20250700

C	-0.67192600	0.82282700	-3.80882300
H	0.93906100	-0.27973600	-2.91255100
C	-2.19294300	1.89651100	-2.28003700
H	-1.78423100	1.62000100	-0.18871200
C	-1.79657400	1.61016200	-3.58300000
H	-0.35454700	0.60387400	-4.82211200
H	-3.06677400	2.51311700	-2.10088800
H	-2.36048000	2.00470300	-4.42089000

Sum of electronic and zero-point Energies= -1273.599007
Sum of electronic and thermal Energies= -1273.582119
Sum of electronic and thermal Enthalpies= -1273.581175
Sum of electronic and thermal Free Energies= -1273.645649

:CF_2

C	0.00000000	0.00000000	-0.59144200
F	0.00000000	-1.02532000	0.19829300
F	0.00000000	1.02532000	0.19829300

Sum of electronic and zero-point Energies= -237.682620
Sum of electronic and thermal Energies= -237.679635
Sum of electronic and thermal Enthalpies= -237.678691
Sum of electronic and thermal Free Energies= -237.705995
