# Supplementary material for: Oxidation of difluorocarbene and subsequent trifluoromethoxylation
Source: Nat Commun. 2019 Nov 25;10:5362. doi: 10.1038/s41467-019-13359-z (PMC6877537; doi:10.1038/s41467-019-13359-z)
Supplement: Supplementary file 5 — Supplementary Data 2 [file 41467_2019_13359_MOESM5_ESM.pdf]

DFT calculations: optimized geometrical coordinates and calculated total energies. All energies are presented with atomic unit.

Relative free energies for trifluoromethoxylation

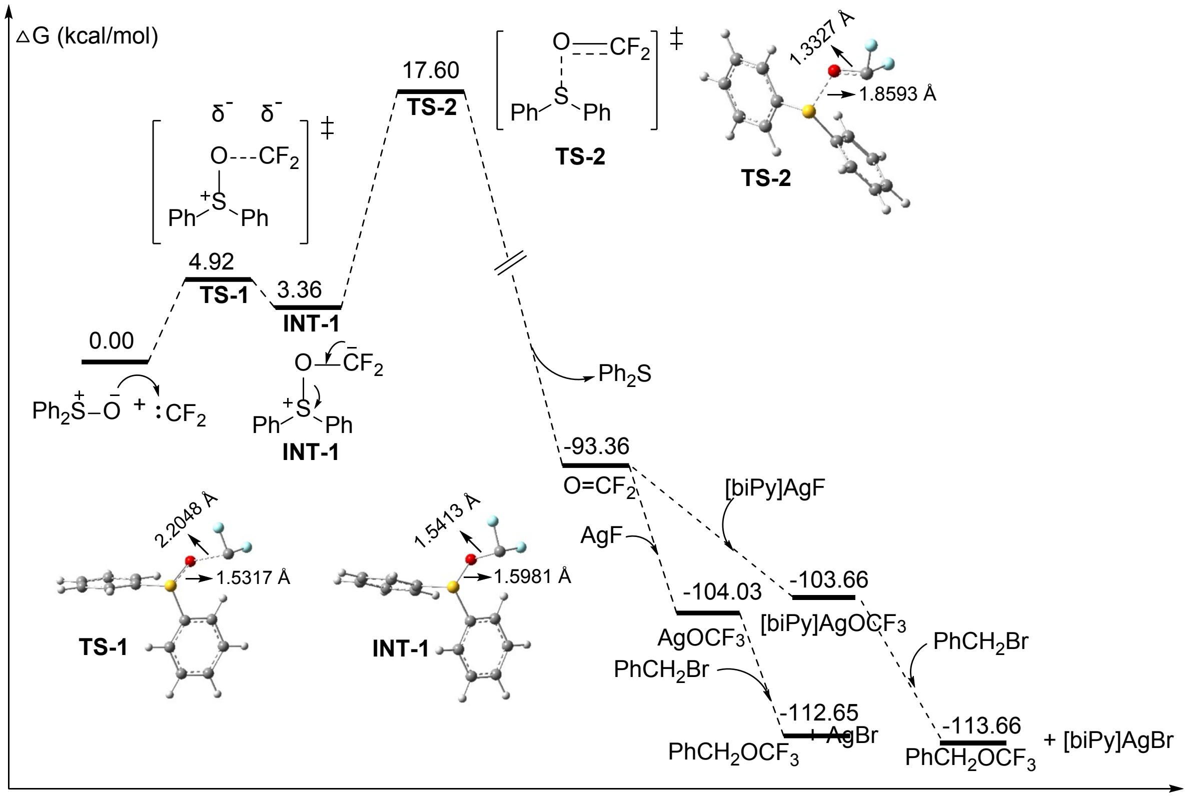

Ph_2SO

C	-0.32265278	0.26676991	-1.35343222
C	-0.99771962	-0.88316252	-1.74646483
C	0.83716969	0.69140872	-1.98420155
C	-0.47518625	-1.64202428	-2.78862488
H	-1.91666079	-1.18394749	-1.25378995
C	1.34810571	-0.07508663	-3.02857286
H	1.31771155	1.60860013	-1.66229122
C	0.69622173	-1.23900446	-3.42668693
H	-0.98764182	-2.54199109	-3.10775946
H	2.25403195	0.24076363	-3.53275375
H	1.09637143	-1.83042722	-4.24204628
C	-0.31119206	0.28422569	1.35640073
C	-1.03162470	-0.80569956	1.83024261
C	0.90562516	0.65872555	1.90780366
C	-0.49848286	-1.55794058	2.87257649
H	-1.99441498	-1.06314879	1.40098965
C	1.42584633	-0.09871163	2.95321098

H	1.42254571	1.53206388	1.52568568
C	0.72793852	-1.20534175	3.43096472
H	-1.04649692	-2.41129818	3.25442420
H	2.37511102	0.17821154	3.39689122
H	1.13635648	-1.79023798	4.24690971
S	-0.98992244	1.27287582	-0.00175010
O	-0.20530598	2.56630597	-0.01719423

Sum of electronic and zero-point Energies= -936.394756
Sum of electronic and thermal Energies= -936.383141
Sum of electronic and thermal Enthalpies= -936.382197
Sum of electronic and thermal Free Energies= -936.434316

TS-1

C	-0.17854734	0.04732058	-1.58526222
C	-1.00880118	0.26126003	-2.67970540
C	1.08904483	-0.51862439	-1.70522880
C	-0.55005827	-0.10874124	-3.94122421
H	-1.98928321	0.70692082	-2.55541899
C	1.53251423	-0.87178025	-2.97078302
H	1.71274942	-0.67796323	-0.83292784
C	0.71380989	-0.66938181	-4.08299857
H	-1.18115609	0.04654015	-4.80757900
H	2.51638667	-1.30873601	-3.09076546
H	1.06836974	-0.95270628	-5.06714573
C	-0.38780242	-0.83843142	1.03525178
C	-1.14687954	-1.99233876	0.86703901
C	0.62904812	-0.73828626	1.97290739
C	-0.85495237	-3.09427298	1.66026072
H	-1.94464326	-2.03759530	0.13266811
C	0.90236312	-1.85362123	2.76177975
H	1.19114653	0.17960398	2.08921161
C	0.16784637	-3.02383226	2.60464666
H	-1.43033155	-4.00460109	1.54349167
H	1.69237465	-1.79945993	3.50105470
H	0.38749728	-3.88544456	3.22400641
S	-0.80585438	0.56389401	-0.00149657
O	0.28461192	1.66114714	0.39962527
C	-0.28915913	2.48643434	1.56812398
F	-0.32542949	3.73659137	0.96882167
F	0.86799689	2.58649019	2.32690112

Sum of electronic and zero-point Energies= -1174.085665
Sum of electronic and thermal Energies= -1174.070352
Sum of electronic and thermal Enthalpies= -1174.069408
Sum of electronic and thermal Free Energies= -1174.132468

INT-1

C	-0.17854734	0.04732058	-1.58526222
C	-1.00880118	0.26126003	-2.67970540
C	1.08904483	-0.51862439	-1.70522880
C	-0.55005827	-0.10874124	-3.94122421
H	-1.98928321	0.70692082	-2.55541899
C	1.53251423	-0.87178025	-2.97078302
H	1.71274942	-0.67796323	-0.83292784
C	0.71380989	-0.66938181	-4.08299857
H	-1.18115609	0.04654015	-4.80757900
H	2.51638667	-1.30873601	-3.09076546
H	1.06836974	-0.95270628	-5.06714573
C	-0.38780242	-0.83843142	1.03525178
C	-1.14687954	-1.99233876	0.86703901
C	0.62904812	-0.73828626	1.97290739
C	-0.85495237	-3.09427298	1.66026072
H	-1.94464326	-2.03759530	0.13266811
C	0.90236312	-1.85362123	2.76177975
H	1.19114653	0.17960398	2.08921161
C	0.16784637	-3.02383226	2.60464666
H	-1.43033155	-4.00460109	1.54349167
H	1.69237465	-1.79945993	3.50105470
H	0.38749728	-3.88544456	3.22400641
S	-0.80585438	0.56389401	-0.00149657
O	0.28461192	1.66114714	0.39962527
C	-0.28915913	2.48643434	1.56812398
F	-0.32542949	3.73659137	0.96882167
F	0.86799689	2.58649019	2.32690112

Sum of electronic and zero-point Energies= -1174.090222
Sum of electronic and thermal Energies= -1174.075191
Sum of electronic and thermal Enthalpies= -1174.074246
Sum of electronic and thermal Free Energies= -1174.134960

TS-2

C	0.15664883	-0.42613582	-1.68404242
C	-0.85246894	-0.24364236	-2.62850800
C	1.49663616	-0.17402760	-1.97184695
C	-0.50408220	0.23728196	-3.88517778
H	-1.88608185	-0.45806738	-2.38617617
C	1.82296321	0.29608478	-3.23797805
H	2.26952921	-0.34361253	-1.23235360
C	0.82731928	0.50593667	-4.18866307
H	-1.27745491	0.39788044	-4.62636478
H	2.85966602	0.49738588	-3.47895600

H	1.09150245	0.87589831	-5.17226705
C	0.76672029	-0.45856143	1.05463383
C	1.10511063	-1.30333890	2.11170387
C	1.14484997	0.88848960	1.03014505
C	1.89250341	-0.79967412	3.14168188
H	0.76864870	-2.33357335	2.12383890
C	1.92495667	1.37164971	2.06668508
H	0.82670900	1.53865984	0.22358631
C	2.30250425	0.52796887	3.11398440
H	2.17380358	-1.44361498	3.96558188
H	2.22984212	2.41100252	2.06821009
H	2.91178703	0.91820597	3.92095384
S	-0.34470735	-1.10345774	-0.12508233
O	-1.78380284	0.04862121	0.11726233
C	-2.18452957	0.11980797	1.38631383
F	-3.56381585	-0.16050716	1.37392153
F	-2.16696227	1.48885753	1.71536462

Sum of electronic and zero-point Energies= -1174.068036
Sum of electronic and thermal Energies= -1174.053375
Sum of electronic and thermal Enthalpies= -1174.052430
Sum of electronic and thermal Free Energies= -1174.112247

Ph_2S

C	0.06511160	0.52830176	-1.38905278
C	-0.69710699	0.82983754	-2.51656545
C	0.90223145	-0.58947585	-1.38832409
C	-0.61683315	0.01660381	-3.64488537
H	-1.35414983	1.69221222	-2.51108077
C	0.95927072	-1.40741175	-2.51022959
H	1.50137070	-0.81905966	-0.51401659
C	0.20444802	-1.10533373	-3.64291590
H	-1.21041104	0.25717915	-4.51941422
H	1.60532535	-2.27791109	-2.50338689
H	0.25795644	-1.74293874	-4.51758527
C	-0.06511804	0.53061417	1.38935301
C	-0.91140757	-0.58024424	1.39353878
C	0.70669527	0.82573097	2.51183537
C	-0.96822945	-1.39806915	2.51531330
H	-1.51664464	-0.80510548	0.52226729
C	0.62682201	0.01258037	3.64037584
H	1.37065177	1.68283113	2.50241011
C	-0.20411088	-1.10217026	3.64334744
H	-1.62105260	-2.26356712	2.51229460
H	1.22731100	0.24828366	4.51149835

H	-0.25721410	-1.73948976	4.51830213
S	-0.00086131	1.65011150	-0.00075200

Sum of electronic and zero-point Energies= -861.226515
Sum of electronic and thermal Energies= -861.215841
Sum of electronic and thermal Enthalpies= -861.214897
Sum of electronic and thermal Free Energies= -861.265564

CF_2O

C	0.00000000	0.00000000	0.13895337
O	0.00000000	0.00000000	1.30709438
F	0.00000000	1.05963500	-0.62724863
F	0.00000000	-1.05963500	-0.62724863

Sum of electronic and zero-point Energies= -312.997805
Sum of electronic and thermal Energies= -312.994535
Sum of electronic and thermal Enthalpies= -312.993590
Sum of electronic and thermal Free Energies= -313.023593

AgF

F	0.00000000	0.00000000	1.82010773
Ag	0.00000000	0.00000000	-0.34853127

Sum of electronic and zero-point Energies= -245.541322
Sum of electronic and thermal Energies= -245.538600
Sum of electronic and thermal Enthalpies= -245.537656
Sum of electronic and thermal Free Energies= -245.564808

[biPy]AgF

C	0.45639500	0.70404100	0.20890500
C	1.18230200	1.35447500	-0.78936700
C	1.65651400	2.63733200	-0.54955500
C	1.39798000	3.23712300	0.67509600
C	0.68108900	2.51375000	1.61993700
C	-0.09022700	-0.67485400	0.01208300
C	-0.25845200	-1.22097900	-1.26059700
C	-0.76287200	-2.50906800	-1.37973300
C	-1.08758800	-3.21786300	-0.23150000
C	-0.90425900	-2.59464400	0.99637300
H	1.39722800	0.86626100	-1.73024300
H	2.22658100	3.15663200	-1.31089400
H	1.74642400	4.23583200	0.90456500
H	0.46207900	2.93456000	2.59530000
H	-0.02205000	-0.64897300	-2.14753600
H	-0.90486400	-2.94834000	-2.36016400
H	-1.48162800	-4.22491400	-0.27683000
H	-1.15251500	-3.10326600	1.92158200
N	0.22525900	1.28139100	1.39428900

N	-0.42186900	-1.35725100	1.11532900
F	-1.10684200	-0.18404800	5.28544900
Ag	-0.58318400	-0.12904200	3.18285000

Sum of electronic and zero-point Energies= -740.738359
Sum of electronic and thermal Energies= -740.725516
Sum of electronic and thermal Enthalpies= -740.724572
Sum of electronic and thermal Free Energies= -740.781462

$AgOCF_3$

O	-0.90167907	0.02689840	0.60712325
C	-0.09959109	0.00307097	1.56238020
F	-0.67589837	-0.07901121	2.80877603
F	0.73934657	1.10984639	1.66759943
F	0.80583050	-1.05754462	1.55593649
Ag	-0.00026658	0.00014408	-1.45791648

Sum of electronic and zero-point Energies= -558.572034
Sum of electronic and thermal Energies= -558.565889
Sum of electronic and thermal Enthalpies= -558.564945
Sum of electronic and thermal Free Energies= -558.605421

[biPy]$AgOCF_3$

C	0.39553700	0.66521800	0.07293700
C	1.36688500	1.26465700	-0.72898300
C	1.79624200	2.54888200	-0.42209400
C	1.25039800	3.20102700	0.67474700
C	0.29976600	2.52741100	1.43083800
C	-0.11045200	-0.71410100	-0.21243900
C	0.05594400	-1.30813400	-1.46361800
C	-0.42159100	-2.59605200	-1.66668900
C	-1.05253300	-3.25706100	-0.62197600
C	-1.19179000	-2.58776800	0.58716000
H	1.79924300	0.73468200	-1.56672100
H	2.55347000	3.02868200	-1.03090000
H	1.55402500	4.20259700	0.95067400
H	-0.14828000	2.99078900	2.30298800
H	0.53310100	-0.77369600	-2.27374100
H	-0.30488700	-3.07305700	-2.63265000
H	-1.43713400	-4.26254300	-0.73430000
H	-1.68563100	-3.05815600	1.43043900
N	-0.11515300	1.29382700	1.13957300
N	-0.73620900	-1.35037200	0.78560000
F	-0.60491000	-0.05589000	5.60895100
Ag	-1.38866200	-0.02512300	2.68680600
C	-2.00142700	-0.04679700	5.67860600
O	-2.58958500	-0.10900000	4.58432600

F	-2.27272200	-1.07932400	6.56199300
F	-2.26059600	1.08545200	6.43774900

Sum of electronic and zero-point Energies= -1053.770804
Sum of electronic and thermal Energies= -1053.754064
Sum of electronic and thermal Enthalpies= -1053.753120
Sum of electronic and thermal Free Energies= -1053.821479

$PhCH_2Br$

C	-1.20558607	-1.28388162	-0.30599530
C	-1.20622144	-2.56903965	0.22484371
C	-0.00061217	-3.21408773	0.49067494
C	1.20551773	-2.56943170	0.22662450
C	1.20571220	-1.28446575	-0.30438559
C	0.00034992	-0.63269268	-0.57230201
H	-2.14340065	-0.77770325	-0.51186796
H	-2.14616898	-3.06977727	0.42647817
H	-0.00108154	-4.21666826	0.90272662
H	2.14528375	-3.07000168	0.42970082
H	2.14387012	-0.77873696	-0.50948797
C	0.00227792	0.73987998	-1.14578545
H	0.89896476	0.95883768	-1.71743754
H	-0.89041098	0.96138256	-1.72252127
Br	-0.00044814	2.13928520	0.31486744

Sum of electronic and zero-point Energies= -283.919679
Sum of electronic and thermal Energies= -283.912683
Sum of electronic and thermal Enthalpies= -283.911738
Sum of electronic and thermal Free Energies= -283.953010

AgBr

Br	0.00000000	0.00000000	1.49335679
Ag	0.00000000	0.00000000	-1.11207421

Sum of electronic and zero-point Energies= -158.861752
Sum of electronic and thermal Energies= -158.858830
Sum of electronic and thermal Enthalpies= -158.857885
Sum of electronic and thermal Free Energies= -158.887376

[biPy]AgBr

C	0.00067700	0.76094100	-2.12497600
C	0.19742400	1.50045100	-3.29140000
C	0.18322000	2.88703500	-3.22019300
C	-0.02327100	3.49979300	-1.99203400
C	-0.19417700	2.68957200	-0.87660900
C	-0.00480600	-0.73514900	-2.13646600
C	-0.20651900	-1.45650100	-3.31331800
C	-0.19185300	-2.84399300	-3.26384300

C	0.02008000	-3.47577000	-2.04631800
C	0.19571600	-2.68311200	-0.91912000
H	0.37770300	1.00869900	-4.23763400
H	0.33884600	3.47831200	-4.11490800
H	-0.04389900	4.57709600	-1.88995500
H	-0.34745300	3.12067000	0.10690100
H	-0.39101500	-0.95000300	-4.25094100
H	-0.35115900	-3.42136100	-4.16695900
H	0.04138600	-4.55455000	-1.96124800
H	0.35331000	-3.12961300	0.05678900
N	-0.18010900	1.35795500	-0.94037800
N	0.18096000	-1.35058800	-0.96200600
Ag	0.00691100	-0.01519300	1.04227900
Br	0.01012500	-0.01895000	3.65997000

Sum of electronic and zero-point Energies= -654.061171
Sum of electronic and thermal Energies= -654.048070
Sum of electronic and thermal Enthalpies= -654.047125
Sum of electronic and thermal Free Energies= -654.105641

$PhCH_2OCF_3$

C	-1.09106785	-1.62879140	-0.30869679
C	-1.34367247	-2.95267359	0.02846965
C	-0.28538195	-3.80994382	0.32716910
C	1.02241385	-3.33849609	0.29073606
C	1.27405540	-2.00882824	-0.04051967
C	0.22010695	-1.15016019	-0.34255084
H	-1.91219499	-0.95988226	-0.54651540
H	-2.36358322	-3.31879109	0.05368616
H	-0.48241867	-4.84395684	0.58633134
H	1.84719249	-4.00237100	0.52262876
H	2.29308014	-1.63681212	-0.06462874
C	0.48282056	0.28217383	-0.69988768
H	1.54830232	0.47038863	-0.83575322
H	-0.06226880	0.57122981	-1.60087600
O	0.00744385	1.10225181	0.40632466
C	-0.05588598	2.40470039	0.16147292
F	-0.44811372	3.03271191	1.26558256
F	1.12452381	2.93007076	-0.21322012
F	-0.92840911	2.71658345	-0.81487652

Sum of electronic and zero-point Energies= -683.646215
Sum of electronic and thermal Energies= -683.636150
Sum of electronic and thermal Enthalpies= -683.635206
Sum of electronic and thermal Free Energies= -683.684786
